# Flavinium Catalysed Photooxidation: Detection and Characterization of Elusive Peroxyflavinium Intermediates

**DOI:** 10.1002/anie.201906293

**Published:** 2019-08-23

**Authors:** Jan Zelenka, Radek Cibulka, Jana Roithová

**Affiliations:** ^1^ Department of Spectroscopy and Catalysis Institute for Molecules and Materials Radboud University Nijmegen Heyendaalseweg 135 6525 AJ Nijmegen The Netherlands; ^2^ Department of organic chemistry Faculty of Chemical Technology University of Chemistry and Technology Prague Technická 5 166 28 Prague 6 Czech Republic

**Keywords:** flavin, ion spectroscopy, mass spectrometry, peroxy intermediates, photooxidation

## Abstract

Flavin‐based catalysts are photoactive in the visible range which makes them useful in biology and chemistry. Herein, we present electrospray‐ionization mass‐spectrometry detection of short‐lived intermediates in photooxidation of toluene catalysed by flavinium ions (Fl^+^). Previous studies have shown that photoexcited flavins react with aromates by proton‐coupled electron transfer (PCET) on the microsecond time scale. For Fl^+^, PCET leads to FlH^.+^ with the H‐atom bound to the N5 position. We show that the reaction continues by coupling between FlH^.+^ and hydroperoxy or benzylperoxy radicals at the C4a position of FlH^.+^. These results demonstrate that the N5‐blocking effect reported for alkylated flavins is also active after PCET in these photocatalytic reactions. Structures of all intermediates were fully characterised by isotopic labelling and by photodissociation spectroscopy. These tools provide a new way to study reaction intermediates in the sub‐second time range.

## Graphical abstract

**Active site switch occurred** after N5‐hydrogen blocking which resulted from proton coupled electron transfer. The short‐lived peroxy‐flavinium reaction intermediates were characterised by mass spectrometry and ion spectroscopy. Although the effect of N5‐hydrogen blocking was predicted more than 40 years ago, this is the first direct evidence.

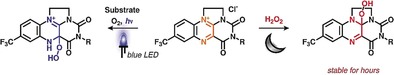

## Introduction

Hydroperoxyflavins mediate flavin‐based oxidations involving oxygen atom transfer.^1^, ^2^ In biological systems, these oxidations use O_2_ as a source of oxygen.^3^ The chemoselectivity of hydroperoxyflavins prompted their development as oxidants in organic synthesis.^4^ Recently, researchers shifted their focus towards flavin‐photocatalysed oxidation reactions and developed procedures for photooxidation of substituted toluenes, benzyl alcohols and benzaldehydes using O_2_ as the terminal oxidant.^5^, ^6^ The mechanism of the flavin derivative catalysed photooxidation of benzyl‐alcohols has been thoroughly studied by femtosecond transient absorption measurements (UV/Vis)^7^ and by NMR.^8^ Both studies agree that this photocatalytic reaction is initiated by electron transfer from a substrate to the flavin moiety, thus mimicking the predominant enzymatic mechanism. The results also show that the catalyst regenerates in a reaction with oxygen. However, the nature of the interaction between oxygen and the reduced flavin in photoreactions as well as the structure of the possible oxo‐intermediates remains unknown. Therefore, this study aims to identify as many intermediates as possible for a deeper understanding of flavin photocatalysis.

**Figure 1:**
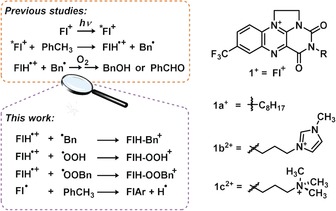
Flavinium photooxidation pathways.

To provide a new perspective into flavin photocatalysis, we investigated intermediates of the recently published flavinium (**1 a** ^+^) photocatalytic oxidation of substituted toluenes (Figure 1).^9^ The previous results indicate that flavinium photocatalysis most likely proceeds through the proton coupled electron transfer (PCET) mechanism. PCET transforms toluene into the benzyl radical which was detected by trapping with TEMPO. The other PCET product, the one‐electron reduced protonated flavin radical, has been detected by EPR. Herein, we report detection of further intermediates of this photooxidation reaction by electrospray ionization mass spectrometry (ESI‐MS) and their characterization by infrared photodissociation (IRPD) spectroscopy.^10^ The ESI‐MS approach cannot address processes on the sub‐millisecond time scale. Therefore, we aimed at detecting intermediates formed by subsequent reactions after the initial PCET. We note that NMR, UV/Vis or EPR spectroscopies have failed or would most probably fail to detect these intermediates.^9^

## Results

**Figure 2:**
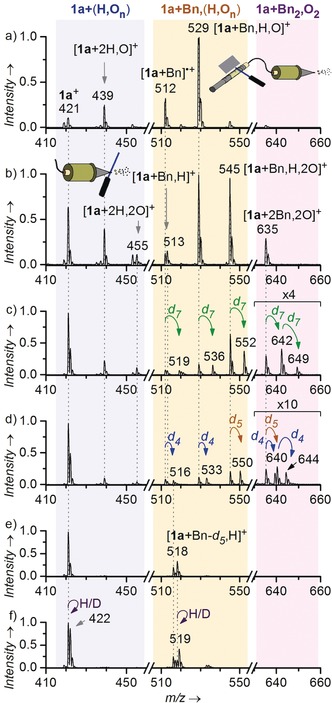
Flavinium ions and their oxidised derivatives detected by ESI‐MS. The dominant signals are denoted with the respected *m*/*z* ratio and tentative structure. Solution of toluene and **1 a** ^+^ (10 mol %) in acetonitrile was irradiated a) in a vial (445 nm LEDs, 30 seconds) and b) at the capillary tip (445 nm laser diode, continuous). c) Solution of toluenes *d_0_* and *d_8_* (1:1) and **1 a** ^+^ (2 mol % to the sum toluene concentration) irradiated at the capillary tip (445 nm laser diode, continuous). d) Same as c) with toluenes *d_0_* and *d_5_*. e) Toluene‐*d_5_* and **1 a** ^+^ (4 mol %) in dry acetonitrile, irradiated in a Schlenk tube (445 nm LEDs, 5 min, under Ar). f) Mixture from e, after addition of 10 μl of D_2_O.

Photochemical reactions involve intermediates with a large internal energy. Accordingly, their half‐life is usually rather short. In order to determine which intermediates we could detect at various reaction times, we infused acetonitrile solution of **1 a** ^+^ and toluene directly into the mass spectrometer through a silica capillary and triggered the photochemical reaction in various time delays from the ESI‐MS detection. We varied the reaction time by irradiating the infused solution either in the vial, along the silica capillary, or at the tip of the capillary (Figure 2 and Figures S2, S15, S16 in the Supporting Information).^11^ The irradiation at the tip of the capillary, right before it evaporates into the gas phase, allowed us to observe short‐lived intermediates. These intermediates were formal adducts of flavinium cation (**1 a** ^+^) with: hydrogen peroxide, [**1 a**+2 H,2 O]^+^ (*m*/*z* 455), benzylhydroperoxide, [**1 a**+Bn,H,2 O]^+^ (*m*/*z* 545), and dibenzylperoxide, [**1 a**+2 Bn,2 O]^+^ (*m*/*z* 635) (Figure 2 b). Signals of these intermediates disappear if the irradiation is blocked or if we irradiate further along the capillary, thereby prolonging the reaction time.

Furthermore, we observed formal adducts of **1 a^+^** with: water, [**1 a**+2 H,O]^+^ (*m*/*z* 439), benzyl radical, [**1 a**+Bn]^.+^ (*m*/*z* 512), toluene, [**1 a**+Bn,H]^+^ (*m*/*z* 513), toluene and oxygen, [**1 a**+Bn,H,O]^+^ (*m*/*z* 529) and minor signals of oxidised flavinium, [**1 a**+O]^+^ (*m*/*z* 437) and [**1 a**+2 O]^+^ (*m*/*z* 453). These signals are observed also at longer reaction times (irradiation in the syringe, or along the capillary, Figure 2 a, Figure S16 in the Supporting Information) and they are also detected after irradiation is interrupted. Hence, these ions probably correspond to long‐lived intermediates, or oxidised by‐products.

The benzyl containing intermediates clearly originate from the reaction between the photoexcited catalyst and toluene, because they are absent if the reaction mixture is not irradiated. The hydroperoxy intermediate, [**1 a**+2 H,2 O]^+^, could theoretically also stem from a dark reaction between **1 a** ^+^ and H_2_O_2_ formed during the photocatalytic reaction.^9^ Nevertheless, a control dark reaction of **1 a** ^+^ with H_2_O_2_ did not yield any signal of [**1 a**+2 H,2 O]^+^, confirming it to be exclusively a product of photoreaction.

According to the previous studies, the dark reaction with H_2_O_2_ leads to neutral complex [**1 a**+H,2 O].^4^, ^9^ To probe if we could detect this complex, we have prepared a charge‐tagged flavinium ion **1 b** ^2+^ (Supporting Information, Figure S1).^12^ The remote imidazolium group keeps the intermediates cationic, even when the flavinium reaction site is neutralised. In the dark reaction of H_2_O_2_ with **1 b** ^2+^ we have indeed observed the signal of [**1 b**+H,2 O]^+^ (**1 b** ^2+^ + HO_2_^−^, Figure S6 b in the Supporting Information). Hence, we conclude that the dark reaction with H_2_O_2_ yields neutral intermediate [**1 a**+H,2 O], but this intermediate is not easily protonated and thus it is not detected by ESI‐MS. Therefore, the signal of [**1 a**+2 H,2 O]^+^ is a signature of an intermediate formed in the photoreaction.

Next, we investigated the C−H bond activation of toluene by experiments with isotopically labelled substrates. First, we irradiated a reaction mixture containing equimolar ratio of C_6_H_5_−CH_3_ and C_6_D_5_−CD_3_ (Figure 2 c). We noticed that all signals containing per‐deuterated benzyl group are only *d_7_*‐labelled. The intensities of [**1 a**+Bn,H,2 O]^+^ (*m*/*z* 545) and [**1 a**+Bn‐*d_7_*,H,2 O]^+^ (*m*/*z* 552) signals imply that the C−H activation step is associated with kinetic isotope effect (KIE) of approximately 1.5 (the correct value is 1.6±0.1, see the results below and Figure S31 in the Supporting Information). Surprisingly, however, this KIE is not retained for the photooxidation by‐products [**1 a**+Bn]^+^ (*m*/*z* 512 and 519) and [**1 a**+Bn,H,O]^+^ (*m*/*z* 529 and 536). These ions are formed with KIE closer to 1 (the correct value is 1.2±0.1, see the explanation accompanying Figure S31 in the Supporting Information) suggesting their formation through a different reaction pathway than the peroxy‐intermediates.

We repeated the experiments with a reaction mixture containing equimolar ratio of C_6_H_5_−CH_3_ and C_6_D_5_−CH_3_ (Figure 2 d). In this case, the energy barrier for benzylic C−H activation is equivalent for both isotopologues. Accordingly, the signals of the intermediates [**1 a**+Bn,H,2 O]^+^ (*m*/*z* 545) and [**1 a**+Bn‐*d_5_*,H,2 O]^+^ (*m*/*z* 550) are in the 1:1 ratio. On the contrary, the signals of the photooxidation by‐products, [**1 a**+Bn]^+^ (*m*/*z* 512 and 516) and [**1 a**+Bn,H,O]^+^ (*m*/*z* 529 and 533) are not equally abundant and reveal KIE of 1.4±0.1 and 1.2±0.1, respectively. The *m*/*z* shift of 4 instead of 5 in these ions suggests that these by‐products result from an activation of the aromatic C−H/C−D bond rather than the benzylic C−H bond (Figure 2 d). The possibility to activate toluene at two sites reveals itself also in the “dibenzyl peroxy” intermediates [**1 a**+2 Bn,2 O]^+^. These ions contain one benzyl group and one tolyl group. We further confirmed the aromatic C−H bond activation by an experiment with benzene (Supporting Information, Figure S12 h).

Finally, we performed the reaction with C_6_D_5_−CH_3_ in a Schlenk tube under anaerobic and dry conditions (freeze‐pump‐thaw, argon). The aim was to track possible products of the initial hydrogen/deuterium atom transfer in the photochemical reaction. After irradiation for 5 min in the tube, we sprayed the solution and detected signals of **1 a** ^+^ (*m*/*z* 421), [**1 a**+Bn‐*d_4_*]^+^ (*m*/*z* 516), and [**1 a**+Bn‐*d_5_*,H]^+^ (*m*/*z* 518) (Figure 2 e). We tested the number of exchangeable (acidic) hydrogen atoms in these ions by addition of a small amount of D_2_O to the sample (Figure 2 f). All the signals partially shifted by Δ*m/z+*1. Such shift is expected for [**1 a**+Bn‐*d_5_*,H]^+^ (*m*/*z* 518, forming [**1 a**+Bn‐*d_5_*,D]^+^ with *m*/*z* 519) and its dehydrogenated analogue [**1 a**‐H_2_+Bn‐*d_5_*,H]^+^ (*m*/*z* 516, forming [**1 a**‐H_2_+Bn‐*d_5_*,D]^+^ with *m*/*z* 517). The hydrogen exchange in “**1 a** ^+^” is uniquely associated with anaerobic conditions, as we did not observe such reactivity under aerobic conditions. Accordingly, the anaerobic conditions lead probably to formation of deprotonated neutral [**1 a**‐H] that we detect in this case as deuterated ions [**1 a**‐H+D]^+^.

### Gas‐phase reactivity of the detected intermediates

**Figure 3:**
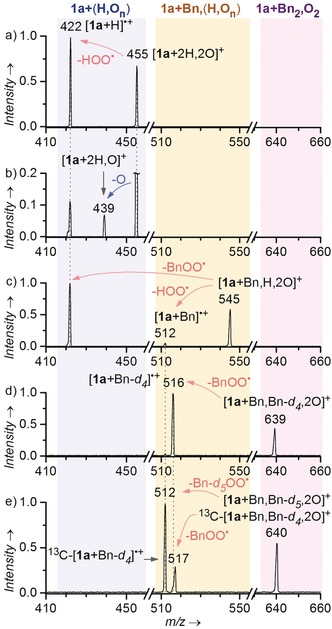
a) Collision‐induced dissociation (CID) of [**1 a**+2 H,2 O]^+^ (p(Xe)=0.20 mtorr, *E*_CM_=5.6 eV). b) Reactivity of [**1 a**+2 H,2 O]^+^ with dimethyl sulphide (*E*_CM_=0 eV, p(Me_2_S)=0.68 mtorr). For pressure, collision energy dependencies and analogous experiments with [**1 a**+H,2 O]^+^ please refer to the Supporting Information. c) CID of [**1 a**+Bn,H,2 O]^+^ (p(Xe)=0.1 mtorr (nom.), *E*_CM_=4.7 eV). d) CID of [**1 a**+C_6_D_4_−CH_3_, C_6_H_5_−CH_2_O_2_]^+^ (p(Xe)=0.1 mtorr (nom.), *E*_CM_=4.2 eV). e) CID of [**1 a**+C_6_H_4_−CH_3_, C_6_D_5_−CH_2_O_2_]^+^ with isobaric ^13^C isotopologue of [**1 a**+C_6_D_4_−CH_3_, C_6_H_5_−CH_2_O_2_]^+^ (p(Xe)=0.1 mtorr (nom.), *E*_CM_=4.2 eV).

We have probed unimolecular and bimolecular reactivity of the detected intermediates in the gas phase. In the collisional experiments with xenon gas, the short‐lived peroxy‐intermediates [**1 a**+2 H,2 O]^+^ and [**1 a**+Bn,H,2 O]^+^ readily lost hydroperoxy or benzylperoxy radicals (Figure 3 a,c). Fragmentation of a closed‐shell organic ion into two radicals is unusual and reflects a very weak C−O (or N−O) bond in the [**1 a**+2 H,2 O]^+^ intermediate. Expected elimination of H_2_O_2_ or BnOOH appears only as a minor channel at low collision energies and is thus most probably kinetically suppressed in the gas phase. However, this channel might be important in solution as it corresponds to generation of hydrogen peroxide (or benzylhydroperoxide) and regeneration of **1 a** ^+^ in the photocatalytic cycle. To test the oxygen atom transfer ability of the peroxy‐intermediates, we collided them with dimethyl sulphide at thermal conditions (with zero collision energy). The intermediates [**1 a**+2 H,2 O]^+^ (Figure 3 b) and [**1 a**+Bn,H,2 O]^+^ transfer one oxygen atom to dimethyl sulphide. The reaction is however very sluggish and accompanied by more facile losses of hydroperoxy or benzylperoxy radicals.

Collisional experiments with [**1 a**+2 Bn,2 O]^+^ show exclusive elimination of benzylperoxy radical. Intermediates from photooxidation of a mixture of toluene‐*d_0_* and toluene‐*d_5_* provide the most illustrative picture. The [**1 a**+C_6_D_4_−CH_3_, C_6_H_5_−CH_2_O_2_]^+^ intermediate (*m*/*z* 639) loses exclusively the C_6_H_5_−CH_2_O_2_^.^ fragment (Figure 3 d). Hence, the tolyl (C_6_D_4_−CH_3_) group is not bound through the oxygen. Analogically, the [**1 a**+C_6_H_4_−CH_3_, C_6_D_5_−CH_2_O_2_]^+^ ions (*m*/*z* 640) lose C_6_D_5_−CH_2_O_2_^.^. We also detect a loss of C_6_H_5_−CH_2_O_2_^.^, but its intensity is consistent with fragmentation of ^13^C isotopologue of [**1 a**+C_6_D_4_−CH_3_, C_6_H_5_−CH_2_O_2_]^+^, which is isobaric with the parent ions. We did not detect any oxygen‐transfer reactivity with this type of complexes.

### Photodissociation spectra of the detected intermediates

Next, we have determined structures of the detected intermediates by helium tagging infrared photodissociation (IRPD) spectroscopy.^13^, ^14^, ^15^ The correct structures were assigned based on comparison of the experimental data with theoretical IR spectra of possible isomers. We present the results for [**1 a**+2 H,2 O]^+^ here; the results for other complexes are shown in the Supporting Information (Figure S39), as their structures are analogous.

**Figure 4:**
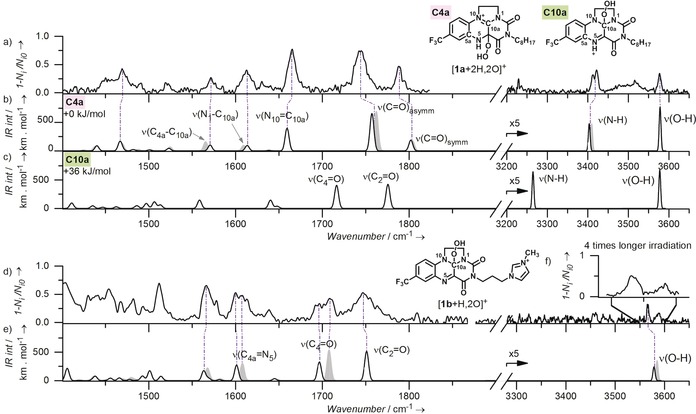
Comparison between IRPD and calculated vibrational spectra. a) IRPD, b) and c) theoretical spectra of hydroperoxyhydroflavinium [**1 a**+2 H,2 O]^+^ (regioisomer C4a and regioisomer C10a). d) IRPD and e) theoretical spectra of charge‐tagged hydroperoxyflavinium [**1 b**+H,2 O]^+^ (regioisomer C10a). The grey spectra in b) and e) correspond to alternative conformers of the same regioisomers lying 0.2 kJ mol^−1^ and 3 kJ mol^−1^, respectively, higher in energy. Further details and related IRPD spectra are in the Supporting Information (figures S38, S39, S40, and S42).

The IRPD spectrum of [**1 a**+2 H,2 O]^+^ shows an O−H stretching band at 3577 cm^−1^ and two overlapping N−H stretching bands at 3422 cm^−1^ and 3412 cm^−1^ (Figure 4 a,b). Neglecting the double appearance of the N−H stretching band, the experimental spectrum matches best with the theoretical spectrum of an isomer with the hydroperoxy group at the C4a carbon atom (structure C4a in Figure 4 b). The spectra of other possible isomers do not fit with the experiment (Supporting Information, Figure S38). We have further confirmed this assignment by comparison of the fingerprint region. The range between 1500 cm^−1^ and 1850 cm^−1^ shows characteristic pattern of C=O, C=C, and C=N vibrations that indicates saturation and unsaturation along the polycyclic backbone. This pattern is perfectly consistent with the hydroperoxy substituent in the C4a position and protonation of the N5 nitrogen atom.

We have further analysed the “double appearance” of the N−H stretching band. The [**1 a**+2 H,2 O]^+^ ion has two “acidic” hydrogen atoms. Observing one O−H and two N−H stretches thus suggests that the studied ions are either a mixture of two closely related isomers with the same O−H stretching frequency, or that the N−H band is split. In order to enlighten this problem, we have performed a two‐colour IR–IR experiment.^16^ In the experiment, we first depleted all complexes absorbing IR photons at 3412 cm^−1^ and recorded the IR spectrum of the remaining complexes (Supporting Information, Figure S42). This spectrum clearly shows one N−H stretch at 3422 cm^−1^ and the O−H stretch. In analogy, depleting the isomers absorbing at 3422 cm^−1^ led to a spectrum with one N−H band at 3412 cm^−1^ and with the O−H stretch. These experiments clearly show that the [**1 a**+2 H,2 O]^+^ ions are a mixture of two closely related isomers/conformers with the same frequency of the O−H band, but with slightly shifted N−H bands.

We have explored possible conformations of the [**1 a**+2 H,2 O]^+^ isomer shown in Figure 4 b. Indeed, we found two conformers with relative energies within 0.2 kJ mol^−1^ separated by a barrier of approximately 16 kJ mol^−1^ (Supporting Information, Figures S33). These two conformers have slightly shifted N−H vibrations, but the O−H vibration resides at the same position exactly as observed in the experiment (see the grey shadowing in Figure 4 b and Figure S42 in the Supporting Information). Further, we note that the wide band between the NH and OH bands in the spectrum might correspond to O−H stretches of conformers forming intramolecular hydrogen bonds (see details in the Supporting Information, Figures S39 e, S40, S41).

Intermediates with the benzyl group, [**1 a**+Bn,H,2 O]^+^, have almost an identical IRPD spectrum to that of [**1 a**+2 H,2 O]^+^ except the absence of the OH stretching vibration (Supporting Information, Figure S39 d). This implies that H is replaced by Bn in otherwise analogous structure of the ions. Hence, in accordance to the fragmentation spectra in Figure 3, the added hydrogen atom is dominantly attached to the N5 position and the C4a atom carries the benzylperoxy group.

We conclude that the peroxy‐intermediates in this photoreaction differ from those observed previously in dark reactions of **1** ^+^;^9^, ^17^ namely instead of the peroxy radical attack at the C10a position, we observed exclusively the attack at the C4a position. In order to confirm this active site switch, we measured IRPD spectrum of the product of the dark reaction between charge‐tagged flavinium **1 b** ^2+^ and HO_2_^−^, [**1 b**+H,2 O]^+^. The positions of double‐bond stretching bands in the IRPD spectrum of [**1 b**+H,2 O]^+^ are consistent with the hydroperoxylation in the position C10a (Figure 4 d,e). The range of X−H vibration does not contain any N−H stretches as expected. The O−H stretch is represented by two bands (see the insert in Figure 4 c) which again implies the presence of more conformers, this time caused by conformations of the imidazolium tail (Figures S34–S36). This conformational flexibility is also consistent with rather broad bands detected in the lower wavenumber range. In summary, this experiment shows that the charge‐tagging has reported otherwise neutral complexes formed in solution and that our analysis is perfectly consistent with previous assessments based on NMR spectroscopy.^9^, ^17^

**Figure 5:**
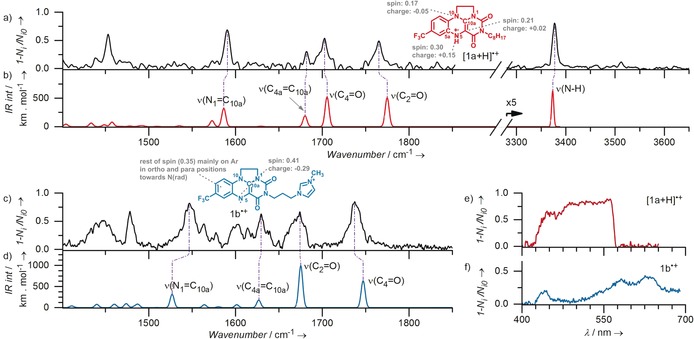
a) IRPD and b) theoretical spectra of [**1 a**+H]^+^. c) IRPD and d) theoretical spectra of charge‐tagged flavin radical **1 b^.^** ^+^. VisPD spectra of [**1 a**+H]^+^ e) and of **1 b** ^.+^ f). For more details see the Supporting Information (Figure S43). Mulliken spin and charge populations refer to the heavy atoms together with the attached hydrogen atoms (B3LYP‐D3/cc‐pVTZ//B3LYP‐D3/6‐311+**, SMD, solvent=Acetonitrile).

Other interesting aspects of the studied reaction are colour changes and their cause. Irradiation of yellow acetonitrile solution of **1 a** ^+^ (in the absence of oxygen) turns the colour to red. The previous EPR experiments suggested that the solution contains a mixture of stable radicals **1 a** ^.^ and [**1 a**+H]^+^.^9^ With our approach, we can measure visible spectra of individual isolated ions and thereby link these mass‐selected ions to colours. The desired cation radicals can be generated upon elimination of HOO^.^ from their hydroperoxy precursors [**1 b**+H,2 O]^+^ and [**1 a**+2 H,2 O]^+^ in the gas phase. Hence, we have generated the desired radicals by in‐source fragmentation of the precursors (Supporting Information, Figure S21) and measured their helium tagging photodissociation spectra. The spectra in the infrared range confirm the predicted molecular structures (Figure 5). Absorption of [**1 a**+H]^+^ in the visible range suggests the red colour for this radical (Figure 5 e). For the charge‐tagged “neutral” radical **1 b** ^.+^, the visPD spectrum corresponds to a blueish colour (Figure 5 f). In order to check possible effects of the imidazolium charge‐tag on the visPD spectrum (such as a π–π interaction between flavin rings and imidazolium) we have also synthesised analogue **1 c** ^2+^ with a trimethylammonium group instead of methylimidazolium. The visPD spectrum of **1 c** ^.+^ is identical to that of **1 b** ^.+^ (Supporting Information, Figure S43), thus excluding significant effects of imidazolinium on the measured visPD spectrum of hydroperoxyflavin intermediate. We note that the visPD spectra of **1 b** ^.+^ and its analogue **1 c** ^.+^ are similar to the spectrum of the neutral blue flavin semiquinone.^18^ Finally, we measured also the visPD spectrum of [**1 a**+Bn]^+^ (Supporting Information, Figure S43). This spectrum is almost identical to the spectrum of [**1 a**+H]^+^. This spectra overlap would make UV/Vis detection of the [**1 a**+Bn]^+^ very hard in solution as it could be misinterpreted as [**1 a**+H]^+^. Probably for similar reasons, previous EPR study failed to detect the [**1 a**+Bn]^+^ intermediates.^9^

## Discussion

**Figure 6:**
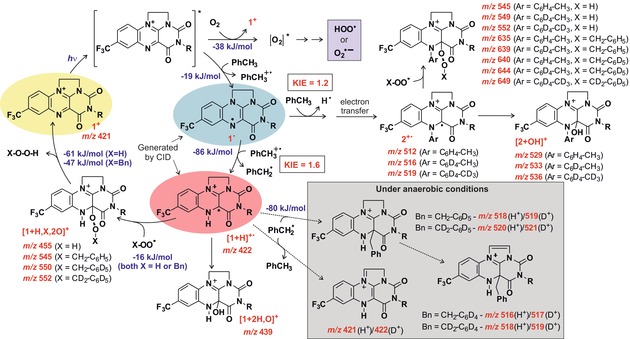
Intermediates in flavinium photocatalyzed oxidation of toluene. The blue numbers refer to B3LYP‐D_3_/6‐311G+** calculated reaction Gibbs energies in acetonitrile obtained for the **1 a^+^** catalyst (see Computational Details). The coloured ovals indicate the colours of the ions as determined from visPD experiments. The red m/z values refer to the observed intermediates.

Using electrospray ionization mass spectrometry, we have trapped and characterised a number of short‐lived intermediates and by‐products formed during the title photocatalytic oxidation. Knowing the structure and reactivity of individual species isolated from the reaction mixture, we can discuss the photocatalytic cycle and side pathways (Figure 6). We will support the discussion with DFT calculated thermochemistry of the reaction steps.

Most importantly, we characterised elusive peroxy‐intermediates [**1 a**+2 H,2 O]^+^ and [**1 a**+Bn,H,2 O]^+^. The hydroperoxy or benzylperoxy group is located at the C4a position of **1** which markedly differentiates intermediates in this photocatalytic reaction from those previously observed in dark oxidation reactions.^9^, ^17^ The C4a reaction site binds the radicals with a small binding energy. According to the DFT calculations, the binding energy of HOO^.^ is 16 kJ mol^−1^ and that of BnOO^.^ is 17 kJ mol^−1^ (Figure 6). Therefore, these intermediates can easily detach the peroxy radicals. Alternatively, they can lose H_2_O_2_ or BnOOH and regenerate the catalyst.

The formation of these key intermediates is driven by protonation at the N5 position during the initial proton coupled electron transfer reaction cascade (Figure 6). Protonation at any other reaction site is thermodynamically disfavoured (Supporting Information, Figure S37). The initially formed radical **1** ^.^ and the radical cation [**1**+H]^+^ are formed in exothermic steps with energy release of 19 kJ mol^−1^ and 86 kJ mol^−1^, respectively, by electron transfer with toluene and then proton transfer with toluene radical cation. Either **1** ^.^ or [**1**+H]^+^ could be hardly directly detected from the reaction mixture, probably due to their high reactivity under the conditions of the experiment. Nevertheless, we were able to track by‐products to which these intermediates transformed in absence of oxygen. In absence of oxygen‐based radicals, [**1**+H]^+^ reacts with the benzyl radical. The reaction either regenerates toluene by hydrogen atom transfer or leads to a coupling product (see the gray panel in Figure 6).^6^, ^19^

Once the N5 position is filled, the [**1**+H]^+^ intermediate reacts with the hydroperoxy radical. This mode of reactivity was shown previously for electrochemically generated alkylated analogue of [**1**+H]^+^.^20^, ^21^, ^22^ The hydroperoxy radicals in the reaction mixture most probably from excited molecular oxygen (singlet oxygen) or from superoxide (O_2_^−^), both resulting from reactivity of the excited flavinium with oxygen.^23^, ^24^ We note that analogous reactivity was suggested for oxygen activation in a flavin‐dependent monooxygenase based on DFT calculations.^3^

The most important side‐pathway of the photocatalytic cycle is formation of arylated flavin radical cation **2** ^+^. Several possible reaction sequences can lead to **2** ^+^. For example, initially formed radical **1** ^.^ can make aromatic electrophilic substitution at the aromatic ring of toluene. The product molecule, aryl‐substituted flavin molecule **2**, could transform to the observed **2** ^+^ by electron transfer reaction. The **2** ^+^ intermediate reacts further in analogy to [**1**+H] ^+^. Hence, it forms either the benzylperoxy intermediate [**2**+Bn,2 O]^+^ or the hydroxylated by‐product [**2**+H,O]^+^.

The observed reactivity and site‐selectivity agrees well with the “N5‐blocking” effect described by Bruice et al.^20^, ^21^, ^22^ Their observations showed that alkylation at the N5 position activates the C4a position for reactions with peroxides. This effect is known for more than 40 years. However, so far, it was impossible to prove experimentally that simple protonation activates this reaction pathway too.^25^ Herein, we bring a clear spectroscopic evidence that protonation suffices to activate the C4a reaction site. This reactivity mode is important for flavins in biological systems. There the N5‐blocking approach^26^ and formation of hydroperoxy‐intermediates are important as well.^1^, ^27^

## Conclusion

This study shows the first detection of short‐lived peroxy‐intermediates in a photocatalytic oxidation reaction. We have employed electrospray ionization mass spectrometry and detected the intermediates in photooxidation of toluene using flavinium catalyst. The short‐lived (sub‐second time scale) intermediates are formed after initial proton‐coupled electron transfer between photoexcited flavinium and toluene. The protonation blocks the N5 position of the reduced flavinium and thus activates the C4a position for radical reactions. This radical cation couples with hydroperoxy radical to form the detected peroxy intermediate.

Next to the key intermediates, we have detected also arylated flavinium ions. Alternatively to the initial electron transfer–proton transfer sequence, the flavinium can react along the pathway electron transfer–aromatic electrophilic substitution with toluene. This pathway leads to N5 arylation of the flavinium catalyst. Hence, N5‐blocked flavinium salts might be resistant towards this deactivation pathway and thus represent catalysts with a higher turnover number. Additionally, N5‐blocking effect could open an activation pathway for aromatic rings without formation of the stable adducts.

The structure of all the detected intermediates was confirmed by isotope labelling studies and by measuring IR and visible spectra of the mass‐selected intermediates. Thereby, we have confirmed the photocatalytic reactive site switch to the C4a position (contrary to the usual dark C10a reactivity). We have clearly shown that this site switch was induced by protonation of the N5 position. This finding is in agreement with the known concept of the N5‐blocking effect that was shown for N5‐alkylated flavin derivatives.

## Experimental Section

**Mass spectrometry and Gas‐phase reactivity** experiments were performed with a TSQ 7000 (Finnigan, Inc.) quadrupole‐octopole‐quadrupole mass spectrometer equipped with and electrospray (ESI) ion source.^28^ If not mentioned otherwise, 20–150 μm solutions containing **1 a** ^+^ or **1 b** ^2+^ were introduced to the ions source under mild ionization conditions. All experiments including details of capillary irradiation are described in detail in the Supporting Information. Photocatalyst **1 a** ^+^(Cl^−^) was prepared according to published procedure;^9^ synthesis of **1 b** ^2+^(Br^−^)_2_ is described in the Supporting Information.

Gas‐phase reactivity experiments of the ions mass‐selected by the first quadrupole were done in the octopole collision cell heated to 70 °C, filled with a reactant gas with pressures below 1 mTorr at nominally zero collision energy (Supporting Information, Figure S22). Reaction products were mass‐analysed by the second quadrupole.

**IRPD spectroscopy** experiments were done with the ISORI instrument,^29^ featuring the same ion source as TSQ 7000. Ions were ionized under conditions similar to other MS experiments. Desired ions were then mass selected and transferred to the ion trap, where they were cooled to 3 K. At this temperature, ions formed complexes with He molecules. These complexes were irradiated by 8 pulses from optical parametric oscillator/amplifier (Laservision) pumped by Nd:YAG laser operating at 10 Hz frequency. After irradiation, the ions were expelled from the trap, mass‐analysed by a second quadrupole and the number of the He complexes, *N*, was by a Daly‐type detector operating in the counting mode. This experiment has been repeated without irradiation to obtain the reference number of the He complexes population prior to dissociation, *N*_0_. The IRPD spectra were then constructed as the wavenumber‐dependent ratio (1−*N*/*N*_0_). The wavelength was determined by wavelength meter WS‐600 (HighFinesse GmbH). In two‐colour experiments, second laser wavelength has been determined by comparison with the spectrum obtained using the first laser. For visPD experiments^30^ SuperK laser system (NKT photonics) was used.

**DFT calculations** were carried out using B3LYP functional^31^ with D3 dispersion correction^32^ and 6‐311+** basis set as implemented in Gaussian G09 revision D.01. For calculations of IR spectra in Figures 4 b, 5 b, 5 d and S38, we used the pc‐3 basis set^33^ for the carbon atom of the CF_3_ group. The IR spectra were calculated in the harmonic approximation and scaled by 0.985 in the range below 2000 cm^−1^ and by 0.952 in the range above 2000 cm^−1^. In all calculations except the structure and IR spectrum in Figure 5 b, the side chain of **1 a** ^+^ has been reduced from octyl to methyl. For further details please refer to the Supporting Information.

## Conflict of interest

The authors declare no conflict of interest.

## Supporting information

As a service to our authors and readers, this journal provides supporting information supplied by the authors. Such materials are peer reviewed and may be re‐organized for online delivery, but are not copy‐edited or typeset. Technical support issues arising from supporting information (other than missing files) should be addressed to the authors.

Supplementary
