# Supplementary material for: Flavinium Catalysed Photooxidation: Detection and Characterization of Elusive Peroxyflavinium Intermediates
Source: Angew Chem Int Ed Engl. 2019 Aug 23;58(43):15412–20. doi: 10.1002/anie.201906293 (PMC6852162; doi:10.1002/anie.201906293)
Supplement: Supplementary file 1 — Supplementary [file ANIE-58-15412-s001.pdf]

Angewandte Chemie
Eine Zeitschrift der Gesellschaft Deutscher Chemiker

Supporting Information

Flavinium Catalysed Photooxidation: Detection and Characterization of Elusive Peroxyflavinium Intermediates

*Jan Zelenka, Radek Cibulka, and Jana Roithová**

anie_201906293_sm_miscellaneous_information.pdf

Table of content

1 Synthesis of flavinium compounds 3

1.1 Preparation of 3-(3-brompropyl)-10-(2-hydroxyethyl)-7-(trifluoromethyl)isoalloxazine (S2) 3

1.2 Preparation of 3-(3-(1-methylimidazolium-3-yl)propyl)-10-(2-hydroxyethyl)-7-(trifluoromethyl)isoalloxazine bromide (S3a) 4

1.3 Preparation of 1,10-ethyliden-3-(3-(1-methylimidazolium-3-yl)propyl)- 7-(trifluoromethyl)isoalloxazinium dibromide (1b) 4

1.4 Preparation of 3-(3-(trimethylammonio)propyl)-10-(2-hydroxyethyl)-7-(trifluoromethyl)isoalloxazine bromide (S3b) 5

1.5 Preparation of 3-(3-(trimethylammonio)propyl)-10-(2-hydroxyethyl)-7-(trifluoromethyl)isoalloxazine dibromide (1c) 5

2 ESI-MS and ESI-MS^2 experiments 6

2.1 Experimental details 6

2.1.1 Instrumentation 6

2.1.2 Irradiation layout 6

2.1.3 Ionization methods 7

2.1.4 Preparation of solution for the ESI ionization and ionization conditions 8

2.2 MS and MS2 data and conditions 8

2.2.1 MS spectra acquired without irradiation 8

2.2.2 Experimental details of MS spectra acquired with sample irradiation under oxygen-free conditions. 10

2.2.3 MS spectra acquired with syringe irradiation method 12

2.2.4 MS spectra acquired with capillary irradiation method 14

2.2.5 MS spectra acquired with capillary tip irradiation method 15

2.2.6 Fragmentation of the selected ions 19

2.2.7 Fragmentation in the source 25

2.2.8 Reactivity of the selected ions 26

3 Determination of the KIE from the MS experiments 30

4 IRPD and VIS-PD experiments, DFT calculations, UV-VIS 32

4.1 Experimental Details 32

4.2 Computational Details 33

4.2.1 Computational dihedral angle study of ($[1c+H_2O_2]^+$) and ($[1c+H_2O]^+$) 33

4.2.2 Computational study of different conformers of ($[1b+OOH]^+$) 33

4.3 Proton affinity study of the 1• 36

4.4 Comparison between the DFT calculated spectra and the experimental one 37

4.4.1 Comparison between observed $[1a+2H,2O]^+$ spectrum and calculated spectra of different structural isomers 37

4.5 Other IRPD experiments 38

4.5.1 IRPD spectra of selected ions 38

4.5.2 Attenuation experiments 40
4.5.3 2D IRPD experiments with [1a+2H,2O]$^+$ 41
4.6 Comparison between VIS-PD spectra 41
5 XYZ coordinates of the calculated structures 42
6 NMR spectra of the synthetized compounds 47
7 HRMS-ESI+ spectra of the synthetized compounds 74
8 Overview of other work applying helium tagging spectroscopy 74

1 Synthesis of flavinium compounds

Flavinium salt **1a**, precursor **S1** and analogue of **1a** with methyl in N3 position instead of octyl (**1d**) were synthetized by the previously published procedures.[1] Synthesis of the charge-tag derivatives **1b** and **1c** is outlined in figure **S1**.

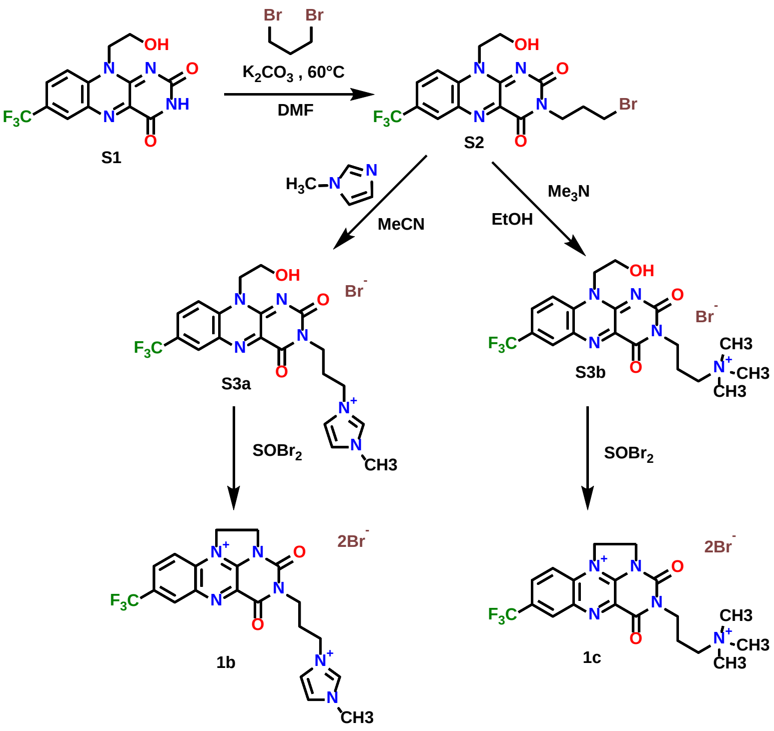

Figure S1. Synthesis of the charge-tagged flavinium derivates **1b** and **1c**.

1.1 Preparation of 3-(3-brompropyl)-10-(2-hydroxyethyl)-7-(trifluoromethyl)isoalloxazine (S2)

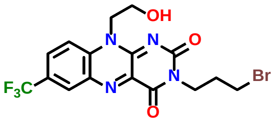

To a mixture of 10-(2-hydroxyethyl)-7-trifluoromethylisoalloxazine (**S1**, 400 mg, 1.22 mmol), 1,3-dibromopropane (1ml, 1.99g, 9.85 mmol) in DMF (50ml) powdered K_2CO_3 (0.21g, 1.5 mmol) was added. The reaction mixture was then heated to 65°C for 300 min in apparatus equipped by drying tube packed with $CaCl_2$ in a flask covered by an aluminium foil. Reaction was quenched by evaporation of a solvent (14 torr, 70°C in bath). To the residual slurry, solution of 1 ml of 35% HCl diluted by 20 ml of water was added. This suspension was then extracted three times by 20 ml of $CHCl_3$. Mixed organic phases were then dried by $MgSO_4$, filtered and evaporated. The crude product was purified by a column chromatography ($CHCl_3$: MeOH 15:1). Oily residue from the chromatography was precipitated by Et_2O. Precipitate **S2** weighted 300 mg (55% of theoretical yield).

^{1}H NMR (500 MHz, Chloroform-*d*) δ 8.56 (s, 1H, *6Ar-**H***), 8.06 (dd, *J* = 9.1, 2.0 Hz, 1H, *8Ar-**H***), 8.03 (d, *J* = 9.1 Hz, 1H, *9Ar-**H***), 4.92 (t, *J* = 5.4 Hz, 2H, *NC**H₂**CH₂OH*), 4.21 (t, *J* = 5.4 Hz, 2H, ***CH₂**OH*), 4.16 (t, *J* = 7.0 Hz, 2H, *NC**H₂**CH₂CH₂Br*), 3.44 (t, *J* = 6.9 Hz, 2H, *NCH₂CH₂C**H₂**Br*), 2.25 (p, *J* = 6.9 Hz, 2H, *NCH₂C**H₂**CH₂Br*).

^{13}C NMR (126 MHz, Chloroform-*d*) δ 158.93 (***C**=O*), 155.23 (***C**=O*), 149.75 (*10a-**C***), 138.22 (*4a-**C***), 135.43 (*9a-**C***), 134.96 (*5a-**C***), 131.33 (q, *J* = 3.1 Hz, *8-**CH***), 130.37 (q, *J* = 4.0 Hz, *6-**CH***), 128.78 (q, *J* = 34.4 Hz, *7**C**-CF₃*), 122.91 (q, *J* = 272.5 Hz, ***C**F₃*), 117.46 (*9-**CH***), 59.65 (*NCH₂**C**H₂OH*), 47.41 (*N**C**H₂CH₂OH*), 41.16 (*N**C**H₂CH₂CH₂Br*), 30.89 (*NCH₂**C**H₂CH₂Br*), 30.12 (*NCH₂CH₂**C**H₂Br*).

^{15}N NMR (51 MHz, Chloroform-*d*) δ 343.81 (***5N***), 168.13 (***3N***), 153.62 (***10N***).

^{19}F NMR (471 MHz, Chloroform-*d*) δ -62.66 (s, 3F, $\mathit{CF_3}$).

HRMS (ESI+, *m/z*): calcd for $C_{16}H_{15}BrF_3N_4O_3$ $[M+H]^+$: 447.02796; found: 447.03016. calcd for $C_{16}H_{14}BrF_3N_4NaO_3$ $[M+Na]^+$: 469.00991; found: 469.01187.

1.2 Preparation of 3-(3-(1-methylimidazolium-3-yl)propyl)-10-(2-hydroxyethyl)-7-(trifluoromethyl)isoalloxazine bromide (S3a)

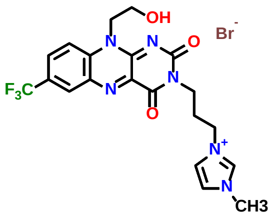

A suspension of **S2** (150 mg, 0.34mmol) in N-methylimidazole (40 µl, 0.50 mmol) and MeCN (1.5 ml) was heated overnight to 60-70 °C (bath temperature) in a flask equipped by drying tube packed with $CaCl_2$. Solid residue was suspended in 3 ml of MeCN, heated up to reflux and then left to slowly cool. Solid was then filtered off and washed with MeCN. Solid residue **S3a** weighted 146 mg (82% of theoretical yield) and was used without further purification.

^{1}H NMR (500 MHz, DMSO-d_6) δ 9.16 (s, 1H, *2-ImAr**H***), 8.51 (d, *J* = 1.6 Hz, 1H, *6Ar-**H***), 8.33 (d, *J* = 9.1 Hz, 1H, *9Ar-**H***), 8.23 (dd, *J* = 9.1, 2.2 Hz, 1H, *8Ar-**H***), 7.82 (t, *J* = 1.8 Hz, 1H, *4Im-C**H***), 7.74 (t, *J* = 1.8 Hz, 1H, *5Im-C**H***), 5.01 (t, *J* = 5.8 Hz, 1H, *O**H***), 4.75 (t, *J* = 5.8 Hz, 2H, $\mathit{N\mathbf{CH_2}CH_2OH}$), 4.27 (t, *J* = 7.2 Hz, 2H, $\mathit{NCH_2CH_2\mathbf{CH_2}Im}$), 3.95 (t, *J* = 6.6 Hz, 2H, $\mathit{N\mathbf{CH_2}CH_2CH_2Im}$), 3.89 – 3.80 (m, 5H, $\mathit{\mathbf{CH_3}}$, $\mathit{\mathbf{CH_2}OH}$), 2.18 (p, *J* = 7.0 Hz, 2H, $\mathit{NCH_2\mathbf{CH_2}CH_2Im}$).

^{13}C NMR (126 MHz, DMSO- d_6) δ 159.65 (*C=O*), 155.27 (*C=O*), 150.26 (*10a-**C***), 139.94 (*4a-**C***), 137.18 (*2Im-**C**H*), 136.34 (*9a-**C***), 134.52 (*5a-**C***), 130.42 (*8-**C***), 129.17 + 129.01 (*6-**C***), 126.44 (q, *J* = 33.2 Hz, *7**C**-CF$_3$*), 124.16 + 124.00 (*5Im-**C**H*), 123.96 (q, *J* = 272.1 Hz, $\mathit{\mathbf{C}F_3}$), 122.81 + 122.64 (*4Im-**C**H*), 119.65 (*9-**C***), 57.94 ($\mathit{\mathbf{C}H_2OH}$), 47.37 ($\mathit{N\mathbf{C}H_2CH_2OH}$), 47.22 ($\mathit{NCH_2CH_2\mathbf{C}H_2Im}$), 38.44 ($\mathit{N\mathbf{C}H_2CH_2CH_2Im}$), 36.26 ($\mathit{\mathbf{C}H_3}$), 28.54 ($\mathit{NCH_2\mathbf{C}H_2CH_2Br}$).

^{13}C NMR (126 MHz, DMSO- d_6, **65 °C**) δ 159.55 (*C=O*), 155.17 (*C=O*), 150.35 (*10a-**C***), 139.88 (*4a-**C***), 136.41 (*9a-**C***), 134.68 (*5a-**C***), 130.38 (q, *J* = 3.3 Hz, *8-**C***), 129.08 (q, *J* = 4.0 Hz, *6-**C***), 126.59 (q, *J* = 33.5 Hz, *7**C**-CF$_3$*), 124.11 (*5Im-**C**H*), 123.96 (q, *J* = 272.8 Hz, $\mathit{\mathbf{C}F_3}$), 122.80 (*4Im-**C**H*), 119.57 (*9-**C***), 58.06 ($\mathit{\mathbf{C}H_2OH}$), 47.54 ($\mathit{N\mathbf{C}H_2CH_2OH}$), 47.33 ($\mathit{NCH_2CH_2\mathbf{C}H_2Im}$), 38.54 ($\mathit{N\mathbf{C}H_2CH_2CH_2Im}$), 36.31 ($\mathit{\mathbf{C}H_3}$), 28.57 ($\mathit{NCH_2\mathbf{C}H_2CH_2Im}$).

^{15}N NMR (51 MHz, DMSO-d_6) δ 336.67 (***5N***), 181.95 (*3Im-**N***), 171.35 (*1Im-**N***), 166.28 (***3N***), 154.42 (***10N***).

^{19}F NMR (471 MHz, DMSO-d_6) δ -60.72 (s, 3F, $\mathit{CF_3}$).

HRMS (ESI+, *m/z*): calcd for $C_{20}H_{20}F_3N_6O_3$ $[M-Br]^+$: 449.15490; found: 449.15471.

1.3 Preparation of 1,10-ethyliden-3-(3-(1-methylimidazolium-3-yl)propyl)-7-(trifluoromethyl)isoalloxazinium dibromide (1b)

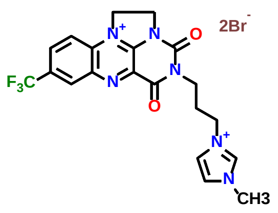

To **S3a** (19.7 mg; 37 µmol) in a small Ar flushed Schlenk tube covered by aluminium foil $SOBr_2$ (200 µl; 2.58 mmol) was added. The mixture was then stirred for 150 minutes. Crude sluggish product was obtained by an evaporation of $SOBr_2$ under the reduced pressure (<1 mbar). It has been used / characterized without further purification.

NOTE: Attempts to precipitate the compound from Acetonitrile / Et_2O have led to a crystalline mass which is 1:1 mixture of an unknown impurity (m/z: M-2Br + OH) and the desired compound. No procedure to isolate and precipitate the desired compound as pure crystalline mass has been found. During MS experiments the desired ion was isolated by mass selection.

^{1}H NMR (500 MHz, DMSO-d_6) δ 9.10 (s, 1H, *2-ImAr**H***), 8.96 (d, *J* = 1.8 Hz, 1H, *6Ar-**H***), 8.62 (dd, *J* = 8.9, 2.0 Hz, 1H, *8Ar-**H***), 8.42 (d, *J* = 8.8 Hz, 1H, *9Ar-**H***), 7.76 (t, *J* = 1.8 Hz, 1H, 4*Im-C**H***), 7.64 (t, *J* = 1.8 Hz, 1H, *5Im-C**H***), 5.34 (dd, *J* = 10.3, 8.5 Hz, 2H, 10$\mathit{N\mathbf{CH_2}CH_2N}$ *– assigned by small interaction with 9a-C*), 4.69 (t, *J* = 9.4 Hz, 2H, 10$\mathit{NCH_2\mathbf{CH_2}N}$), 4.30 (dd, *J* = 8.4, 6.8 Hz,

2H, $NCH_2CH_2CH_2Im$), 4.02 (t, *J* = 6.4 Hz, 2H, $NCH_2CH_2CH_2Im$), 3.78 (s, 3H, CH_3), 2.19 – 2.09 (p, *J* = 14.2 Hz, 2H, $NCH_2CH_2CH_2Im$).

^{13}C NMR (126 MHz, DMSO- d_6) δ 157.52 (*C=O*), 147.05 (*C=O*), 144.61 (*10a-**C***), 138.96 (*5a-**C***), 136.24 (*4a-**C***), 133.47 (d, *J* = 3.0 Hz, *8-**C***), 131.23 (*9a-**C***), 130.89 (q, *J* = 33.7 Hz, *7**C**-CF_3*), 130.21 (d, *J* = 4.0 Hz, *6-**C***), 124.45 (q, *J* = 274.0 Hz, CF_3), 123.98 (*5Im-**C**H*), 122.61 (*4Im-**C**H*), 120.41 (*9-**C***), 51.11 (10NCH_2CH_2N), 46.71 ($NCH_2CH_2CH_2Im$), 46.51 (10NCH_2CH_2N), 39.41 ($NCH_2CH_2CH_2Im$), 36.22 (CH_3), 28.43 ($NCH_2CH_2CH_2Im$).

^{15}N NMR (51 MHz, DMSO-d_6) δ 350.01 (*5**N***), 181.85 (*3Im-**N***), 171.93 (*10**N***), 171.34 (*1Im-**N***), 163.16 (*3**N***), 134.89 (*1**N***).

^{19}F NMR (471 MHz, DMSO-d_6) δ -61.09 (s, 3F, CF_3).

HRMS (ESI+, *m/z*): calcd for $C_{20}H_{19}F_3N_6O_2Br$ [M-Br]$^+$: 511.07050; found: 511.06834. Calcd for $C_{21}H_{22}F_3N_6O_3$ [M-2Br+CH_3O]$^+$: 463.17055; found: 463.16788. Calcd for $C_{20}H_{20}F_3N_6O_3$ [M-2Br+OH]$^+$: 449.15490; found: 449.15207. Calcd for $C_{20}H_{18}F_3N_6O_2$ [M-Br-HBr]$^+$: 431.14433; found: 431.14215.

1.4 Preparation of 3-(3-(trimethylammonio)propyl)-10-(2-hydroxyethyl)-7-(trifluoromethyl)isoalloxazine bromide (S3b)

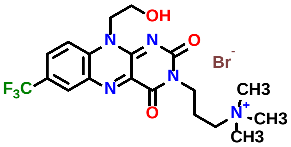

To a suspension of **S2** (105 mg; 235 µmol) in acetonitrile (0.6 ml) in brown capped vial solution of trimethylamine in ethanol (4.2M, 150 µl; 630 µmol) was added. After addition the starting compound dissolved readily. After approximately 60 minutes the product started to precipitate. Mixture has been left to stir overnight. In the morning the mixture was diluted by excess of Et2O and centrifuged. The solid product was suspended in Et2O and suspension was transferred into flask, evaporated on RVO and dried. Isolated yield was 72 mg (61 %).

^{1}H NMR (500 MHz, DMSO-d_6) δ 8.55 – 8.51 (m, 1H, *6Ar-**H***), 8.34 (d, *J* = 9.1 Hz, 1H, *9Ar-**H***), 8.25 (dd, *J* = 9.2, 2.2 Hz, 1H, *8Ar-**H***), 5.01 (t, *J* = 5.8 Hz, 1H, *OH*), 4.77 (t, *J* = 5.8 Hz, 2H, NCH_2CH_2OH), 4.01 (t, *J* = 6.4 Hz, 2H, $NCH_2CH_2CH_2N(CH_3)_3$), 3.87 (q, *J* = 5.8 Hz, 2H, NCH_2CH_2OH), 3.47 – 3.40 (m, 2H, $NCH_2CH_2CH_2N(CH_3)_3$), 3.06 (s, 9H, $N(CH_3)_3$), 2.14 – 2.04 (m, 2H, $NCH_2CH_2CH_2N(CH_3)_3$).

^{13}C NMR (126 MHz, DMSO-d_6) δ 159.65 (C=O), 155.21 (C=O), 150.27 (*10a-**C***), 139.87 (*4a-**C***), 136.34 (*9a-**C***), 134.53 (*5a-**C***), 130.49 (d, *J* = 3.0 Hz, *8-**C***), 129.14 (d, *J* = 4.3 Hz, *6-**C***), 126.48 (q, *J* = 33.4 Hz, *7**C**-CF_3*), 123.96 (q, *J* = 272.0 Hz, CF_3), 119.67 (d, *J* = 4.3 Hz, *9-**C***), 63.63 ($NCH_2CH_2CH_2N(CH_3)_3$), 57.92 (NCH_2CH_2OH), 52.73 ($N(CH_3)$)), 52.70 ($N(CH_3)$)), 52.67 ($N(CH_3)$)), 47.40 (NCH_2CH_2OH), 38.59 ($NCH_2CH_2CH_2N(CH_3)_3$), 21.87 ($NCH_2CH_2CH_2N(CH_3)_3$).

^{15}N NMR (51 MHz, DMSO-d_6) δ 336.87 (*5**N***), 165.91 (*3**N***), 154.48 (*10**N***), 48.91 ($N(CH_3)_3$).

^{19}F NMR (471 MHz, DMSO-d_6) δ -60.71 (s, 3F, CF_3).

HRMS (ESI+, *m/z*): calcd for $C_{20}H_{20}F_3N_6O_3$ [M-Br]$^+$: 426.17530; found: 426.17484.

1.5 Preparation of 3-(3-(trimethylammonio)propyl)-10-(2-hydroxyethyl)-7-(trifluoromethyl)isoalloxazine dibromide (1c)

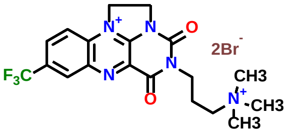

To **S3b** (14.7 mg; 29 µmol) in a small Ar flushed Schlenk tube covered by aluminium foil $SOBr_2$ (200 µl; 2.58 mmol) was added. The mixture was then stirred for 220 minutes. Crude sluggish product was obtained by an evaporation of $SOBr_2$ under the reduced pressure (<1 mbar). It has been used / characterized without further purification.

NOTE: Attempts to precipitate the compound from Acetonitrile / Et_2O have led to a crystalline mass which is 1:1 mixture of an unknown impurity (m/z: M-2Br + OH) and the desired compound. No procedure to isolate and precipitate the desired compound as pure crystalline mass has been found. During MS experiments the desired ion was isolated by mass selection. From the 19F-NMR it can be established that the impurity and the target compound are in 1:3 ratio in the case of the NMR sample.

^{1}H NMR (500 MHz, DMSO-d_6) δ 8.94 (d, J = 1.9 Hz, 1H, *6Ar-**H***), 8.59 (dd, J = 9.0, 1.9 Hz, 1H, 8*Ar-**H***), 8.40 (d, J = 8.9 Hz, 1H, 9*Ar-**H***), 5.30 (dd, J = 10.4, 8.5 Hz, 2H, $NC\mathbf{H_2}CH_2N$), 4.69 (t, J = 9.4 Hz, 2H, $NCH_2C\mathbf{H_2}N$), 4.08 (t, J = 5.8 Hz, 2H, $NC\mathbf{H_2}CH_2CH_2N(CH_3)_3$), 3.50 – 3.42 (m, 2H, $NCH_2CH_2C\mathbf{H_2}N(CH_3)_3$), 2.97 (s, 12H, $N(C\mathbf{H_3})_3$ + $N(C\mathbf{H_3})_3$ from impurity), 2.08 (tt, J = 10.1, 5.2 Hz, 2H, $NCH_2\mathbf{C}H_2CH_2N(CH_3)_3$).

^{13}C NMR (126 MHz, DMSO-d_6) δ 157.58 (C=O), 147.04 (C=O), 144.55 (*10a-**C***), 139.68 (uninterpreted), 139.00 (5*a-**C***), 136.12 (uninterpreted), 133.52 (*8-**C***), 131.34 (*9a-**C***), 130.83 (d, J = 33.8 Hz, *7**C**-CF₃*), 130.23 (*6-**C***), 123.37 (d, J = 273.2 Hz, $\mathbf{C}F_3$), 120.28 (*9-**C***), 62.90 ($NCH_2CH_2\mathbf{C}H_2N(CH_3)_3$), 52.56 ($N(\mathbf{C}H_3)_3$), 51.19 ($NCH_2\mathbf{C}H_2N$), 46.56 ($N\mathbf{C}H_2CH_2N$), 39.47 ($N\mathbf{C}H_2CH_2CH_2N(CH_3)_3$), 21.42 ($NCH_2\mathbf{C}H_2CH_2N(CH_3)_3$).

^{15}N NMR (51 MHz, DMSO-d_6) δ 349.89 (*5**N***), 172.14 (*10**N***), 153.22 (*3**N***), 135.14 (*1**N***), 48.71 ($\mathbf{N}(CH_3)_3$).

^{19}F NMR (471 MHz, DMSO-d_6) δ -60.79 (impurity), -61.09 (compound, s, 3F, $C\mathbf{F_3}$).

2 ESI-MS and ESI-MS^2 experiments

2.1 Experimental details

2.1.1 Instrumentation

If not stated otherwise, reported MS and MS^2 spectra were measured on Finnigan TSQ-7000 machine with an ESI ion source. For sample irradiation, 1W laser diode (445nm, blue, e-bay) with Thorlabs ballast set at 1A current was used. In all cases fused silica capillary (internal diameter 100 µm, outer diameter 190 µm, polyimide coating PostNova, part no. Z-FSS-100190 was used).

2.1.2 Irradiation layout

In our experiments, we have irradiated the sample at three different positions: at a syringe/vial (1), at a capillary (2) and at the tip of a capillary (3). (1) For the syringe irradiation, we placed Hamilton syringe to a syringe pump and we focused a laser beam through the end of a glass part of the syringe as illustrated on Figure S2a. We placed alumina foil in opposite to the laser beam to reflect the laser beam back through the solution. (2) For the capillary irradiation (Figure S2b) we removed coating of the fused capillary by published procedure.[2] First we burnt the capillary coating quickly by a lighter. Then we removed the carbon deposit by a tissue wetted with acetonitrile. We removed the coating approximately 10-15 cm away from the capillary tip (uncoated capillary often broke and replaced by a new one where the window was at slightly different position). (3) When we irradiated the capillary tip (Figure S2c, S2d) we removed coating in same way as previously described. We were forced to bypass the microswitch of the ESI head so we could leave it opened in the course of spraying. Although this system resembles the previously published nano-ESI setup,[3] we applied much higher flow-rate (0.20-0.40 ml/h compared to nano-ESI flow) to improve our chance to detect reactive intermediates.

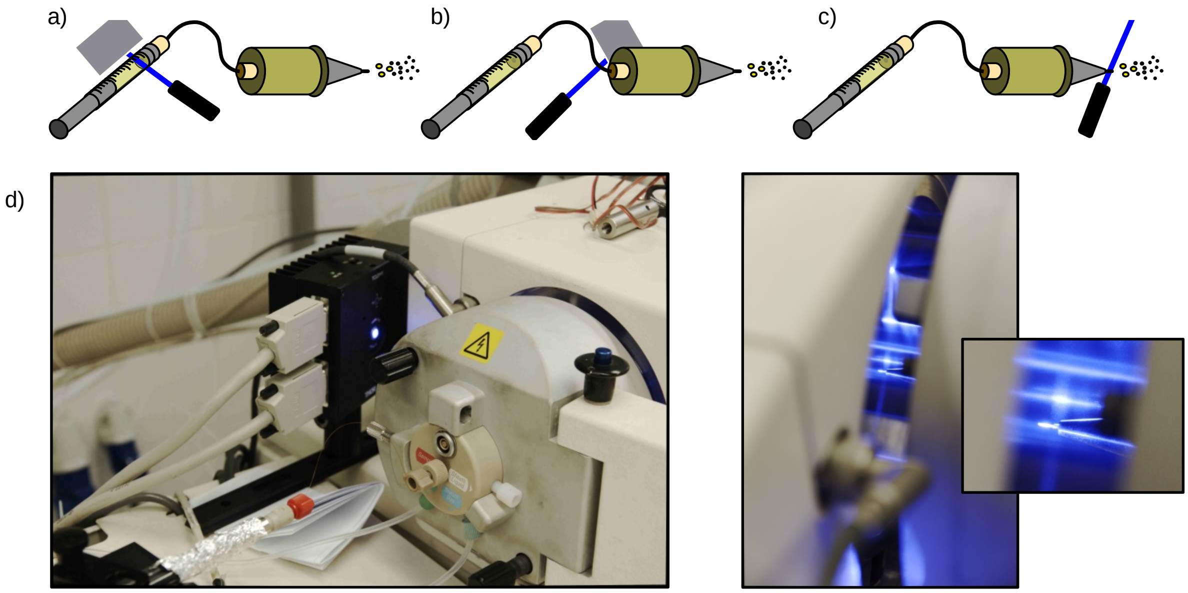

Figure S2. Irradiation at the syringe (a), the capillary (b) and the tip of a capillary (c), (d)

2.1.3 Ionization methods

2.1.3.1 Syringe ionization

When noted that syringe ionization has been applied, the current for ESI-MS has been delivered directly to the sample by custom-made syringe piston for Hamilton syringe.

Piston description: Stainless steel screw has been used as an electrode. The sealing and cap comes from original Hamilton piston. The custom piston has been made from 3D printed PET. We have quickly tested the piston and did not observe any ions coming from this syringe when sprayed under conditions of experiments mentioned below. This piston has been connected to ESI-MS source through 20 MΩ resistor. The .stl 3D model ready for printing (optimized for 0.20 mm layer-height) is downloadable on our group website, or accessible through the corresponding author.

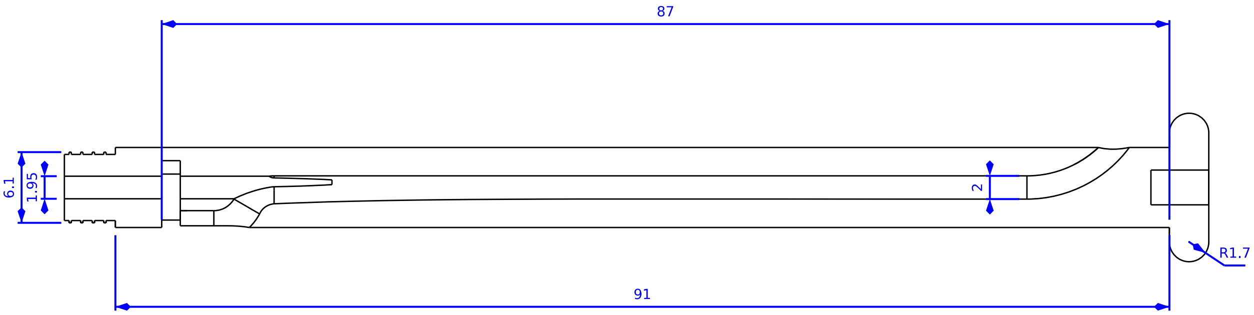

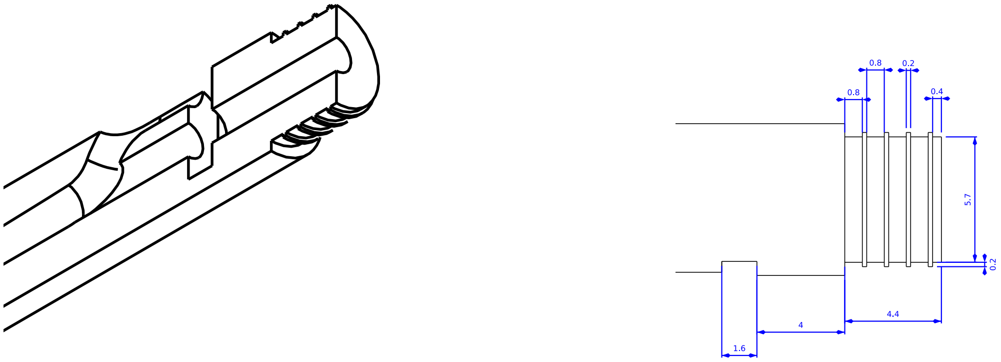

Figure S3. Schematics of piston used for syringe ionization (dimensions are in mm).

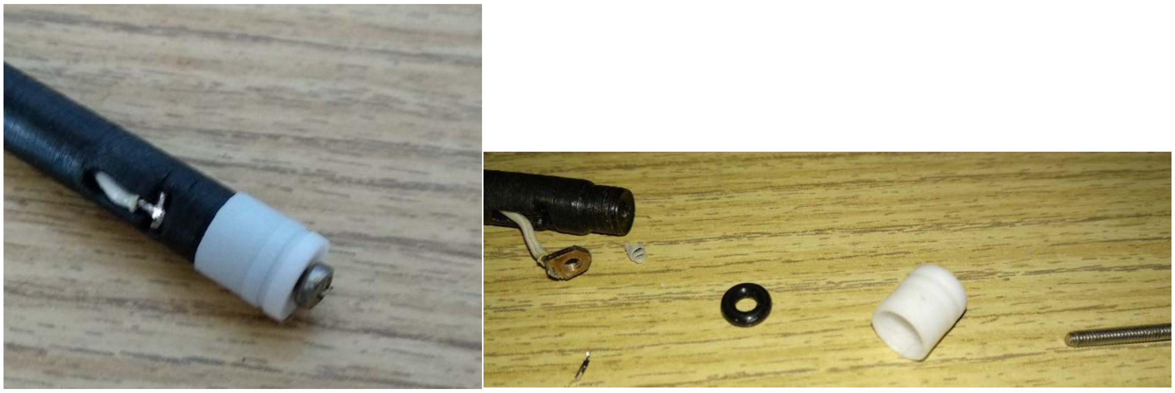

Figure S4. Pictures of completed and disassembled piston head.

2.1.3.2 “Overpressure unit”

When noted that “overpressure unit” ionization technique has been applied, [4] high voltage was applied by stainless steel wire to the diluted mixture in 4ml vial. The wire, fused silica capillary connected to MS and needle connected to tube for pressurizing the vial were induced through the septum in the vial cap. Pressure has been regulated manually based on the sample viscosity and sample flow to optimize the signal intensity.

2.1.4 Preparation of solution for the ESI ionization and ionization conditions

Syringe was covered by alumina foil if irradiation was applied to the capillary or capillary tip. In some capillary-tip irradiation experiments syringe was replaced by overpressure unit described above. Acetonitrile was used as an exclusive solvent in the whole study as it is the solvent used in the original work.[1] If not stated otherwise, solvent was not degassed and was shaken with air in vial or syringe to ensure that substantial amount of oxygen is dissolved in the analysed mixture.

2.2 MS and MS2 data and conditions

2.2.1 MS spectra acquired without irradiation

Except the total ion current change, no other change of spectrum was observed if small amount of water, hydrogen peroxide, toluene, 1,3-dimethoxybenzene, chlorobenzyl-alcohol or 4-chlorobenzyaldehyde was added to the solution of flavinium salt **1a**. Spectrum of pure flavinium salt is shown in Figure S5.

Sample and conditions used in Figure S5:

Sample: 0.7 mg of **1a** was dissolved in 4 ml of acetonitrile. 100 µl of this concentrated solution was then diluted by 1 ml of acetonitrile, transferred into the syringe and sprayed by syringe ionization technique. Conditions: flow rate 0.20 ml/h, spray voltage 4.5 kV, capillary temperature 150°C, Capillary voltage 20 V, Tube lens voltage 100 V.

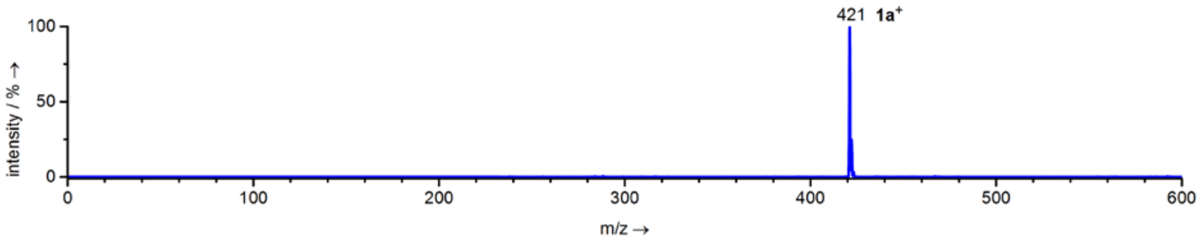

Figure S5. Spectrum of non-irradiated flavinium salt **1a**.

Conversely, in the case of charge-tagged derivatives **1b** and **1c**, in some cases pseudobase formed through a reaction with an additive is detected. The most important pseudobase is the one formed after hydrogen peroxide addition to the solution of flavinium salt as seen in the Figure S6 because it represents the charge-tagged hydroperoxyflavin **2**.

Samples and conditions used in Figure S6:

(a) Sample: 0.5 mg of **1b** was dissolved in 2 ml of acetonitrile. 100 µl of the concentrated solution was then diluted by 1 ml of acetonitrile, transferred into the syringe and sprayed by syringe ionization technique. Conditions: flow rate 0.20 ml/h, spray voltage 5 kV, emult 1100 V, sheath gas flow ~20, Capillary voltage 30 V, Tube lens voltage 70 V.

(b) Sample: sample used in (a) after addition of 100 µl of 4-chlorobenzyl alcohol solution (1.4 mg dissolved in 4 ml of acetonitrile (the spectrum still looked like in the case (a)) and 50 µl of concentrated (30%) H_2O_2 ([**1b**+H,2O]$^+$ formed). It was sprayed using the same spraying conditions as (a), except emult 1200 V.

(c) Sample: 2.6 mg of **1c** was partially dissolved in 0.6 ml of acetonitrile. 50 µl of the concentrated solution was then diluted by 2 ml of acetonitrile, transferred into the “overpressure unit” and sprayed. Conditions: spray voltage 4.5 kV, emult 1200 V, sheath gas flow ~25, capillary temperature 150°C, Capillary voltage 0 V, Tube lens voltage 60 V. The ion of m/z corresponding to the the precursor **S3b** described in Figure 1 is present as an impurity in the **1c**, from which we prepared the sample (confirmed by NMR).

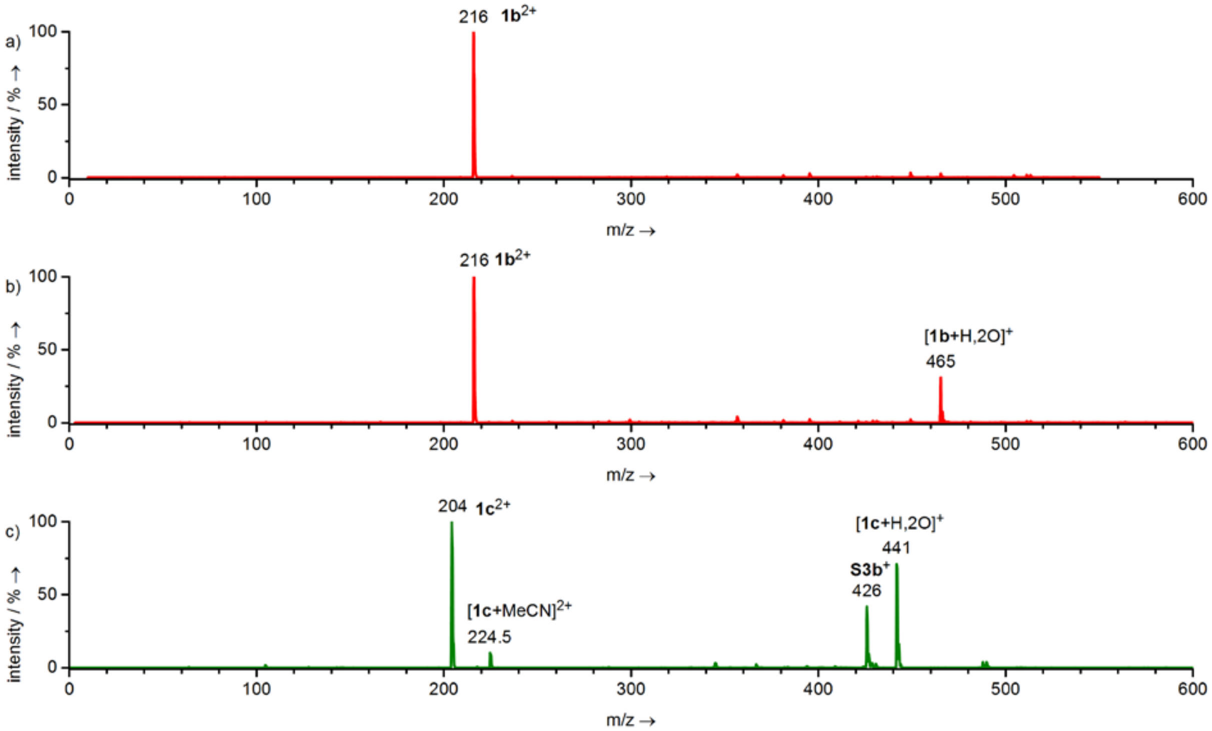

Figure S6. a) Spectrum of non-irradiated charge-tagged flavinium **1b** and b) the spectrum after addition of H_2O_2 to the sample used in a). c) Spectrum of the trimethylammonium charge tag **1c** + H_2O_2.

2.2.2 Experimental details of MS spectra acquired with sample irradiation under oxygen-free conditions

We have prepared an air-free sample by Schlenk-line technique and irradiated it by a home-made diode light-source (Luxeon STAR/0, 4 x 1030 mW@700 mA, 448 nm).

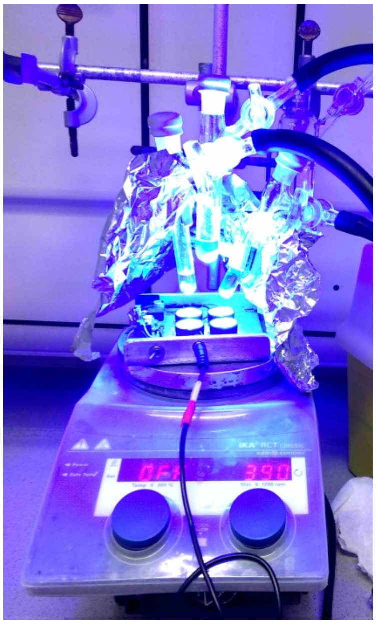

Figure S7. Photo of the irradiation setup.

Samples and conditions used in Figure **2** and **S8**:

In all oxygen-free conditions experiments, Schlenk tubes were dried by a heat-gun under vacuum and then cooled under Argon atmosphere to room temperature prior to use.

Conditions: spray voltage 4.5 kV, capillary temperature 275°C, capillary voltage 0 V, tube lens voltage 100 V, emult 1400 V, sheath gas 20, aux gas 35.

(a) Sample: 0.6 mg of **1a** and 8 µl of toluene-d_8 were dissolved in 1 ml of acetonitrile (dry). This solution was then degassed by 5 freeze-thaw cycles. Then the solution was irradiated for 5 minutes under Argon atmosphere. After the reaction the sample was diluted by 10 ml of acetonitrile (dry). To ensure minimal contact with external atmosphere, MS capillary, wire for ionization and N2 source for the overpressure injection of the sample were introduced through the septum. Conditions: spray voltage 4.5 kV, capillary temperature 275°C, Capillary voltage 0 V, Tube lens voltage 100 V, emult 1400 V, sheath gas 20, auxiliary gas 35.

(b) Sample: Sample from a) + 10 µl D_2O. Conditions as in (a)

(c) Sample: 0.9 mg of **1a** and 8 µl of toluene-d_5 were dissolved in 2 ml of acetonitrile (dry). This solution was then degassed by 5 freeze-thaw cycles. Then the solution was irradiated for 5 minutes under Argon atmosphere. After the reaction the sample was diluted by 10 ml of acetonitrile (dry). To ensure minimal contact with external atmosphere, MS capillary, wire for ionization and N2 source for the overpressure injection of the sample were introduced through the septum. Conditions: spray voltage 4.5 kV, capillary temperature 275°C, Capillary voltage 0 V, Tube lens voltage 80 V, emult 1400 V, sheath gas 8, auxiliary gas 0.

(d) Sample: Sample from c) + 10 µl D_2O in 100 µl of acetonitrile (dry). Conditions as in (c)

(e) Sample: 0.8 mg of **1a**, 8 µl of toluene-d_8 and 8 µl of toluene-d_0 were dissolved in 1 ml of acetonitrile (dry). This solution was then degassed by 5 freeze-thaw cycles. Then the solution was irradiated for 5 minutes under Argon atmosphere. After the reaction the 100 µl of the reaction mixture was diluted by 2 ml of acetonitrile (dry). MS experiment has been carried out in overpressure unit. Conditions: spray voltage 4.5 kV, capillary temperature 275°C, Capillary voltage 0 V, Tube lens voltage 100 V, emult 1200 V, sheath gas 21, auxiliary gas 35

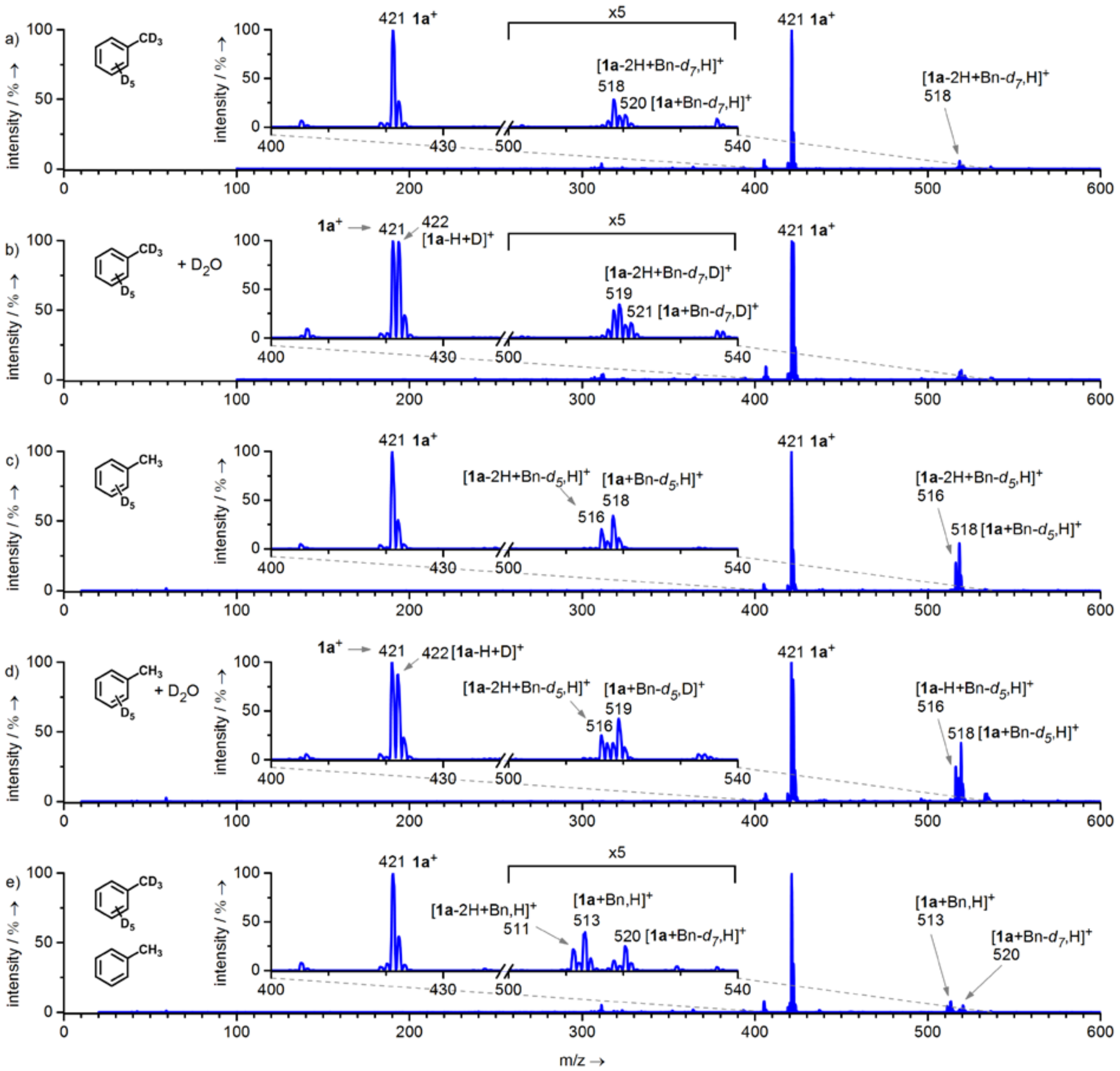

Figure S8. a) Spectrum of **1a**$^+$ and toluene-*d8* irradiated under anaerobic conditions b) (a) after addition of D2O c) spectrum of **1a**$^+$ and toluene-*d5* irradiated under anaerobic conditions d) (b) after addition of D2O e) spectrum of **1a**$^+$, toluene-*d8* and toluene-*d0* irradiated under anaerobic conditions.

2.2.3 MS spectra acquired with syringe irradiation method

When we irradiated the syringe, we have observed mainly formal adduct with oxygen. We observed this adduct in case that solution of sole flavinium salt or flavinium salt and water was irradiated (b). One hydrogen atom can be exchange for deuterium in this adduct. This exchange has been proved by addition of deuterium-labelled water (c). In case that 4-chlorobenzyl alcohol is added instead of water formal adduct of flavinium salt and water is observed (d).

In case of spectra (a) and (d) decrease of the total ion current is observed during irradiation. It can be explained by formation of water which then forms neutral flavin pseudobase. This effect is not observable in cases (b) and (c) as water is present from the beginning and total ion current remains relatively low, but constant over the time of the irradiation.

Samples and conditions used in Figure S9:

All spectra shown in Figure S9 were measured on the Finnigan LCQ Deca XP mass spectrometer. Blue laser diode current was set to 0.4 A. All spectra in this figure represent an average of the acquisition between 7-20 minutes after the start of the irradiation.

Conditions: All spectra were recorded at flow rate 0.44 ml/h, spray voltage 4 kV, capillary temperature 175°C, capillary voltage -50 V, Tube lens offset -50 V.

Samples:

a) Sample: 0.8 mg of **1a** was dissolved in 4 ml of acetonitrile. 100 µl of the concentrated solution was then diluted by 1 ml of acetonitrile, transferred into the syringe and sprayed by syringe ionization technique.

b) Sample: same as in (a) + 100 µl of H_2O.

c) Sample: same as in (a) + 100 µl of D_2O.

d) Sample: same as in (a) + 100 µl of 4-chlorobenzyl alcohol solution (1.4 mg dissolved in 4 ml of acetonitrile).

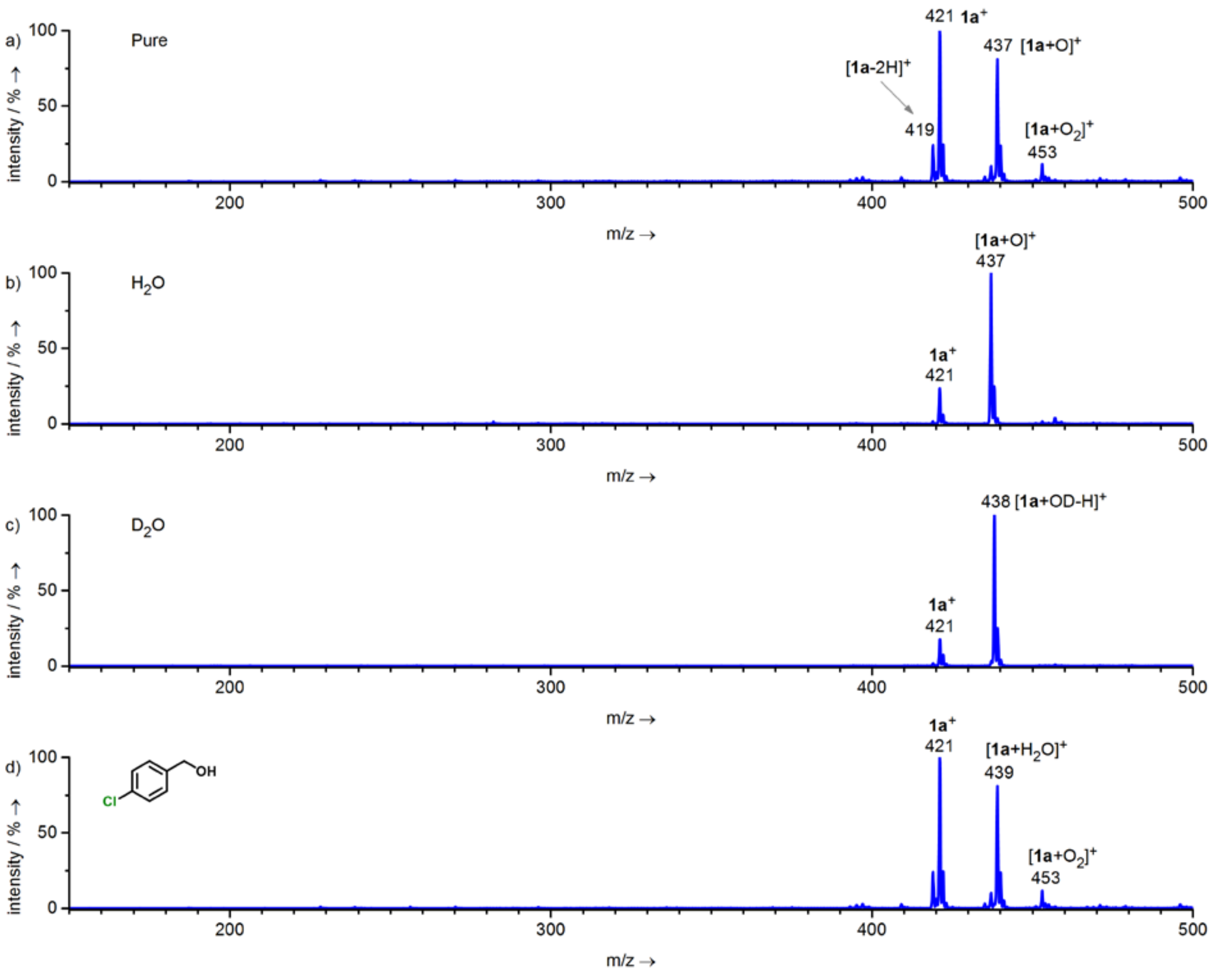

Figure S9. a) Spectrum of syringe-irradiated flavinium salt **1a** . b) Spectrum of syringe-irradiated flavinium salt **1a** + H_2O. c) Spectrum of syringe-irradiated flavinium salt **1a** + D_2O. d) Spectrum of syringe-irradiated flavinium salt **1a** + 4-chlorobenzyl alcohol.

As mentioned in the article, formal adduct with oxygen ([**1a**+O]$^+$) and water ([**1a**+H2O]$^+$) are present even after the irradiation is stopped as shown in Figure S10.

Sample and conditions used in Figure S10:

Sample: 0.5 mg of **1a** was dissolved in 4 ml of acetonitrile. 100 µl of the concentrated solution was then diluted by 1 ml of acetonitrile and 100 µl of 4-chlorobenzyl alcohol solution (1.4 mg dissolved in 4 ml of acetonitrile) was added.

Conditions: flow rate 0.40 ml/h, spray voltage 5 kV, capillary temperature 80°C, Capillary voltage 30 V, Tube lens voltage 110 V, emult was changed from 1200 to 1350 V during acquisition due to decreasing signal intensity. For the latter spectra when irradiation was stopped flow rate was decreased to 0.18 ml/h

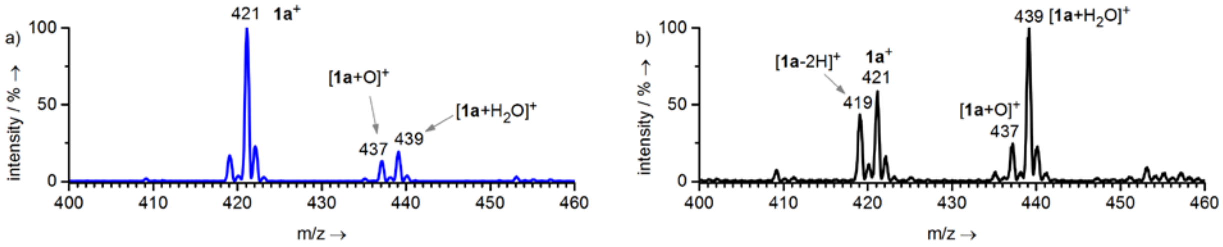

Figure S10. Spectrum (a) is an average of the acquisition between 3-6 minutes after the start of the irradiation. (b) Spectrum of the same sample after prolonged irradiation time and turning the light source off (emult was changed to 1800-2000 V). The impurities on the baseline are caused by relatively low intensity of the target ions and are most probably product of side-reactions during a photocatalysis. We speculate that [**1a**-2H]$^+$ is most probably a partially decomposed catalyst **1**.

2.2.4 MS spectra acquired with capillary irradiation method

We have tried a slight variation of the recently published capillary irradiation technique[2] where capillary is irradiated in front of the ESI head as illustrated in Figure S2b. However, it was not effective for our system.

Sample and conditions used in Figure S11:

Sample: 0.8 mg of **1a** was dissolved in 4 ml of acetonitrile. 100 µl of the concentrated solution was then diluted by 1 ml of acetonitrile and 100 µl of 4-chlorobenzyl alcohol solution (1.4 mg dissolved in 4 ml of acetonitrile) was added. Conditions: flow rate 0.20 ml/h, spray voltage 5 kV, capillary temperature 130°C, Capillary voltage 0 V, Tube lens voltage 60 V, emult 1400 V, syringe ionization technique applied.

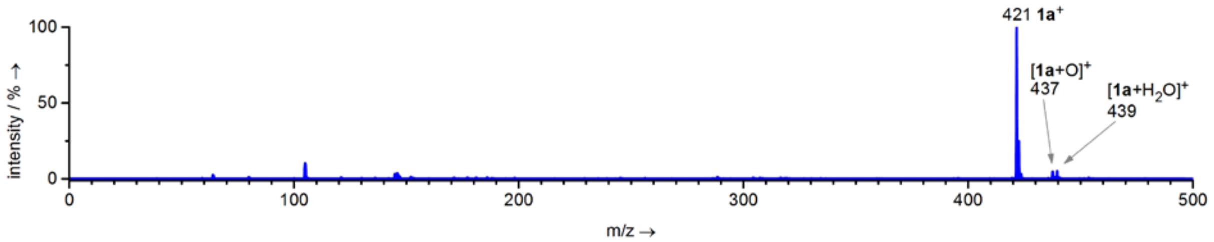

Figure S11. Spectrum acquired with irradiation applied to a capillary just before ESI-head entrance. Ion m/z 105 has fragmentation which fits to [Na+2CH_3CN]$^+$ cluster.

2.2.5 MS spectra acquired with capillary tip irradiation method

To observe reaction intermediates we developed our own system which was inspired by recently published nano-esi tip irradiation technique. [3] For our system liquid-ionization is crucial because when capillary is pulled out of the source as in our case (Fig S2c and d) classical tip irradiation is ineffective. By this system we have been finally able to see elusive reaction intermediates as illustrated in Figure S12.

During tip-irradiation we observed rapid drop of total ion current in virtually all cases. Subsequently relative intensity of trace impurities raise. To rule out possibility of misinterpretation of such impurity for emerging intermediate we have employed technique where for each spectrum of acquisition two scans were acquired. First scan was irradiated and the second was not. Then we have normalized our spectra to the total ion current of the average of non-irradiated averaged spectra and overlaid averaged irradiated spectrum with the non-irradiated one (pink dash-dotted lines in Figure S12). Thus minimize a chance of misinterpretation caused by total ion current shift induced by irradiation.

Samples and conditions used in Figure S12:

Sample (a, pure): 1.1 mg of **1a** was dissolved in 4 ml of acetonitrile. 50 µl of the concentrated solution was then diluted by 1 ml of acetonitrile. Conditions: flow rate 0.15 ml/h, spray voltage 5 kV, capillary temperature 150°C, Capillary voltage 0 V, Tube lens voltage 60 V, emult 1200 V, syringe ionization technique applied.

Sample (b, H_2O): 0.8 mg of **1a** was dissolved in 4 ml of acetonitrile. 100 µl of the concentrated solution was then diluted by 1 ml of acetonitrile and 100 µl of H_2O was added. Conditions: flow rate 0.40 ml/h, spray voltage 4.5 kV, capillary temperature 50°C, Capillary voltage 0 V, Tube lens voltage 110 V, emult 1300 V, syringe ionization technique applied.

Sample (c, D_2O): 1.3 mg of **1a** was dissolved in 4 ml of acetonitrile. 100 µl of the concentrated solution was then diluted by 1 ml of acetonitrile and 50 µl of D_2O was added. Conditions: flow rate 0.15 ml/h, spray voltage 5.5 kV, capillary temperature 150°C, Capillary voltage 0 V, Tube lens voltage 60 V, emult 1200 V, sheath gas 8, auxiliary gas 40, syringe ionization technique applied.

Sample (d, H_2O_2): 100 µl of the concentrated solution used in (c) was diluted by 1 ml of acetonitrile and 100 µl of 30% H_2O_2 was added. Conditions: flow rate 0.15 ml/h, spray voltage 5.5 kV, capillary temperature 150°C, Capillary voltage 0 V, Tube lens voltage 60 V, emult 1200 V, sheath gas 8, auxiliary gas 0, syringe ionization technique applied.

Sample (e, 4-chlorobenzyl alcohol): 1.1 mg of **1a** was dissolved in 4 ml of acetonitrile. 100 µl of this concentrated solution was diluted by 1 ml of acetonitrile and 100 µl of 4-chlorobenzyl alcohol solution (1.4 mg dissolved in 4 ml of acetonitrile) was added. Conditions: spray voltage 5 kV, capillary temperature 150°C, Capillary voltage 0 V, Tube lens voltage 60 V, emult 1400 V, sheath gas 8, auxiliary gas 0, syringe ionization technique applied.

Sample (f, toluene): 1.1 mg of **1a** was dissolved in 4 ml of acetonitrile. 50 µl of this concentrated solution was diluted by 1 ml of acetonitrile and 20 µl of toluene solution (8.8 mg dissolved in 4 ml of acetonitrile) was added. Conditions: flow rate 0.20 ml/h, spray voltage 5 kV, capillary temperature 100°C, Capillary voltage 0 V, Tube lens voltage 60 V, emult 1200 V, sheath gas 10, auxiliary gas 0, syringe ionization technique applied.

Sample (g, dimethoxybenzene): 1.0 mg of **1a** was dissolved in 4 ml of acetonitrile. 100 µl of this concentrated solution was diluted by 1 ml of acetonitrile and 100 µl of toluene solution (5.6 mg dissolved in 4 ml of acetonitrile) was added. Conditions: flow rate 0.18 ml/h, spray voltage 5 kV, capillary temperature 100°C, Capillary voltage 0 V, Tube lens voltage 100 V, emult 1200 V, sheath gas 28, auxiliary gas 0, syringe ionization technique applied.

Sample (h, benzene): 0.9 mg of **1a** was dissolved in 4 ml of acetonitrile. 200 µl of this concentrated solution was diluted by 2 ml of acetonitrile and 200 µl of benzene solution (8.0 mg dissolved in 4 ml of acetonitrile) was added. Conditions: spray voltage 4.5 kV, capillary temperature 270°C, Capillary voltage 0 V, Tube lens voltage 80 V, emult 1500 V, sheath gas 8, auxiliary gas 0, overpressure applied.

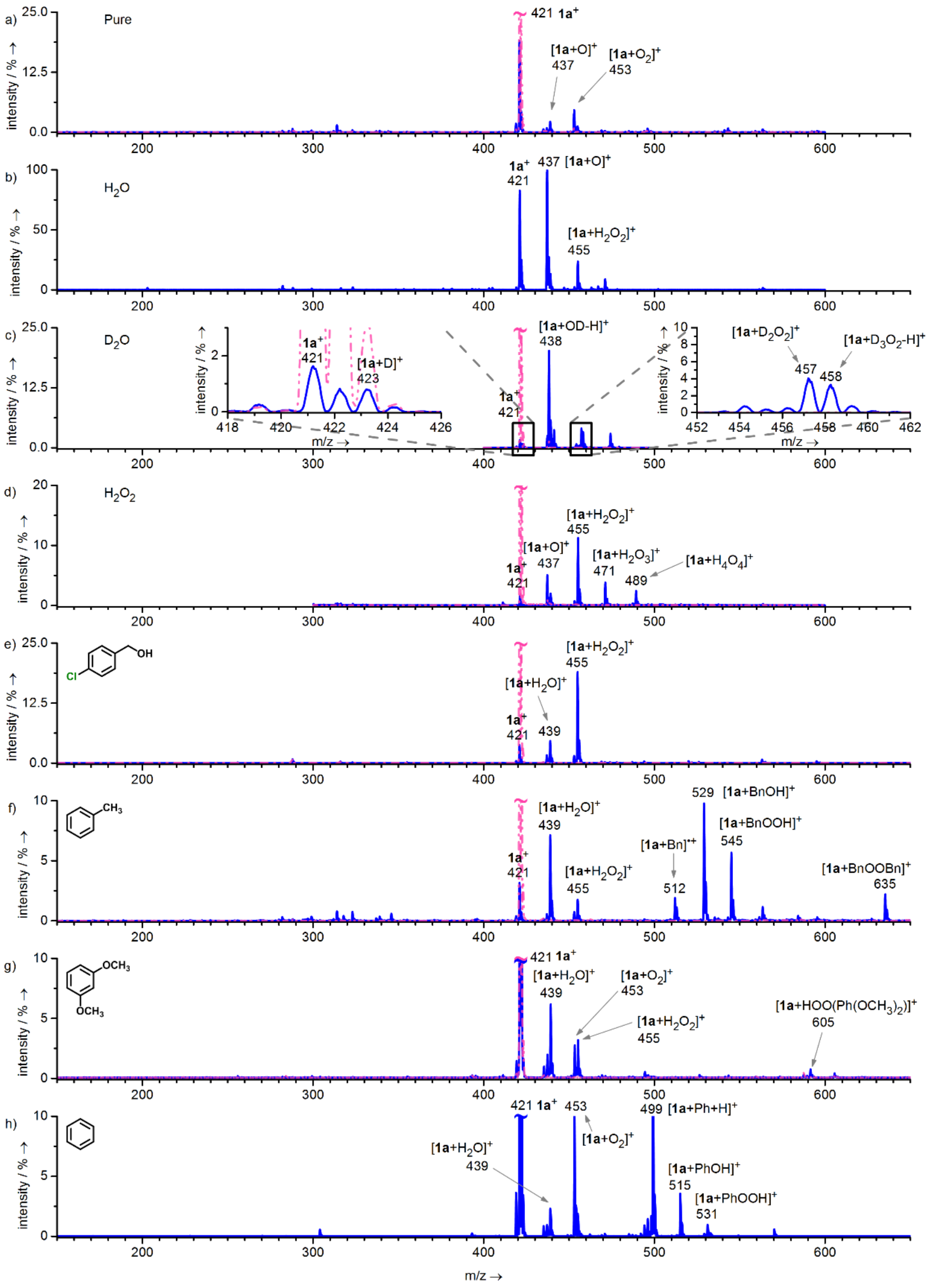

Figure S12. Spectrum acquired with irradiation applied to the tip of a capillary a) and spectra after addition either of water b), deuterated water c), hydrogen peroxide d), 4-chlorobenzyl alcohol e), toluene f), or dimethoxybenzene g) to the sole flavinium salt **1a** solution.

We have employed the same technique to the charge-tagged analogues as illustrated in Figure S13.

Samples and conditions used in Figure S13:

Sample (a, pure): 0.5 mg of **1b** was dissolved in 2 ml of acetonitrile. 100 µl of the concentrated solution was then diluted by 1 ml of acetonitrile. Conditions: flow rate 0.20 ml/h, spray voltage 5 kV, capillary temperature 100°C, Capillary voltage 30 V, Tube lens voltage 70 V, emult 1400 V, syringe ionization technique applied.

Sample (b, 4-chlorobenzyl alcohol): 100 µl of 4-chlorobenzyl alcohol solution (1.4 mg dissolved in 4 ml of acetonitrile) was added to the sample used in a). Conditions: flow rate 0.20 ml/h, spray voltage 5 kV, capillary temperature 100°C, Capillary voltage 30 V, Tube lens voltage 70 V, syringe ionization technique applied.

Sample (c, 4-chlorobenzyl alcohol and H_2O_2): 100 µl of 30% H_2O_2 was added to the sample used in b). Conditions: flow rate 0.20 ml/h, spray voltage 5 kV, capillary temperature 150°C, Capillary voltage 0 V, Tube lens voltage 60 V, syringe ionization technique applied.

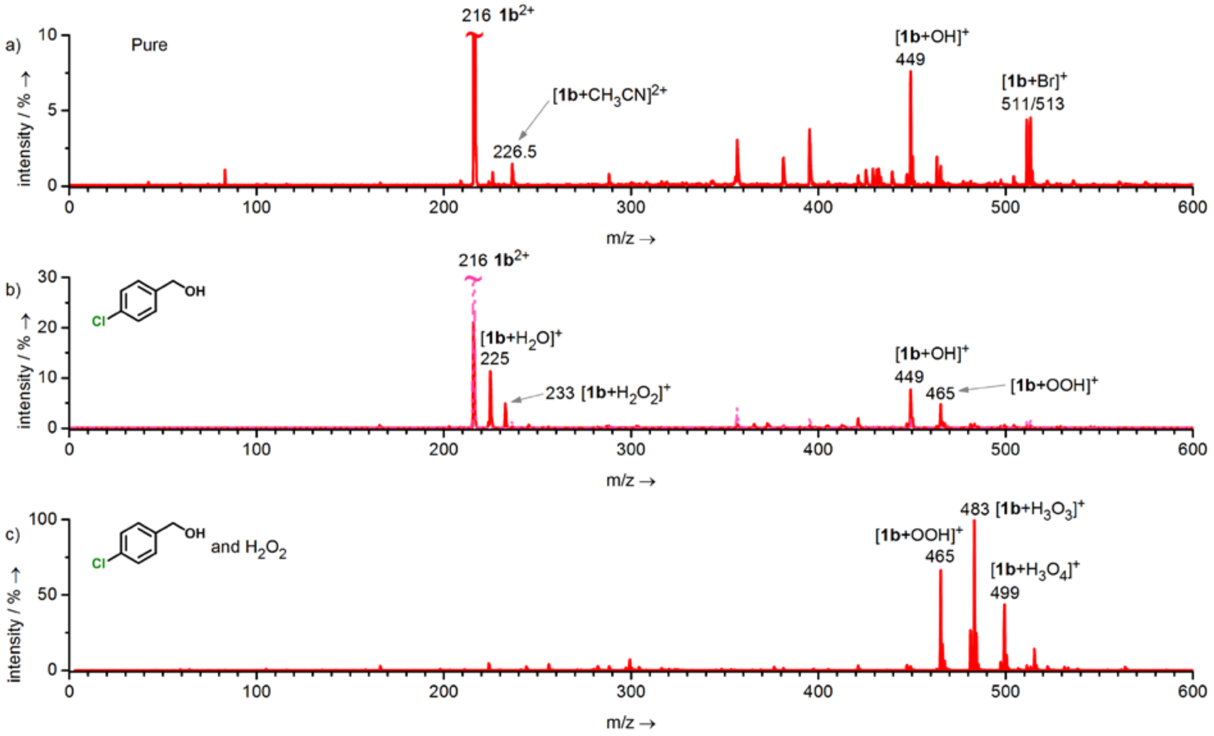

Figure S13. Spectrum acquired with irradiation applied to the tip of a capillary a) and spectra after addition of 4-chlorobenzyl alcohol b) and after subsequent addition of hydrogen peroxide c), to the sole flavinium salt **1b** solution. The formal hydroperoxy adduct shown in b) is most probably hydroxyl adduct of oxidized catalyst as illustrated in the fragmentation section.

Samples and conditions used in figure S14:

Sample (a, vial): 1 mg of **1b** was dissolved in 4 ml of acetonitrile. 200 µl of the concentrated solution was diluted by 2 ml of acetonitrile and toluene solution (5.5 mg dissolved in 4 ml of acetonitrile) was added. Conditions: spray voltage 4.5 kV, capillary temperature 250°C, Capillary voltage 30 V, Tube lens voltage 60 V, emult 1400 V, sheath gas 10, auxiliary gas 0, overpressure applied, vial irradiated by LED

Sample (b, tip): same as in (a), capillary tip irradiation applied instead of vial irradiation.

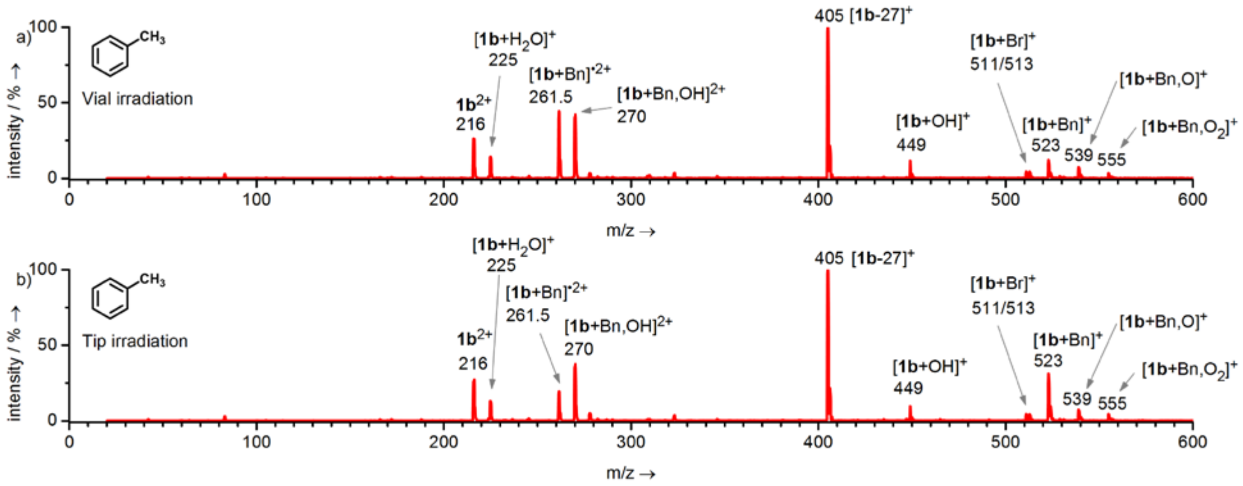

Figure S14. Mixture of **1b** and toluene in acetonitrile irradiated a) in vial and b) at the capillary tip.

2.2.6 Time evolution of the spectra

To estimate the timescale on which we are operating we have sequentially irradiated two distinguished points on the capillary tip. The distance between points was approximately 2-3 mm, which corresponds to time difference of ~300-450 milliseconds under the flow which we have used. The irradiation points are described in Figure S15, the spectra in Figure S16.

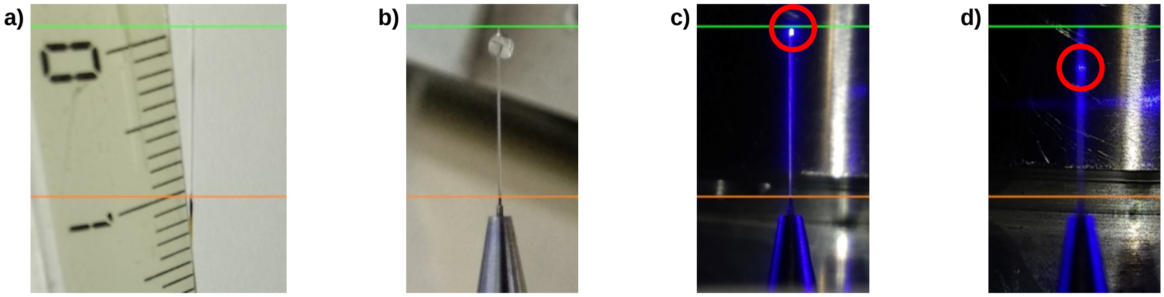

Figure S15. a) de-coated capillary tip with a ruler b) detail of the capillary in the ESI head c) first point of irradiation (in red) c) second point of irradiation (in red) located approximately 2mm from the tip**.**

Samples and conditions used in Figure S16:

Sample (a, tip): 1.5 mg of **1a** was dissolved in 4 ml of acetonitrile. 200 µl of the concentrated solution was diluted by 2 ml of acetonitrile and toluene solution (8.3 mg dissolved in 4 ml of acetonitrile) was added. Conditions: spray voltage 4.5 kV, capillary temperature 250°C, Capillary voltage 0 V, Tube lens voltage 60 V, emult 1500 V, sheath gas 12, auxiliary gas 0, syringe irradiation technique applied, flow 0.2 ml/h capillary tip irradiated by laser.

Sample (b, almost tip): same as in (a), point of irradiation moved slightly back.

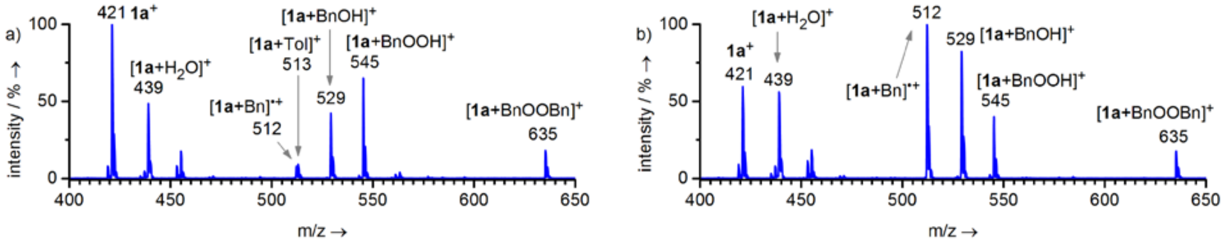

Figure S16. Mixture of **1a** and toluene in acetonitrile irradiated a) at the capillary tip b) approximately 2 mm behind the tip. Notice the huge difference in the intensity of [**1a**+Bn]$^{\bullet+}$ peak.

2.2.7 Fragmentation of the selected ions

To further describe ions observed by us we performed their fragmentation by collisions with xenon gas (CID = collision induced dissociation). In all cases, the structures of parent and daughter ions are merely hypothetical and should be regarded as possible interpretation of the m/z. We have started by identification of oxo-intermediates present after irradiation of the original mixture or addition of water (Figure S17). If not stated otherwise all observed parent ions were generated by capillary-tip irradiation. It is apparent that original flavinium **1a**$^+$ and its adduct [**1a**+O]$^+$ both undergoes McLafferty rearrangement with neutral loss of octene with similar intensity. This leads us to hypothesis that oxidation occurred on the aromatic moiety or at the ethylene bridge. Conversely the adduct [**1a**+O_2]$^+$ has completely different and complex fragmentation pattern which has not been assigned.

Samples and conditions used in Figure S17:

a) Sample: 1.4 mg of **1a** was dissolved in 4 ml of acetonitrile. 200 µl of the concentrated solution was then diluted by 2 ml of MeCN. Conditions: Overpressure unit, spray voltage 4.5 kV, capillary temperature 250 °C, Capillary voltage 0 V, Tube lens voltage 100 V, p(Xe) = 0.10 (uncorrected, ion gauge) mTorr, E_{coll}(center-of-mass) = 9.3 eV.

b) Sample: as in (a) + 50 µl of H_2O. Conditions: Overpressure unit, spray voltage 4.5 kV, capillary temperature 250 °C, Capillary voltage 0 V, Tube lens voltage 120 V, p(Xe) = 0.09 (uncorrected, ion gauge) mTorr, E_{coll}(center-of-mass) = 9.0 eV.

c) Sample: as in (a). Conditions: Overpressure unit, spray voltage 4.5 kV, capillary temperature 250 °C, Capillary voltage 0 V, Tube lens voltage 120 V, p(Xe) = 0.07 mTorr (uncorrected, ion gauge), E_{coll}(center-of-mass) = 8.7 eV.

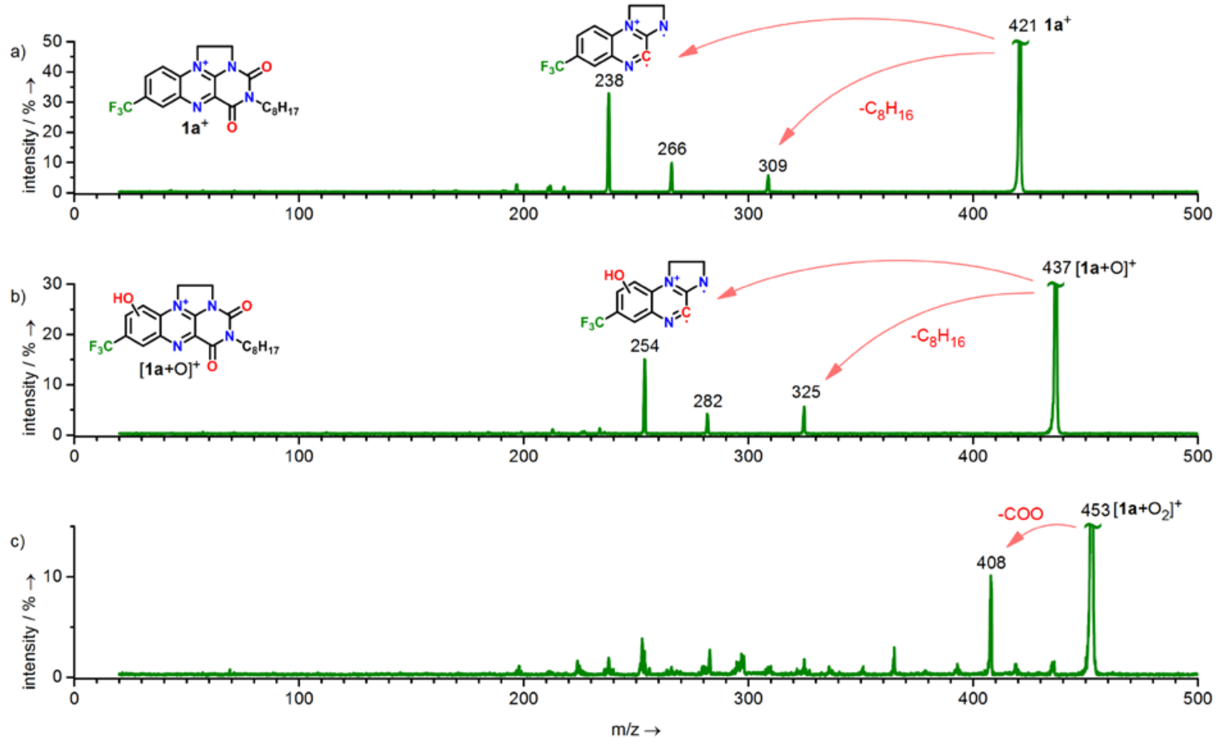

Figure S17. Fragmentation of original flavinium ion a), its formal adduct with oxygen atom b) and with oxygen molecule c).

We performed fragmentation of **1a**$^+$-benzyl adducts observed in oxygen-free experiments. In the case of D2O addition there is clearly visible that the [**1a**+Bn(d7),D]$^+$ fragments with the same intensity as the [**1a**+Bn(d7),H]$^+$ ion in the spectrum prior to the D2O addition, whereas the other ions fragments with much lower intensity under the same conditions.

Samples and conditions used in Figure S18:

a) Sample: 0.8 mg of **1a**, 8 µl of toluene and 8 µl of toluene-d8 were dissolved in 2 ml of acetonitrile and reacted in a same way as described in paragraph 2.2.2. Instead of dilution of the reaction mixture in Schlenk tube, 100 µl of this sample was transferred into vial and diluted by 2 ml of CH3CN (under N2 atmosphere). Conditions: spray voltage 4.5 kV, capillary temperature 275 °C, capillary voltage 0, tube lens voltage 100 V, overpressure applied. p(Xe) = 0.12 mTorr (uncorrected, ion gauge, transmission at ZCE 86%), E_{coll}(center-of-mass) = 5.0 eV.

b) Sample: Sample described in paragraph 2.2.2. b) Conditions: spray voltage 4.5 kV, capillary temperature 275 °C, capillary voltage 0, tube lens voltage 80 V, sheath gas 20, auxiliary gas 35, overpressure applied. p(Xe) = 0.11 mTorr (uncorrected, ion gauge, transmission at ZCE 88%), E_{coll}(center-of-mass) = 4.9 eV.

c) Sample and conditions same as in b). p(Xe) = 0.11 mTorr (uncorrected, ion gauge, transmission at ZCE 88%), E_{coll}(center-of-mass) = 4.9 eV.

d) Sample and conditions same as in b). p(Xe) = 0.11 mTorr (uncorrected, ion gauge, transmission at ZCE 88%), E_{coll}(center-of-mass) = 4.9 eV.

e) Sample and conditions same as in a). p(Xe) = 0.12 mTorr (uncorrected, ion gauge, transmission at ZCE 86%), E_{coll}(center-of-mass) = 4.9 eV.

d) Sample and conditions same as in b). p(Xe) = 0.11 mTorr (uncorrected, ion gauge, transmission at ZCE 88%), E_{coll}(center-of-mass) = 4.9 eV.

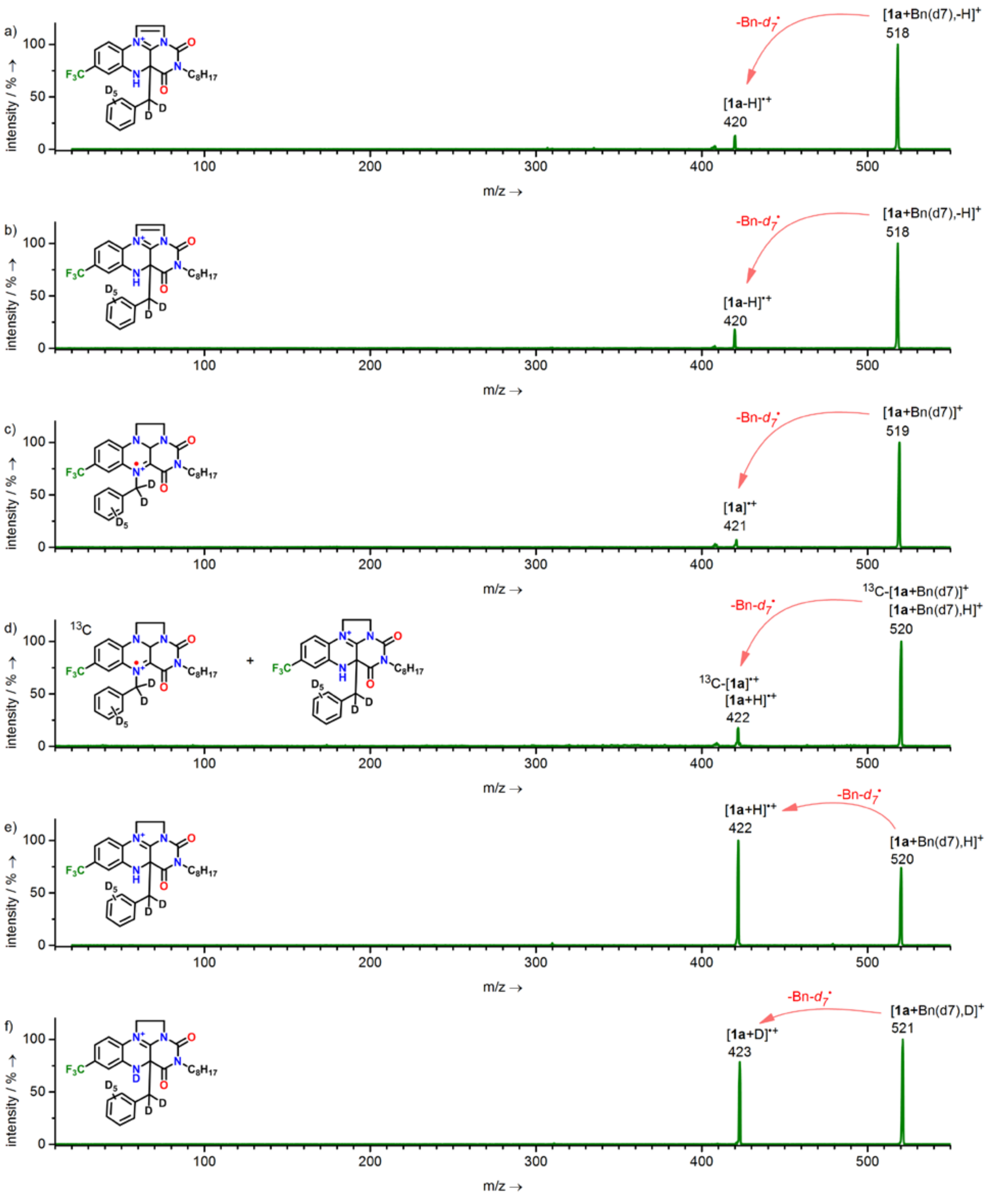

Figure S18. Fragmentation of the a) [**1a**+Bn(d7),-H]+, b) [**1a**+Bn(d7),-H]+, c) [**1a**+Bn(d7)]+, d) [**1a**+Bn(d7)](13C)+, e) [**1a**+Bn(d7),H]+ and f) [**1a**+Bn(d7),D]+.

We have also performed CID of formal water and hydrogen peroxide adducts summed in Figure S19 and fragmentation of adducts with aromatics summed in Figure S20.

Samples and conditions used in Figure S19:

a) Sample: 1.1 mg of **1a** was dissolved in 4 ml of acetonitrile. 50 µl of this concentrated solution was diluted by 1 ml of acetonitrile and 20 µl of toluene solution (8.8 mg dissolved in 4 ml of acetonitrile) was added. Conditions: flow rate 0.20 ml/h, spray voltage 5 kV, capillary temperature 100°C, Capillary voltage 0 V, Tube lens voltage 60 V, emult 1800 V, sheath gas 10, auxiliary gas 0, syringe ionization technique applied. p(Xe) = 0.20 mTorr, E_{coll}(center-of-mass) = 5.8 eV.

b) Sample and conditions from a). p(Xe) = 0.20 mTorr, E_{coll}(center-of-mass) = 5.6 eV.

c) Sample: 100 µl of concentrated solution from a) was diluted by 1 ml of acetonitrile and 50 µl of D_2O was added. Conditions: flow rate 0.15 ml/h, spray voltage 5 kV, capillary temperature 150°C, Capillary voltage 0 V, Tube lens voltage 60 V, emult 2000 V, sheath gas 15, auxiliary gas 30, syringe ionization technique applied. p(Xe) = 0.20 mTorr, E_{coll}(center-of-mass) = 5.6 eV.

d) Sample and conditions from c). p(Xe) = 0.20 mTorr, E_{coll}(center-of-mass) = 5.6 eV.

e) Sample: Sample: 1.2 mg of **1a** was dissolved in 4 ml of acetonitrile. 200 µl of this concentrated solution was diluted by 2 ml of acetonitrile and 100 µl of 30% H_2O_2 was added. Conditions: Overpressure unit, spray voltage 4.5 kV, capillary temperature 250 °C, Capillary voltage 0 V, Tube lens voltage 60 V, p(Xe) = 0.13 mTorr, E_{coll}(center-of-mass) = 4.9 eV.

f) Sample: 1.3 mg of **1a** was dissolved in 4 ml of acetonitrile. 100 µl of this concentrated solution was diluted by 1 ml of acetonitrile and 100 µl of 30% H_2O_2 was added. Conditions: flow rate 0.15 ml/h, spray voltage 5.5 kV, capillary temperature 150°C, Capillary voltage 0 V, Tube lens voltage 60 V, emult 2000 V, sheath gas 8, auxiliary gas 0, syringe ionization technique applied. p(Xe) = 0.20 mTorr, E_{coll}(center-of-mass) = 3.2 eV.

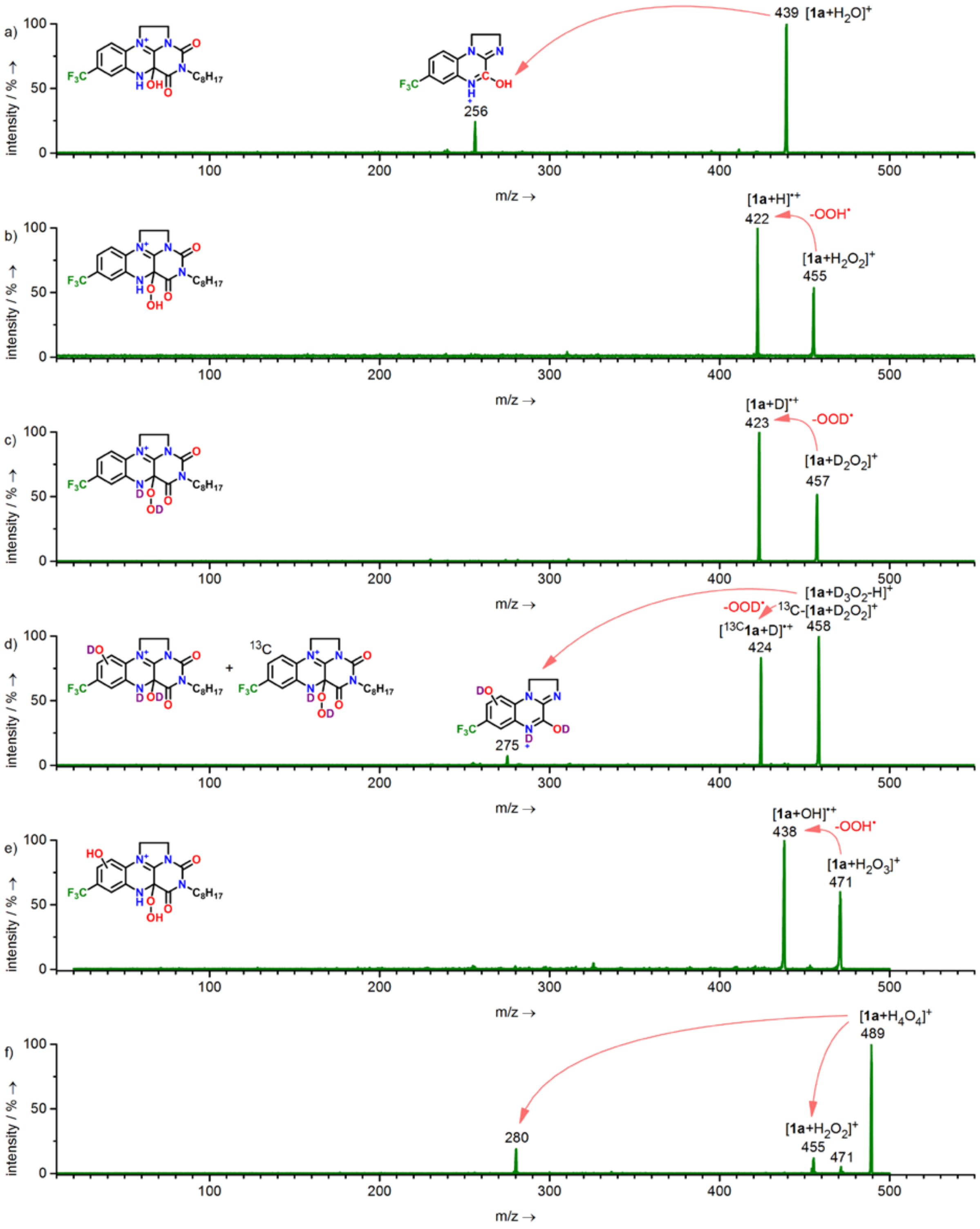

Figure S19. Fragmentation of the a) [**1a**+2H,O]+, b) [**1a**+2H,2O]+, c) [**1a**+2D,2O]+, d) [**1a**+3D,2O]+, e) [**1a**+2H,3O]+ and f) [**1a**+4H,4O]+.

Samples and conditions used in Figure S20:

a) Sample: 1.1 mg of **1a** was dissolved in 4 ml of acetonitrile. 50 µl of this concentrated solution was diluted by 1 ml of acetonitrile and 20 µl of toluene solution (8.8 mg dissolved in 4 ml of acetonitrile) was added. Conditions: flow rate 0.20 ml/h, spray voltage 5 kV, capillary temperature 100°C, Capillary voltage 0 V, Tube lens voltage 60 V, emult 1800 V, sheath gas 10, auxiliary gas 0, syringe ionization technique applied. p(Xe) = 0.20 mTorr, E_{coll}(center-of-mass) = 5.0 eV.

b) Sample: 0.9 mg of **1a** was dissolved in 4 ml of acetonitrile 200 µl of this concentrated solution was diluted by 2 ml of acetonitrile and 200 µl of benzene solution (8 mg dissolved in 4 ml of acetonitrile) was added. Conditions: Overpressure unit, spray voltage 4.5 kV, capillary temperature 275 °C, Capillary voltage 0 V, Tube lens voltage 60 V, p(Xe) = 0.12 mTorr, E_{coll}(center-of-mass) = 4.8 eV.

c) Sample: 1.4 mg of **1a** was dissolved in 4 ml of acetonitrile 200 µl of this concentrated solution was diluted by 2 ml of acetonitrile and 100 µl of toluene solution (5.5 mg dissolved in 4 ml of acetonitrile) was added. Conditions: spray voltage 4.5 kV, capillary temperature 250 °C, Capillary voltage 0 V, Tube lens voltage 60 V, p(Xe) = 0.13 mTorr, E_{coll}(center-of-mass) = 4.4 eV.

d) Sample: as in (c) Conditions: spray voltage 4.5 kV, capillary temperature 250 °C, Capillary voltage 0 V, Tube lens voltage 60 V, p(Xe) = 0.09 mTorr, E_{coll}(center-of-mass) = 3.9 eV.

e) Sample: 1.4 mg of **1a** was dissolved in 4 ml of acetonitrile 200 µl of this concentrated solution was diluted by 2 ml of acetonitrile and 100 µl of 1,3-dimethoxybenzene solution (5.6 mg dissolved in 4 ml of acetonitrile) was added. Conditions spray voltage 4.5 kV, capillary temperature 250 °C, Capillary voltage 0 V, Tube lens voltage 81 V, p(Xe) = 0.13 mTorr, E_{coll}(center-of-mass) = 4.1 eV.

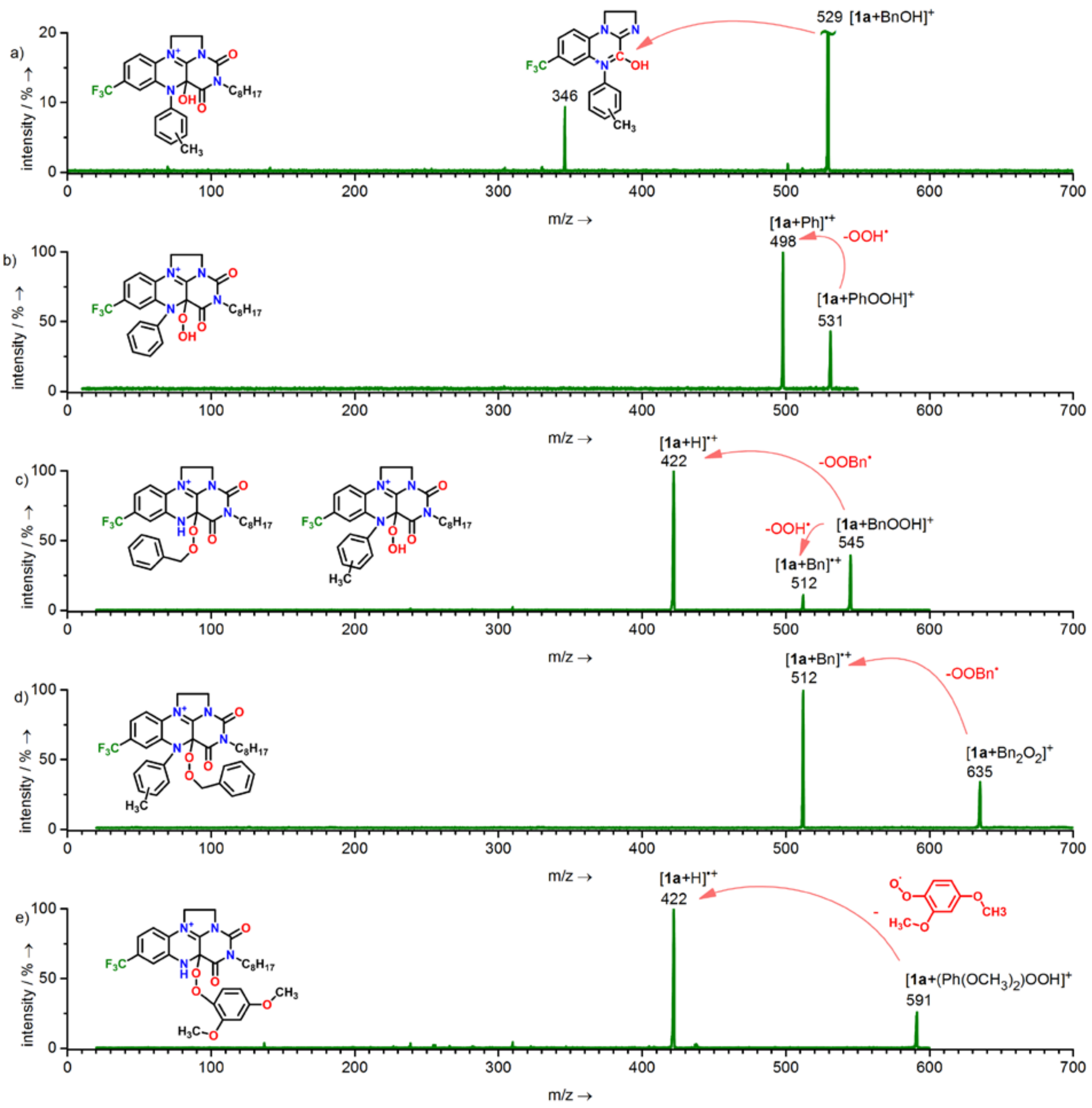

Figure S20. Fragmentation of the a) [**1a**+Bn,H,O]$^+$, b) [**1a**+H,Ph,2O]$^+$, c) [**1a**+H,PhCH$_3$,2O]$^+$ + [**1a**+Bn,H,2O]$^+$, d) [**1a**+PhCH$_3$,Bn,2O]$^+$, e) [**1a**+(MeO)$_2$Ph,H,2O]$^+$.

2.2.8 Fragmentation in the source

When we hardened the source conditions we were able to generate fragments of OOH$^•$ (m=33) radical loss directly in the source which was used for IRPD experiments. This is illustrated on [**1b**+OOH]$^+$ in-source fragmentation to **1b**$^{•+}$ in figure S21

Samples and conditions used in Figure S21:

Sample (a): 0.5 mg of **1a** was dissolved in 2 ml of acetonitrile. 100 µl of this concentrated solution was diluted by 1 ml of acetonitrile and sprayed. Conditions: flow rate 0.20 ml/h, spray voltage 5 kV, capillary temperature 150°C, Capillary voltage 80 V, Tube lens voltage 160 V, emult 1200 V, sheath gas 20, auxiliary gas 0, syringe ionization technique applied.

Sample (b): 1.0 mg of **1b** was dissolved in 4 ml of acetonitrile. 200 µl of this concentrated solution was diluted by 2 ml of acetonitrile and 100 µl of 35% H_2O_2 was added. Conditions: Overpressure unit, spray voltage 4.5 kV, capillary temperature 250 °C, Capillary voltage 0 V, Tube lens voltage 60 V, p(Xe) = 0.13 mTorr, E_{coll}(center-of-mass) = 4.9 eV.

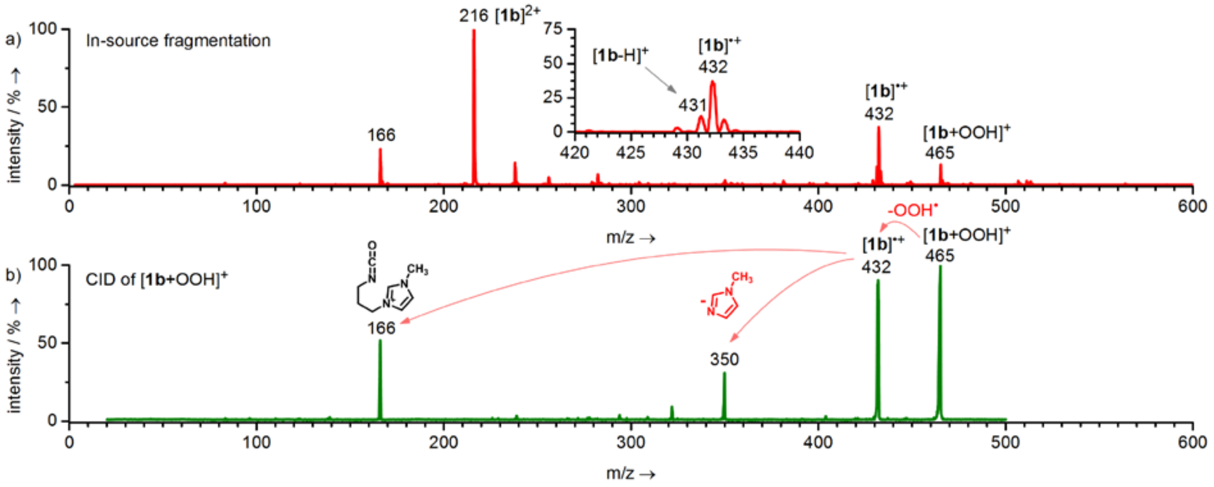

Figure S21. Fragmentation of the [**1b**+OOH]$^+$ a) in source under harsh conditions b) in the collision cell.

2.2.9 Reactivity of the selected ions

2.2.9.1 Reactivity of 1a+H2O2

We have measured the gas-phase reactivity of **1a**+H2O2. We have observed a reaction with dimethyl sulphide. To document the reactivity, we have recorded multiple spectra with varying pressure and collision energy. For comparison we also recorded spectra using unreactive xenon gas instead of dimethyl sulphide. Results of these experiments (Figure S24) indicate that the OOH (m=33) neutral loss is a fragmentation channel and that O (m=16) neutral loss is a reactivity channel.

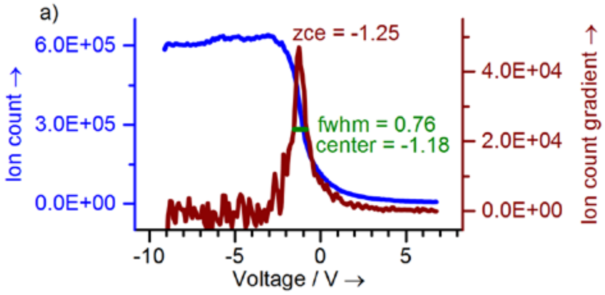

Figure S22. Energy distribution of the **1a**+H2O2 ion. Determination of the zero collision energy (zce).

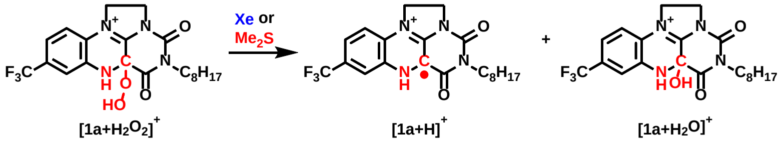

Figure S23. Scheme of the main reactivity and fragmentation channels of the **1a**+H2O2 ion.

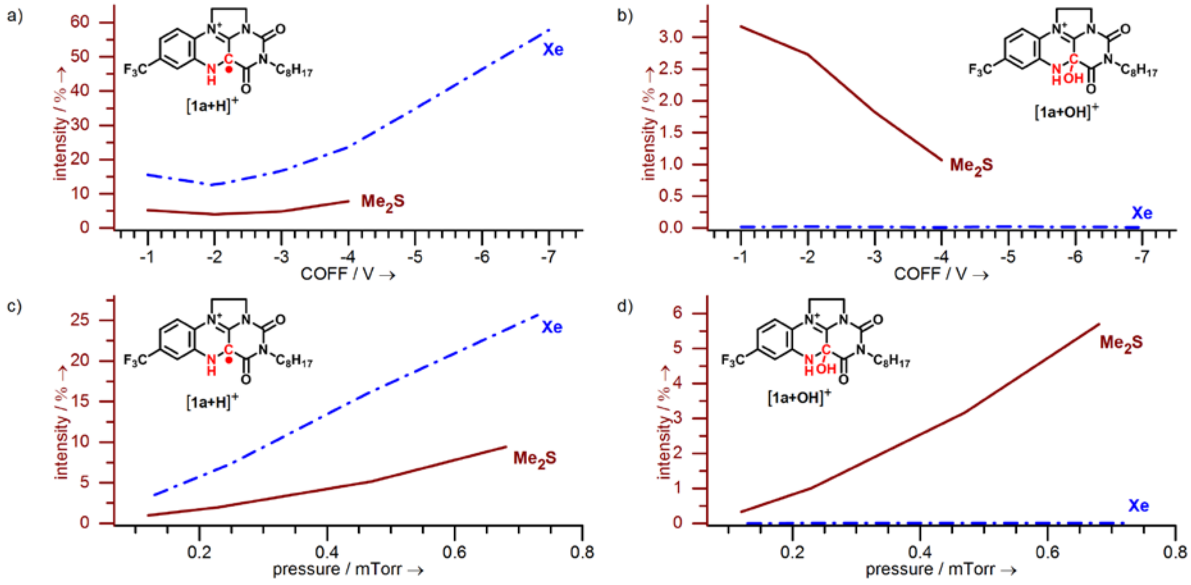

Figure S24. Relative intensity of fragmentation channels based on the pressure and collision offset variation. The relative intensity has been obtained as intensity of fragmentation channel divided by total ion current. Noise has been subtracted from both values prior to the calculation. a) Collision offset dependence of OOH (m=33) neutral loss; b) Collision offset dependence of O (m=16) neutral loss; c) pressure dependence of OOH (m=33) neutral loss; d) pressure dependence of O (m=16) neutral loss. Xenon (blue dash-dotted lines) or dimethyl sulphide (full red lines) has been used as a collision gas.

2.2.9.2 Reactivity of 1b+H_2O_2

We have also recorded gas-phase reactivity of charge-tagged analogue 1b+H_2O_2 with dimethyl sulphide in analogy to abovementioned experiments with 1a+H_2O_2. The fragmentation/reactivity pattern is the same. Only difference is that in the charge-tagged species we observed visible a single-charged fragments. These fragments are most probably experimental artefacts as they are present even in collisions with unreactive xenon. We hypothesize that these artefacts are caused by an unknown persistent impurity present in the collision cell. In analogy to 1a+H_2O_2, the results of the experiments with 1b+H_2O_2 (Figure S27) indicate that the OOH (m=16.5) neutral loss is a fragmentation channel and that O (m=8) neutral loss is a reactivity channel.

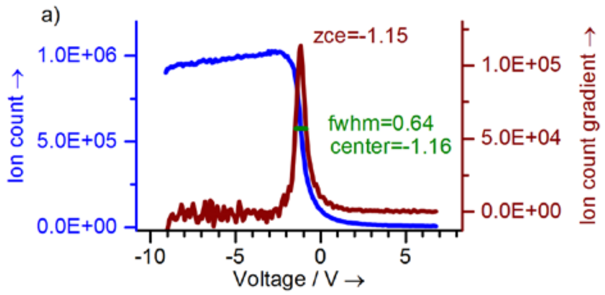

Figure S25. Energy distribution of the **1b**+H2O2 ion. Determination of the zero collision energy (zce).

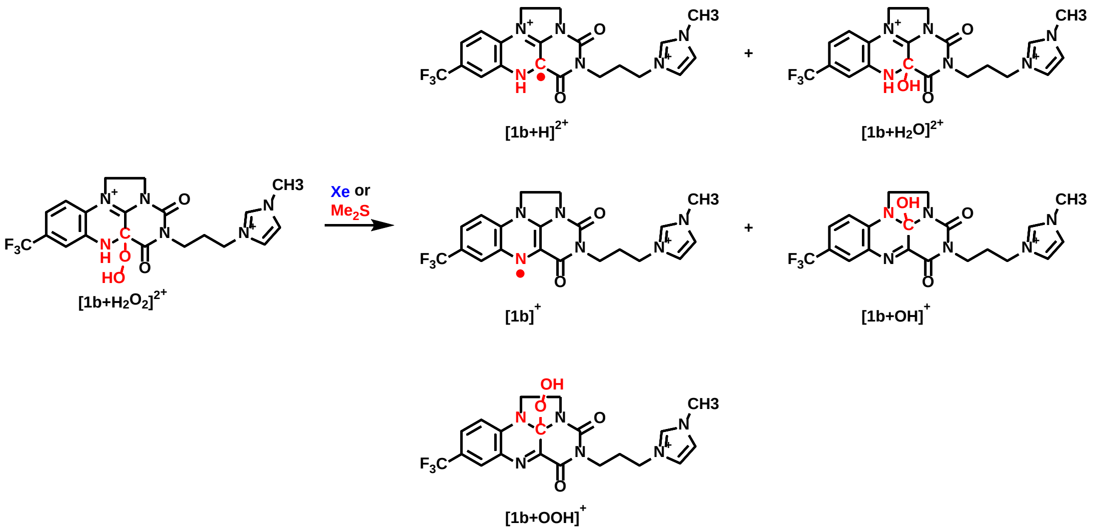

Figure S26. Scheme of the main reactivity and fragmentation channels of the **1b**+H2O2 ion.

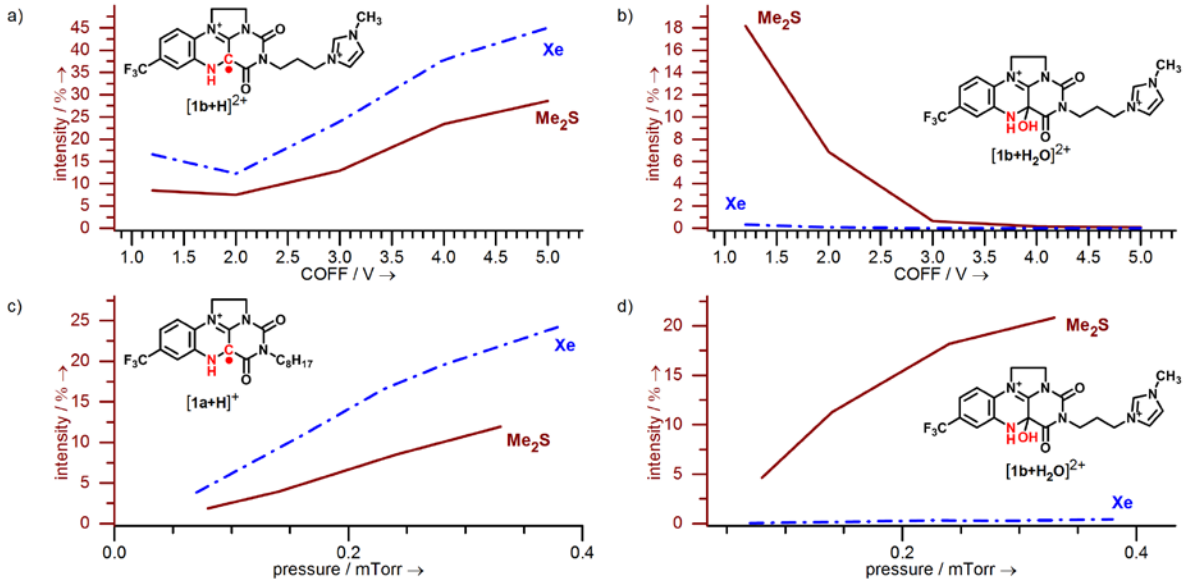

Figure S27. Relative intensity of fragmentation channels based on the pressure and collision offset variation. The relative intensity has been obtained as intensity of fragmentation channel divided by total ion current. Noise has been subtracted from both values prior to the calculation. a) Collision offset dependence of OOH (m=33) neutral loss; b) Collision offset dependence of O (m=16) neutral loss; c) pressure dependence of OOH (m=33) neutral loss; d) pressure dependence of O (m=16) neutral loss. Xenon (blue dash-dotted lines) or dimethyl sulphide (full red lines) has been used as a collision gas.

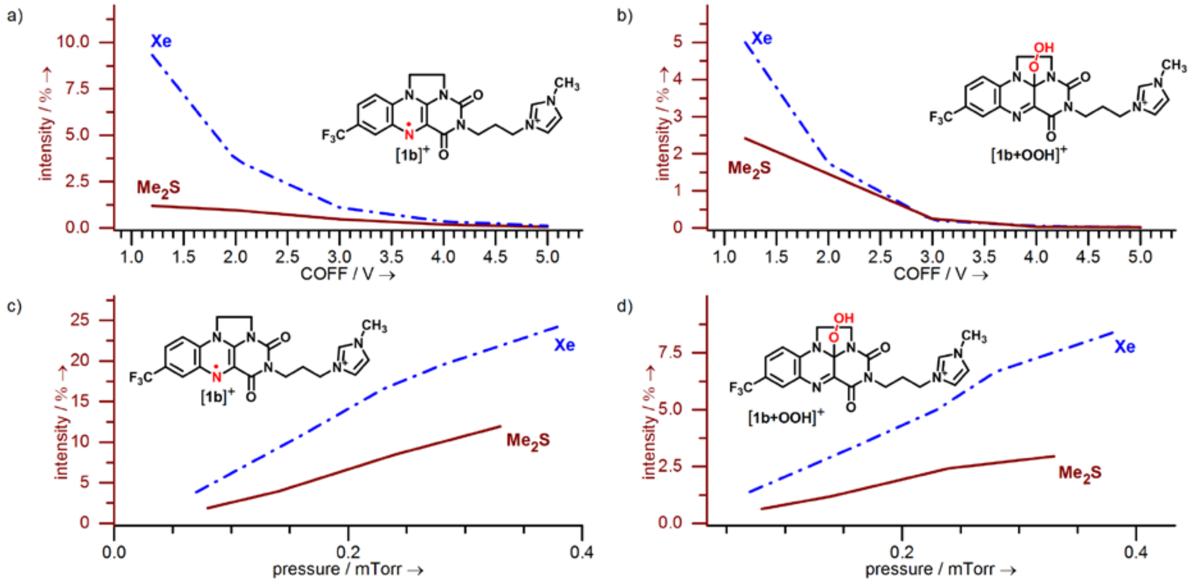

Figure S28. Relative intensity of artefact fragmentation channels based on the pressure and collision offset variation. The relative intensity has been obtained as intensity of fragmentation channel divided by total ion current. Noise has been subtracted from both values prior to the calculation. a) Collision offset dependence of H2O2^{+} (m=34) loss; b) Collision offset dependence of H^{+} (m=1) loss; c) pressure dependence of H2O2^{+} (m=34) loss; d) pressure dependence of H^{+} (m=1) loss. Xenon (blue dash-dotted lines) or dimethyl sulphide (full red lines) has been used as a collision gas.

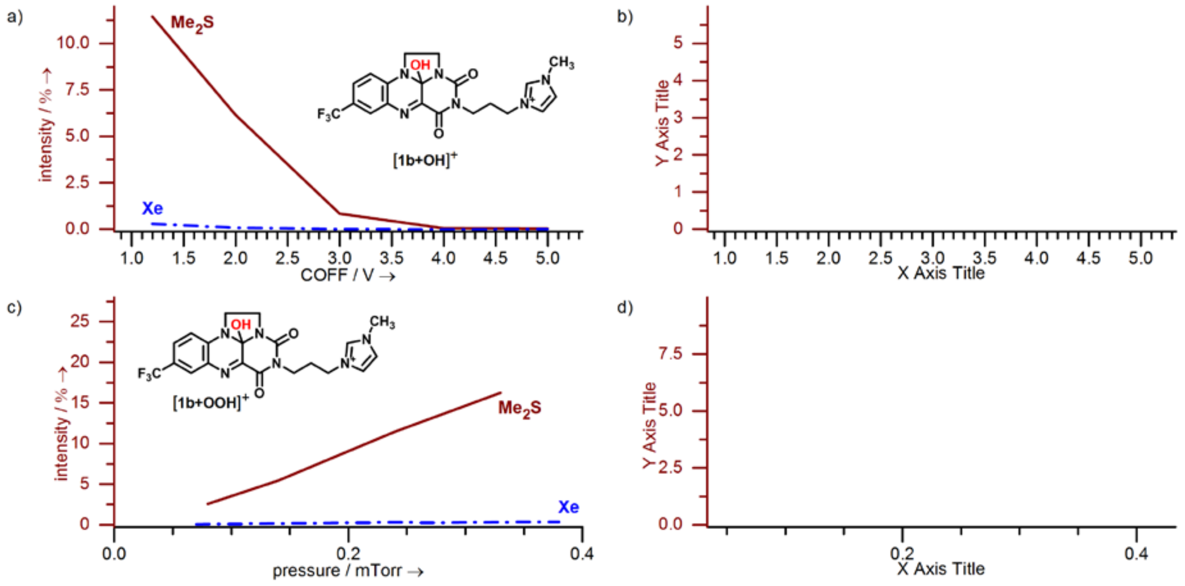

Figure S29. Relative intensity of artefact fragmentation channels based on the pressure and collision offset variation. The relative intensity has been obtained as intensity of fragmentation channel divided by total ion current. Noise has been subtracted from both values prior to the calculation. a) Collision offset dependence of OH^{+} (m=17) loss; c) Collision offset dependence of OH^{+} (m=17) loss. Xenon (blue dash-dotted lines) or dimethyl sulphide (full red lines) has been used as a collision gas.

2.2.9.3 Reactivity of 1b+OOH

We did not observed any reactivity with dimethyl sulphide in the case of 1b+OOH ion (Figure S30), which is in a strict contrast to abovementioned cases. However, both the fragmentation (neutral loss of OOH) and the IRPD spectra of this ion suggest that this ion is truly a flavin-hydroperoxide. We speculate, that the absence of the reactivity is caused by a steric hindrance of the active site by bulky imidazolium group, or by the fact, that nucleophilic dimethyl sulphide is attracted by the charge tag, thus cannot react with the hydroperoxy moiety.

Sample and conditions used in Figure S30:

Sample : 0.5 mg of **1b** was dissolved in 2 ml of acetonitrile. 100 µl of this concentrated solution was diluted by 2 ml of acetonitrile and 100 µl of 35% H_2O_2 and 100 µl of chlorobenzyl alcohol solution (1.4 mg dissolved in 4 ml of acetonitrile) were added. Conditions: Syringe ionization technique, spray voltage 5 kV, capillary temperature 175 °C, Capillary voltage 30 V, Tube lens voltage 100 V, p(Me_2S) = 0.40 mTorr, zero collisional energy.

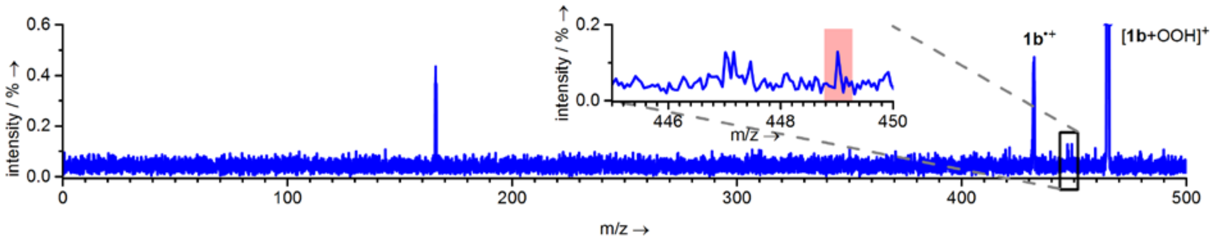

Figure S30. Spectrum of collisions between 1b+OOH ion and dimethyl sulphide at zero-collision energy. Notice the absence of the neutral O loss (m/z 449) fragment in the spectrum. In the intersection, the spike present in the position m/z 449 (red box) comes just from one scan of the whole acquisition.

3 Determination of the KIE from the MS experiments

The KIE was determined from repeated experiments illustrated in Figure 2c,d of the main article and Figure S31. First, we have evaluated the toluene d_0/d_5 experiments (Figure S31a). We have used signals of the peroxy intermediates [**1a**+Bn,H,2O]$^+$ (*m/z* 545) and [**1a**+Bn-d_5,H,2O]$^+$ (*m/z* 550, Δ*m/z* = +5) as a reporter of the actual concentration of toluenes d_0, and d_5. To obtain the KIE of the aromatic activation of the substrate we have compared signals of [**1a**+Bn]$^{•+}$ (*m/z* 512) and [**1a**+Bn-d_4]$^{•+}$ (*m/z* 516, Δ*m/z* = +4) ions giving KIE of 1.38 ± 0.04. Separately, we have evaluated the set of the [**1a**+Bn,H,O]$^+$ (*m/z* 529) and [**1a**+Bn-d_4,H,O]$^+$ (*m/z* 534, Δ*m/z* = +4) ions giving KIE of 1.17 ± 0.02. We have used averaged value of the peak height for the calculations. Uncertainties have been established as standard deviations.

Then we evaluated toluene d_0/d_8 experiments (Figure S31b). We have used signals of the hydroxy intermediates [**1a**+Bn,H,O]$^+$ (*m/z* 529) and [**1a**+Bn-d_7,H,O]$^+$ (*m/z* 536, Δ*m/z* = +7) as a reporter of the actual concentration of toluenes d_0, and d_8 based on KIE value acquired in previous experiment as it has much better ratio reproducibility compared to [**1a**+Bn]$^{•+}$. Then we have evaluated the KIE of the formation of [**1a**+Bn,H,2O]$^+$ (*m/z* 545, Δ*m/z* = +7) and [**1a**+Bn-d_7,H,2O]$^+$ (*m/z* 552, Δ*m/z* = +7) ions. The KIE value for [**1a**+Bn,H,2O]$^+$ formation was 1.64 ± 0.20.

When we calculated the KIE of [**1a**+Bn]$^{•+}$ channel from d_0/d_8 experiments, using [**1a**+Bn,H,O]$^+$ as a reporter, we have obtained KIE of 1.14 ± 0.05, which is almost the same value as the KIE of the reporter ion ([**1a**+Bn,H,O]$^+$). This experiment clearly shows that the accuracy of the calculation is lower than anticipated by stdev, but at the same time, it shows that the KIE of the formation of ([**1a**+Bn,H,O]$^+$) and [**1a**+Bn,H,2O]$^+$ is almost the same, and it differ rapidly from the KIE of the formation of [**1a**+Bn,H,2O]$^+$ ion. We believe that the [**1a**+Bn] $^{•+}$ KIE is value is less accurately determined as in case of d_0/d_5 experiments as the isotopic pattern of [**1a**+Bn-d_0/d_4]$^+$ and [**1a**+Bn-d_0/d_7]$^+$ ion pairs differ (Figure S31c and f), which is in contrast to the rest of the ion pairs (Figure S31d,e,g and h). In summary, we report KIE of 1.2 ± 0.1 for aromatic C-H/D activation as value

determined from [**1a**+Bn,H,O]$^+$ ion pair and KIE of 1.6 ± 0.1 for benzylic C-H/D activation as value determined from [**1a**+Bn,H,2O]$^+$ ion pair.

Samples and conditions used in Figure S31:

Sample (a): To 100 µl of toluene-d_0 solution (8.4 mg dissolved in 4 ml of acetonitrile) and 100 µl of toluene-d_5 solution (8.7 mg dissolved in 4 ml of acetonitrile) diluted by 2 ml of acetonitrile 200 µl of **1a** solution (1.2 mg of **1a** dissolved in 4 ml of acetonitrile) was added. Conditions: spray voltage 4.5 kV, capillary temperature 275 °C, Capillary voltage 0 V, Tube lens voltage 80 V, emult=1200 V, sheath gas 8, auxiliary gas 0, tip irradiation by LED laser.

Sample (b): To 100 µl of toluene-d_0 solution (8.4 mg dissolved in 4 ml of acetonitrile) and 100 µl of toluene-d_8 solution (9.4 mg dissolved in 4 ml of acetonitrile) diluted by 2 ml of acetonitrile 200 µl of **1a** solution (1.2 mg of **1a** dissolved in 4 ml of acetonitrile) was added. Conditions: spray voltage 4.5 kV, capillary temperature 275 °C, Capillary voltage 0 V, Tube lens voltage 80 V, emult=1500 V, sheath gas 8, auxiliary gas 0, tip irradiation by LED laser.

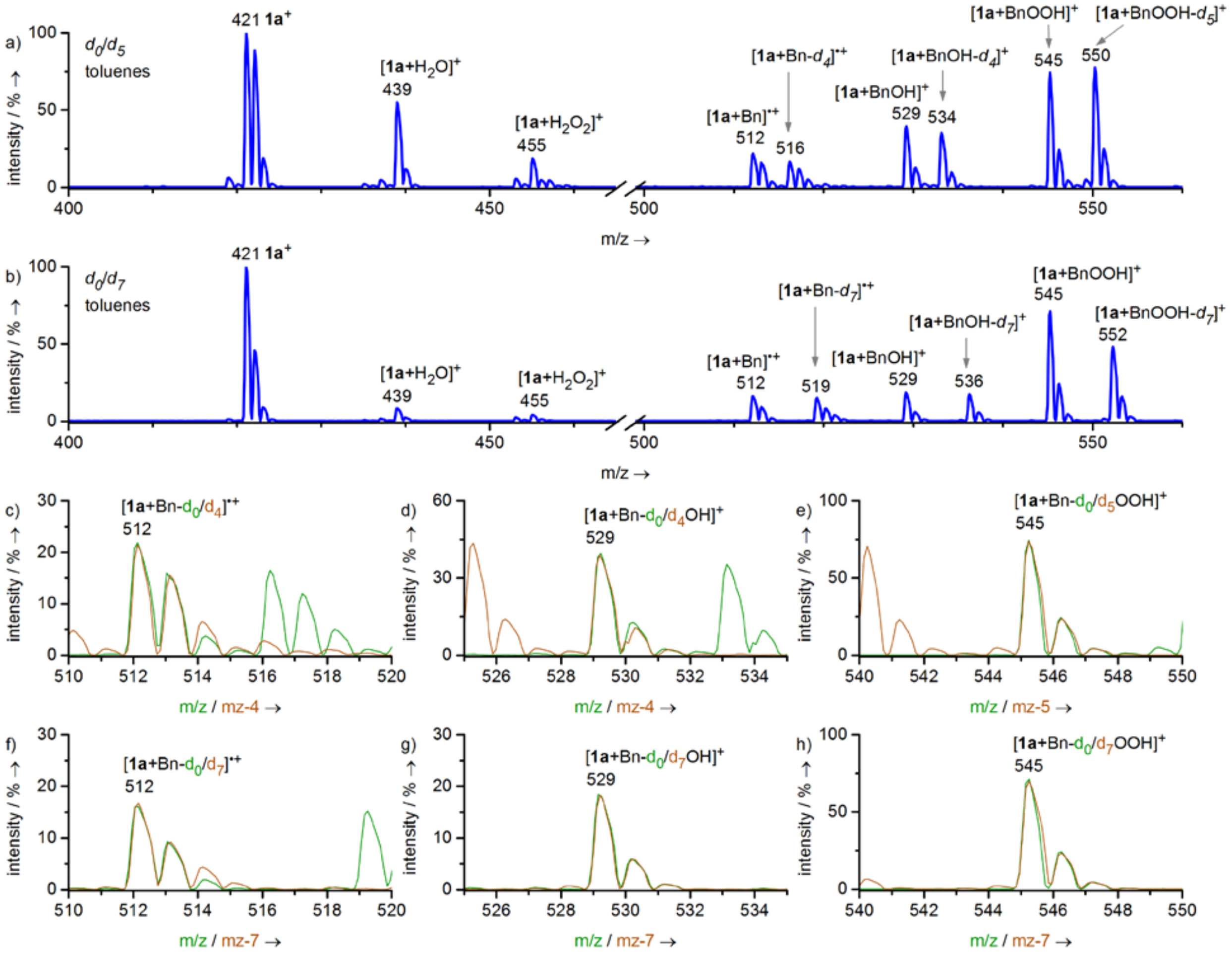

Figure S31. Determination of KIE by a peak-height: a) Mixture of d_0 and d_5 toluenes used as substrates b) analogous experiment with d_0 and d_7 toluenes. Isotopic pattern overlap of c) [**1a**+Bn-d_0/d_4]$^+$, d) [**1a**+Bn-d_0/d_4OH]$^+$, e) [**1a**+Bn-d_0/d_4OOH]$^+$, f) [**1a**+Bn-d_0/d_7]$^+$, g) [**1a**+Bn-d_0/d_7OH]$^+$, h) [**1a**+Bn-d_0/d_7OOH]$^+$. In cases of pattern overlap, intensities of deuterated channels have been renormalized.

4 IRPD and VIS-PD experiments, DFT calculations, UV-VIS

4.1 Experimental Details

Ion spectroscopy (IRPD) technique has been described elsewhere in detail (see citations in the main article). For the generation of ions, the capillary tip irradiation technique described in the mass spectrometry section was used. Samples were prepared in a way as described in previous mass spectrometry sections. Ionization conditions have been almost the same to the one described in mass spectrometry section. Special attention has been paid to keeping the voltage of transfer quadrupole of TSQ-7000 part of our IRPD machine at higher voltage than the first lens to ensure that the ions do not pre-trap in transfer quadrupole as otherwise it impairs the spectrum. When possible, the background check has been carried out by observing a sudden drop of signal of the desired photo-generated ion, when LED laser irradiating the silica capillary tip has been shut down.

The general procedure for IRPD, VIS and two-colour experiments is described in Figure S32. The conditions varies slightly at each case, mainly in a change of helium pulses or time how long the quadrupole bender guides ions to the trap. These changes are done exclusively to ensure the stability of a signal to give a reasonable signal to noise ratio. In most cases the cycle time (N) has been 1 second.

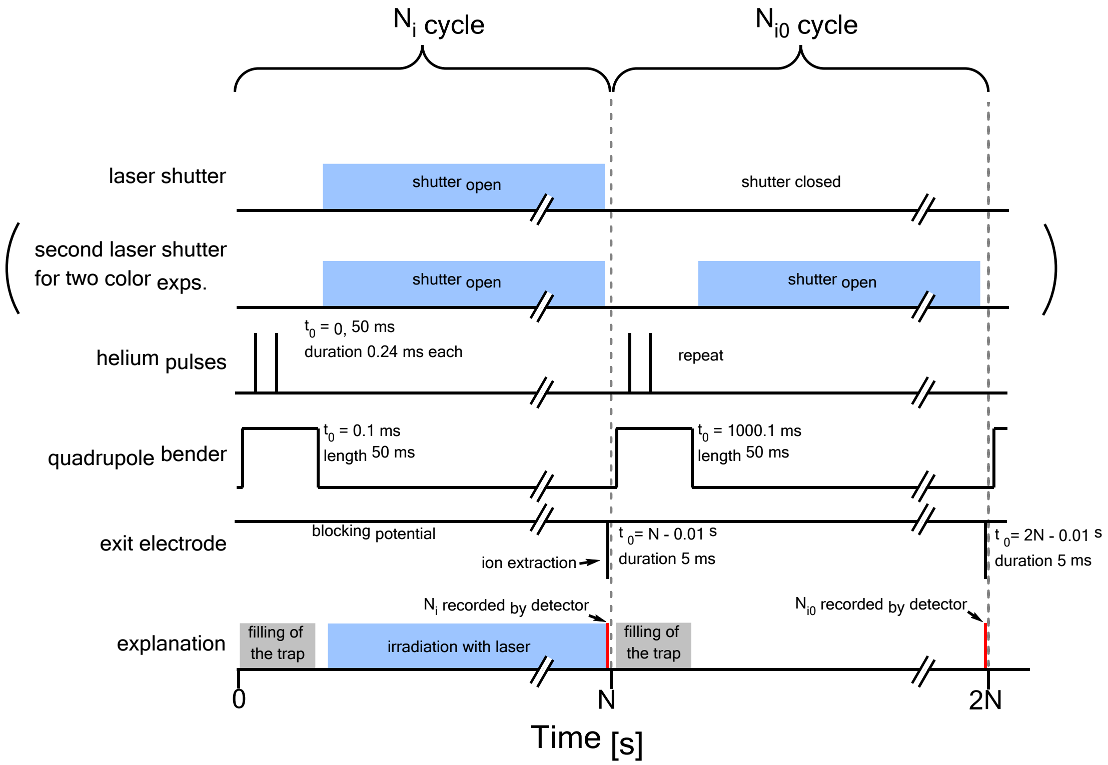

Figure S32. Pulse sequence of IRPD experiments. Second shutter was opened only in case of two-colour experiments, otherwise the laser and the shutter was not part of the setup. General Experimental procedure for one-color experiments: Ions were mass selected by a first quadrupole and guided into wired 4-pole trap. There they were cooled to 3K by helium buffer gas. At this temperature and pressure, the ions forms clusters with helium. These clusters are then irradiated at various wavelengths by IR or visible range tunable laser(s). Usually energy of one absorbed photon is enough to dissociate the helium from the ion and thus reporting absorption at specific wavelength. We measure the whole spectrum by varying the wavelength of the irradiation, reporting the dissociation yield ($1-N_i/N_{i0}$) as a function of absorption coefficient at specific wavelength. The N_{i0} cycle serves as a reference value for the number of cluster formed. In case of two-colour experiments used in this article, we use second laser to deplete population of specific isobaric impurity by targeting a wavelength where the impurity absorbs and the target ion does not.

4.2 Computational Details

If not stated otherwise the calculations were done by the B3Lyp DFT functional with the D3 version of Grimme´s dispersion with Becke-Johnson damping on the 6-311+G** basis in Gaussian G09. For spectra presented in the main article in figures **4** and **5**, carbon atom in the CF3 group was described by an additional PC-3 basis set for a better prediction of the electron dispersion. Structures pre-optimizations were carried out by AM1 and further refined by the B97 DFT functional on the 6-31+G basis set. If not stated otherwise, the methylated analogue of **1a** has been calculated instead of the original octylated one. Scaling factor for vibrational spectra stays the same as in the main article.

4.2.1 Computational dihedral angle study of ([1c+H_2O_2]$^+$) and ([1c+H_2O]$^+$)

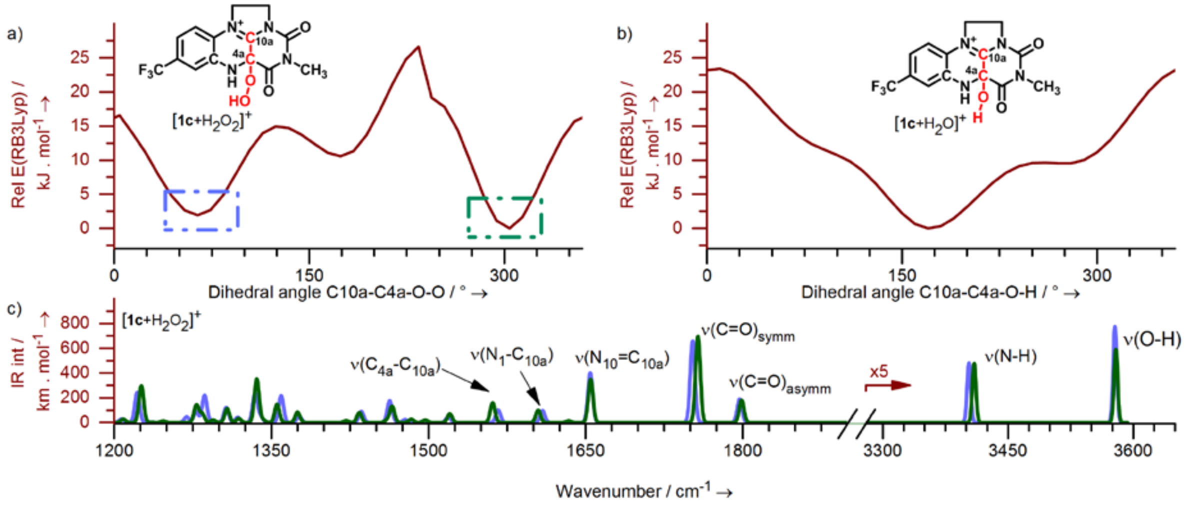

Figure S33. Dihedral angle study of C4a-hydroperoxyhydroflavinium ([**1c**+H_2O_2]$^+$) (a) and of C4a-hydroxyhydroflavinium ([**1c**+H_2O]$^+$) (b). Rotation was carried out by 10° steps and structure optimization was carried out at each step. Blue and green rectangles in graph (a) describe two possible stable conformers of ([**1c**+H_2O_2]$^+$). The vibrational spectra of these two conformers are plotted into graph (c). Calculations were carried out without additional PC-3 basis set.

4.2.2 Computational study of different conformers of ([1b+OOH]$^+$)

To determine most stable conformer of [**1b**+OOH]$^+$ we have performed series of DFT calculation. We quickly discovered that the most stable conformers are ones in which the imidazolium charge-tag is located above or below flavinium moiety (Figure S34). We have then performed variety of dihedral angle studies to estimate the amount of conformers with similar energies. The big amount of conformers which are close in the energy corresponds well with the broadened peak observed during IRPD analysis of this ion.

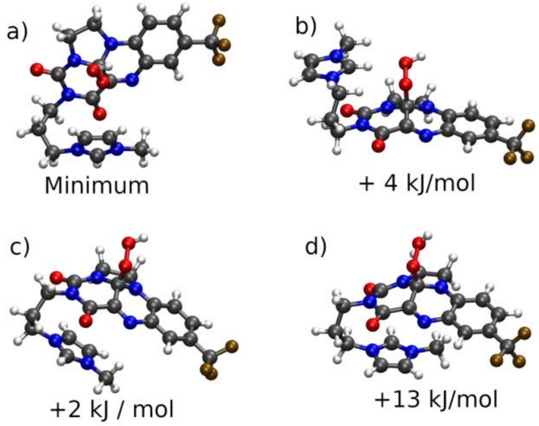

Figure S34. Sub selection of possible conformers of [**1b**+OOH]$^+$. Calculations were carried out without additional PC-3 basis set.

4.2.2.1 Dihedral angle studies of conformers of ([1b+OOH]$^+$)

To map potential energy surface of found minima we have performed studies of dihedral angles of the OOH group and the chain between imidazolium charge-tag and flavinium moiety (Figures S35 and S36).

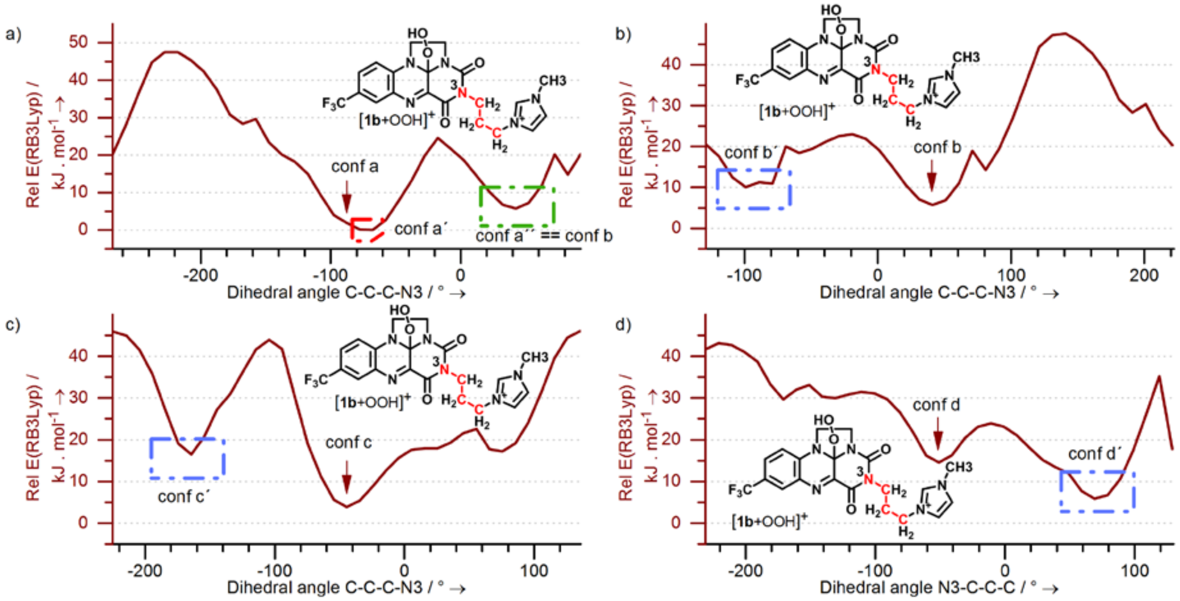

Figure S35. Dihedral angle study of charge-tagged C10a-hydroperoxyflavin ([**1b**+OOH]$^+$) – dihedral angle of the connection between imidazolium tag and flavin moiety. Dihedral study of conformer described in Figure a) S34a, b) S34b, c) S34c, d) S34d. Rotation was carried out by 10° steps and for each step structure optimization was carried out. Red arrow points to the start of the calculations. Minima in blue rectangles are caused by the interaction between the hydrogen atoms of imidazolium ring and the carbonyl atoms of flavinium moiety. Minimum in the green rectangle in (a) is virtually the same as the starting point of b). Calculations were carried out without additional PC-3 basis set.

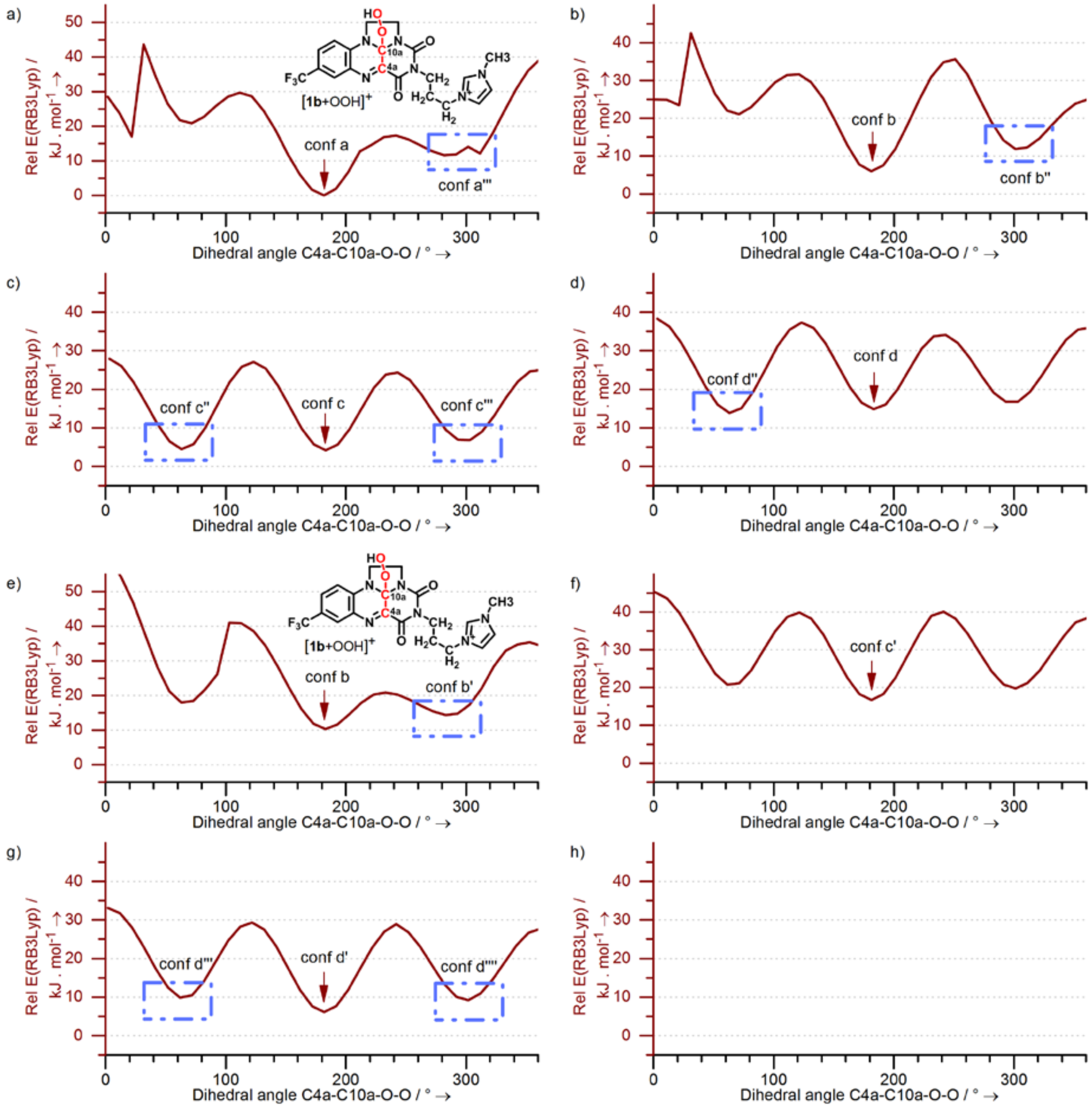

Figure S36. Dihedral angle study of charge-tagged C10a-hydroperoxyflavin ([**1b**+OOH]$^{+}$) – dihedral angle of the C4a-C10-O-O bond of structures described in Figures S34 and S35.

4.3 Proton affinity study of the 1•

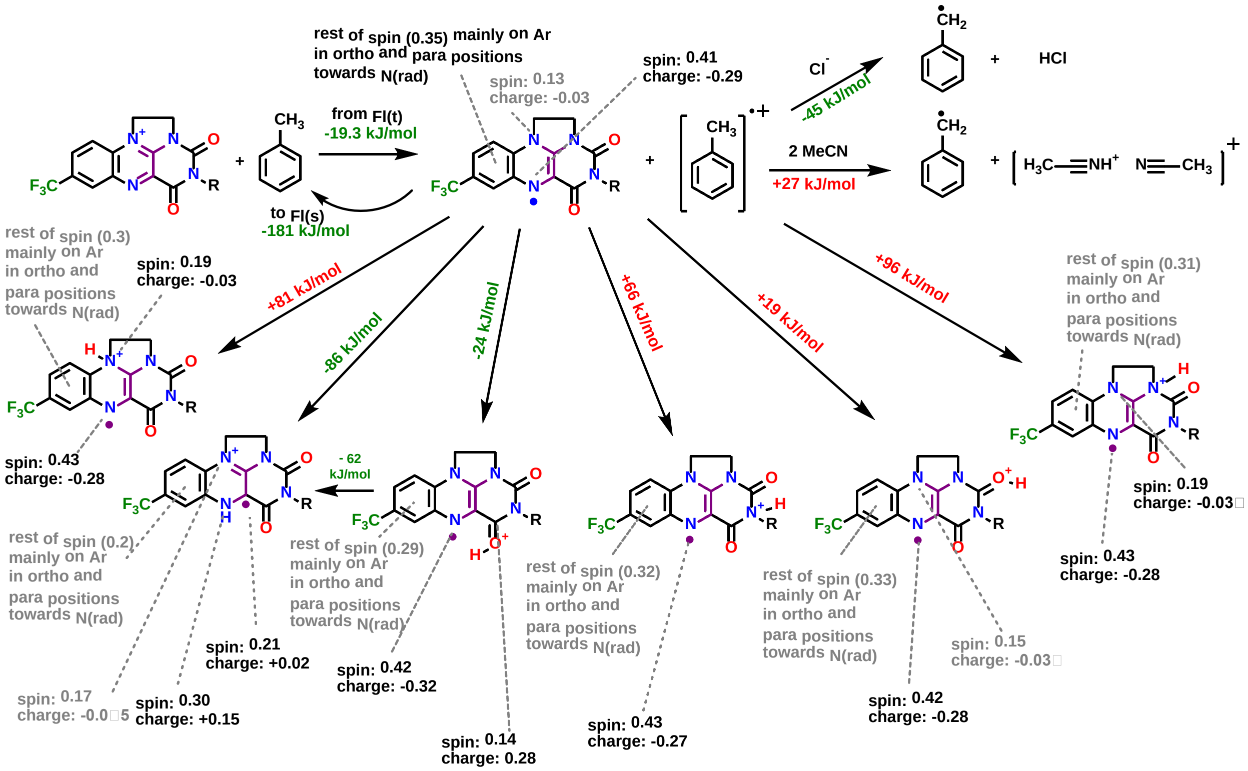

Figure S37. Structures and energies calculated with: B3Lyp/6-311+G** SCRF(SMD, solvent=Acetonitrile), empirical dispersion: gd3bj. Mulliken population calculated with: B3Lyp/cc-pvtz SCRF(SMD, solvent=Acetonitrile), empirical dispersion: gd3bj.

4.4 Comparison between the DFT calculated spectra and the experimental one

4.4.1 Comparison between observed [1a+2H,2O]$^+$ spectrum and calculated spectra of different structural isomers

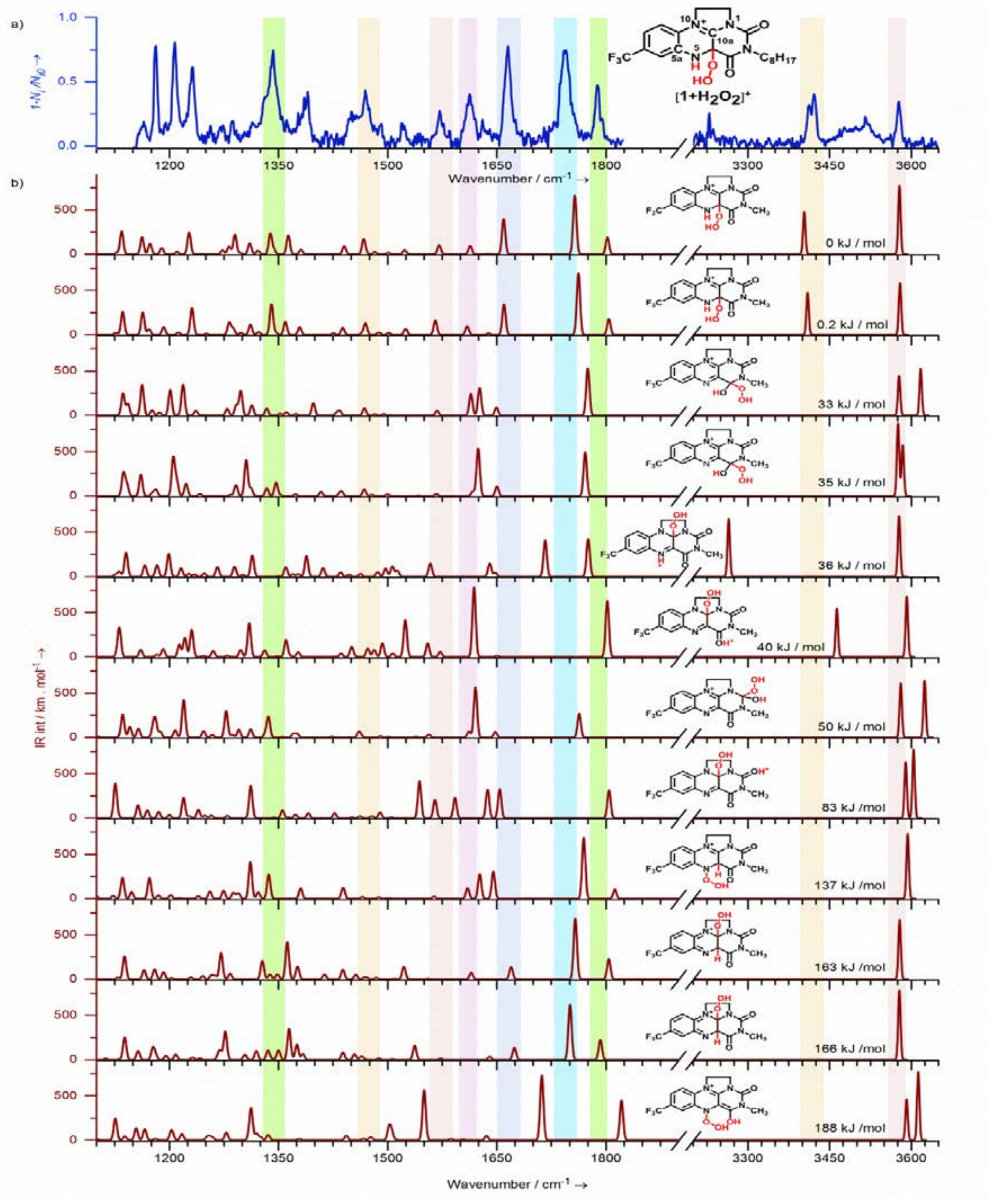

Figure S38. Comparison between experimental and DFT (PC-3 basis set applied for CF3 carbon atom, otherwise conditions were same as one described at the computational details section)

4.5 Other IRPD experiments

4.5.1 IRPD spectra of selected ions

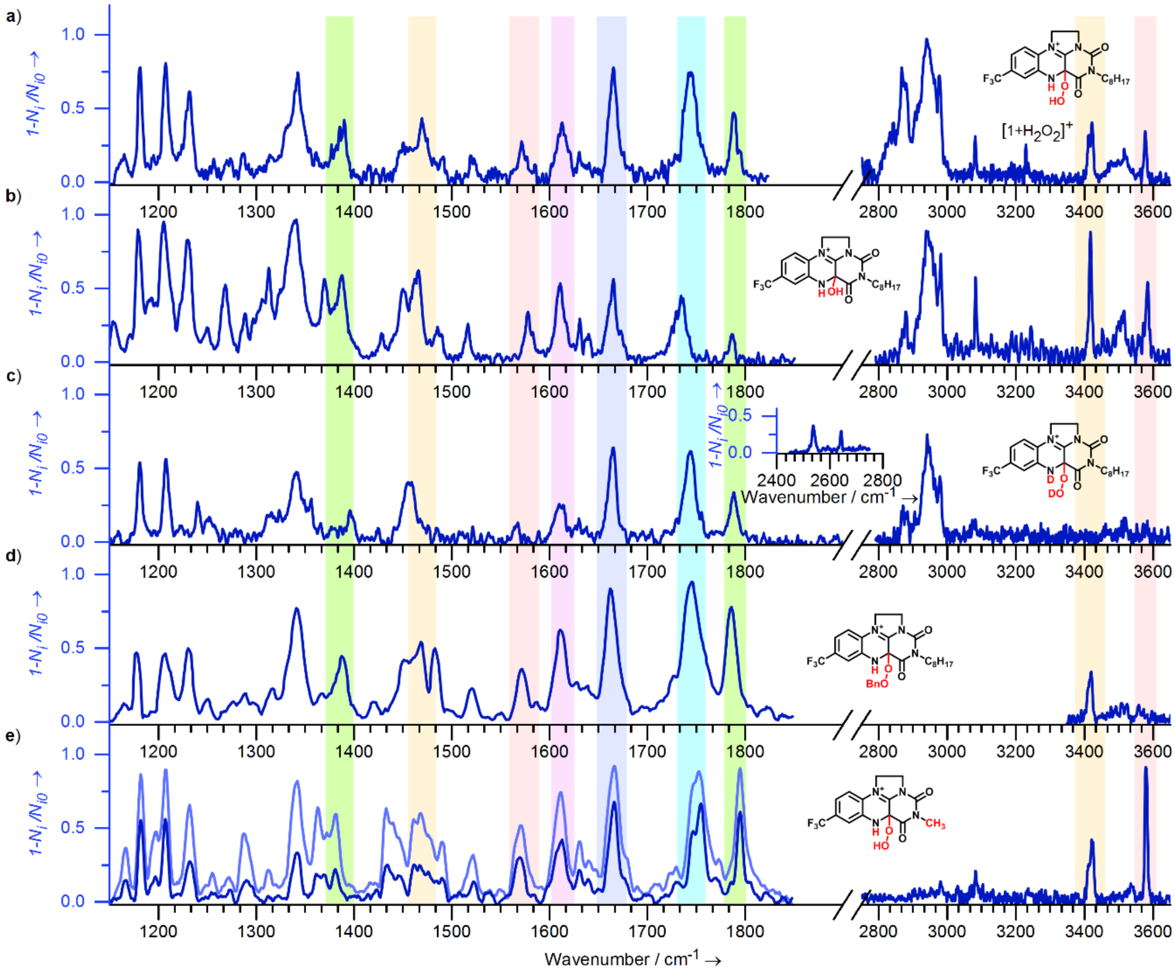

Figure S39. Comparison between experimental spectra of compounds which were assigned as C4a and N5 substituted **1**$^+$: a) [**1a**+2H,2O]$^+$, b) [**1a**+2H,O]$^+$, c) [**1a**+2D,2O]$^+$, d) [**1a**+Bn,H,2O]$^+$, e) analogue of compound used in a) where **1a** is methylated instead of octylated, notice the gain of the intensity of the O-H stretching vibration. Light blue shape in figure e) describes spectrum with full laser power.

4.5.2 Possible hydrogen bonding vs. non-linear effects

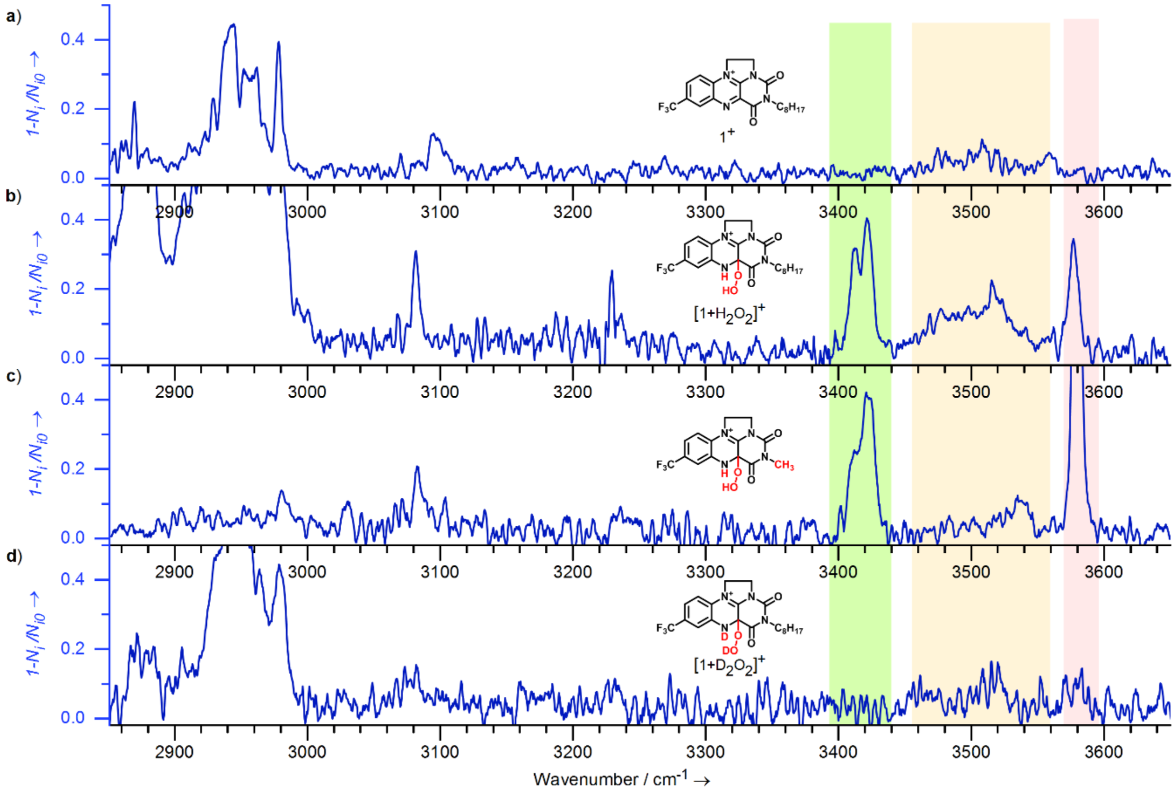

Figure S40. Comparison between upper range experimental spectra of compounds a) **1**$^+$: b) [**1a**+2H,2O]$^+$, c) analogue of compound used in b) where **1a** is methylated instead of octylated, d) deuterated analogue of b). Notice that the spectra a) and d) has been recorded at a lower attenuation compared to spectrum b), which is apparent from the attenuation of the C-H stretch bands present in range 2850-3000 cm^{-1}.

All spectra contain a signal between 3450 cm^{-1} and 3560 cm^{-1} that is slightly above the level of noise. For the ions [**1a**+2H,2O]$^+$ (b) and its methyl-analogue shown in (c), the band could be ascribed to hydrogen bonding of the hydroxyl group. However, it seems that the presence of the octyl group also affects the spectra in this range. Possible contribution of some combination bands reflecting vibrations of the octyl group is also partly supported by a weaker dependence of intensity of this band on the irradiation power (Figure S41).

4.5.3 Attenuation experiments

Attenuation experiments at given absorption bands can provide extinction coefficients and the estimate of the fraction of the ions that absorb at the given wavelength. Here, we have use it only as a qualitative measure, because the peaks are partly overlapping. Fundamental bands are characterized by exponential increase of the attenuation yield with the laser power. Overtones and combination bands have weaker power-dependence characteristics. See ref. *J. Phys. Chem. A* **2015**, *119*, 2532 or *Faraday Discuss.* 2019, doi: 10.1039/c8fd00196k. We have performed this analysis for [**1a**+2H,2O]$^+$ for the O-H stretching vibration (3577 cm^{-1}), the N-H stretches (3421 cm^{-1} and 3412 cm^{-1}) and the band between N-H and O-H stretching vibrations (3513 cm^{-1}).

Experimental details: Sample: 0.4 mg of **1a** was dissolved in 4 ml of acetonitrile. 300 µl of this concentrated solution was diluted by 2 ml of acetonitrile and 100 µl of 4-chlorobenzyl alcohol solution (1.4 mg dissolved in 4 ml of acetonitrile) was added. Conditions: “overpressure unit” applied, spray voltage 7 kV, capillary temperature 250°C, auxiliary gas 12, irradiated at the capillary tip. IRPD experiments: Helium was applied to the trap in two 0.25ms pulses separated by 25 ms. Quadrupole bender directed ions to the trap for 70 ms. Shutter has been opened with 40 ms delay from the sequence start and closed 930 ms from the sequence start. In case of 4 second cycles the shutter has been opened for 3930 ms.

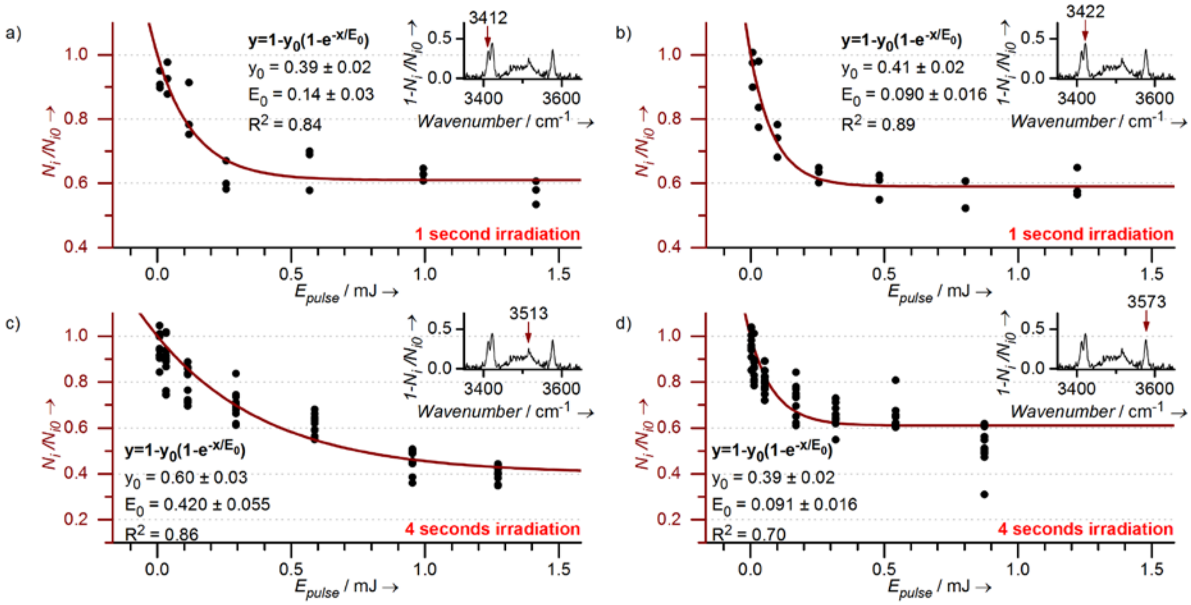

Figure S41 Attenuation curves of a) NH stretch vibration b) second NH stretch vibration c) probable non-linear effect d) OH stretch vibration.

4.5.4 2D IRPD experiments with [1a+2H,2O]$^{+}$

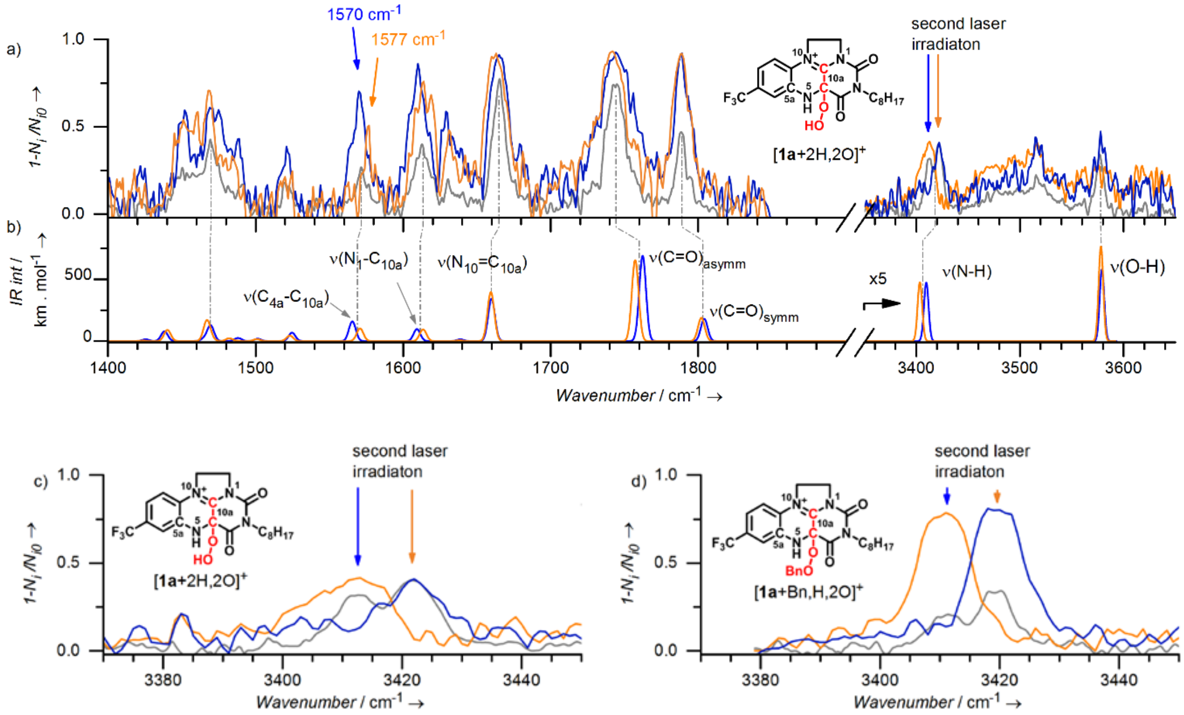

Figure S42 2D irradiation experiments. a) Experimental spectra. Grey full line describes irradiation of a mixture of two conformers (lower laser intensity). In orange: First OPO parked at 3422 cm^{-1} (depleting the ions absorbing at this wavelength), the second OPO scanned the orange spectrum. In blue: First OPO parket at 3412 cm^{-1} (depleting the ions absorbing at this wavelength), the second OPO scanned the blue spectrum. The detail can be seen in c). b) Theoretical spectra of two conformers. The theoretically predicted shifts of the N-H stretch and N_{4a}-C_{10a} stretch are perfectly consistent with the experimental results. d) Analogous experiment with [**1a**+Bn,H,2O]$^{+}$.

4.6 Comparison between VIS-PD spectra

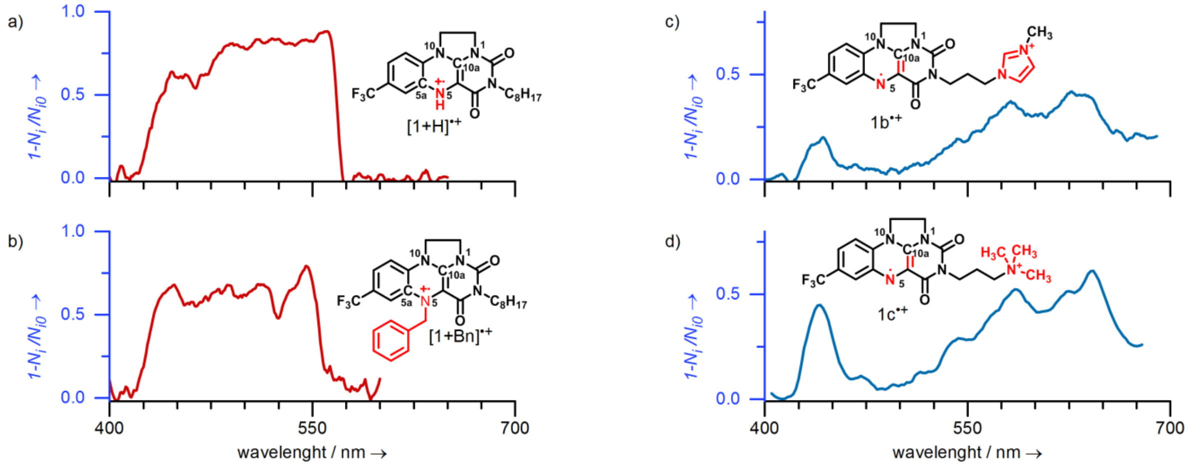

Figure S43. Comparison between VIS-PD spectra [**1a**+H]$^{•+}$ (a) and [**1a**+Bn]$^{•+}$ (b) radicals and comparison between VIS-PD spectra **1b**$^{•+}$ (c) and **1c**$^{•+}$ (c) radicals.

5 XYZ coordinates of the calculated structures

Optimized Structures
The format of individual records is following:

number_of_atoms
NAME charge multiplicity
electronic_energy(Hartree)
zero_point_energy(Hartree)
number_of_imaginary_frequencies
method/basis_set
atom1 x y z
...

37
1aH2O2_fig4b 1 1 -1360.3457474 0.2666805 0
B3LYP-D3/6-311+G** (with additional pc-3 on C of CF3 group)
C -0.0004729433 0.0011932053 -0.0002932103
C -0.0001538924 0.0008016502 1.3929757641
C 1.2374436644 0.0021442291 2.0682391397
C 2.44350734 -0.0202096224 1.3729002744
C 2.4272525755 -0.0264086264 -0.0127856723
C 1.2047772311 -0.0104657451 -0.689530871
N 1.1809367353 0.0784683032 3.4760974145
C 0.0434952494 -0.0337893021 4.1125604578
C -1.2123829524 -0.4574362651 3.4460858681
N -1.1845903952 0.0577109115 2.1347261568
N 0.1072396216 0.2609509489 5.40299685
C -1.0307183001 0.4594369791 6.2197913372
N -2.2624739293 0.3596813832 5.5653014792
C -2.456184863 0.0102779672 4.23322995
C 2.2563054293 0.4824804413 4.4254565468
C 1.5036847338 0.587096228 5.78736519
O -3.5257341327 0.0142112168 3.6802010239
O -1.2189606491 -1.9007867489 3.3453878062
O -1.2445838561 -2.4357607004 4.7032265193
O -0.9039574498 0.7495399319 7.3761993736
C -3.4486588635 0.6667276337 6.3969779335
C 1.2021379521 0.0292558603 -2.2013990607
F 0.0232689417 -0.3563788393 -2.7059835785
F 1.4432566157 1.2753018817 -2.6413261513
F 2.1498311403 -0.7676448381 -2.7099917657
H 3.3843049585 -0.0331498134 1.9080872462
H 3.3557814422 -0.0500086348 -0.5667468044
H -0.9384511305 -0.0013317363 -0.5405712528
H -4.3279037271 0.5336565994 5.7750182017
H -3.4737895062 -0.0107507311 7.2484770243
H -3.3886064117 1.6936674275 6.752998033
H 2.6813989838 1.4307539404 4.0974068733
H 3.0330592786 -0.2804601527 4.4410828129
H 1.85783063 -0.1369168675 6.5184906995
H 1.5404621299 1.5836312543 6.2234751852
H -1.9407701191 -3.1054026457 4.6136646439
H -2.0750977713 0.064405267 1.6512581795

37
1aH2O2_fig4b_shadow 1 1 -1360.3465242
0.2667601 0 B3LYP-D3/6-311+G** (with additional pc-3 on C of CF3 group)
C 0.0000417497 -0.0011574889 0.0007314433
C 0.0003963169 -0.0025091715 1.3958302959
C 1.2399131233 0.0000602112 2.0683525468
C 2.4454142796 -0.0229006322 1.3714475216
C 2.428211373 -0.029734396 -0.0136600381
C 1.2040700431 -0.0131051323 -0.6890504083
N 1.183918619 0.068371149 3.4730681852
C 0.0508843193 -0.03089345 4.1178935935
C -1.2362494494 -0.3820979843 3.476038582
N -1.1793825081 0.0467021443 2.1333448797
N 0.1426852714 0.2069267443 5.4230026714
C -0.9714608616 0.441357943 6.2614337356
N -2.2147763111 0.4328846733 5.6161333795
C -2.4177086313 0.2518323398 4.2543207928
C 2.2865158276 0.3803743847 4.4252819438
C 1.5546638787 0.4687025235 5.7984766677
O -3.445732573 0.4959138916 3.6812953056
O -1.4948456892 -1.7852147565 3.6939638535
O -0.3604549829 -2.5176591564 3.155254445
O -0.8135942495 0.6811209247 7.4255619396
C -3.3850142951 0.8091858283 6.442271179
C 1.2033483925 0.0246255015 -2.2007273204
F 0.0053060726 -0.2974676371 -2.7058375884
F 1.5137011498 1.2533732968 -2.6439089008
F 2.1059617657 -0.8255666354 -2.7068027821
H 3.3870437895 -0.0352085747 1.9050742367
H 3.3560711304 -0.0522991129 -0.5688184244
H -0.9381874339 0.0012291006 -0.5390328477
H -4.2733579373 0.4033031439 5.9674468867
H -3.2502565807 0.3908908628 7.4355265075
H -3.4653264588 1.8943229301 6.5072386508
H 2.7578188881 1.3173864949 4.1300732218
H 3.021346906 -0.4228907959 4.3995624098
H 1.8928523831 -0.2891047532 6.5023303757
H 1.6329484775 1.449528292 6.2639372531
H -0.809781945 -3.1283736656 2.5504499041
H -2.0681253635 0.1417524846 1.6572679391

53
1bOOH_fig4e 1 1 -1703.435313 0.4048386 0
B3LYP-D3/6-311+G**
C -0.5514464685 -0.3805784191 0.362802395
N -0.2886673491 -0.3311733913 1.6724033534
C 1.0358749069 -0.6831072099 1.8695457737
C 1.5617419562 -0.948488357 0.6442534185
N 0.5513419337 -0.7657885198 -0.2824579413
C -1.2314611986 0.1325532643 2.7081892493
C -0.7718068362 1.4014536305 3.4305383131
C -0.5162201078 2.626294421 2.5389150703
N 0.8594420938 2.6732651818 2.0093325461
C 1.069096382 2.4139260066 0.6547279694
C 2.4872556385 2.4551252865 0.2154174065
C 3.5316792013 2.4890513819 1.2847336639
N 3.123547881 3.1549952518 2.4713151198
C 1.8336284614 3.2100330194 2.9040080484
N 2.8055476562 2.4439159136 -1.0299555687
C 4.1253631474 2.6475036079 -1.3829987159
C 5.0791092961 3.1289687814 -0.4373563135
N 4.6921691013 3.1961261942 0.8732978153
C 6.338253834 3.5579325034 -0.8839867573
C 6.6695974984 3.4367512259 -2.2205664757
C 5.7531272958 2.908625958 -3.146501179
C 4.4885359874 2.5393602728 -2.7343331315
C 4.2265401645 3.8963568375 3.0915719981
C 5.2428471344 4.0496510569 1.9326610001
C 6.1736713128 2.7525134733 -4.5820625002
F 7.0753737471 1.7509552241 -4.7231819983
O 3.7924097451 1.0680622104 1.5062733408
O 4.7880188914 0.9413805001 2.5563574421
O 0.1512260007 2.1423911579 -0.0996139211
O 1.5088185036 3.6868736164 3.9694982558
C 0.7312259108 -0.7307126025 -1.7383118678
F 5.1328238047 2.4666528788 -5.3889049279
F 6.7603922538 3.8732593102 -5.0525738569
H 7.0550521873 3.9626418965 -0.1813056152
H 7.648867792 3.7528716468 -2.5580122286
H 3.7467909025 2.1907279466 -3.4399850595
H -0.6635477969 3.5292442386 3.1260854539
H -1.1877692521 2.6508652226 1.6829833177
H 5.3123266518 5.0797127874 1.5757815507
H 6.2361138869 3.7121958517 2.2343837762
H 4.6474224678 3.3302790929 3.921555047
H 3.8602466851 4.8524954285 3.4607725065
H 5.5542939497 0.6295082908 2.0518888711
H -1.567145513 1.6500205467 4.1385663445
H 0.1129987641 1.2017900225 4.0396356022
H -2.1823393679 0.3017637793 2.2015068611
H -1.3734916408 -0.6825088329 3.4198429361
H 1.4892968634 -0.7008379498 2.8443527266
H 2.5583026738 -1.229329504 0.3552820586
H -1.4835840943 -0.1094214495 -0.0998087804
H 1.4022095621 -1.5358828072 -2.0314018249
H -0.2340981923 -0.8729665422 -2.2196051057
H 1.1443905626 0.2404343602 -2.0141769843

53
1bOOH_fig4e_shadow 1 1 -1703.4338546
0.4048729 0 B3LYP-D3/6-311+G**
C -0.6752998651 1.0803310211 0.30738315
N -0.370993763 0.4690971842 1.5100121558
C 0.9451824326 0.2436237846 1.5561744547
N 1.4921202889 0.6931948797 0.4237237423
C 0.4939836167 1.2184585045 -0.3750407374
C -1.3354950504 0.0176051692 2.5338484216
C -2.3578329675 -0.9700087096 1.9740661744
C -1.7612745084 -2.1253153074 1.1562591043
N -0.5291113302 -2.6893570232 1.7375745929
C 0.6182344888 -2.7256715116 0.9111082293
N 1.7462522537 -3.2061640385 1.5087027414
C 1.8607521534 -3.3925251092 2.9136540168
C 0.5990341587 -3.8735114073 3.556843297
C -0.6694159215 -3.3641060796 2.9717699255
N 2.9399251247 -4.2908215851 3.0656217314
C 3.7133935932 -4.4635464833 1.8293634411
C 2.9774031688 -3.5676601604 0.8041923465
N 0.6107264121 -4.6820978926 4.5513141895
C 1.8139604057 -5.21541714 4.9693493152
C 2.9918389397 -5.0979693259 4.1795277021
C 4.1323969483 -5.8346938609 4.5234040588
C 4.1246181942 -6.6151967748 5.6660105356
C 2.9820011833 -6.6906616258 6.4788650489
C 1.8324672168 -6.0121627316 6.1243925044
O 2.0817382041 -2.1030509604 3.6006458501
O 3.2855948522 -1.4845816026 3.0535495085
C 3.0361421611 -7.5225481795 7.7320017159
F 3.511668715 -8.7622702904 7.4798613201
O 0.607713513 -2.342772554 -0.2452321072
O -1.7511271808 -3.4644546812 3.5004941314
C 2.9018845472 0.5448546999 0.0525576139
F 3.8619302335 -6.9691093907 8.6516212097
F 1.8276135926 -7.6574125331 8.30683795
H 5.022372846 -5.7805290087 3.9096939517
H 5.0121178985 -7.1733127321 5.9372292744
H 0.923702315 -6.0943384365 6.705065795
H -2.5032640351 -2.9200642802 1.073444282
H -1.4942715605 -1.8039459789 0.1532404736
H 3.7053985592 -5.5160951981 1.5368422025
H 4.7483110802 -4.1428350184 1.9603164008
H 3.5623099396 -2.6797496077 0.5624424948
H 2.7358118645 -4.0940449463 -0.1175267257
H 3.8817476833 -1.5378662688 3.8154210097
H -2.9154030226 -1.3602904977 2.8257835069
H -3.0769908054 -0.4489914976 1.3355913189
H -1.8299759558 0.8966184778 2.9488567752
H -0.7432014158 -0.4389648417 3.3262377253
H -1.6801867979 1.3583549281 0.0426109667
H 0.697955154 1.6336405295 -1.3466124919
H 1.4737004539 -0.2422209908 2.3602266886
H 3.3083564747 1.5145724363 -0.2314835601
H 3.4431192337 0.155713991 0.9121806908
H 2.9768204307 -0.1553611472 -0.7790961089

55
1aH_fig5b 1 2 -1484.6551368 0.4464309 1
B3LYP-D3/6-311+G** (with additional pc-3 on C of CF3 group)
C -0.2673389156 -1.5045200094 0.7552313769
C -0.1766886903 -1.009590776 2.1239712716
C 0.6320344867 0.0654597417 2.3887502923
N 1.3524746533 0.6916916413 1.4504725572
C 1.3463158171 0.2940404072 0.0988679612
N 0.5134120787 -0.7998030204 -0.1759873752
N 0.7941829265 0.6003668735 3.6113902517
C 1.7464081666 1.7353597598 3.549895445
C 2.1250226155 1.8072930544 2.0361455864
N -0.8541928173 -1.5652632087 3.1688987776
C -0.7273739319 -1.070673549 4.4600928101
C 0.1214886654 0.0477364433 4.7031062057
C 0.2551293938 0.5510355023 5.9947071362
C -0.4393782229 -0.0398446525 7.0397646385
C -1.2735676273 -1.1382889417 6.8017920887
C -1.419898887 -1.6524241893 5.5244147791
C -1.9863718702 -1.7892346706 7.9664588688
F -1.1421318791 -2.5672835019 8.6607762066
O 2.0194929125 0.8746252949 -0.711455001
C 0.4200539304 -1.2422228719 -1.5950447918
O -0.9673815148 -2.4547560892 0.4667107908
F -2.4729023435 -0.8689639882 8.8072276043
F -3.0030421571 -2.5562799741 7.5554392902
H 0.8974509038 1.4016159854 6.1813169114
H -0.3393278274 0.3540871989 8.0425892284
H -2.0707119368 -2.4990420128 5.3448594564
H 2.6098302907 1.5252891341 4.1821835713
H 1.2581656113 2.6464257472 3.8969862466
H 1.8249930096 2.7471327326 1.5752633574
H 3.1888362155 1.6529133113 1.8634436643
H -1.4509601588 -2.3628300827 2.9668649647
C -0.8413640112 -0.7228257402 -2.285489335
H -0.8105025873 -1.101589317 -3.3124747039
H -1.7226054883 -1.1726070964 -1.8194939134
H 1.3193733965 -0.8772101478 -2.0855933059
H 0.4350328299 -2.3310569476 -1.5806581375
C -0.9701315518 0.8017610467 -2.304065702
C -2.197697586 1.2832047167 -3.0803321729
H -1.0348046119 1.1808909623 -1.2750725702
H -0.064923586 1.2428002947 -2.7349816636
C -2.351401274 2.8054637256 -3.0782888512
H -2.1309985509 0.9266546451 -4.1149679196
H -3.1006431086 0.8248713209 -2.6581918889
C -3.5653522424 3.2952228415 -3.8702250672
H -2.4282871103 3.160873156 -2.0420276536
H -1.4421531892 3.2609609304 -3.4898899287
C -3.7197250602 4.8180993962 -3.8669678276
H -3.4877854843 2.9424082695 -4.9060117729
H -4.4754903941 2.8384665952 -3.4612282352
C -4.9335997787 5.2980998253 -4.6643325347
H -3.7982571832 5.1700593676 -2.8310112646
H -2.809171505 5.2723000099 -4.2752391206
H -5.0197902769 6.3872789429 -4.6444266729
H -4.8637382151 4.9891780013 -5.7114664635
H -5.8612684357 4.8831057779 -4.2585345142

50
1b_fig5d 1 2 -1552.431113 0.3849142 1 B3LYP-D3/6-311+G** (with additional pc-3 on C of CF3 group)
C 0.1064134619 -0.1174384608 -0.014141502
N 0.1110558087 -0.0160963449 1.3675723532
C 1.3689853935 -0.1542472219 1.8031691583
N 2.1703973878 -0.3412697813 0.7501979318
C 1.3978140786 -0.3233935044 -0.3964433158
C -1.0482631356 0.2601880782 2.2619928656
C -2.3874309831 0.1286035673 1.5547641699
C -2.7222867096 -1.3042560586 1.1231424735
N -3.8963019742 -1.3329017538 0.2519647614
C -3.6748592499 -1.0296417673 -1.0812065557
N -4.7954039777 -1.1252362771 -1.8719141228
C -6.0261368785 -1.3887563377 -1.3449106049
C -6.3057018587 -1.6359447014 -0.0230500667
C -5.1733770183 -1.5867895818 0.8957860371
N -6.9686700803 -1.359112184 -2.3106910412
C -6.3712756945 -1.1455895188 -3.635362468
C -4.8947165298 -0.7891942456 -3.3037904875
N -7.5755490183 -1.8890893503 0.3929358397
C -8.5372547595 -1.9068129165 -0.5766163513
C -8.2912333667 -1.6547707344 -1.9677898401
C -9.3119532986 -1.695901842 -2.9086494915
C -10.6105279961 -1.9844794722 -2.500934426
C -10.8785335692 -2.230208282 -1.1493739408
C -9.8671653989 -2.1934497135 -0.204809338
C -12.2848548542 -2.5844541889 -0.7424418268
F -13.1907492048 -1.8268210555 -1.3866890729
O -2.5807427641 -0.7065053069 -1.5349433485
O -5.2060803532 -1.7327515208 2.096367883
C 3.6238518737 -0.5389068025 0.8003490642
F -12.5699974742 -3.8681006299 -1.0401063438
F -12.489540731 -2.4281151715 0.5721579765
H -9.1033656904 -1.5036392445 -3.9537766692
H -11.4112708098 -2.0132731344 -3.227728329
H -10.0641364132 -2.3805886234 0.8418898122
H -2.9619367365 -1.911366695 1.9942013469
H -1.8994964273 -1.7694271238 0.5817882765
H -6.8785928053 -0.3357507806 -4.1606182279
H -6.4504183131 -2.0581431018 -4.2321777636
H -4.1812519086 -1.374301935 -3.880560224
H -4.6827674754 0.2707118077 -3.4505111225
H -2.4203922853 0.8012247078 0.6945580012
H -3.1543652635 0.4730591455 2.2522613836
H -0.9228311733 1.2734862571 2.6463676578
H -0.977176101 -0.4380811145 3.0983507849
H -0.800355521 -0.0878999826 -0.6025048166
H 1.8243415172 -0.4668731417 -1.3740765585
H 1.6852630646 -0.1168116881 2.8320646358
H 4.1175896379 0.2598581711 0.248141921
H 3.9484228669 -0.5164452248 1.8384546768
H 3.8734100206 -1.5049482421 0.3631665002

33
Fl+_fig6 1 1 -1208.8198988 0.2364186 0 B3LYP-D3/6-311+G**
C -0.8899289537 -0.4142344036 -0.0271707145
N 0.129959959 -1.3145255659 -0.0261418539
C -0.6713509207 1.0027949282 -0.0223363422
C -1.7457836999 1.899548233 -0.0159414972
N 0.635664348 1.4133681265 -0.019747482
C -3.0274478729 1.3963164988 -0.0135102323
H -1.5684174262 2.9662426291 -0.012172308
C -3.2610574832 0.0016230001 -0.0209813057
H -3.8657902015 2.0818049985 -0.0062164612
C -2.2186435831 -0.8928261911 -0.026883062
C -4.6835544153 -0.4853291921 0.0090135985
H -2.3769599965 -1.9627777437 -0.029217496
C 1.3570198571 -0.8675781357 -0.0224415233
C 1.6133638668 0.5180657015 -0.0205779173
C 2.5514167185 -1.7546680931 -0.0085364274
N 2.8275292048 1.066920699 -0.0077123854
C 3.9901982667 0.3013224655 0.0189309925
N 3.7842280251 -1.0862407892 -0.0034075851
O 5.0802265645 0.8245055713 0.0567151841
O 2.4816294195 -2.964469554 -0.0006610278
C 4.9796969088 -1.9454732067 0.012946356
H 4.9075150669 -2.671994725 -0.7943674822
H 5.8544297728 -1.3195330663 -0.1255994837
H 5.0444875026 -2.467977935 0.9678965524
F -4.7870602744 -1.8113149124 -0.2057920937
F -5.2731421427 -0.2337895616 1.2062002364
F -5.4468473296 0.1292274851 -0.9268391053
C 1.1999354573 2.7936838886 -0.0061900017
C 2.7333574606 2.5486674091 0.0128920465
H 0.8437209273 3.3077148137 0.8839458466
H 0.867279486 3.31454669 -0.9015750008
H 3.2250204967 2.9578108616 -0.8662402947
H 3.1993969905 2.930702076 0.9178892705

33
FI+*t_fig6 1 3 -1208.738158 0.2327195 0 B3LYP-D3/6-311+G**
C -0.0009535001 -0.0079272129 0.0023981085
C 0.0003993252 -0.004452234 1.4695784532
C 1.2377306718 0.0024847409 2.0612697171
N 2.4171435937 0.0029718578 1.4395792814
C 2.5092037534 -0.0114391174 0.038228358
N 1.2830260126 0.0080126035 -0.6074310057
N 1.4247288154 0.001877082 3.4357985826
C 2.8643975403 0.0086482482 3.7624014608
C 3.5521980841 -0.0102191056 2.3798434871
N -1.13035522 -0.0149540196 2.2071269427
C -0.961102708 -0.0135203066 3.5721035515
C 0.3322561252 -0.0029055449 4.2290072332
C 0.4463920658 0.0017925518 5.6409659901
C -0.6991643665 -0.0023782386 6.4345092376
C -1.9363550835 -0.0128324493 5.8170508232
C -2.071857051 -0.0217862484 4.3963463362
C -3.2003232674 -0.0550810441 6.6400664763
F -3.7776535022 -1.2788717271 6.576560475
O 3.59372088 -0.0374897956 -0.5051485844
C 1.2959715508 0.0086979894 -2.0781718448
O -1.0042379679 -0.0222115368 -0.6842245404
F -2.9834215541 0.2150593683 7.938894587
F -4.1140415482 0.832884792 6.1912878486
H 1.427844344 0.0075300257 6.0966959346
H -0.6072714907 0.0022058561 7.5110414162
H -3.0544178889 -0.0326023955 3.9424375095
H 0.6419293104 0.7995255248 -2.4403994417
H 2.3130886888 0.1828346352 -2.4131117164
H 0.9426061476 -0.9529908839 -2.452181084
H 3.102011251 -0.8731319336 4.3571216017
H 3.097801111 0.9094566629 4.3300867803
H 4.1729529299 0.8687428469 2.2136242078
H 4.142456769 -0.911940009 2.2231284749

33
Flrad_fig6 0 2 -1208.9765085 0.2338109 0 B3LYP-D3/6-311+G**
C 0.0011407897 -0.0035022642 0.0009137993
C 0.0009502309 -0.0028406742 1.4108720448
C 1.2805965669 -0.0009690256 2.0624132596
C 2.4686085009 -0.0001275297 1.3371185654
C 2.4290459066 -0.0012454586 -0.0522452528
C 1.1922497415 -0.0053744022 -0.7097946083
N 1.2625210768 -0.0001070703 3.449435552
C 0.0787167222 -0.0039717907 4.0875374512
C -1.1346985687 -0.0042146153 3.434585254
N -1.1958439434 -0.0034501908 2.0731853892
N 0.2472289234 -0.0007627864 5.4276776253
C -0.8181898145 0.0153935086 6.3041872063
N -2.066278702 -0.0001165542 5.6841396168
C -2.3167562823 0.0034584146 4.2839050395
C 2.3876289092 0.0010958766 4.4033223624
C 1.6815157007 0.0244431665 5.7859991372
O -3.4715620928 0.0129314572 3.8770179222
O -0.6498434139 0.0397687872 7.5135825702
C -3.2521386113 0.0074116781 6.5481836557
C 1.175786487 0.0320712328 -2.2068502311
F -0.039612163 -0.2342281923 -2.7340818221
F 1.5443077328 1.2511294846 -2.6965351448
F 2.0418638954 -0.8596612501 -2.7567407954
H 3.4188850722 0.000331854 1.8552412169
H 3.3524416533 -0.0001973009 -0.617091063
H -0.9548393442 -0.0039026629 -0.5055064886
H -3.9112020437 -0.8138966665 6.270185629
H -2.926479624 -0.1090118995 7.5767077521
H -3.7917333474 0.9491721902 6.4372069961
H 3.0071422135 0.8830187942 4.242710463
H 2.9894608478 -0.8967300109 4.2633851136
H 1.9284958837 -0.8481912665 6.3872038562
H 1.9058322264 0.9305971117 6.3455831173

34
FlHrad+_fig6 1 2 -1209.43418 0.2476744 0 B3LYP-D3/6-311+G**
C 0.0016966829 0.000708429 0.001515353
C 0.0006624972 0.0005696997 1.45015804
C 1.2064249486 0.0004960421 2.1121538576
N 2.3952458713 0.0012723605 1.4903892679
C 2.5028912707 0.0009989388 0.1077724983
N 1.2843749482 0.0017013273 -0.5792333562
N 1.3268162188 -0.0028459049 3.4455825953
C 2.7561739502 -0.0050535472 3.8391088341
C 3.5022333431 -0.0017388212 2.4759080788
N -1.1308535351 -0.0014869659 2.210734983
C -1.0729380696 -0.0062849877 3.5917537293
C 0.1917635407 -0.0076777474 4.2443242147
C 0.2602215453 -0.0140586719 5.6364954629
C -0.9084866633 -0.018641772 6.3795238106
C -2.1534200394 -0.0143075277 5.7347057577
C -2.2445434032 -0.0102121608 4.3529637338
C -3.4000980515 -0.0508233875 6.571601816
F -3.576668041 -1.2626372549 7.1625516996
O 3.5799720875 0.0000596813 -0.4523994126
C 1.3843770952 0.0027080328 -2.0456516178
O -1.0276412215 0.0000008581 -0.6582638373
F -3.3634062023 0.8564555647 7.578100436
F -4.5185912386 0.1975441026 5.8594595405
H 1.2234449522 -0.0150240109 6.1282978562
H -0.8539461371 -0.0245553585 7.4605563363
H -3.2008789307 -0.0098014907 3.8455643009
H 1.9191626965 0.8918788736 -2.3783157948
H 1.9146811073 -0.8885038181 -2.3801538136
H 0.3767311062 0.0055686721 -2.4484134085
H 2.9664935025 -0.8984353926 4.424933836
H 2.9678463209 0.8847096364 4.4299313364
H 4.1115306424 0.8896305343 2.3449441636
H 4.1108678672 -0.892826019 2.3401114642
H -2.0360596903 -0.0011445086 1.746526236

37
FlHOOH+_fig6 1 1 -1360.43481 0.266572 0 B3LYP-D3/6-311+G**
C -0.0023715373 -0.0191108998 0.0093487814
C -0.0003040611 -0.0023504533 1.4047005177
C 1.2370880557 0.0087574473 2.0804769528
C 2.4441911558 -0.0187491778 1.388116577
C 2.4313833721 -0.0445983664 0.0027966025
C 1.2065241724 -0.0420224584 -0.6735281483
N 1.1807562227 0.0854077718 3.4793388183
C 0.0479240412 0.007205915 4.1198482715
C -1.2400480148 -0.3426780917 3.4800577876
N -1.1842377165 0.0430519246 2.1295493067
N 0.1442406124 0.246546256 5.4251135095
C -0.9572170367 0.4607148473 6.255745835
N -2.2097973106 0.4394396957 5.6222523322
C -2.4188692206 0.2795623177 4.2643673012
C 2.2901183027 0.3917214862 4.4275190925
C 1.5667526601 0.4748957889 5.798167703
O -3.4627015082 0.5186152054 3.7063269815
O -1.5112844662 -1.7614108662 3.7124210868
O -0.4342051556 -2.5278546372 3.1277131692
O -0.8148721988 0.6893355218 7.4316955448
C -3.3744519506 0.7856223412 6.4576851488
C 1.2093258352 -0.0195466971 -2.1753598881
F 0.0248712779 -0.3888212627 -2.7097871884
F 1.4801659694 1.2223345294 -2.6628427263
F 2.1498181384 -0.8414339485 -2.6996159198
H 3.3805789021 -0.0190600233 1.9299765057
H 3.3635737785 -0.0680898392 -0.5455237626
H -0.948071115 -0.021536906 -0.5171810174
H -4.2623191575 0.3700945913 5.9910813074
H -3.2340834447 0.3508021299 7.4442050061
H -3.4709916467 1.8689838187 6.5410676004
H 2.7508551882 1.3318265717 4.1313578629
H 3.0184856999 -0.4151029955 4.3895867032
H 1.888609121 -0.3058630467 6.4828122852
H 1.6670793007 1.4521070725 6.2635772245
H -0.8352438278 -2.8265736091 2.2909206566
H -2.0597710817 0.0646361419 1.6184168293

50
FlHOOBn+_fig6 1 1 -1630.9025878 0.375595 0 B3LYP-D3/6-311+G**
C -3.0780368393 1.0365018267 -0.0185663787
C -1.7274494199 0.7922237741 -0.2661329182
C -1.355556631 -0.4587319444 -0.8019626752
C -2.2967276945 -1.4476591 -1.0680065398
C -3.6362334358 -1.1960388553 -0.8135652075
C -4.0137952052 0.0474703649 -0.2976456545
N -0.7581709826 1.7631074149 -0.0501141436
C 0.5923866789 1.4024374524 0.1651583409
C 0.8936379119 0.2534108925 -0.7241351108
N 0.0049855243 -0.6276096233 -1.0941619791
N 2.1077615192 0.0374349278 -1.2261345136
C 3.1365309671 0.9787832208 -1.1951856639
N 2.8285434018 2.1975216946 -0.5729988776
C 1.5803107288 2.5628688264 -0.1018749359
C 2.0974860425 -1.1770345684 -2.0856114394
C 0.6322418477 -1.6650416542 -1.9608364747
O 0.9367956791 1.0909401624 1.52949154
O 0.0753678595 -0.0112851555 1.9582120064
C 0.895743941 -0.8907553933 2.7576545579
C 1.8763557515 -1.6952444426 1.9537678307
C 1.4630178371 -2.872574982 1.3216861324
C 2.3658642497 -3.6290921744 0.5786391132
C 3.6902766005 -3.2080459321 0.4527797822
C 4.1067584792 -2.0284487042 1.0693645011
C 3.2032771764 -1.277204039 1.8189826243
O 1.2717674662 3.6965733282 0.1775862838
C 3.8752246621 3.235688829 -0.6054142361
O 4.2046295391 0.7494715204 -1.7080775153
C -5.4587917358 0.3007935474 0.0231239346
F -5.7807061375 -0.1078019921 1.2820848498
F -5.7874809134 1.6112995662 -0.0435585631
F -6.2967546818 -0.3560364649 -0.8114546319
H -1.9839076303 -2.4003837194 -1.4742900793
H -4.3767861073 -1.9570362203 -1.0175409179
H -3.3764443201 1.9941277206 0.3881634071
H 3.705695665 3.915651472 0.2246900977
H 4.8422671768 2.7528268916 -0.5013235882
H 3.8344668167 3.7845723101 -1.547209123
H 0.1082705661 -1.6873294052 -2.9139325667
H 0.557718705 -2.6289672845 -1.4631635425
H 2.8068936291 -1.9007734586 -1.6970789897
H 2.358137946 -0.8945871024 -3.1030250209
H -1.0553969884 2.6497981497 0.3420778966
H 1.3859320043 -0.3005931529 3.5348334187
H 0.1409964328 -1.5295426982 3.2232137994
H 3.5288710881 -0.3653547316 2.3070854622
H 5.1334676772 -1.6957142076 0.9686600577
H 4.3943346527 -3.7965951406 -0.1244867948
H 2.0392047323 -4.5452435464 0.0999776154
H 0.4333331531 -3.1989257733 1.4230319111

50
FlPhMeOOH+_fig6 1 1 -1630.8907997 0.3731003 0 B3LYP-D3/6-311+G**
C 0.0421421729 -0.095174234 0.0203553665
C 0.0472811375 -0.0930919754 1.4162144407
C 1.2957123023 -0.0659583124 2.0738591681
C 2.4971262596 -0.0748272909 1.3715883303
C 2.4764505904 -0.09489173 -0.0135644294
C 1.2463216401 -0.0939707113 -0.6746458286
N -1.1461223649 -0.0727708424 2.1604468266
C -1.1084992256 -0.5602158379 3.4991146472
C 0.1423022306 -0.0507983031 4.1254320607
N 1.2636398526 0.0599172154 3.4668395748
N 0.2318197374 0.3370360642 5.395363099
C -0.8725256683 0.6046641685 6.203759952
N -2.1171429348 0.371740532 5.6112718351
C -2.3423527401 -0.2250610175 4.3798847724
C 1.6237572204 0.7595679621 5.709348036
C 2.3553943794 0.5364169162 4.3620635567
C -2.3918877793 0.1748583468 1.4749970663
C -3.1228376661 -0.8765492357 0.9281323451
C -4.2981356812 -0.6017346535 0.2400492119
C -4.7555870863 0.7146461619 0.0843972742
C -3.9973868475 1.7522661697 0.6351732081
C -2.8167855736 1.4886475256 1.3266859438
C -6.0381767271 0.9953536442 -0.6519456683
O -1.0333860345 -2.0106488408 3.3930918666
O -1.0257466721 -2.5690231687 4.7361035989
O -3.4488028259 -0.5451662338 4.0180604647
C -3.3117829193 0.6422930743 6.433888343
O -0.7296421239 1.0332642398 7.3233650866
C 1.2111062575 -0.1641209742 -2.1750852111
F 2.3206187408 0.3575259112 -2.7445200611
F 1.1202111515 -1.4472182549 -2.6221788121
F 0.1530426635 0.493944586 -2.7030226493
H 3.4362798272 -0.0561601246 1.9083110458
H 3.4045887742 -0.1015775735 -0.5680498391
H -0.8977893004 -0.0998696235 -0.5116827374
H -3.6636929519 -0.2817348267 6.8948870629
H -3.0443646992 1.3603932187 7.2018002542
H -4.0871882211 1.051435837 5.7918964052
H 2.7742055832 1.4506346105 3.946705043
H 3.1199083166 -0.2350540455 4.425786653
H 2.0195191862 0.1326664796 6.5041357116
H 1.6214485004 1.8024053042 6.0170071856
H -2.2276765206 2.2938917164 1.7496037261
H -4.3309684029 2.7776087816 0.5208380043
H -4.8697873213 -1.419377498 -0.1856680664
H -2.7714197526 -1.8944473613 1.0411319577
H -6.167512158 2.0648486477 -0.827266632
H -6.0588001872 0.4819109943 -1.6172243001
H -6.9005117527 0.6398388107 -0.0783043228
H -1.8895849072 -3.0204156249 4.756412072

15
Tol_fig6 0 1 -271.6721416 0.1272324 0 B3LYP-D3/6-311+G**
C -0.0001102167 -0.0051789843 0.0002504651
C 0.0045173651 0.0005454303 1.3941333434
C 1.2135734595 0.0073878 2.0916090763
C 2.4370850483 0.0070269812 1.4127444933
C 2.4182684782 0.0039626192 0.0115376073
C 1.2138875847 -0.0027892556 -0.6892543194
C 3.7432186419 -0.008336281 2.1647098959
H 3.5960993142 0.2363103497 3.2184497459
H 4.4543187676 0.7065105957 1.7411273363
H 4.2109543656 -0.9975049748 2.1137120121
H 3.3573154566 0.0079283552 -0.5329827453
H 1.2218105948 -0.0039825636 -1.7738417621
H -0.9378301957 -0.0083738464 -0.544055199
H -0.9321842909 0.0023202944 1.9409231194
H 1.2083394727 0.0146748277 3.1767883344

15
Tolrad+_fig6 1 2 -271.4388025 0.1251655 0 B3LYP-D3/6-311+G**
C 0.0026824439 0.0114088917 0.0022153645
C -0.001497318 -0.0004810866 1.3693396653
C 1.2338678371 -0.0186138166 2.0890577011
C 2.4765247845 -0.0259984967 1.358788368
C 2.4752245144 -0.0142166846 -0.0078114409
C 1.2406809632 0.0047138327 -0.7011143177
C 1.2604675677 -0.0347994868 3.5585963711
H 0.2670345622 0.0097178541 3.9997506527
H 1.8800604753 0.7947639361 3.9274768111
H 1.7818137437 -0.9422640801 3.8998921234
H 3.4030489081 -0.0408976746 1.9195638505
H 3.4020640855 -0.0191853815 -0.5662970002
H 1.2351210691 0.0138623889 -1.7845878776
H -0.9236355274 0.0259903486 -0.5574214478
H -0.9284473867 0.0040680403 1.9284705857

14
Bnrad_fig6 0 2 -271.0192828 0.1140377 0 B3LYP-D3/6-311+G**
C 0.01611329 -0.0973146332 0.019207695
C -0.0072373537 -0.1541632058 1.4441491035
C 1.245375127 -0.0992628383 2.1239825933
C 2.43369011 0.0055869295 1.4216299632
C 2.4297731105 0.0601160266 0.0210441779
C 1.2117706223 0.0075225635 -0.6705730962
C -1.2168013461 -0.2605273519 2.1504796789
H -2.1645202292 -0.301245492 1.626632179
H -1.2261567532 -0.3029449096 3.2332381398
H 1.2533244081 -0.140997327 3.2078373363
H 3.374650632 0.0459808926 1.9593311579
H 3.3629847136 0.1422409656 -0.5239046346
H 1.2057838111 0.0494034971 -1.7542570175
H -0.9240326066 -0.1375422463 -0.5202854448

17
BnOOH_fig6 0 1 -422.0746492 0.1358319 0 B3LYP-D3/6-311+G**
C -0.0296169672 -0.1952703841 0.008099541
C -0.0015360753 -0.0240547643 1.3910373297
C 1.2196774623 0.1566516588 2.043984516
C 2.406157052 0.162956374 1.3151434747
C 2.3865963573 -0.006723597 -0.0745265885
C 1.160524415 -0.1835560436 -0.7201059129
C 3.6703209816 0.0266465463 -0.8613511087
O 4.2982559647 1.3219427545 -0.8647649451
O 3.4709885293 2.2300756007 -1.6375999945
H 4.4381197758 -0.6003322828 -0.3988138038
H 3.5156530088 -0.3038818167 -1.891464668
H 1.1394467603 -0.313570365 -1.7968688324
H -0.9750463112 -0.3354746034 -0.503764128
H -0.9248017627 -0.03312348 1.9593604165
H 1.2454667379 0.2853748857 3.1202745371
H 3.3543337152 0.2969024973 1.825331388
H 2.9019839153 2.6196169294 -0.9512867458

16
BnOOrad_fig6 0 2 -421.4328034 0.1240059 0 B3LYP-D3/6-311+G**
C 0.0037711384 -0.0268925685 -0.0305874859
C -0.0234805935 -0.0209884899 1.3620909343
C 1.1698735798 0.0044232795 2.0919845732
C 2.391107976 0.0266131935 1.410373436
C 2.4193141699 0.0200303045 0.0172879379
C 1.2256305875 -0.0060101621 -0.7041875126
C 1.137287129 0.0406996715 3.584906905
O 1.0639481638 1.4716203314 3.9933661925
O 0.9993536247 1.6115114619 5.2980619714
H 0.2527723063 -0.4401806097 4.0015917312
H 2.0425341956 -0.361004563 4.0396222843
H 3.3176916023 0.0435381991 1.9740351401
H 3.3695893563 0.0326404634 -0.50414871
H 1.2472484295 -0.0140396698 -1.7880946757
H -0.9246909054 -0.0510093318 -0.589571209
H -0.9718348526 -0.0413250291 1.8883189257

47
FlPhMerad_fig6 1 2 -1479.892112 0.3546462 0 B3LYP-D3/6-311+G**
C 0.0855259151 0.1626623865 -0.0238860195
N 0.0836045193 0.3641323001 1.4430749598
C 1.2959445155 0.13904861 1.9639901724
N 2.1836464351 -0.2047709972 1.0147033129
C 1.5571240641 -0.217588498 -0.3262667463
C 1.5983057597 0.2403027929 3.3079459215
N 0.5759598282 0.6002021177 4.1642579498
C -0.7099110159 0.8355450451 3.6587732457
C -0.9698264463 0.716167058 2.2663574662
C -2.2467133916 0.9508208751 1.7567755753
C -3.273664488 1.3036735986 2.6141421157
C -3.0221300462 1.4249859234 3.9856181579
C -1.7605144563 1.1946319461 4.5103554507
C 3.5084191128 -0.4933112176 1.2923774658

N 3.8410512213 -0.3970558541 2.6420663899
C 2.9726226694 -0.0621727358 3.7047892519
C 0.8064819685 0.7644422176 5.5862640871
C 1.1845705728 2.0099808671 6.0684748582
C 1.3786849421 2.1699198702 7.4368672927
C 1.1968470783 1.1009433045 8.3227495297
C 0.8001222193 -0.1372513295 7.8028921957
C 0.6010558746 -0.3127287507 6.4371574816
C 1.4479072996 1.2730670458 9.7968695659
C -4.1588407596 1.7606366418 4.907696317
F -3.7436943975 2.2370208481 6.1008926361
O 4.2870046573 -0.8004593516 0.4110914868
C 5.2279855908 -0.6956365623 3.0266040524
O 3.3911083111 -0.0414072241 4.8491060582
F -4.9889361197 2.6906465595 4.3789404126
F -4.9315330437 0.6727367583 5.1753314677
H -2.4271476148 0.8576566637 0.6945732113
H -4.2642780628 1.485335747 2.2186142784
H -1.5799422065 1.2913399502 5.5698471555
H 5.6379484293 0.1437874428 3.5862291885
H 5.2556967688 -1.5915720822 3.6471724288
H 5.8035293127 -0.8553709951 2.1210060844
H -0.2153326738 1.0868587101 -0.5150820633
H -0.6115425106 -0.6351864376 -0.2768015393
H 1.6440562302 -1.2103729644 -0.7622550941
H 2.0480836445 0.5138192658 -0.9648196994
H 1.3250124602 2.8369621192 5.3829531568
H 1.6754146219 3.1390550428 7.8223140944
H 0.6446263428 -0.9740329116 8.4747456108
H 0.2941165842 -1.2706008194 6.0349432138
H 1.237931077 2.2956964809 10.1177124722
H 0.8349063652 0.5882705679 10.3867044802
H 2.4968322307 1.0633197069 10.0339977357

48
FlPhMeH+_fig6 1 1 -1480.4934356 0.3658021 0
B3LYP-D3/6-311+G**
C -0.0010969678 0.0055113225 0.0009033931
N -0.0009894805 0.0027951147 1.4789395747
C 1.2390744284 0.0023805718 1.9597815665
N 2.1554076114 0.0611652195 0.9793545274
C 1.5011161402 0.1552294047 -0.3468852285
C 1.5655217893 -0.0865373971 3.308281116
N 0.4894248042 -0.1779443368 4.2233646137
C -0.831250251 -0.2159649824 3.7361723038
C -1.0986441974 -0.1290470475 2.3462722857
C -2.3959284726 -0.1612219701 1.8612681556
C -3.4728999185 -0.2799927437 2.7411729788
C -3.2197378246 -0.3559404419 4.1027755025
C -1.9125380663 -0.3274692866 4.6025090198
C 3.519285682 0.0654591104 1.2063633994
N 3.8555223505 -0.0235996103 2.5554789308
C 2.9223926032 -0.1246648147 3.5716455158
C 0.7165645024 0.0812296753 5.6203411577
C 0.7861892628 1.3992067163 6.0797201411
C 1.0065212185 1.6427946313 7.428986779
C 1.1519600077 0.5877455177 8.3422752067
C 1.0689107909 -0.7232960599 7.8649436417
C 0.8472871477 -0.9828526073 6.5123767772
C 1.3884507445 0.8718874692 9.8007630728
C -4.3462015317 -0.537556247 5.0727030752
F -4.1705623522 0.171524058 6.2165861083
O 4.3262106715 0.1388907102 0.2996997225
C 5.2868149285 -0.0325732434 2.908012929
O 3.4816096058 -0.2512351587 4.7707155002
F -5.543065978 -0.1663135483 4.5655513237
F -4.4870308849 -1.8381848505 5.462070165
H -2.5722423236 -0.0863828301 0.7963817765
H -4.4830651614 -0.304228411 2.3570171001
H -1.739759479 -0.3895526639 5.6673225552
H 5.4976786256 0.7714475529 3.6109815711
H 5.5549678858 -0.992362797 3.3481646447
H 5.8469909873 0.1215390853 1.992057224
H -0.594004632 0.8393791383 -0.3712487876
H -0.4189855565 -0.9339350015 -0.3620599263
H 1.8610618665 -0.6438817899 -0.9902218826
H 1.7302763296 1.1222832215 -0.7908252075
H 0.6726233886 2.2168011147 5.3777186603
H 1.0674642703 2.6663298447 7.7824530952
H 1.1765957185 -1.5523143413 8.5550371633
H 0.7789177685 -1.9997759552 6.1442397018
H 2.2822780635 1.4875290604 9.9379997707
H 0.546841416 1.4238279763 10.2303969386
H 1.5169504064 -0.0510368567 10.3685403095
H 2.8000348764 -0.2816773791 5.4685536206

2
O2_fig6 0 3 -150.3747581 0.0037253 0 B3LYP-
D3/6-311+G**
O 0. 0. 0.1372778412
O 0. 0. 1.3427221588

2
O2*_fig6 0 1 -150.3137677 0.0037 0 B3LYP-D3/6-
311+G**
O 0. 0. 0.1374912797
O 0. 0. 1.3425087203

2
O2rad-_fig6 -1 2 -150.4989732 0.0026832 0
B3LYP-D3/6-311+G**
O 0. 0. 0.0683461241
O 0. 0. 1.4116538759

3
OOHrad_fig6 0 2 -150.9694765 0.013992 0
B3LYP-D3/6-311+G**
O -0.031659008 0. 0.0520782124
O 0.027815312 0. 1.3764828185
H 0.8995122855 0. -0.2652276976

4
HOOH_fig6 0 1 -151.6150736 0.0261638 0
B3LYP-D3/6-311+G**
O 0.0456231654 -0.2817663794 -0.0489188863
O -0.0249571691 -0.2682607246 1.400002872
H 0.8245412126 0.2774273465 -0.20918025
H -0.7964350063 0.3038581404 1.5496967463

48
FlHBn_fig6 1 1 -1480.5130022 0.3685334 0
B3LYP-D3/6-311+G**
C 0.3378619843 0.0698964429 0.2069337709
C -0.0948239247 -0.1321820035 1.5210571996
C 0.8453255 -0.4707890804 2.5007556434
C 2.1967967236 -0.5716005584 2.1793840637
C 2.621986957 -0.3438722812 0.8706409267
C 1.6881296305 -0.0329864761 -0.1174048069
C -1.5341474323 0.0939606669 1.8969460266
C -1.8126458356 1.5862243047 2.3506285763
C -0.9184575004 1.9446573904 3.4719331815
N 0.2431803891 2.5169296232 3.3213375061
C 0.6659502845 3.0029553224 2.0675631423
C -0.3084918478 3.021940107 1.048062732
N -1.5955782487 2.5746791088 1.3110065099
C 1.9634980848 3.4447780885 1.8508252887
C 2.3161393854 3.9425196701 0.6025168212
C 1.3525353993 3.9866081213 -0.4065685817
C 0.0518255573 3.5395114251 -0.195713162
N -1.2056581706 1.6474854922 4.7362589166
C -0.0801257644 2.0373060837 5.6271654184
C 0.9445981768 2.63372744 4.6275482601
C -2.4886832383 1.2947241299 5.1686879
N -3.4683156775 1.2595248221 4.1739035286
C -3.2659183513 1.6140057148 2.8430842324
C 1.7157889288 4.5730701231 -1.7389708048
F 2.9993707061 4.3183087095 -2.086175666
O -2.7113238551 1.0778708642 6.3358830054
C -4.8485040887 0.9968071304 4.6175921442
O -4.1766383712 1.8320878031 2.0784307325
F 0.9401610822 4.1102401181 -2.7465124983
F 1.5843520922 5.9297637868 -1.7529263658
H 2.6920113499 3.4011568867 2.6494362582
H 3.3252826735 4.2864091638 0.4222443159
H -0.6838465924 3.5641060983 -0.9890676841
H -4.8220693313 0.2544649953 5.4103718255
H -5.3108880837 1.9141860843 4.9844169166
H -5.4105469948 0.6164034219 3.7694817093
H 1.1656684167 3.681643538 4.8190692115
H 1.8663003006 2.0571592397 4.5827126471
H 0.3017371505 1.1558927546 6.1362719422
H -0.4306474014 2.7683226345 6.3517529316
H -2.2083948329 2.4833399233 0.5098991933
H -1.835734565 -0.5763305459 2.7040644306
H 0.5172729865 -0.6439511484 3.5207555582
H 2.9166818255 -0.8254825125 2.9491752063
H 3.6744741187 -0.4145863802 0.620680422
H 2.0119593231 0.1375142106 -1.1377626854
H -0.3858658903 0.3286002176 -0.5578200353
H -2.1964003704 -0.0717768187 1.0447221941

37
1aH2O2_figS31c3 1 1 -1360.3221128 0.2665884
0 B3LYP-D3/6-311+G**
C 0.0002465555 0.0020563006 0.0010552324
C 0.0011155015 0.0021703997 1.5514460096
C 1.3896142591 0.0004747477 2.0657950876
N 2.3919437611 0.5450269098 1.3827798193
C 2.3125096169 0.9015381955 0.0165613523
N 1.088393342 0.6184803905 -0.6026961522
N 1.7413801758 -0.5193786245 3.2127956393
C 3.1962071633 -0.2994340263 3.4471950996
C 3.6519618205 0.4560467076 2.1624242727
N -0.6601756957 -1.119949765 2.0935384385
C -0.3989328387 -1.5734165661 3.3835342782
C 0.8376980641 -1.2685029365 3.9893584919
C 1.1526008034 -1.7210513821 5.2680787999
C 0.2395457536 -2.5045278462 5.9550059993
C -0.9809225435 -2.8270172525 5.3541208622
C -1.30457166 -2.3702511295 4.0840889362
O -0.5905296273 1.2905113869 1.8193098002
O -0.5047951014 1.5103207307 3.253958853
C -1.9471405272 -3.7250818262 6.0936832867
F -1.5513739853 -5.0128812931 6.0203988646
O 3.2669671554 1.3597669629 -0.5460152044
C 1.0219338799 0.855675322 -2.0631958428
O -0.9364252526 -0.4599361981 -0.593779924
F -2.0176382398 -3.3984672879 7.397002727
F -3.189697584 -3.6528109551 5.5809369764
H 2.1008514426 -1.4644533475 5.7224604016
H 0.4671752387 -2.8591603763 6.9509319683
H -2.2589970183 -2.6226532228 3.6402896176
H -0.0228732065 0.9727773778 -2.3352542907
H 1.5850801469 1.756646762 -2.2885910411
H 1.4498946728 0.0094680802 -2.6007497718
H 3.6868086965 -1.2641136388 3.5734681846
H 3.3284508482 0.2990805091 4.3473016325
H 4.0226129844 1.456828339 2.3747295266
H 4.3993633889 -0.0877076409 1.5874931897
H -1.4329267221 1.6976150329 3.4641431756
H -1.5045844625 -1.4129990071 1.6174333733

37
1aH2O2_figS31c1 1 1 -1360.3213768 0.2664983
0 B3LYP-D3/6-311+G**
C 0. 0. 0.
C 0. 0. 1.544487
C 1.3804981034 0. 2.0879456981
N 2.4257665205 0.4232843036 1.3924147239
C 2.3940405698 0.6696567645 -0.0006409015
N 1.1741004849 0.4004250821 -0.6287270375
N 1.6671964386 -0.41571913 3.2948660457
C 3.1255297971 -0.2525623392 3.5550360621
C 3.6552242277 0.360413115 2.2223907651
N -0.6599280048 -1.1246131398 2.0791732193
C -0.4896445061 -1.4506111522 3.4285298193
C 0.7057678645 -1.0853372476 4.0810587158
C 0.9354861677 -1.4154579778 5.4139216302
C -0.0234603808 -2.1344166841 6.1098998007
C -1.2031161388 -2.5144338622 5.464679223
C -1.4417723675 -2.1788746336 4.1383809935
O -0.6877522805 1.1772909663 2.0278503497
O -0.0028069858 2.3428794691 1.4775915022
C -2.2195513243 -3.3441369865 6.2170066555
F -1.8574944297 -4.6446032704 6.2255302557
O 3.3832943913 1.0277980431 -0.5754940364
C 1.1701103575 0.5614761375 -2.1008784971
O -1.0103019914 -0.3031051233 -0.5805953522
F -2.3259692515 -2.9466707027 7.497250435
F -3.4400927793 -3.267699685 5.6555423966
H 1.8530463134 -1.1141209836 5.9026773399
H 0.1373003762 -2.3927149716 7.1476078426
H -2.3681025481 -2.4698833481 3.6599319338
H 0.1669385374 0.3417171226 -2.4515401093
H 1.4492269683 1.5836320773 -2.349748318
H 1.8859859747 -0.1278032365 -2.54514952
H 3.5573907353 -1.2276911501 3.7789802969
H 3.2711655711 0.415288241 4.4026487426
H 4.0571022798 1.3632016001 2.3529639265
H 4.3991396661 -0.2620062593 1.7286069581
H -0.7620339433 2.8606151557 1.1666663304
H -1.515474481 -1.3919069982 1.6064959483

36
1aH2O_figS31_minimum 1 1 -1285.1947204
0.2637641 0 B3LYP-D3/CHKBAS
C -0.000936139 -0.0030452521 0.0021995948
C 0.0005453263 -0.0035323225 1.3968688927
C 1.240842539 0.0027377801 2.068090464
C 2.4465828469 -0.0142667577 1.3714743545
C 2.4275075161 -0.0197876792 -0.0137369549
C 1.2029568387 -0.0079162038 -0.6883723801
N 1.1837556168 0.0869926197 3.4755092419
C 0.0543877191 -0.0644591769 4.1169440071
C -1.1888516643 -0.5296336766 3.4530474363
N -1.1720873155 0.0492974613 2.1497108226
N 0.1087101565 0.2609796286 5.4034831458
C -1.0311477654 0.4355230807 6.2194193925
N -2.2603364841 0.2328196924 5.5794292797
C -2.4361464418 -0.0760748704 4.2368990951
C 2.2443221292 0.5515224513 4.413643523
C 1.4867046143 0.6751172043 5.7704515234
O -3.5089304011 -0.1012495873 3.6839080681
O -1.1181306755 -1.948567082 3.5073116292
O -0.9146251841 0.7731570668 7.3638581139
C -3.4629459918 0.4790172871 6.4088250051
C 1.199570272 0.0315895379 -2.2001883714
F 0.014598252 -0.3366053814 -2.7041567664
F 1.459336814 1.2733610305 -2.6412394426
F 2.1346755867 -0.7800766179 -2.7091092335
H -1.9586657934 -2.327795571 3.2108083558
H 3.3883917905 -0.0239599282 1.9050520529
H 3.3548667853 -0.0394972812 -0.569860795
H -0.9393860277 -0.0080500083 -0.5371982815
H -4.3200268634 0.0831268329 5.8733105937
H -3.3362751142 -0.0233095152 7.3645125395
H -3.5882478811 1.5488050701 6.5728782467
H 2.6421938799 1.501150401 4.0571992281
H 3.0428502197 -0.187796425 4.4524817763
H 1.8790417922 0.0040705909 6.5317654552
H 1.4702032497 1.6904344394 6.1627152716
H -2.0645772664 0.1117291997 1.6754491193

53
1bOOH_figS32a 1 1 -1703.435313 0.4048386 0
B3LYP-D3/6-311+G**
C -0.5514464685 -0.3805784191 0.362802395
N -0.2886673491 -0.3311733913 1.6724033534
C 1.0358749069 -0.6831072099 1.8695457737
C 1.5617419562 -0.948488357 0.6442534185
N 0.5513419337 -0.7657885198 -0.2824579413
C -1.2314611986 0.1325532643 2.7081892493
C -0.7718068362 1.4014536305 3.4305383131
C -0.5162201078 2.626294421 2.5389150703
N 0.8594420938 2.6732651818 2.0093325461
C 1.069096382 2.4139260066 0.6547279694
C 2.4872556385 2.4551252865 0.2154174065
C 3.5316792013 2.4890513819 1.2847336639
N 3.123547881 3.1549952518 2.4713151198
C 1.8336284614 3.2100330194 2.9040080484
N 2.8055476562 2.4439159136 -1.0299555687
C 4.1253631474 2.6475036079 -1.3829987159
C 5.0791092961 3.1289687814 -0.4373563135
N 4.6921691013 3.1961261942 0.8732978153
C 6.338253834 3.5579325034 -0.8839867573
C 6.6695974984 3.4367512259 -2.2205664757
C 5.7531272958 2.908625958 -3.146501179
C 4.4885359874 2.5393602728 -2.7343331315
C 4.2265401645 3.8963568375 3.0915719981
C 5.2428471344 4.0496510569 1.9326610001
C 6.1736713128 2.7525134733 -4.5820625002
F 7.0753737477 1.7509552241 -4.7231819983
O 3.7924097451 1.0680622104 1.5062733408
O 4.7880188914 0.9413805001 2.5563574421
O 0.1512260007 2.1423911579 -0.0996139211
O 1.5088185036 3.6868736164 3.9694982558
C 0.7312259108 -0.7307126025 -1.7383118678
F 5.1328238047 2.4666528788 -5.3889049279
F 6.7603922538 3.8732593102 -5.0525738569
H 7.0550521873 3.9626418965 -0.1813056152
H 7.648867792 3.7528716468 -2.5580122286
H 3.7467909025 2.1907279466 -3.4399850595
H -0.6635477969 3.5292442386 3.1260854539
H -1.1877692521 2.6508652226 1.6829833177
H 5.3123266518 5.0797127874 1.5757815507
H 6.2361138869 3.7121958517 2.2343837762
H 4.6474224678 3.3302790929 3.921555047
H 3.8602466851 4.8524954285 3.4607725065
H 5.5542939497 0.6295082908 2.0518888711
H -1.567145513 1.6500205467 4.1385663445
H 0.1129987641 1.2017900225 4.0396356022
H -2.1823393679 0.3017637793 2.2015068611
H -1.3734916408 -0.6825088329 3.4198429361
H 1.4892968634 -0.7008379498 2.8443527266
H 2.5583026738 -1.229329504 0.3552820586
H -1.4835840943 -0.1094214495 -0.0998087804
H 1.4022095621 -1.5358828072 -2.0314018249
H -0.2340981923 -0.8729665422 -2.2196051057
H 1.1443905626 0.2404343602 -2.0141769843

53
1bOOH_figS32b 1 1 -1703.4338546 0.4048729 0
B3LYP-D3/6-311+G**
C -0.6752998651 1.0803310211 0.30738315
N -0.370993763 0.4690971842 1.5100121558
C 0.9451824326 0.2436237846 1.5561744547
N 1.4921202889 0.6931948797 0.4237237423
C 0.4939836167 1.2184585045 -0.3750407374
C -1.3354950504 0.0176051692 2.5338484216
C -2.3578329675 -0.9700087096 1.9740661744
C -1.7612745084 -2.1253153074 1.1562591043
N -0.5291113302 -2.6893570232 1.7375745929
C 0.6182344888 -2.7256715116 0.9111082293
N 1.7462522537 -3.2061640385 1.5087027414
C 1.8607521534 -3.3925251092 2.9136540168
C 0.5990341587 -3.8735114073 3.556843297
C -0.6694159215 -3.3641060796 2.9717699255
N 2.9399251247 -4.2908215851 3.0656217314
C 3.7133935932 -4.4635464833 1.8293634411
C 2.9774031688 -3.5676601604 0.8041923465
N 0.6107264121 -4.6820978926 4.5513141895
C 1.8139604057 -5.21541714 4.9693493152
C 2.9918389397 -5.0979693259 4.1795277021
C 4.1323969483 -5.8346938609 4.5234040588
C 4.1246181942 -6.6151967748 5.6660105356

C 2.9820011833 -6.6906616258 6.4788650489
C 1.8324672168 -6.0121627316 6.1243925044
O 2.0817382041 -2.1030509604 3.6006458501
O 3.2855948522 -1.4845816026 3.0535495085
C 3.0361421611 -7.5225481795 7.7320017159
F 3.511668715 -8.7622702904 7.4798613201
O 0.607713513 -2.342772554 -0.2452321072
O -1.7511271808 -3.4644546812 3.5004941314
C 2.9018845472 0.5448546999 0.0525576139
F 3.8619302335 -6.9691093907 8.6516212097
F 1.8276135926 -7.6574125331 8.30683795
H 5.022372846 -5.7805290087 3.9096939517
H 5.0121178985 -7.1733127321 5.9372292744
H 0.923702315 -6.0943384365 6.705065795
H -2.5032640351 -2.9200642802 1.073444282
H -1.4942715605 -1.8039459789 0.1532404736
H 3.7053985592 -5.5160951981 1.5368422025
H 4.7483110802 -4.1428350184 1.9603164008
H 3.5623099396 -2.6797496077 0.5624424948
H 2.7358118645 -4.0940449463 -0.1175267257
H 3.8817476833 -1.5378662688 3.8154210097
H -2.9154030226 -1.3602904977 2.8257835069
H -3.0769908054 -0.4489914976 1.3355913189
H -1.8299759558 0.8966184778 2.9488567752
H -0.7432014158 -0.4389648417 3.3262377253
H -1.6801867979 1.3583549281 0.0426109667
H 0.697955154 1.6336405295 -1.3466124919
H 1.4737004539 -0.2422209908 2.3602266886
H 3.3083564747 1.5145724363 -0.2314835601
H 3.4431192337 0.155713991 0.9121806908
H 2.9768204307 -0.1553611472 -0.7790961089

53
1bOOH_figS32c 1 1 -1703.4345519 0.405409 0 B3LYP-D3/6-311+G**
C -0.4453755004 0.6621127987 -0.2007911401
N -0.2435807052 0.494761299 1.1109936191
C 1.1195528465 0.4555987833 1.3447175889
C 1.7358015921 0.5925458771 0.1378721765
N 0.739740389 0.7256812391 -0.8125874553
C -1.3062432956 0.3228191139 2.1329802885
C -2.322168401 -0.7465077508 1.7334310647
C -1.8383730804 -2.2006192121 1.8472119173
N -0.4848428349 -2.4777201015 1.3296971432
C -0.2785400394 -2.4256161302 -0.0457338625
C 1.0856732822 -2.8224389687 -0.487300634
C 1.8919645828 -3.6191643234 0.4968512451
N 1.6955017815 -3.1831191885 1.8536857711
C 0.5587731459 -2.5862122907 2.2888726036
N 1.5432971049 -2.4808565726 -1.6364978506
C 2.8590075807 -2.7657509459 -1.9512908036
C 3.7810646851 -3.189050613 -0.9520645637
N 3.2910110088 -3.4288168529 0.3005745666
C 5.1461935051 -3.280750466 -1.2680132666
C 5.5730868873 -3.0084647263 -2.5541920416
C 4.6595240824 -2.629699932 -3.5529720783
C 3.3186989098 -2.4991474292 -3.24954772
C 2.9414472666 -3.1869290591 2.6273697654
C 4.0343213204 -3.4422562414 1.5599204677
C 5.1744149246 -2.3300396168 -4.9338261673
F 5.9115619595 -3.3490417138 -5.422376579
O 1.4410445013 -4.9578330801 0.280036531
O 2.1183958515 -5.80740097 1.2549312759
O -1.1485591372 -2.0452246133 -0.806316366
O 0.4291215856 -2.13769031 3.4139466636
C 0.9358160972 0.7610936898 -2.2683575048
F 4.1775344744 -2.09038639 -5.8076070061
F 5.9752888708 -1.2358854107 -4.9298733099
H 5.8580431631 -3.5909547581 -0.5142073988
H 6.6243012738 -3.1014039946 -2.7977943214
H 2.600637618 -2.1915943581 -3.9979068385
H -1.8251296955 -2.5061520358 2.8901913629
H -2.5275021785 -2.8446305024 1.301005372
H 4.7932383622 -2.6580084746 1.550520574
H 4.5191089314 -4.4068215666 1.71960118
H 2.9197267977 -3.9756183604 3.3780845228
H 3.0608708085 -2.22819894 3.131923164
H 2.3628584027 -6.5510973886 0.6845633226
H -3.1808891717 -0.6391199287 2.401381495
H -2.69407282 -0.5553251436 0.726074855
H -0.796984532 0.0551342516 3.0575009615
H -1.793885556 1.289344725 2.2663946615
H 1.5150977062 0.3272012645 2.336963082
H 2.7784744403 0.6125812853 -0.1267397304
H -1.3991426136 0.6931428083 -0.6957734563
H 1.8383603468 1.3281313651 -2.4875365253
H 1.0224718029 -0.2640281712 -2.6284937845
H 0.0808717101 1.2524704611 -2.7280350152

53
1bOOH_figS32d 1 1 -1703.4304792 0.4049984 0 B3LYP-D3/6-311+G**
C -0.0223765069 0.1900557626 -0.0213922773
N -0.0045736718 0.0917281193 1.43667959
C 1.3224538691 0.0219416097 1.9561011202
N 2.1003171899 0.6629119514 0.925869501
C 1.413051272 0.6584119552 -0.3691043725
C 3.1967038731 1.4236756645 1.1774717678
N 3.6246000187 1.4631719139 2.5248997774
C 2.7932526437 1.2360511328 3.6271333973
C 1.4372268492 0.7238399769 3.2794859421
C -1.0761972019 0.1515274419 2.2854882719
C -0.8258533635 0.5163564398 3.6383630157
N 0.435330471 0.9041065783 4.05739865
C -2.3978622801 -0.0515432567 1.8585390996
C -3.4368896886 0.0614850672 2.7650045024
C -3.1895683006 0.3880028572 4.1085722569
C -1.8968165121 0.6226314533 4.5372050696
C 4.9871529595 1.9669261517 2.7851827744
C 5.0655507133 3.4022665194 3.3214734752
C 4.2875146295 4.450606489 2.5266235644
N 2.8244595841 4.3514034024 2.7507358971
C 1.9075254718 4.1591092517 1.7981146576
N 0.7059715634 4.0548767875 2.3804500946
C 0.8615819454 4.1948473073 3.7481293457
C 2.1882302609 4.3839586518 3.9790766875
C -0.5634563714 3.7997833798 1.6937646123
O 3.1556603329 1.4807903202 4.7561574106
O 1.7774160854 -1.2988466812 2.2447782143
O 1.7811092417 -2.0411970758 0.9871199102
O 3.7645582771 2.0688034013 0.3102045708
C -4.3555458397 0.5118018238 5.0512442073
F -5.1869927436 1.5119026785 4.6671906616
F -5.0937977647 -0.6175732383 5.078904777
F -3.9657568058 0.7773036793 6.3114710328
H -2.6013071799 -0.3234759937 0.8308739096
H -4.4532716731 -0.1156157568 2.4352443493
H -1.6814074849 0.898998137 5.5607344852
H 5.525250687 1.8858299077 1.8440788777
H 5.443459708 1.2952706562 3.5121761709
H -0.7807517991 0.9102832368 -0.3353242883
H -0.2401525004 -0.7754675797 -0.4806313818
H 1.9042405474 -0.0284825578 -1.0508603702
H 1.440348842 1.6582783533 -0.802967766
H 1.4159430492 -2.8844944452 1.2932838624
H 6.1177645552 3.6983539798 3.2962841556
H 4.7639932002 3.4249175221 4.3691918808
H 4.5879228218 5.4568728261 2.8214481952
H 4.4464191498 4.3344464774 1.4552285677
H 2.7243043539 4.5026235732 4.9032902687
H 0.0320442218 4.1219387379 4.4289725224
H 2.1167899731 4.0648979916 0.7462021316
H -0.9930026819 2.8708535203 2.0668177698
H -1.250181094 4.6258029042 1.8747495883
H -0.3745797184 3.7163855432 0.6255104464

53
1bOOH_figS33b_bluerect 1 1 -1703.4322257 0.4050565 0 B3LYP-D3/6-311+G**
C -0.0020511887 -0.0071445332 -0.0162011702
N 0.0122097408 -0.019798326 1.3229344624
C 1.3263309504 -0.0335362683 1.7492172121
C 2.1103812182 -0.028709557 0.6391339577
N 1.2635509062 -0.0112077938 -0.449927679
C -1.1585626765 -0.027986266 2.2287377938
C -2.2117787419 -1.0863634552 1.9017315805
C -1.6919490125 -2.5326607213 1.7947176175
N -1.4307747232 -2.9137098165 0.396640041
C -0.1048636971 -3.0447639939 -0.0358576365
C 0.0464862187 -3.280029045 -1.499320293
C -1.1749266987 -3.0615081995 -2.3331853411
N -2.3804687529 -3.4341755138 -1.6908238043
C -2.5861978636 -3.279633298 -0.3529517504
N 1.1725005509 -3.600786961 -2.0184410672
C 1.2193325282 -3.8975609561 -3.3750103343
C 0.0305747846 -4.0930691171 -4.1374920283
N -1.1684833257 -3.8111021715 -3.5330247084
C 0.1216639482 -4.6033590171 -5.4394193069
C 1.3650571736 -4.843138479 -5.9979176446
C 2.5402830553 -4.6034344784 -5.268539179
C 2.4659362489 -4.1543299382 -3.9635442149
C -3.355542432 -4.0010648824 -2.6257979137
C -2.4790469772 -4.4038988244 -3.8373657766
C 3.8774624606 -4.813615044 -5.92555512
F 4.2346046449 -3.7343066468 -6.6651094316
O -1.1064252017 -1.6051873569 -2.5793040971
O -2.2785117008 -1.1977008132 -3.3312888872
O 0.8378135618 -2.9090111182 0.7165065954
O -3.6620415641 -3.437393469 0.1801311281
C 1.6953473634 -0.0734763229 -1.850646927
F 4.8579292007 -5.0141797237 -5.0233550318
F 3.862880609 -5.872078163 -6.7601804702
H -0.776289778 -4.7917675784 -6.0135583716
H 1.4321706324 -5.2232777927 -7.0093269384
H 3.3549799883 -4.0224278878 -3.3611912256
H -0.7620272503 -2.675183194 2.3414701129
H -2.437996305 -3.2193127853 2.1858953194
H -2.3762838085 -5.4871618611 -3.932509476
H -2.8867275469 -4.0056026539 -4.7680652071
H -4.1019531839 -3.2554062521 -2.8980052803
H -3.8535133399 -4.8527431164 -2.1657705752
H -1.9032922693 -1.0633266691 -4.2148819556
H -2.9497708157 -1.0304263777 2.7059186851
H -2.756383166 -0.8247844856 0.9912849872
H -1.6001685083 0.9703225165 2.2208909688
H -0.748830817 -0.2009054182 3.2237754619
H 1.5859528956 -0.0643058231 2.7924798727
H 3.1800728397 -0.0627724485 0.5306641263
H -0.8705081987 -0.0348918634 -0.6517236727
H 2.2508674786 -0.9981913901 -2.0034250397
H 0.8150550101 -0.0768350046 -2.4868856388
H 2.3216571137 0.7892780715 -2.0738156445

53
1bOOH_figS33c_bluerect 1 1 -1703.4297698 0.4048427 0 B3LYP-D3/6-311+G**
C -0.0011363403 -0.0357506976 0.0019500335
N -0.0049607019 -0.007264488 1.3865253626
C 1.2615283486 0.0269888057 1.8204494318
N 2.0687820385 0.024701781 0.7536077833
C 1.2991463403 -0.0171121381 -0.395536396
C -1.231591239 -0.0344032648 2.2227454668
C -0.9423027859 0.0643490985 3.7135711322
C -0.465102324 1.4549926853 4.1599424676
N 0.113295461 1.4338420906 5.504399037
C 1.4660026654 1.0724306452 5.6098357501
N 2.0253890368 1.2266674653 6.8281859337
C 1.3319996742 1.8290013878 7.9340452875
C -0.14545855 1.567529715 7.9229100622
C -0.7941245105 1.529224767 6.5888633358
C 3.3362943675 0.6983253273 7.215346882
C 3.2878931756 0.7135408405 8.7648858627
N 1.9594026184 1.2440845599 9.0654800418
N -0.8124781806 1.3901489739 9.0038562074
C -0.1375852102 1.3182847318 10.2052998135
C 1.2785275183 1.1686420988 10.2509184635
C -0.8787751503 1.2492794194 11.3943626922
C -0.2391584743 1.0257216146 12.5979952168
C 1.152210209 0.8333989948 12.6320557043
C 1.9064210349 0.8943619207 11.4745367272
O 1.4071090422 3.264326471 7.8934535511
O 2.8190007672 3.6329556909 7.8705390466
O 2.0870322029 0.6282788744 4.64340761
O -1.9849531829 1.5783433059 6.3925136941
C -1.0091294446 0.9863677099 13.8888217537
F -2.3399861807 0.9618219354 13.6897887643
C 3.5347127859 0.0672952418 8.0093071092
F -0.686345337 -0.1030631192 14.6234577238
F -0.7377656132 2.0664757829 14.6601988218
H 2.9786168457 0.7527828566 11.5166774
H 1.6417063384 0.6373357429 13.5779279727
H -1.9533427593 1.3566487437 11.3342841473
H 0.2906445207 1.8661203232 3.4929515332
H -1.308498084 2.143106153 4.1867751895
H 3.3901930021 -0.2870148887 9.1896892025
H 4.0715558281 1.3556968596 9.1719297272
H 4.1317170828 1.3375665959 6.8337671582
H 3.4593553659 -0.3058199326 6.8109696744
H 2.8424651762 4.2553794787 8.6120293746
H -1.8669173953 -0.1722256315 4.2432940846
H -0.214111321 -0.69730956 4.0030458374
H -1.8627978765 0.7916116116 1.8871095002
H -1.7467032554 -0.9687672302 1.9953479485
H -0.9115643872 -0.0691300314 -0.5712408681
H 1.7360482044 -0.0294099568 -1.379118572
H 1.5881161628 0.0954848032 2.854252137
H 3.8936312185 0.9307244483 0.2504481082
H 3.8382160792 0.1561248173 1.8503270521
H 3.9434474045 -0.8488575793 0.3840530176

53
1bOOH_figS33d_bluerect 1 1 -1703.4338132 0.4045849 0 B3LYP-D3/6-311+G**
C 0.0184638418 0.0032021748 0.0029473736
N 0.017931407 -0.0049579535 1.340536643
C 1.3284950716 0.0226891496 1.7857443028
C 2.125318616 0.0446573597 0.6837925237
N 1.2874317945 0.0323366594 -0.418400696
C -1.178075136 -0.0121037182 2.2124376632
C -1.5096312182 1.37426432 2.7656830259
C -2.07133379 2.3774226607 1.7538166098
N -3.446071072 2.0808537856 1.3165171439
C -3.6403337103 1.3350978128 0.1598163695
C -5.0510127388 1.1879812657 -0.2670061413
C -6.0266194427 2.153796501 0.332553671
N -5.7358098323 2.4738575538 1.6972381271
C -4.4809334452 2.5011277542 2.2084744155
N -5.4086118202 0.286634585 -1.1087992343
C -6.7513489594 0.1187643587 -1.3773469378
C -7.7518870937 0.7312515769 -0.5666581705
N -7.3381184149 1.6072551886 0.3966212369
C -7.1319142941 -0.789164029 -2.377647773
C -8.4643555093 -1.0958401793 -2.5667523467
C -9.4437792673 -0.5238503739 -1.7356235055
C -9.0975479212 0.3717556732 -0.7418702645
C -6.9506189549 2.6262175417 2.503487515
C -8.0549118709 2.0058516072 1.6093168491
C -8.8963558465 -2.0461829928 -3.6481790524
F -9.6653386694 -3.0430037099 -3.1522871585
O -5.9137799161 3.2974850245 -0.5345372888
O -6.7961993789 4.3349399243 -0.0135767357
O -2.6999881329 0.8436408201 -0.4479007336
O -4.2177796907 2.8345786721 3.3455913137
C 1.7168467145 0.0455105879 -1.821637893
F -9.6374928205 -1.4200807321 -4.5924439583
F -7.8493529694 -2.617545021 -4.2748021213
H -9.8628490444 0.8113399968 -0.1155501626
H -10.4849788454 -0.7857865628 -1.8779050607
H -6.3556364445 -1.2471263823 -2.9752422976
H -2.094383313 3.3611582198 2.2183277961
H -1.4584172257 2.4340155344 0.855013075
H -8.514628475 1.1289374607 2.069573757
H -8.8318497993 2.7387893456 1.3839558415
H -7.1432924801 3.6789539258 2.7062493156
H -6.828416803 2.0986219266 3.4485297395
H -7.3048664437 4.5414617349 -0.8112780693
H -2.2333905741 1.2553866117 3.5739903316
H -0.6115365616 1.8068234392 3.2178149099
H -0.961672738 -0.7062705831 3.0250228625
H -1.9963617187 -0.4244841235 1.6258113444
H 1.5771990874 0.0139266659 2.8327766111
H 3.1971955882 0.0608941277 0.58811908
H -0.871037529 0.0285125561 -0.6070175903
H 2.3102543308 0.9391264713 -2.0115882209
H 2.3067435561 -0.8463078304 -2.0306915782
H 0.8337602008 0.0544831376 -2.4568103782

34
1aN10H_figS35 1 2 -1209.371499 0.2478307 0 B3LYP-D3/6-311+G**
C -0.0082924117 -0.1042186261 0.0255936868
C -0.0391442608 -0.2975853551 1.4952725868
C 1.1866002283 -0.3900980936 2.0919780392
N 2.3759237519 -0.1691149 1.5198169174
C 2.4890156638 0.0828846319 0.1532319126
N 1.2776431276 0.0356890421 -0.5372288565
N 1.3671475162 -0.6524649644 3.5070500742
C 2.6890408391 0.0695975883 3.822052613
C 3.4687297864 -0.1157282533 2.5147106343
N -1.1884282834 -0.2927339569 2.1853618712
C -1.0733730581 -0.252565451 3.5566730901
C 0.1414598809 -0.3466466072 4.2776582925
C 0.2111179453 -0.2480471033 5.6517216716
C -0.9616319678 -0.0322580495 6.3698109618
C -2.1801923448 0.0639482605 5.6889387768
C -2.2450505803 -0.0489056357 4.3108641333
C -3.4317964919 0.2921590663 6.4913924425
F -3.6938888545 -0.747122751 7.3243917856
O 3.5653571696 0.314152441 -0.3567981362
C 1.3255568603 0.250769721 -1.9919089371
O -1.0029101266 -0.0559248427 -0.6686450986
F -3.3300911012 1.3922182259 7.2783246745
F -4.5267870642 0.4609512076 5.7234660408
H 1.1591626788 -0.3397403641 6.1655444679
H -0.9212677935 0.0552553769 7.4478108478
H -3.1831422595 0.0323891499 3.7789289547
H 0.9847161844 1.2590200409 -2.2318828133
H 2.349362981 0.119931728 -2.3264471133
H 0.6766702802 -0.4757126517 -2.4758598138
H 3.1487635279 -0.3965073748 4.6866564588
H 2.4326306168 1.1097005681 4.0091678932
H 4.1222333096 0.7283486533 2.3107637358
H 4.0338733445 -1.0474527704 2.4907272931
H 1.5519979171 -1.664852056 3.6195189098

34
1aC4OH_figS35 1 2 -1209.4097716 0.2468777 0 B3LYP-D3/6-311+G**
C 0.0843952105 -0.1510950586 0.0375879021
C -0.0404720989 -0.2977639851 1.4262620699
C 1.117887153 -0.7306542688 2.0646940948
N 2.2707910291 -0.9953129676 1.4341347893
C 2.4199627385 -0.8674828994 0.0653313383
N 1.2556914598 -0.4145363868 -0.6016446922
N 1.1828567578 -0.931531768 3.3728282725
C 2.5347030608 -1.4007069167 3.7633429161
C 3.2983534467 -1.4460049478 2.409043917
N -1.1853231923 -0.052010416 2.1089569391
C -1.1294871002 -0.2578939103 3.4588453842
C 0.0483916111 -0.7048194845 4.1501237364
C 0.0517714809 -0.9021092646 5.5264304553
C -1.1048606081 -0.6637449535 6.2548555574
C -2.2656570771 -0.2243570071 5.6001286715
C -2.2854718985 -0.0253494722 4.2317591708
C -3.5087445737 -0.0074821199 6.4127004871
F -4.0461401081 -1.1818526755 6.8431924489

O 3.4558551676 -1.1207760056 -0.5068668116
C 1.3229041028 -0.2509282298 -2.0667971578
O -0.9220523312 0.2448208083 -0.7122455511
F -3.2685664552 0.7238396837 7.5304359763
F -4.482404741 0.6315486471 5.7304196372
H 0.9517684627 -1.2390587421 6.023713439
H -1.1048966138 -0.8175854171 7.3262728104
H -3.1785382968 0.310178151 3.7220531043
H 2.3581467734 -0.37446787 -2.3651943857
H 0.7043663876 -1.0081191391 -2.5484459039
H 0.9746490957 0.7445579643 -2.3347495475
H 2.455882231 -2.3822798525 4.2274594461
H 2.9748308177 -0.6923582263 4.4630439605
H 4.1423235986 -0.7605793315 2.388555975
H 3.6249757343 -2.4510024491 2.152694946
H -1.699136695 0.4019192052 -0.1385725443

34
1aN3H_figS35 1 2 -1209.3760535 0.2472519 0 B3LYP-D3/6-311+G**
C -0.1344929743 0.2348081145 0.0383708482
C -0.0929019819 0.1865258237 1.4581458282
C 1.1494661554 0.1587330744 2.087170444
N 2.3788742907 0.1618944063 1.4887870852
C 2.5735864848 0.1546106733 0.1532599857
N 1.3075456593 0.4135312455 -0.6392655302
N 1.2772204417 0.0803748403 3.4067424498
C 2.6933321435 0.0508263924 3.8321466274
C 3.4605564254 -0.0078389922 2.4943430373
N -1.2458519967 0.12633673 2.1878756197
C -1.1162839808 0.0554030329 3.5396313092
C 0.1438335637 0.034606762 4.2174046837
C 0.2312776354 -0.0343753284 5.6032291626
C -0.9328436363 -0.0877443193 6.3576551994
C -2.1805830304 -0.0711494936 5.7152126353
C -2.279947202 -0.0024160602 4.3377354017
C -3.4196990131 -0.1633918524 6.5573436833
F -3.5627116201 -1.3957271558 7.1176696346
O 3.6193631934 0.0056835737 -0.4083304551
C 1.3579059592 -0.3006230793 -1.9652978383
O -1.0596522311 0.2090530507 -0.7241842594
F -3.4026551935 0.7159585683 7.5913980238
F -4.5516704776 0.0751538432 5.8623034018
H 1.1966433688 -0.0441897601 6.0914110802
H -0.8716162303 -0.1410397939 7.4369716261
H -3.2396702653 0.0086772664 3.8390916918
H 2.2698281646 -0.0059121543 -2.4739095149
H 1.3395958183 -1.3710855241 -1.7744796209
H 0.4871935217 0.0088593777 -2.5329763462
H 2.8703815587 -0.827715265 4.4493063091
H 2.912886875 0.9539955535 4.4003288602
H 4.1800984698 0.8001653547 2.3897270608
H 3.9460169022 -0.9687046774 2.3364570618
H 1.3415090425 1.4254431228 -0.8399267221

34
1aC2OH_figS35 1 2 -1209.3914633 0.2462912 0 B3LYP-D3/6-311+G**
C -0.0190589872 -0.1182314292 -0.0276674531
C -0.0946899476 -0.3142315047 1.4100258864
C 1.0433957254 -0.77016507 2.0347281978
N 2.2282840576 -1.0397517228 1.4087909956
C 2.3283089864 -0.8598198883 0.0907391832
N 1.2804987435 -0.4236185602 -0.612807572
N 1.1334615574 -1.0014467552 3.3500101034
C 2.4924232494 -1.4210477952 3.7406124832
C 3.2197973074 -1.5861179228 2.3828463356
N -1.2225440855 -0.0607679404 2.1207015046
C -1.1482902249 -0.2736408036 3.4722093107
C 0.0252506319 -0.7450653629 4.1489079644
C 0.0499441094 -0.930180727 5.5271834553
C -1.0885051255 -0.6589586965 6.274351511
C -2.2479486129 -0.2001825695 5.6348471335
C -2.2845749277 -0.0120546106 4.2626229475
C -3.4734588428 0.0485526805 6.4630282419
F -4.0460083473 -1.1119523723 6.8890782784
O 3.4389975224 -1.0985739118 -0.5631317048
C 1.4420046308 -0.2440551579 -2.0701956113
O -0.9178452265 0.2692776334 -0.743328829
F -3.1986515325 0.7601260012 7.5862011949
F -4.4343752356 0.7256416864 5.7988345292
H 0.9510117985 -1.2821854925 6.011962713
H -1.0733891752 -0.8025940476 7.3471118774
H -3.1776200602 0.3425247513 3.7659466865
H 2.2272589913 0.4842763466 -2.265713212
H 1.6865301853 -1.1993063971 -2.5322262958
H 0.494319318 0.1201391003 -2.4514112026
H 2.451175815 -2.3584605763 4.2919694914
H 2.9474339113 -0.6461510013 4.3570565408
H 4.1387780898 -1.0058358493 2.341872186
H 3.4087072789 -2.6311189742 2.1424910998
H 4.1734920012 -1.3769995041 0.0150629745

34
1aN1H_figS35 1 2 -1209.3650145 0.2473968 0 B3LYP-D3/6-311+G**
C -0.0023142131 -0.2291069704 0.0260565657
C -0.0366759747 -0.2774231553 1.4974001409
C 1.1486332207 -0.3607210903 2.1504917804
N 2.4299835969 -0.5255626242 1.509066548
C 2.4846787379 0.0150673 0.0831378926
N 1.3014424935 -0.084667182 -0.5730851919
N 1.3091911243 -0.2483753038 3.4754019921
C 2.7320046974 -0.2265155107 3.8605689572
C 3.4226939651 0.0591416957 2.5182494081
N -1.2011793278 -0.1474519591 2.1831885991
C -1.0848712633 -0.0928625849 3.55135178
C 0.1630435265 -0.1280108294 4.2580179732
C 0.2227057563 -0.0416473419 5.6439187967
C -0.9536604619 0.0791148098 6.3719940177
C -2.1841718743 0.1138740548 5.7046169618
C -2.2557347795 0.026653648 4.3227982632
C -3.4420525314 0.2034536684 6.5182019746
F -3.7372214619 -0.9767269161 7.1309396334
O 3.5420141675 0.3879624816 -0.3299889798
C 1.3354232595 0.2027216303 -2.0218305696
O -0.9819462885 -0.2315819313 -0.6784234157
F -3.347558343 1.1240197632 7.5103293216
F -4.5256204661 0.5319567087 5.7827212264
H 1.1789780253 -0.0723749975 6.1489679981
H -0.9117996534 0.1446764797 7.4514827305
H -3.2047477346 0.0566804346 3.8046956506
H 1.1900885791 1.2701082299 -2.1931725831
H 2.2973208222 -0.1112327003 -2.4171799799
H 0.535681417 -0.3592180631 -2.4942777529
H 3.0151571193 -1.1919282398 4.2824072771
H 2.9196480124 0.5673399806 4.580591408
H 3.4866221667 1.1245534153 2.3129652664
H 4.3819724921 -0.4315789113 2.3971293549
H 2.6234035109 -1.5383121724 1.4140451645

6
MeCN 0 1 -132.8118844 0.0450331 0 B3LYP-D3/6-311+G**
C -0.0549859525 0.0156467244 -0.0422053108
C 0.0912343042 0.0264960389 1.4005318482
H 1.1356998674 -0.1466809212 1.6677085038
H -0.5260789007 -0.7593335899 1.8404396304
H -0.2244213914 0.9937874313 1.7967983747
N -0.1712241717 0.0076385861 -1.1900271095

13
2MeCNH 1 1 -266.0464388 0.1004653 0 B3LYP-D3/6-311+G**
C -0.0255421514 0.0436866224 0.0625905368
C -0.006448043 0.025007098 1.496756882
N 0.0088162641 0.0097922986 2.6391926999
N 0.0473337571 -0.0252174089 5.2370991147
C 0.0260481288 -0.0141178597 6.3862093573
C -0.0052333112 0.0030833033 7.8293418284
H -0.854091366 0.6706996023 -0.2745501504
H 0.9219063718 0.4506225551 -0.2993691001
H -0.1564763843 -0.9775979596 -0.3037557879
H 0.0284852133 -0.0076982589 3.7454270476
H -0.9699466115 -0.3741662047 8.1755217484
H 0.7944318187 -0.6305872142 8.2184431039
H 0.1346884796 1.026747826 8.183422172

1
Cl -1 1 -460.4086803 n/a 0 B3LYP-D3/6-311+G**
Cl 0. 0. 0.

2
HCl 0 1 -460.8401591 0.006541 0 B3LYP-D3/6-311+G**
Cl 0. 0. -0.1433197332
H 0. 0. 1.1513197332

37
1aC4aOOHN5H_fig36 1 1 -1360.3457474 0.2666805 0 B3LYP-D3/6-311+G** (with additional pc-3 on C of CF3 group)
C -0.0004729433 0.0011932053 -0.0002932103
C -0.0001538924 0.0008016502 1.3929757641
C 1.2374436644 0.0021442291 2.0682391397
C 2.44350734 -0.0202096224 1.3729002744
C 2.4272525755 -0.0264086264 -0.0127856723
C 1.2047772311 -0.0104657451 -0.689530871
N 1.1809367353 0.0784683032 3.4760974145
C 0.0434952494 -0.0337893021 4.1125604578
C -1.2123829524 -0.4574362651 3.4460858681
N -1.1845903952 0.0577109115 2.1347261568
N 0.1072396216 0.2609509489 5.40299685
C -1.0307183001 0.4594369791 6.2197913372
N -2.2624739293 0.3596813832 5.5653014792
C -2.456184863 0.0102779672 4.23322995
C 2.2563054293 0.4824804413 4.4254565468
C 1.5036847338 0.587096228 5.78736519
O -3.5257341327 0.0142112168 3.6802010239
O -1.2189606491 -1.9007867489 3.3453878062
O -1.2445838561 -2.4357607004 4.7032265193
O -0.9039574498 0.7495399319 7.3761993736
C -3.4486588635 0.6667276337 6.3969779335
C 1.2021379521 0.0292558603 -2.2013990607
F 0.0232689417 -0.3563788393 -2.7059835785
F 1.4432566157 1.2753018817 -2.6413261513
F 2.1498311403 -0.7676448381 -2.7099917657
H 3.3843049585 -0.0331498134 1.9080872462
H 3.3557814422 -0.0500086348 -0.5667468044
H -0.9384511305 -0.0013317363 -0.5405712528
H -4.3279037271 0.5336565994 5.7750182017
H -3.4737895062 -0.0107507311 7.2484770243
H -3.3886064117 1.6936674275 6.752998033
H 2.6813989838 1.4307539404 4.0974068733
H 3.0330592786 -0.2804601527 4.4410828129
H 1.85783063 -0.1369168675 6.5184906995
H 1.5404621299 1.5836312543 6.2234751852
H -1.9407701191 -3.1054026457 4.6136646439
H -2.0750977713 0.064405267 1.6512581795

37
1aC4aOOHN5Halt_fig36 1 1 -1360.3465242 0.2667601 0 B3LYP-D3/6-311+G** (with additional pc-3 on C of CF3 group)
C 0.0000417497 -0.0011574889 0.0007314433
C 0.0003963169 -0.0025091715 1.3958302959
C 1.2399131233 0.0000602112 2.0683525468
C 2.4454142796 -0.0229006322 1.3714475216
C 2.428211373 -0.029734396 -0.0136600381
C 1.2040700431 -0.0131051323 -0.6890504083
N 1.183918619 0.068371149 3.4730681852
C 0.0508843193 -0.03089345 4.1178935935
C -1.2362494494 -0.3820979843 3.476038582
N -1.1793825081 0.0467021443 2.1333448797
N 0.1426852714 0.2069267443 5.4230026714
C -0.9714608616 0.441357943 6.2614337356
N -2.2147763111 0.4328846733 5.6161333795
C -2.4177086313 0.2518323398 4.2543207928
C 2.2865158276 0.3803743847 4.4252819438
C 1.5546638787 0.4687025235 5.7984766677
O -3.445732573 0.4959138916 3.6812953056
O -1.4948456892 -1.7852147565 3.6939638535
O -0.3604549829 -2.5176591564 3.155254445
O -0.8135942495 0.6811209247 7.4255619396
C -3.3850142951 0.8091858283 6.442271179
C 1.2033483925 0.0246255015 -2.2007273204
F 0.0053060726 -0.2974676371 -2.7058375884
F 1.5137011498 1.2533732968 -2.6439089008
F 2.1059617657 -0.8255666354 -2.7068027821
H 3.3870437895 -0.0352085747 1.9050742367
H 3.3560711304 -0.0522991129 -0.5688184244
H -0.9381874339 0.0012291006 -0.5390328477
H -4.2733579373 0.4033031439 5.9674468867
H -3.2502565807 0.3908908628 7.4355265075
H -3.4653264588 1.8943229301 6.5072386508
H 2.7578188881 1.3173864949 4.1300732218
H 3.021346906 -0.4228907959 4.3995624098
H 1.8928523831 -0.2891047532 6.5023303757
H 1.6329484775 1.449528292 6.2639372531
H -0.809781945 -3.1283736656 2.5504499041
H -2.0681253635 0.1417524846 1.6572679391

37
1aC4OOHC4OH_fig36 1 1 -1360.333215 0.266068 0 B3LYP-D3/6-311+G** (with additional pc-3 on C of CF3 group)
C 0.0432089201 -0.1600435058 0.0877516079
N 0.0975988479 -0.0975708316 1.5665167427
C 1.3616121621 -0.0598216755 1.9842787455
N 2.2493517121 -0.1459183662 0.9984304609
C 1.5447767862 -0.3075706987 -0.3043039353
C 1.7603635861 0.0984597632 3.3302907802
N 3.0129851706 0.2063267267 3.6437288092
C 3.948923083 0.1302311285 2.6451256451
C 3.6012822663 -0.0736407388 1.2739061301
C 4.5858939227 -0.1736486021 0.2869701766
C 5.9114682911 -0.0639103748 0.6589198638
C 6.2732450141 0.1442614117 2.0050489126
C 5.3117545018 0.238507692 2.9855881536
C -0.9742436455 0.3514499348 2.3937124036
N -0.6145349548 0.574456536 3.707161032
C 0.6039874667 0.020355571 4.3100226904
C 7.741843543 0.2759636951 2.3405291076
F 8.2487591753 1.3958680109 1.8016835706
O -2.0521704876 0.5614048288 1.9000630347
C -1.666256614 1.0593738815 4.6162670991
O 0.9592553597 0.6316916614 5.4768654573
O 0.2451480924 -1.3682891818 4.4847695626
O 1.4266376906 -2.1228027889 4.8468258659
F 7.9501417176 0.3218177491 3.6583699304
F 8.4368341661 -0.7580641829 1.8443090571
H 4.3185678229 -0.3356838693 -0.7490529297
H 6.6853140172 -0.1414744392 -0.0947076312
H 5.5678396325 0.3899111284 4.0254186053
H -1.2855460336 1.8959923842 5.2005623049
H -2.4995423061 1.3979881329 4.0077961928
H -2.017135017 0.2573292378 5.2697169145
H 1.775446805 -1.2921728383 -0.7109852382
H 1.8795814355 0.4621292899 -0.9983421186
H -0.4077158858 0.7538363109 -0.2970691715
H -0.5549868797 -1.0111992385 -0.2300483701
H 1.2874588474 -2.2793423891 5.7933604552
H 0.3093546567 0.4373754304 6.1647129878

37
1aC4aOOHC4OHalt_fig36 1 1 -1360.332699 0.2662573 0 B3LYP-D3/6-311+G** (with additional pc-3 on C of CF3 group)
C 0.1283557405 -0.1981432516 -0.1182324797
C 0.0836047583 -0.043477506 1.2818717799
C 1.3157439136 0.081342426 1.9971855747
C 2.5423420958 0.058964023 1.3274578229
C 2.5463846535 -0.0944110157 -0.045212054
C 1.3421107382 -0.2246653492 -0.7663280663
N 1.2087663383 0.211276663 3.3678355407
C 0.0100607998 0.2348574648 3.9463839131
C -1.1780706987 0.1390110917 3.1883824886
N -1.1381694562 0.001576452 1.9003977209
N 0.0580492827 0.3291294108 5.2704181846
C -1.0717754179 -0.0150476347 6.0830424803
N -2.2366492664 -0.195516198 5.3791282335
C -2.4529837501 0.3055264776 4.0022003271
C 2.2702774041 0.3386670789 4.4061973082
C 1.4623748895 0.3067383108 5.7395783461
O -3.4999771996 -0.3274119553 3.4025921915
O -0.9039312171 -0.1843734452 7.2643957738
C -3.4068060022 -0.6529585445 6.1529883879
C 1.4212349231 -0.3910667348 -2.2673604865
F 0.2112915841 -0.4778939072 -2.8238248813
F 2.0617852181 0.6479226948 -2.8241500527
F 2.105138945 -1.5010444728 -2.5835112928
H 3.4727950796 0.1594425036 1.8707757917
H 3.4900346399 -0.1140447155 -0.5764476053
H -0.8077421942 -0.2907390842 -0.6520923271
H -3.9626071423 -1.3731424017 5.5572620703
H -3.0409466674 -1.132998796 7.055735118
H -4.0433875925 0.1890314806 6.4263795012
H 2.7974355569 1.2813208669 4.25897856
H 2.9712834334 -0.4898998612 4.3151377165
H 1.6357937964 -0.5993687529 6.3187463477
H 1.6625579992 1.1715159979 6.3683938157
O -2.5916349595 1.731937226 3.9962298947
O -3.8581213846 2.0275511462 4.6580543358
H -4.1469817738 2.7885436948 4.1308658197
H -4.3145518564 0.1425858898 3.6349653399

37
1aC10aOOHN5H_fig36 1 1 -1360.3330824 0.2671956 0 B3LYP-D3/6-311+G** (with additional pc-3 on C of CF3 group)
C 0.0218824497 -0.0208727428 0.0165934991
C 0.0728730839 -0.0548905292 1.4170281787
C 1.3112901908 -0.0084943355 2.1204926208
C 2.4937853678 0.1227006984 1.3729176485
C 2.4317749296 0.152270074 -0.0043260996
C 1.1998047993 0.071854545 -0.6873408996
N 1.2720043669 -0.0254132952 3.481650218
C 0.1297225786 -0.5723399492 4.1494340033
C -1.1048599168 -0.1160721615 3.4742964027
N -1.0848049327 0.0106861331 2.1780601826
N 0.2323497682 -0.0803745885 5.4790014571
C -0.8505542833 0.316912038 6.23340922
N -2.133933261 0.214405642 5.6068127078
C -2.3287128887 0.1824125524 4.2472029176
C 2.3418789294 0.294901974 4.4339233652
C 1.6003213106 0.3724183666 5.793614898
O -3.3819421371 0.3636812361 3.667499169
O -0.7388278646 0.7425787517 7.3534282664
C -3.307924699 0.4884442905 6.4567844832
C 1.2022806771 0.1036583499 -2.1970969664
F -0.0353617253 0.0060399502 -2.6956860424
F 1.7400027984 1.2471500656 -2.6459563302
F 1.9306469613 -0.9054388788 -2.6938036627
H 3.4481430894 0.1710253486 1.879449962
H 3.3488745369 0.2275299722 -0.5757239805
H -0.9308207433 -0.0640101707 -0.4967832902
H -3.1109204834 0.0948605891 7.4494684679
H -3.4859599777 1.5626521754 6.5229259267
H -4.1698384948 0.0031140295 6.0064889832
H 2.8123295901 1.2398114802 4.1617410249
H 3.0886513865 -0.5012097539 4.4361317287
H 2.0606569987 -0.2769817339 6.5357178389
H 1.5576440877 1.3860263813 6.1906608667
H -1.9780337762 0.2618326348 1.7413146385
O 0.0453040401 -1.9977462989 4.0426677759
O 1.2389561188 -2.5418175 4.6618565892

H 0.84659179 -3.1458352391 5.3115202491

37
1aC10aOOHC4OH_fig36 1 1 -1360.3318277 0.2667631 0 B3LYP-D3/6-311+G** (with additional pc-3 on C of CF3 group)
C -0.0395714914 -0.0638661465 -0.0409601552
N 0.029651767 -0.1913809081 1.425828734
C 1.3747768081 -0.1107543553 1.907516717
N 2.1616063191 -0.445198133 0.7643259793
C 1.4425923288 -0.1791315343 -0.4869948377
C 1.6594341537 -1.0589580996 3.0131009212
N 2.8311602786 -1.5942998137 3.1988290963
C 3.7772954506 -1.429398845 2.2386909186
C 3.4252188351 -0.9074998174 0.9409908768
C 4.3681636478 -0.970542662 -0.0998860901
C 5.6299679077 -1.4607328693 0.1540438423
C 5.9964595472 -1.9313532448 1.4373929224
C 5.0816552867 -1.9312605475 2.4576688214
C -1.0202228506 -0.5805590143 2.1894875301
N -0.6710894703 -1.0154029556 3.5499381238
C 0.5664359063 -1.3699392688 3.8776842334
C 7.3976570274 -2.4505365538 1.6390972884
F 8.3054935467 -1.5040032166 1.3525214001
O 1.7045631429 1.1635508097 2.464859237
O 1.5241817267 2.1499901474 1.4066220348
O -2.16670298 -0.6267096319 1.8349825199
C -1.8019632929 -1.3119707223 4.4549500007
O 0.7552184779 -2.0196016005 4.9960314995
F 7.6058337029 -2.8527856814 2.897265719
F 7.6417649452 -3.4929708064 0.827735303
H 4.1137665804 -0.6027229741 -1.0848778604
H 6.3623439674 -1.4793457225 -0.6440752065
H 5.3262079994 -2.3162742341 3.4383988797
H -1.4963307054 -1.0975502662 5.4758948505
H -2.635334477 -0.6838041826 4.1571227948
H -2.088262385 -2.3600566278 4.3624337035
H 1.5992392291 -1.0023279944 -1.1844968061
H 1.7925242698 0.7524178653 -0.9344301646
H -0.4690544168 0.8962129578 -0.3209264178
H -0.661596527 -0.8639726931 -0.4402812725
H 1.0832869426 2.8562501454 1.9024889648
H 1.7075067986 -2.2180693249 5.0868675025

37
1aC2OOHC2OH_fig36 1 1 -1360.3274696 0.2662124 0 B3LYP-D3/6-311+G** (with additional pc-3 on C of CF3 group)
C -0.0414169146 -0.0231690724 0.0300123369
C -0.0204342677 0.0420733211 1.4387715902
C 1.2442199032 0.0596533973 2.1051448946
C 2.4391937448 0.0167746364 1.3828123812
C 2.3789502358 -0.0446769687 0.0036271445
C 1.1417293946 -0.065594102 -0.6713174426
N 1.1981134639 0.113748633 3.484104705
C 0.0283646689 0.1871920229 4.1180605446
C -1.1968740848 0.1544821085 3.4107749864
N -1.2087500623 0.0803828898 2.1117883603
N 0.1499128305 0.2864810229 5.4379014782
C -0.9820863678 -0.0166443049 6.3131608298
N -2.2284199954 0.2345048959 5.6252492018
C -2.4422771436 0.1676538225 4.2418846217
C 2.3084945211 0.1705620037 4.4719015517
C 1.5608243964 0.0319365856 5.8260414021
O -3.5486278093 0.1433792332 3.766076258
O -0.8566433184 0.6697937254 7.4957221847
C -3.4547742379 0.0961036485 6.4421635447
C 1.1487761523 -0.152639313 -2.1810414727
F -0.0793672549 -0.0448069131 -2.6914681586
F 1.9071111997 0.8199366657 -2.7088027822
F 1.6639416028 -1.3252941964 -2.5823609643
H 3.3955153187 0.0351638697 1.8888293886
H 3.2977440193 -0.07494748 -0.5689763866
H -1.0027536236 -0.0367131933 -0.4656660261
H -3.9950743814 -0.8053749199 6.1522253065
H -3.1797858522 0.0422129538 7.4912349309
H -4.1013356496 0.954429287 6.266935362
H 2.8208626244 1.1283384632 4.3695035103
H 3.0106482703 -0.6417830713 4.293747165
H 1.6412972285 -0.9726629222 6.2421984576
H 1.8908403697 0.7591194462 6.5634381048
O -0.8616960436 -1.3641482243 6.7753591819
O -0.8237146166 -2.1867964908 5.5871634785
H -1.5157442336 -2.8345796248 5.7919967608
H -1.1665568947 1.5764987486 7.3723462984

37
1aC10aOOHC2OH_fig36 1 1 -1360.3135465 0.2658406 0 B3LYP-D3/6-311+G** (with additional pc-3 on C of CF3 group)
C -0.0146586568 -0.1273478721 -0.0461007156
N 0.0236390864 -0.304384578 1.4188655204
C 1.3813245672 -0.1921720772 1.9496587637
N 2.1780477032 -0.4086520312 0.8015689792
C 1.4850948153 -0.0935366621 -0.4433981172
C 1.6675714839 -1.1971312107 3.0232651495
N 2.8213685647 -1.7527217579 3.1382932938
C 3.7798774781 -1.5227379702 2.1836462637
C 3.4570304729 -0.8910298444 0.946897823
C 4.4115844209 -0.8372029696 -0.0755477683
C 5.675660161 -1.352929785 0.1480892954
C 6.0134954643 -1.9444498233 1.378455008
C 5.0720412444 -2.0432381848 2.3798705311
C -0.9652560316 -0.6596870378 2.2213990979
N -0.7544890295 -1.0820618325 3.4722618756
C 0.562386247 -1.5169267532 3.9470788834
C 7.4059358193 -2.4934309144 1.5578536516
F 8.3315606007 -1.5521304062 1.3060811498
O 1.5445430824 1.0634325385 2.5967217662
O 1.2052293748 2.1009427511 1.6216499708
O -2.2198823955 -0.6379419935 1.8160861853
C -1.904251456 -1.3068227754 4.3788280603
O 0.6471241566 -2.0795373753 4.9973787967
F 7.6103945589 -2.9480986571 2.7981485495
F 7.6357892676 -3.5084168735 0.7061765837
H 4.1752732582 -0.3731843698 -1.0243441317
H 6.4207815176 -1.2916494042 -0.6357034769
H 5.292244125 -2.5234499394 3.3237668658
H -2.5994300094 -0.476174851 4.2892329205
H -2.402883397 -2.2436092681 4.1315426435
H -1.5053096405 -1.3644377475 5.3866597387
H 1.7163054968 -0.8444731869 -1.1995173456
H 1.7608941167 0.8945185389 -0.8149377969
H -0.5061303026 0.81157213 -0.3046242485
H -0.5342754129 -0.9694737203 -0.5060142611
H 1.8809976439 2.7613227933 1.838209926
H -2.3440047553 -0.1411142048 0.995102875

37
1aN5OOHC4aH_fig36 1 1 -1360.2923915 0.2653619 0 B3LYP-D3/6-311+G** (with additional pc-3 on C of CF3 group)
C 0.1355563451 -0.4159434376 0.1916006127
N 0.1328639787 0.0621709598 1.596717023
C 1.343770012 0.4472567779 1.958965556
N 2.242325092 0.342511729 1.0078895856
C 1.6294230574 -0.2470912539 -0.2143691012
C 1.7023577734 0.8648782752 3.331892978
N 2.9817371262 0.2255802736 3.6856475082
C 3.9898476621 0.3688231472 2.6684209238
C 3.6217331506 0.4420452666 1.3144417506
C 4.5879137241 0.5613234747 0.3213571502
C 5.9290379255 0.5850592641 0.6854070426
C 6.297024511 0.4954831058 2.0282522279
C 5.3299349196 0.3921645144 3.0236379339
C -0.9332606169 -0.1545530986 2.5103849671
N -0.6576787556 0.1931628723 3.8295725395
C 0.5846448862 0.5803775475 4.3499894296
C 7.7640966189 0.4852368422 2.3951370619
F 8.4579295166 1.3480243295 1.6421243519
O 2.8469453334 -1.0132418573 4.1716374655
O 2.2873497291 -1.9234659742 3.0391287656
O -1.9684942727 -0.611848628 2.1137864072
C -1.7501600736 -0.0021096446 4.8104216302
O 0.7701023841 0.7500681011 5.520623173
F 7.9536342298 0.8085605421 3.6789889077
F 8.2903075286 -0.7350522405 2.199485027
H 4.3042043684 0.630994197 -0.7209319098
H 6.6902699759 0.6822589696 -0.0777606668
H 5.6030597055 0.3344064315 4.0688938179
H -1.6820572524 0.7851807225 5.5560822857
H -2.6958392322 0.0445174529 4.2798975349
H -1.6433400536 -0.9720886177 5.2959235544
H 2.1209831562 -1.1960196558 -0.4276727654
H 1.7549653368 0.4292235302 -1.0581783465
H -0.5370862355 0.1971474125 -0.4048153919
H -0.2030757844 -1.4499355714 0.1627094114
H 2.6162652004 -2.7799104315 3.353040491
H 1.8737129353 1.9482503997 3.3431995612

37
1aC10aOOHC4aH_fig36 1 1 -1360.2825675 0.2654144 0 B3LYP-D3/6-311+G** (with additional pc-3 on C of CF3 group)
C 0.0081793313 -0.2953423517 -0.0859840474
N 0.0401232164 -0.2794783812 1.3786296736
C 1.3088996382 -0.0187141172 1.9122012087
N 2.2179832018 -0.3554261313 0.8197602543
C 1.5102111356 -0.363141071 -0.4763153387
C 1.6067075505 -0.8797140421 3.1054638161
N 2.9816043844 -0.7845167134 3.5140955586
C 3.8471292482 -0.602921651 2.5754647717
C 3.4880515931 -0.5337899632 1.114601746
C 4.5094308657 -0.6960772547 0.1459425678
C 5.809123149 -0.7532555332 0.5574350859
C 6.1923307449 -0.6890707742 1.9487493072
C 5.2596287639 -0.6129133121 2.9199732014
C -1.0585869491 -0.4410234847 2.2095999369
N -0.7542824475 -0.4832171104 3.5960714418
C 0.5152344675 -0.6215019164 4.1618087231
C 7.6710451724 -0.7323438824 2.2621114418
F 8.2971610562 0.2943589245 1.6685407704
O 1.5401730628 1.3132517868 2.346262538
O 1.363333102 2.1702554896 1.1901470945
O -2.1776676333 -0.5277005222 1.7723649286
C -1.884911382 -0.404881233 4.5440828766
O 0.7229130168 -0.6228371458 5.342059001
F 7.9050595533 -0.6698860661 3.569958696
F 8.2111603004 -1.8644718987 1.7873560592
H 4.2615911128 -0.7144944459 -0.9068514052
H 6.5943089158 -0.8350861771 -0.1857856847
H 5.5089048265 -0.5981874776 3.972869599
H -1.8173795016 0.5184288361 5.1185915179
H -2.8047249778 -0.4293552118 3.9690425659
H -1.8412749295 -1.2524192088 5.225875125
H 1.7594927143 -1.2768348422 -1.0163656386
H 1.8190657843 0.5049810387 -1.0597750794
H -0.4612059154 0.6054623731 -0.4804276892
H -0.5476608183 -1.1645889434 -0.4351067946
H 0.7757788076 2.8485179886 1.5585882934
H 1.4479038509 -1.9294070693 2.8105858457

37
1aC10aOOHC4aHalt_fig36 1 1 -1360.2842331 0.265882 0 B3LYP-D3/6-311+G** (with additional pc-3 on C of CF3 group)
C -0.0164421504 0.0772188577 0.024133686
N 0.0084726523 0.2869034828 1.4826648227
C 1.2908823947 0.6964424444 1.9235549249
N 2.1792084755 0.0478045199 0.9324766949
C 1.4631457791 -0.1820278343 -0.3368951781
C 1.6588307322 0.3714073264 3.3718943283
N 3.0288884441 0.0422940932 3.6110797202
C 3.84202914 -0.1846204405 2.6455417002
C 3.4163901646 -0.277976368 1.2192974093
C 4.3318940467 -0.7875771957 0.2591094462
C 5.6054836881 -1.0825324704 0.6433368046
C 6.0652889102 -0.9286875945 2.004757549
C 5.22608093 -0.5019875596 2.9686138955
C -0.9323315488 -0.3850969753 2.263334116
N -0.5817828182 -0.6498429908 3.5968112412
C 0.712112848 -0.62345964 4.0945166232
C 7.5079750629 -1.2765106591 2.2934342922
F 8.3229242514 -0.5064801318 1.5574303196
O 1.5266640781 2.0977410945 1.8937930909
O 1.3340010464 2.5903686548 0.5420223223
O -1.9813595033 -0.7460478385 1.793320377
C -1.5930675094 -1.3656568129 4.4059003758
O 1.0620168748 -1.2170554503 5.0757498342
F 7.8142480012 -1.1077764049 3.5768854894
F 7.7502630747 -2.5532809021 1.962677723
H 4.0306829651 -0.8968032486 -0.7733416778
H 6.310690477 -1.4402856426 -0.0984447973
H 5.5245883097 -0.3946139212 4.0029426932
H -2.5721201112 -0.9772916125 4.1422597955
H -1.5590749158 -2.4361178243 4.2025967828
H -1.3711729721 -1.1876309695 5.454214016
H 1.6360567704 -1.2095227996 -0.6585677327
H 1.8325045191 0.5115213862 -1.0925516701
H -0.4021915701 0.952818192 -0.4910488345
H -0.6563074517 -0.7745412598 -0.1946390076
H 0.7492383272 3.3452156753 0.7131838509
H 1.4820632469 1.3046928668 3.9278307173

37
1aN5OOHC2OH_fig36 1 1 -1360.2728644 0.2648599 0 B3LYP-D3/6-311+G** (with additional pc-3 on C of CF3 group)
C 0.0458180421 -0.2520832373 0.1201200519
N 0.0133540146 0.0246704714 1.5788723831
C 1.2931920717 -0.0535447674 2.1038542152
N 2.1898789374 -0.2410311911 1.1131812681
C 1.5537279269 -0.1094076462 -0.2051230122
C 1.5819191437 0.1261462258 3.4149362677
N 2.932263364 -0.0138118664 3.8242288005
C 3.8956582638 0.1635477832 2.769362919
C 3.5473545956 -0.0098727574 1.4155578734
C 4.5171926785 0.0917209807 0.4213633027
C 5.8333644735 0.3637039649 0.7748861817
C 6.1782478833 0.5460314482 2.1130758824
C 5.2096846641 0.4523396372 3.1078240795
C -1.0284274351 0.1924827082 2.4071227036
N -0.8379304168 0.369934609 3.7020493427
C 0.5221587454 0.3826376641 4.3519726467
C 7.6171630685 0.8008055236 2.487890598
F 8.2478729863 1.5243757274 1.5518615627
O 3.1209346617 -1.1867288501 4.5301600252
O 2.8554873749 -2.3639591184 3.6143477928
O -2.2691012937 0.204082442 1.9611572702
C -2.0040472089 0.5697750687 4.5903447561
O 0.5847984096 0.5818932806 5.5280424335
F 7.7174666307 1.4559692989 3.6515054394
F 8.2918517695 -0.3562426024 2.6190680663
H 4.2564446064 -0.0457417949 -0.6202340057
H 6.589484272 0.4435451153 0.0049721234
H 5.4614929199 0.5920489029 4.1507742067
H -2.5556090139 1.4563402131 4.2818125371
H -2.6496268106 -0.3063592666 4.553763419
H -1.606516413 0.7033549979 5.5920680319
H 1.887231275 -0.8924238767 -0.884590517
H 1.7819114118 0.8680986668 -0.6395259423
H -0.5546720462 0.4730197563 -0.4294962847
H -0.3139590857 -1.2656613923 -0.0664919272
H 3.7048728289 -2.8230232744 3.6965904174
H -2.3446323554 0.0405671684 1.0111743651

6 NMR spectra of the synthetized compounds

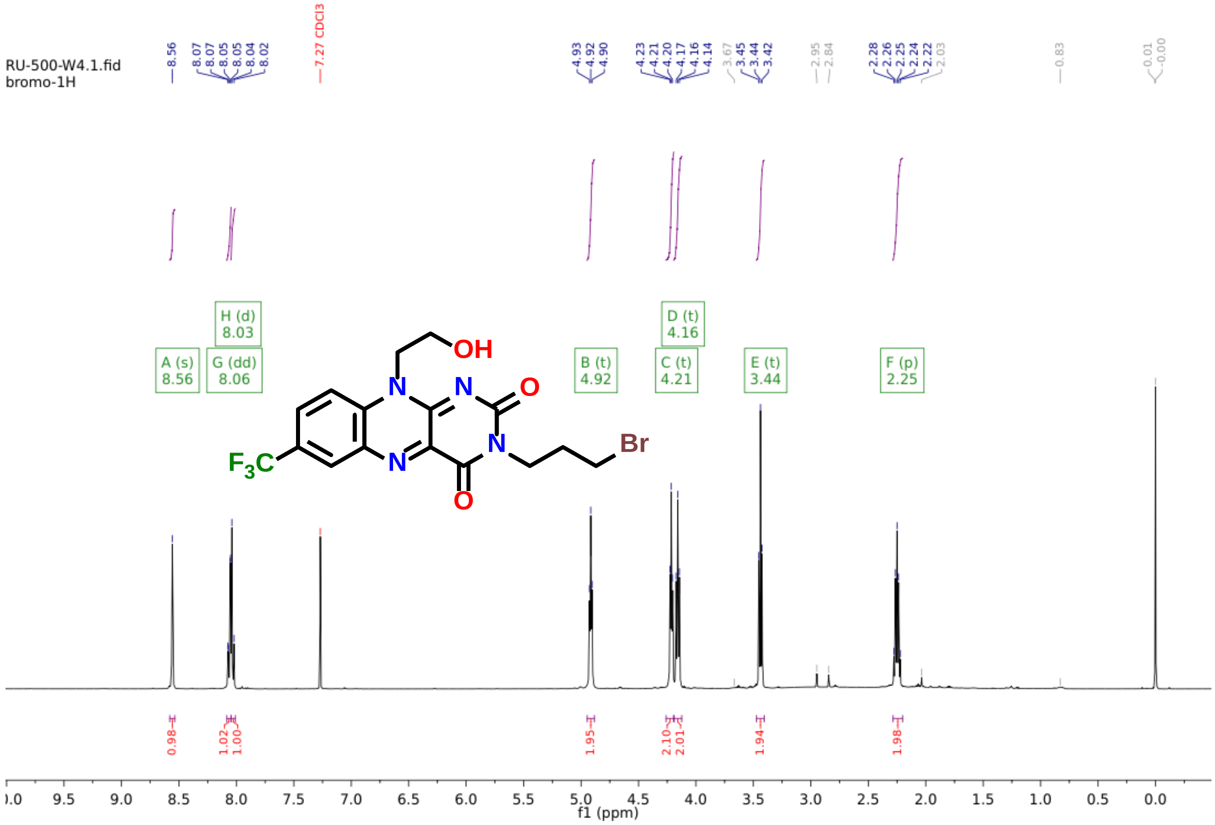
RU-500-W4.1.fid
bromo-1H
A (s)
8.56
H (d)
8.03
G (dd)
8.06
B (t)
4.92
D (t)
4.16
C (t)
4.21
E (t)
3.44
F (p)
2.25
f1 (ppm)

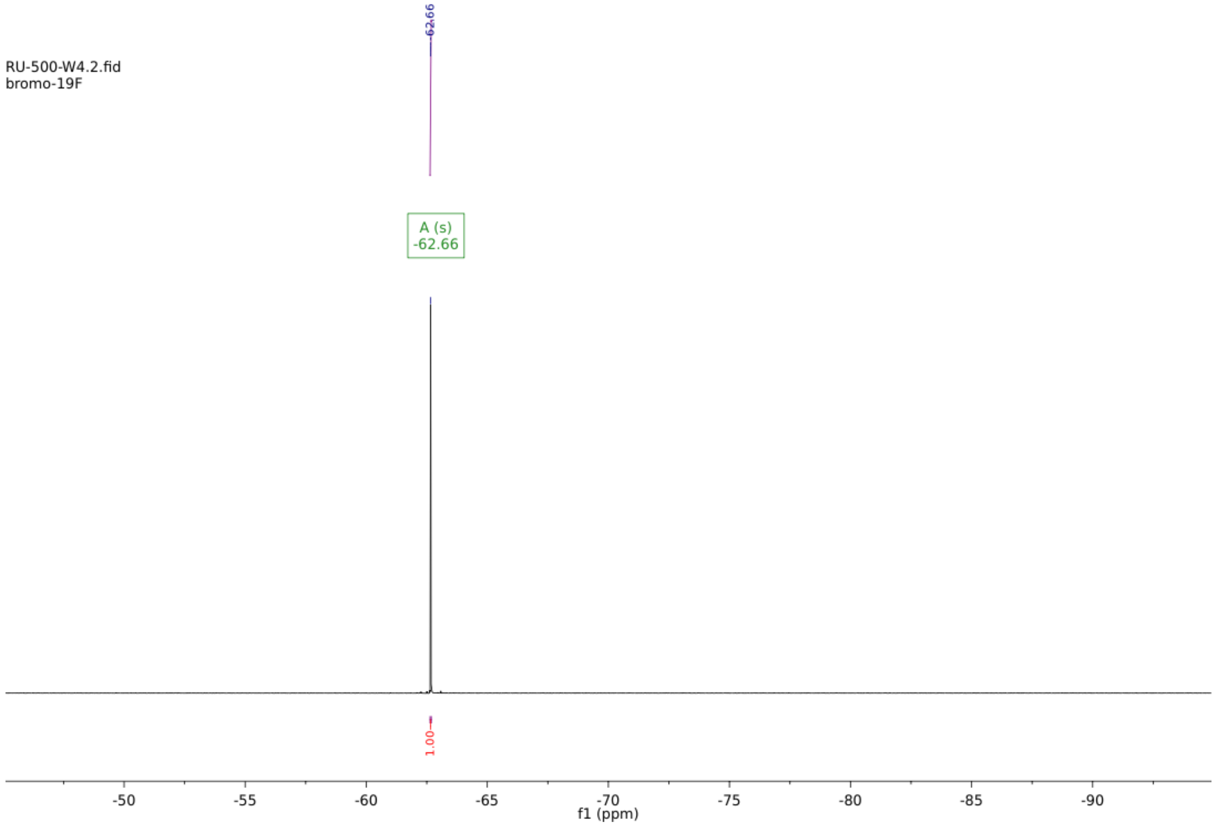
RU-500-W4.2.fid
bromo-19F
A (s)
-62.66
f1 (ppm)

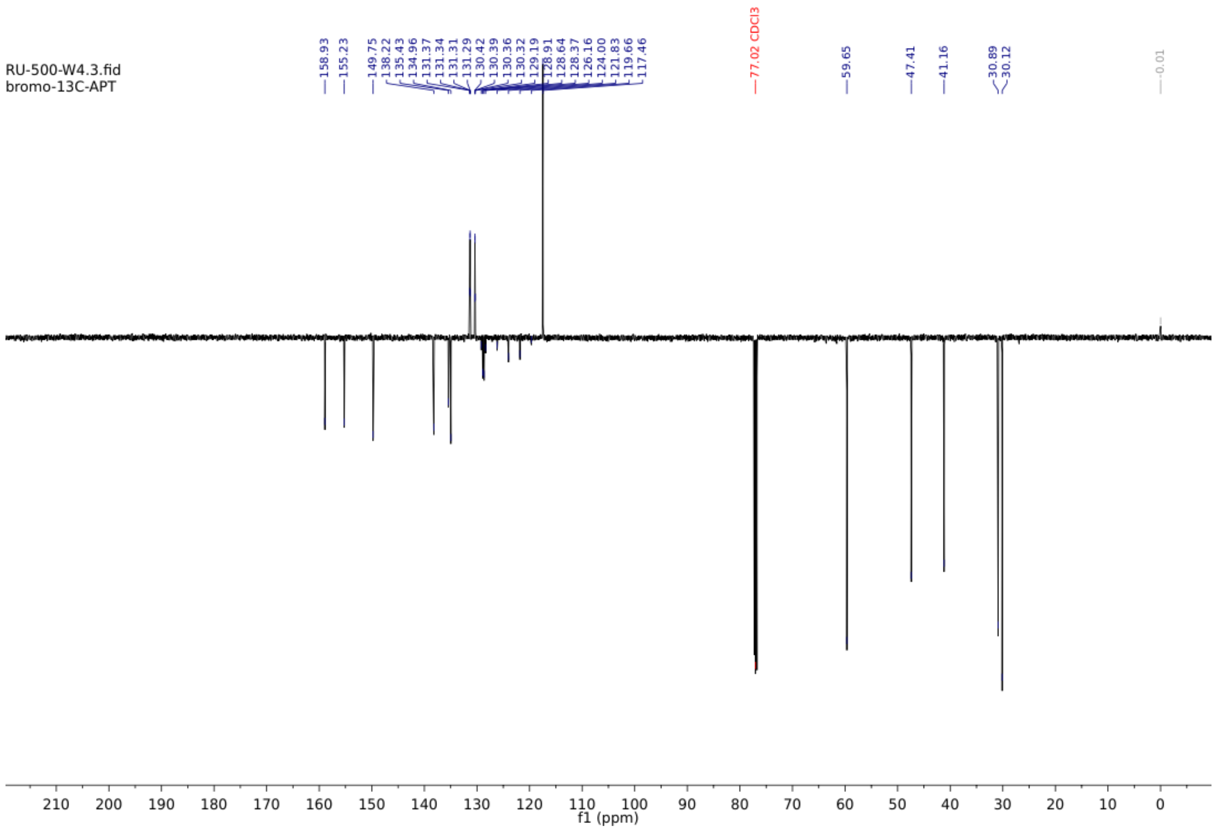
RU-500-W4.3.fid
bromo-13C-APT
158.93
155.23
149.75
138.22
135.43
134.96
131.37
131.34
131.31
131.29
130.42
130.39
130.36
130.32
129.19
128.91
128.64
128.37
126.16
124.00
121.83
119.66
117.46
77.02 CDCl3
59.65
47.41
41.16
30.89
30.12
0.01
f1 (ppm)

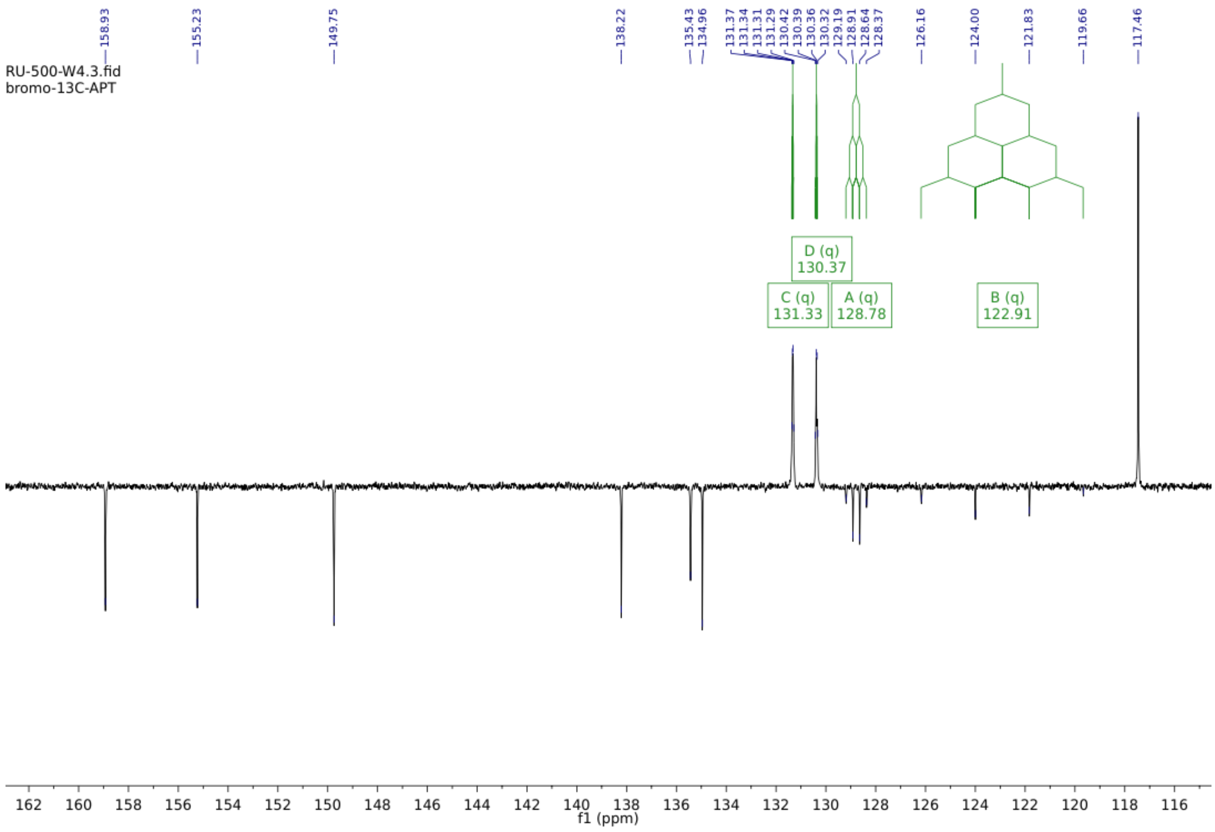
RU-500-W4.3.fid
bromo-13C-APT
158.93
155.23
149.75
138.22
135.43
134.96
131.37
131.34
131.31
131.29
130.42
130.39
130.36
130.32
129.19
128.91
128.64
128.37
126.16
124.00
121.83
119.66
117.46
D (q)
130.37
C (q)
131.33
A (q)
128.78
B (q)
122.91
f1 (ppm)

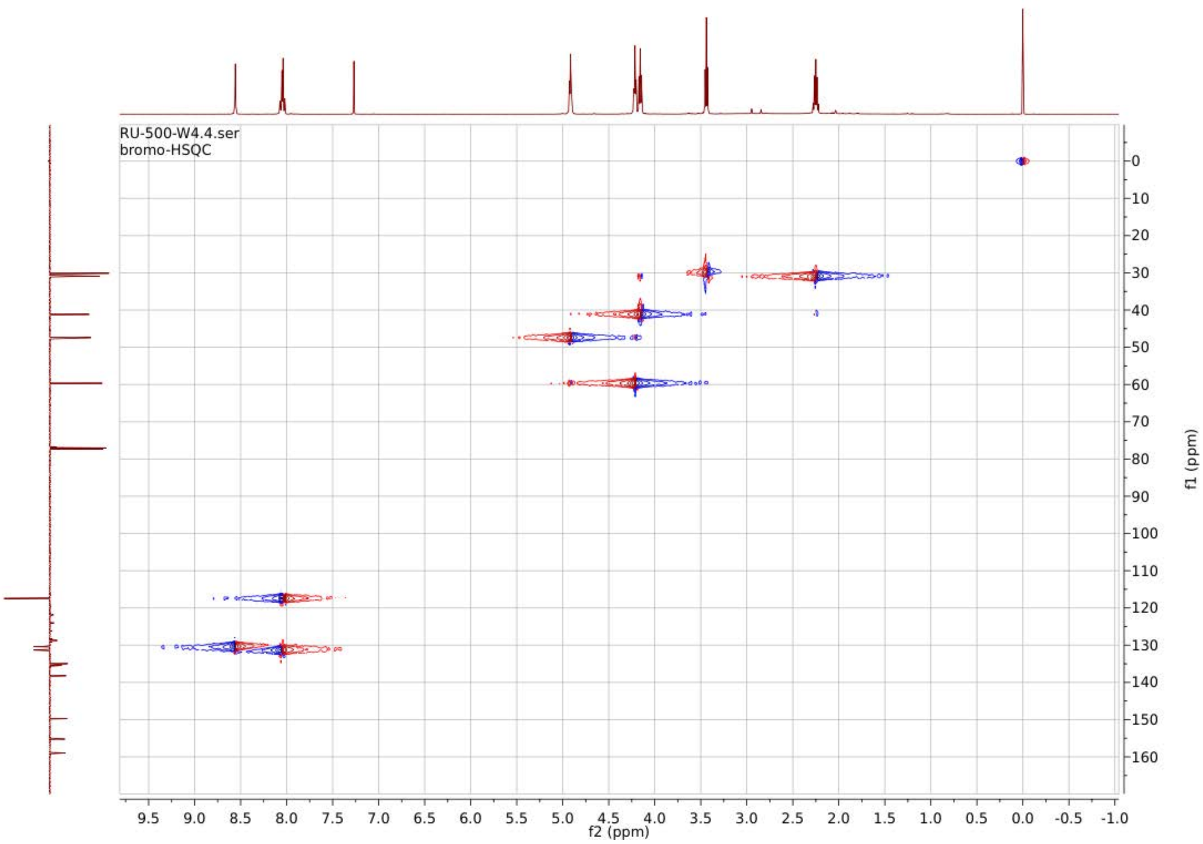
RU-500-W4.4.ser
bromo-HSQC
f1 (ppm)
f2 (ppm)

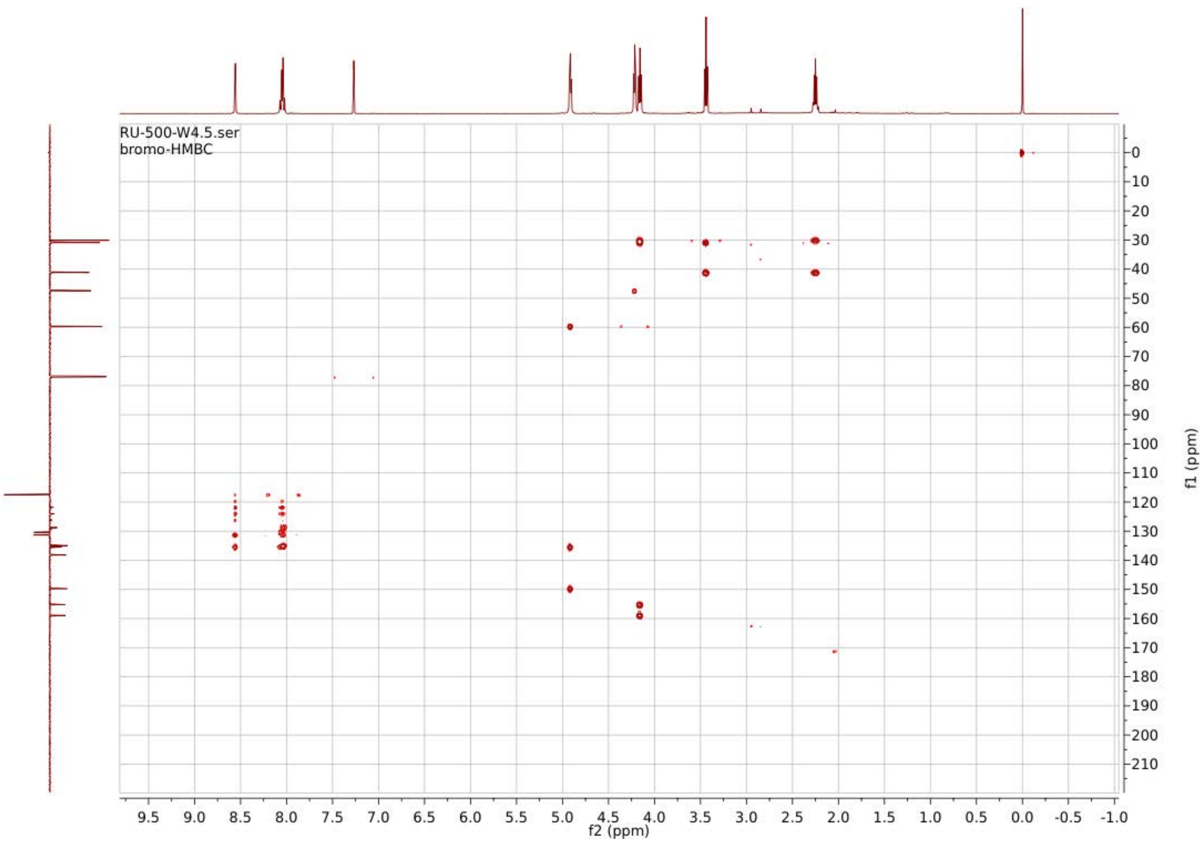
RU-500-W4.5.ser
bromo-HMBC
f1 (ppm)
f2 (ppm)

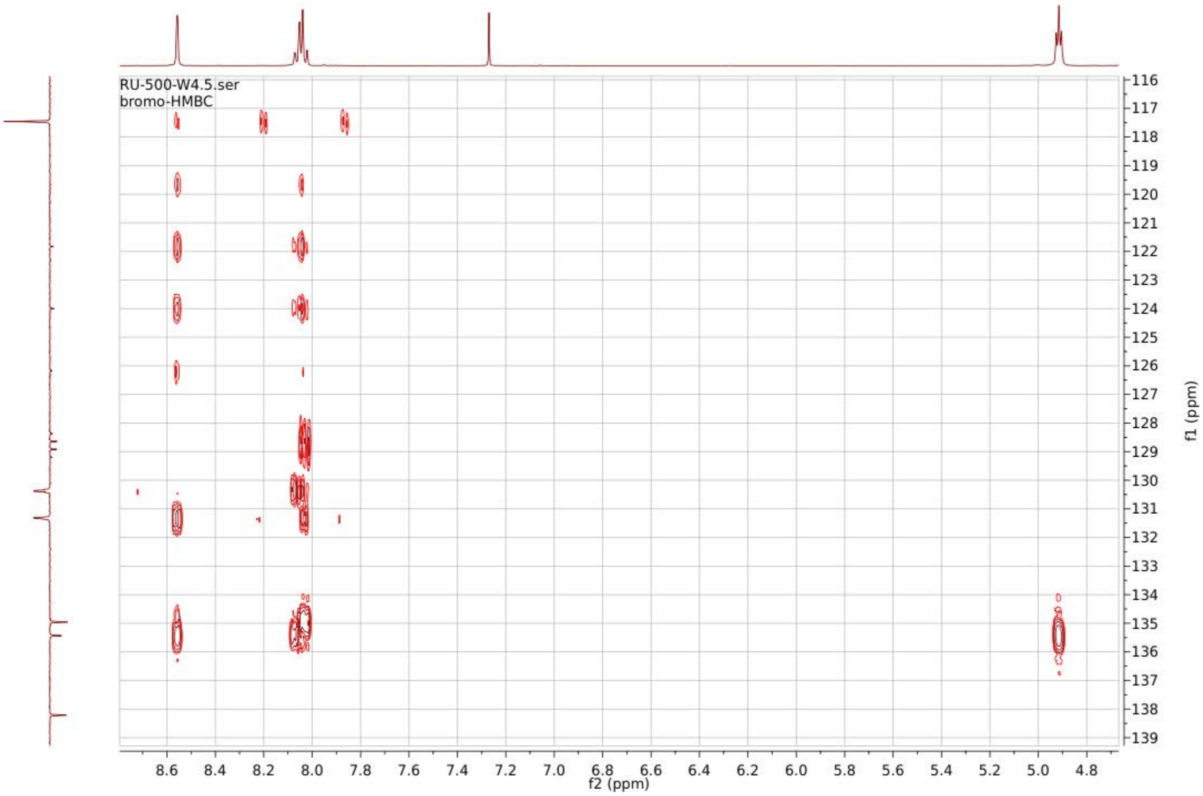
RU-500-W4.5.ser
bromo-HMBC
f2 (ppm)
f1 (ppm)

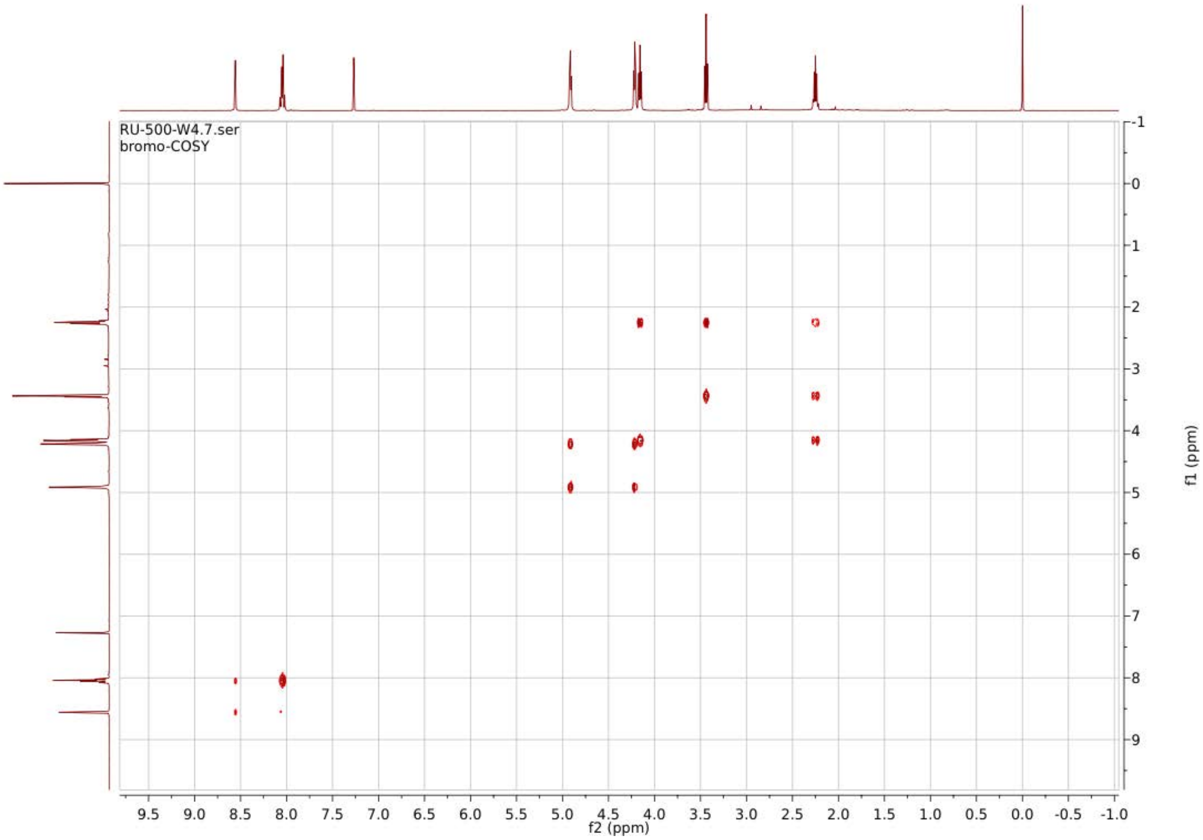
RU-500-W4.7.ser
bromo-COSY
f2 (ppm)
f1 (ppm)

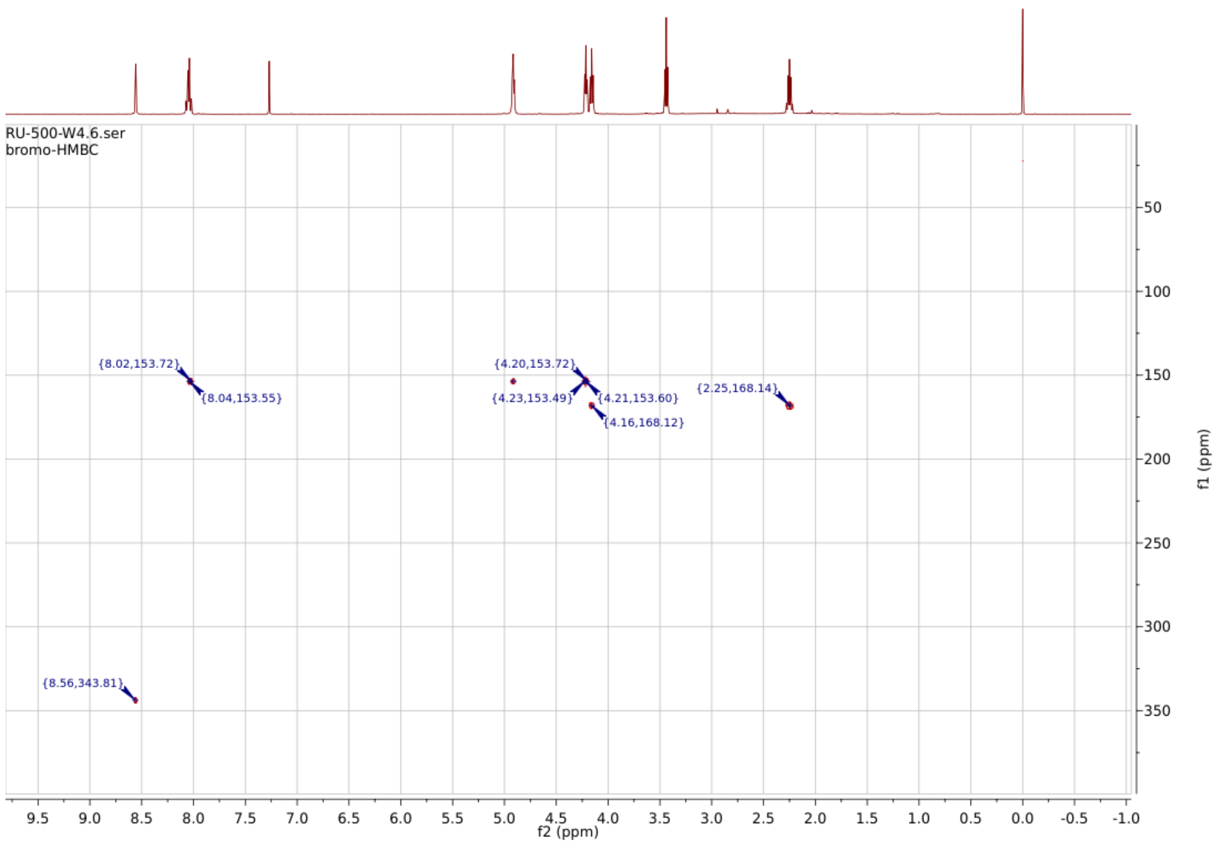
RU-500-W4.6.ser
bromo-HMBC
{8.02,153.72}
{8.04,153.55}
{4.20,153.72}
{4.23,153.49}
{4.21,153.60}
{4.16,168.12}
{2.25,168.14}
{8.56,343.81}
f2 (ppm)
f1 (ppm)

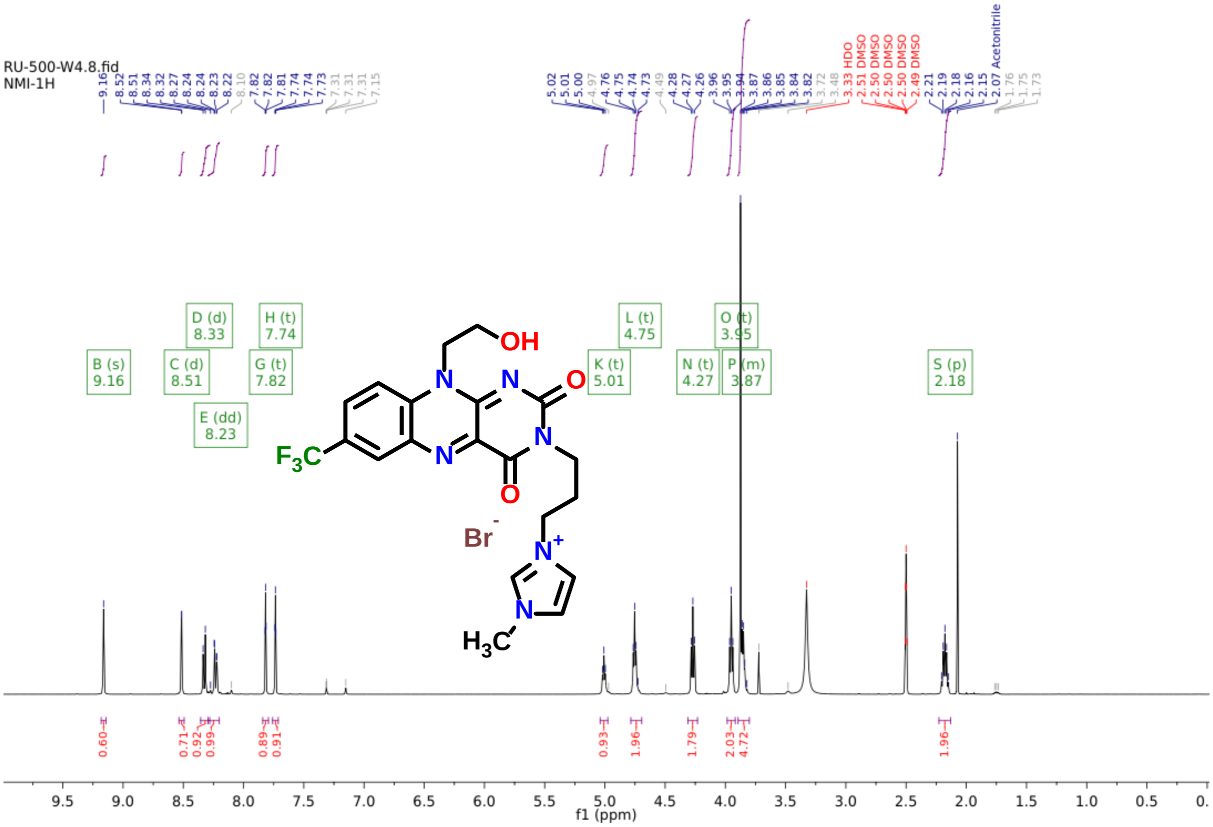
RU-500-W4.8.fid
NMI-1H
B (s) 9.16
C (d) 8.51
D (d) 8.33
E (dd) 8.23
G (t) 7.82
H (t) 7.74
K (t) 5.01
L (t) 4.75
N (t) 4.27
O (t) 3.95
P (m) 3.87
S (p) 2.18
OH
N
O
F3C
Br-
N+
H3C
f1 (ppm)

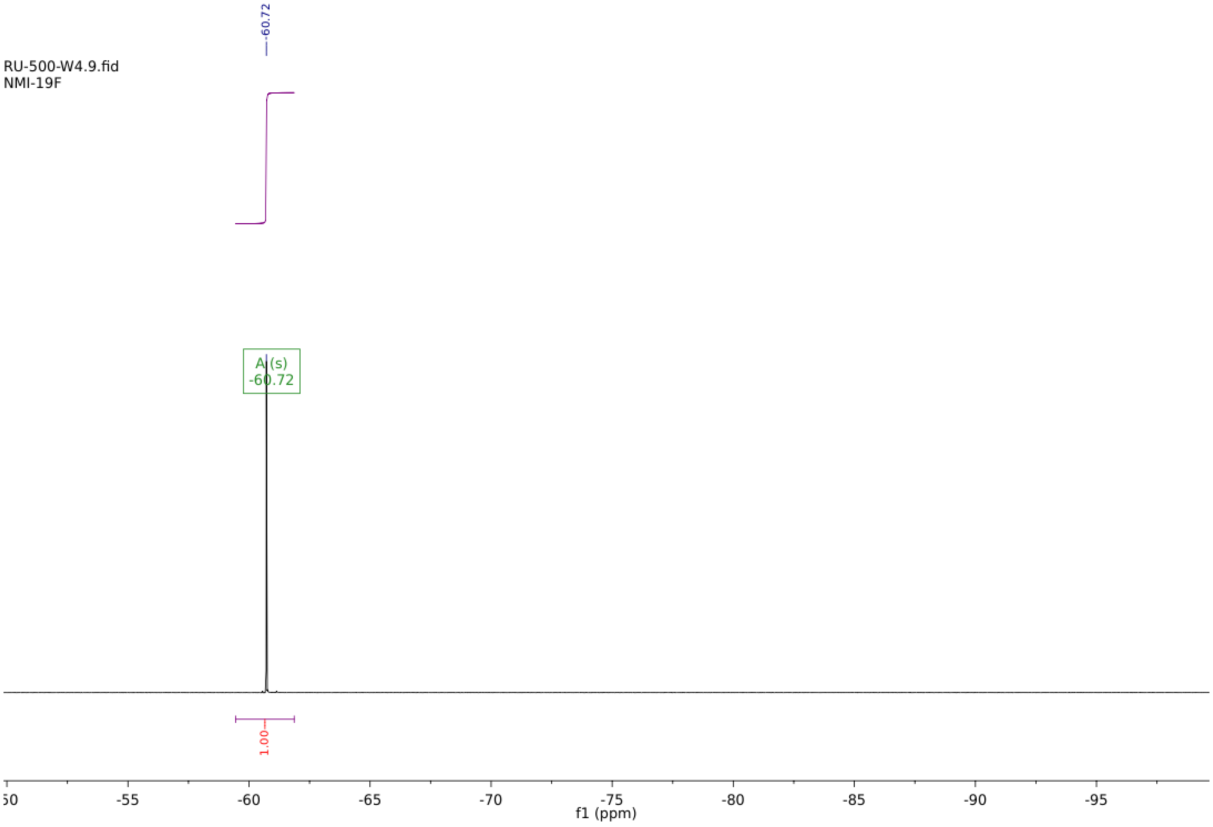
RU-500-W4.8.fid
NMI-19F
-60.72
A (s) -60.72
1.00
f1 (ppm)

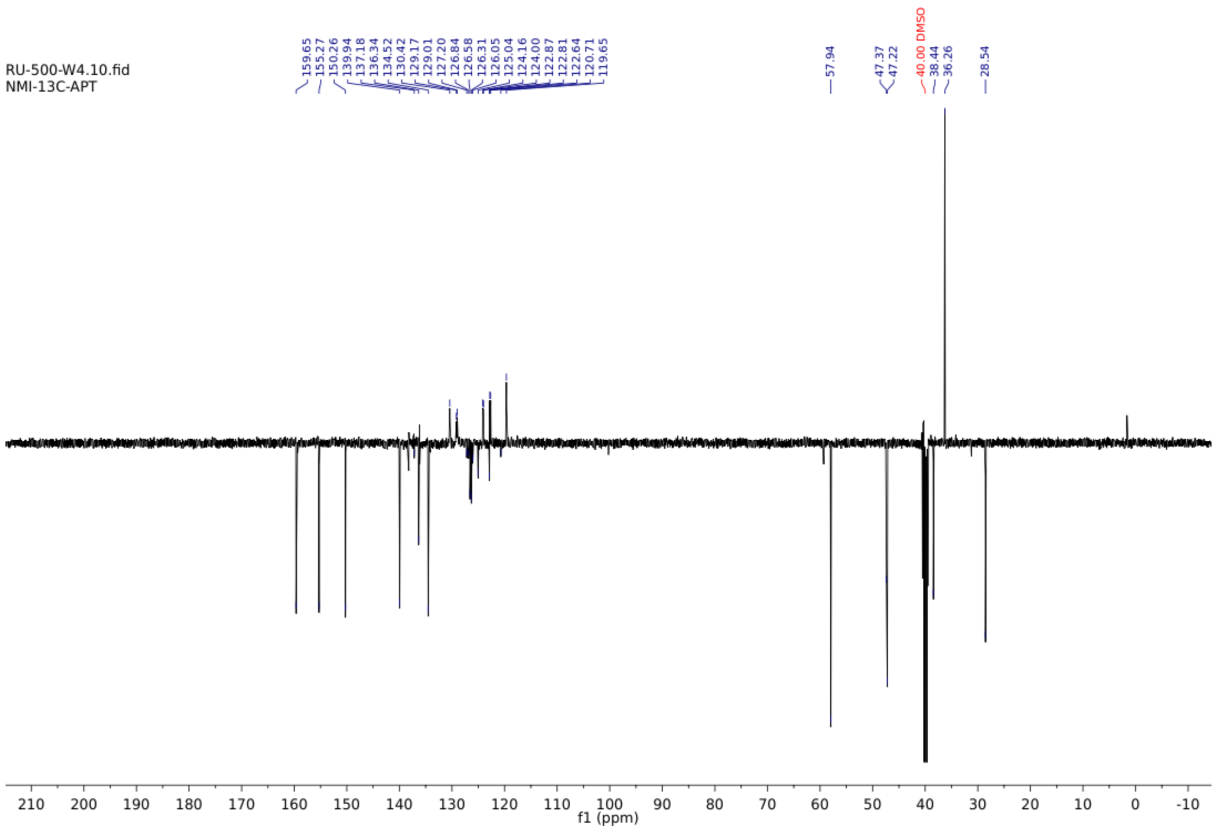
RU-500-W4.10.fid
NMI-13C-APT
159.65
155.27
150.26
139.94
137.18
136.34
134.52
130.42
129.17
129.01
127.20
126.84
126.58
126.31
126.05
125.04
124.16
124.00
122.87
122.81
122.64
120.71
119.65
57.94
47.37
47.22
40.00 DMSO
38.44
36.26
28.54
f1 (ppm)

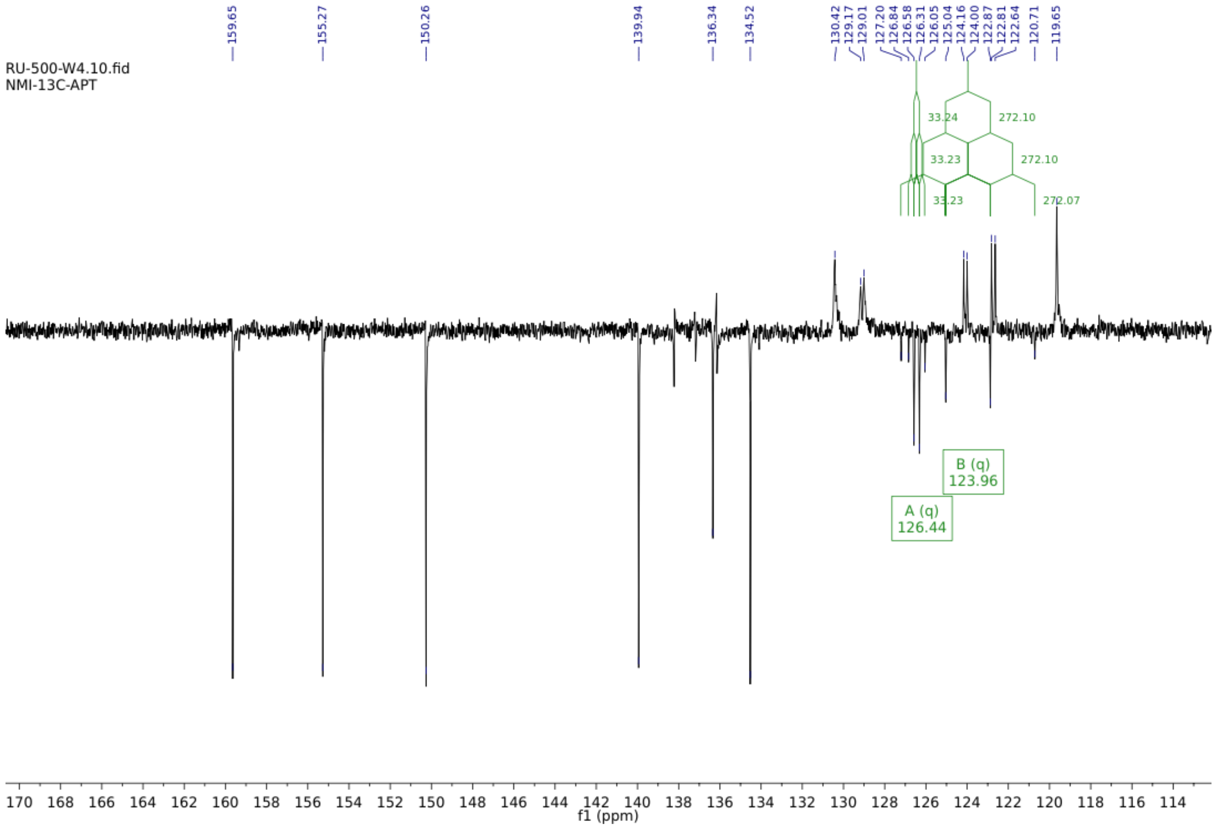
RU-500-W4.10.fid
NMI-13C-APT
159.65
155.27
150.26
139.94
136.34
134.52
130.42
129.17
129.01
127.20
126.84
126.58
126.31
126.05
125.04
124.16
124.00
122.87
122.81
122.64
120.71
119.65
33.24
33.23
33.23
272.10
272.10
272.07
B (q)
123.96
A (q)
126.44
f1 (ppm)

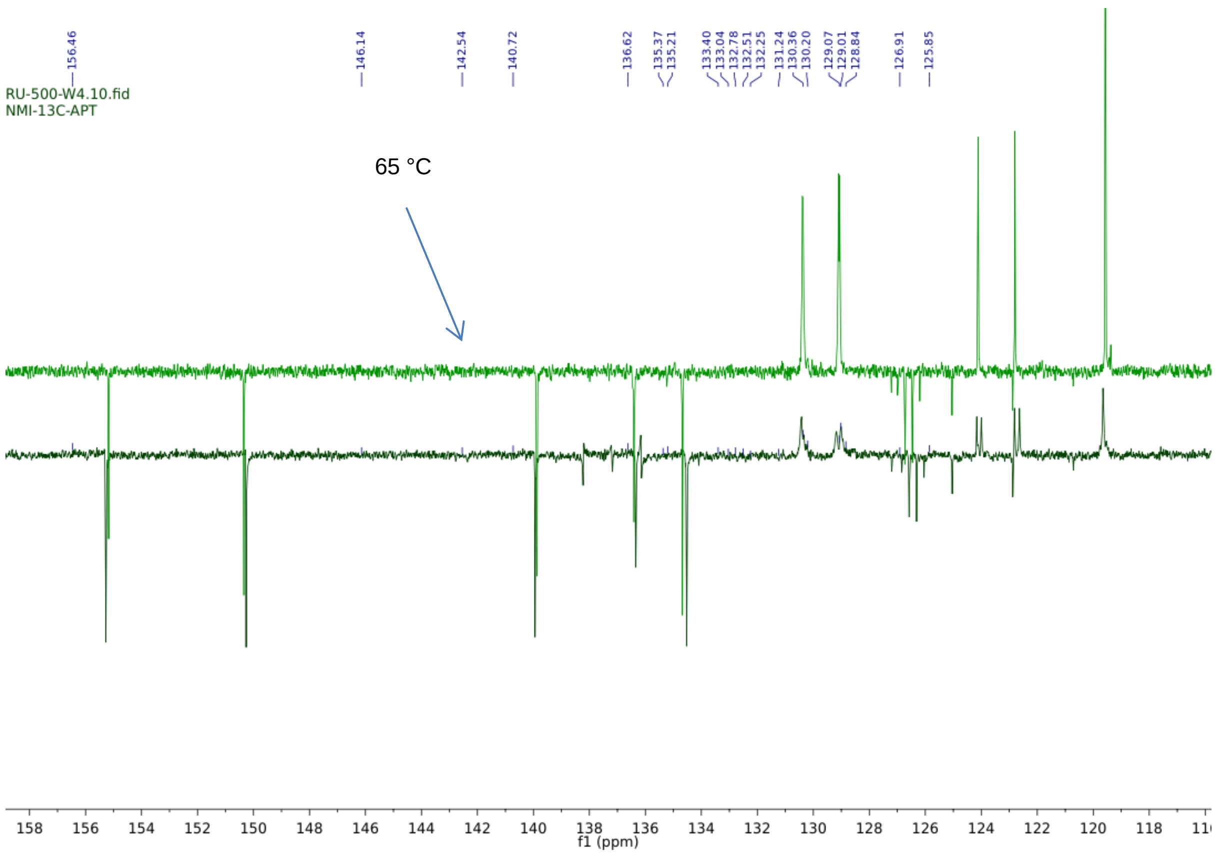
RU-500-W4.10.fid
NMI-13C-APT
65 °C
f1 (ppm)

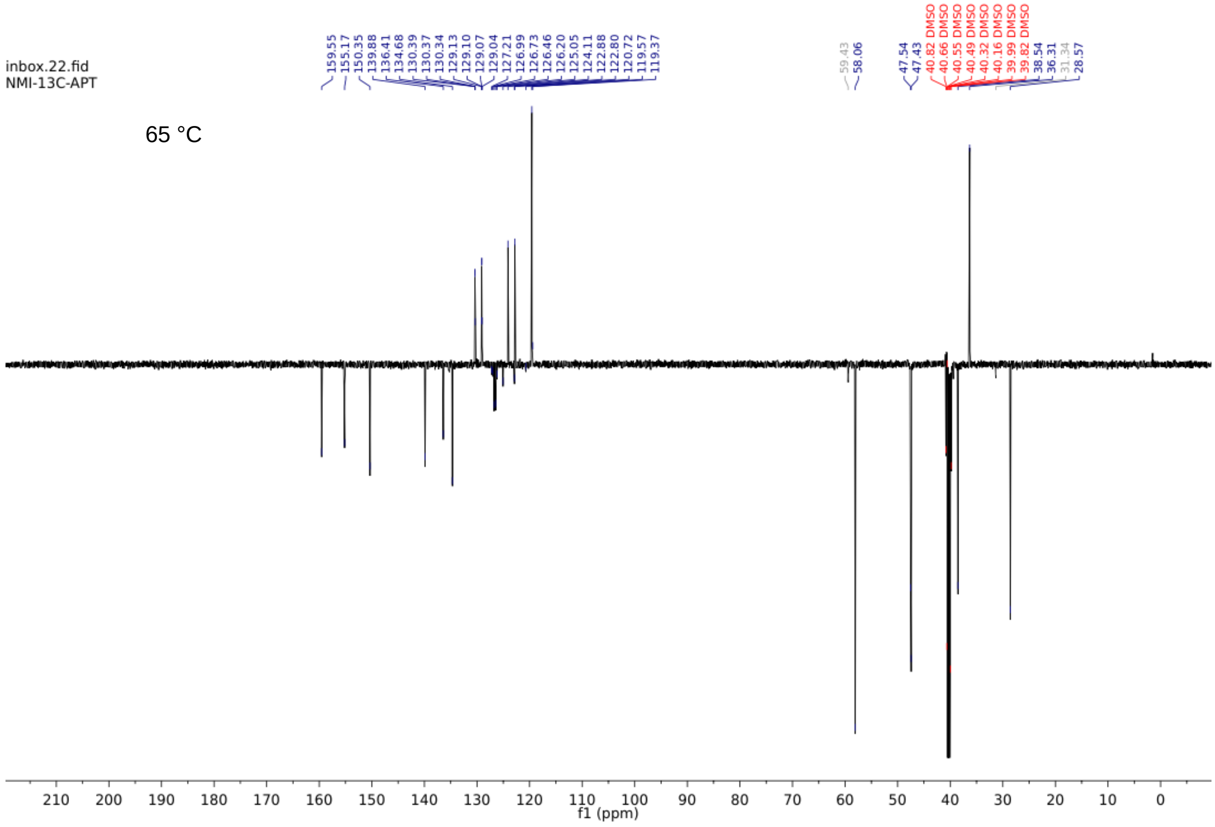
inbox.22.fid
NMI-13C-APT
65 °C
f1 (ppm)

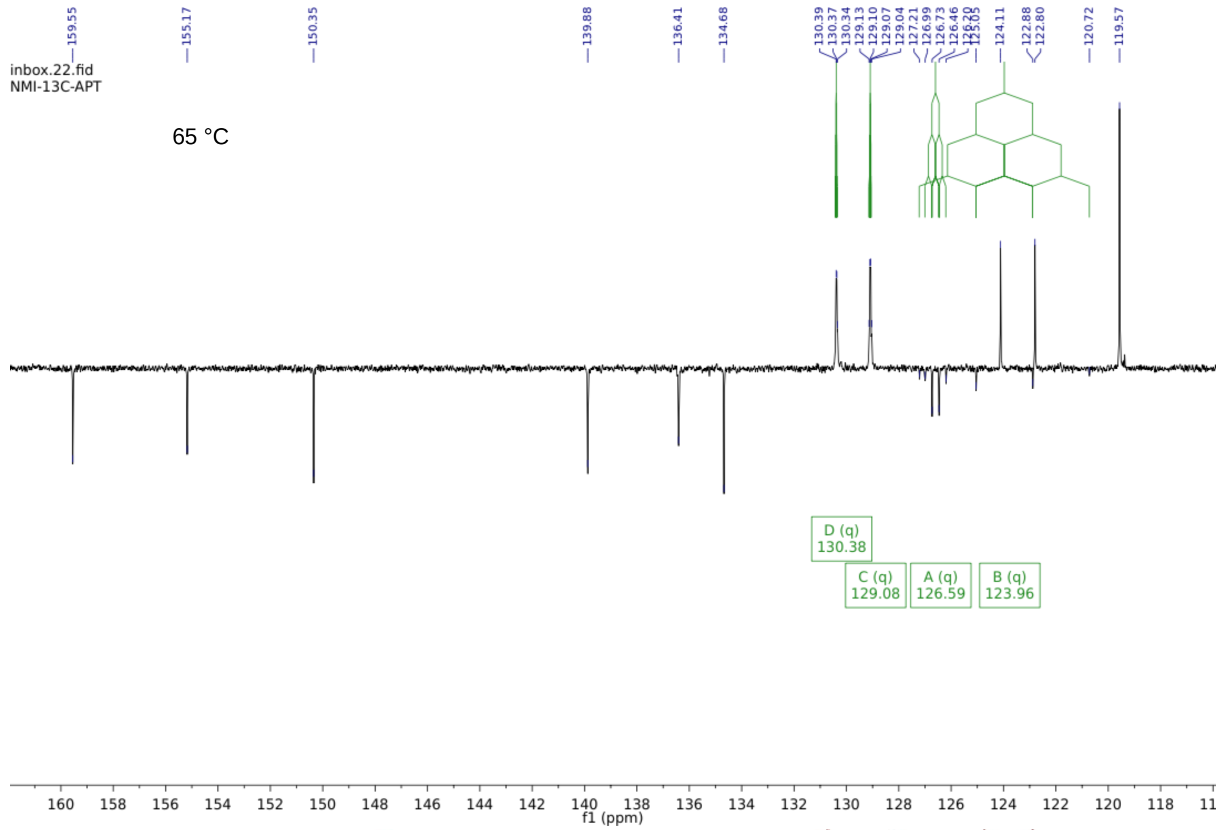

inbox.22.fid
NMI-13C-APT
65 °C
159.55
155.17
150.35
139.88
136.41
134.68
130.39
130.37
130.34
129.13
129.10
129.07
129.04
127.21
126.99
126.73
126.46
124.11
122.88
122.80
120.72
119.57
D (q)
130.38
C (q)
129.08
A (q)
126.59
B (q)
123.96
f1 (ppm)

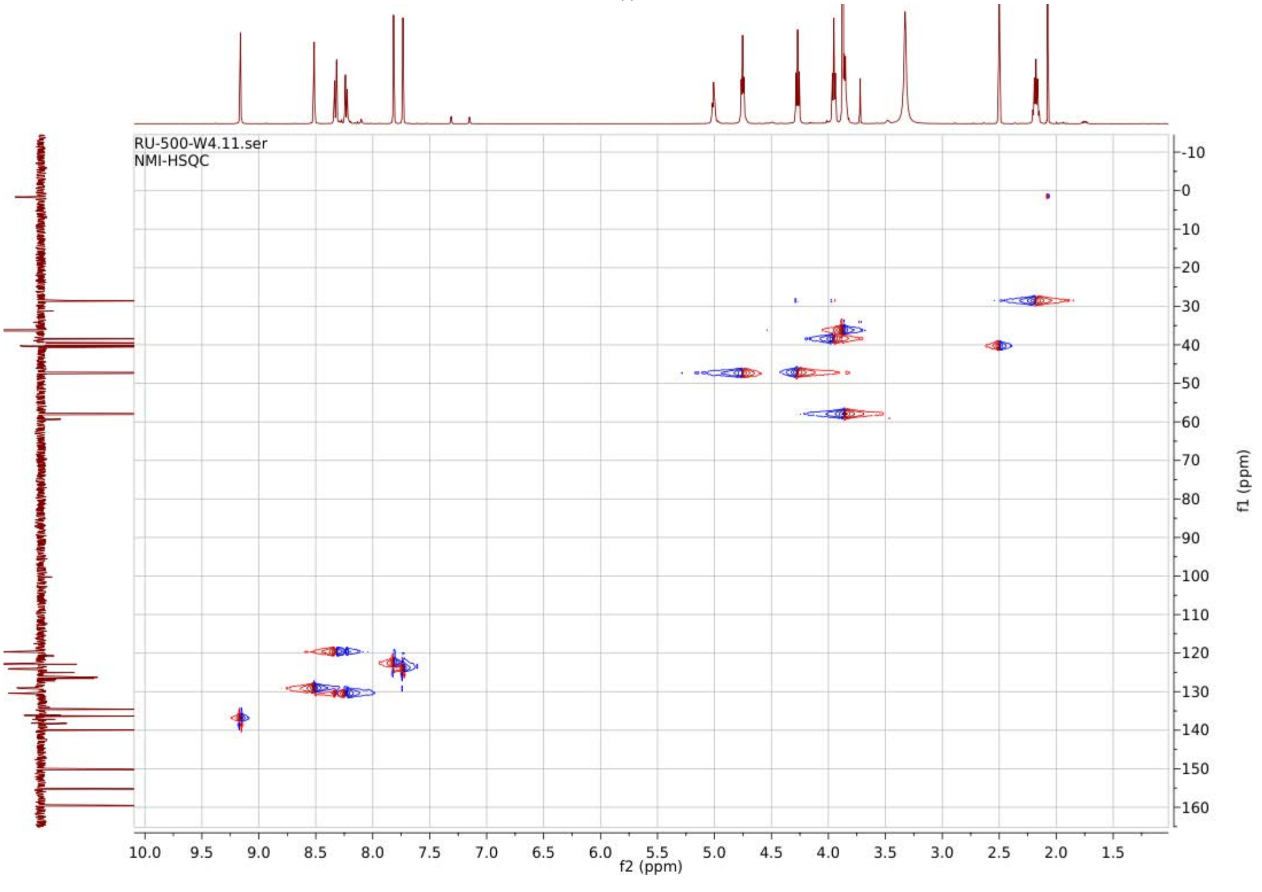

RU-500-W4.11.ser
NMI-HSQC
f2 (ppm)
f1 (ppm)

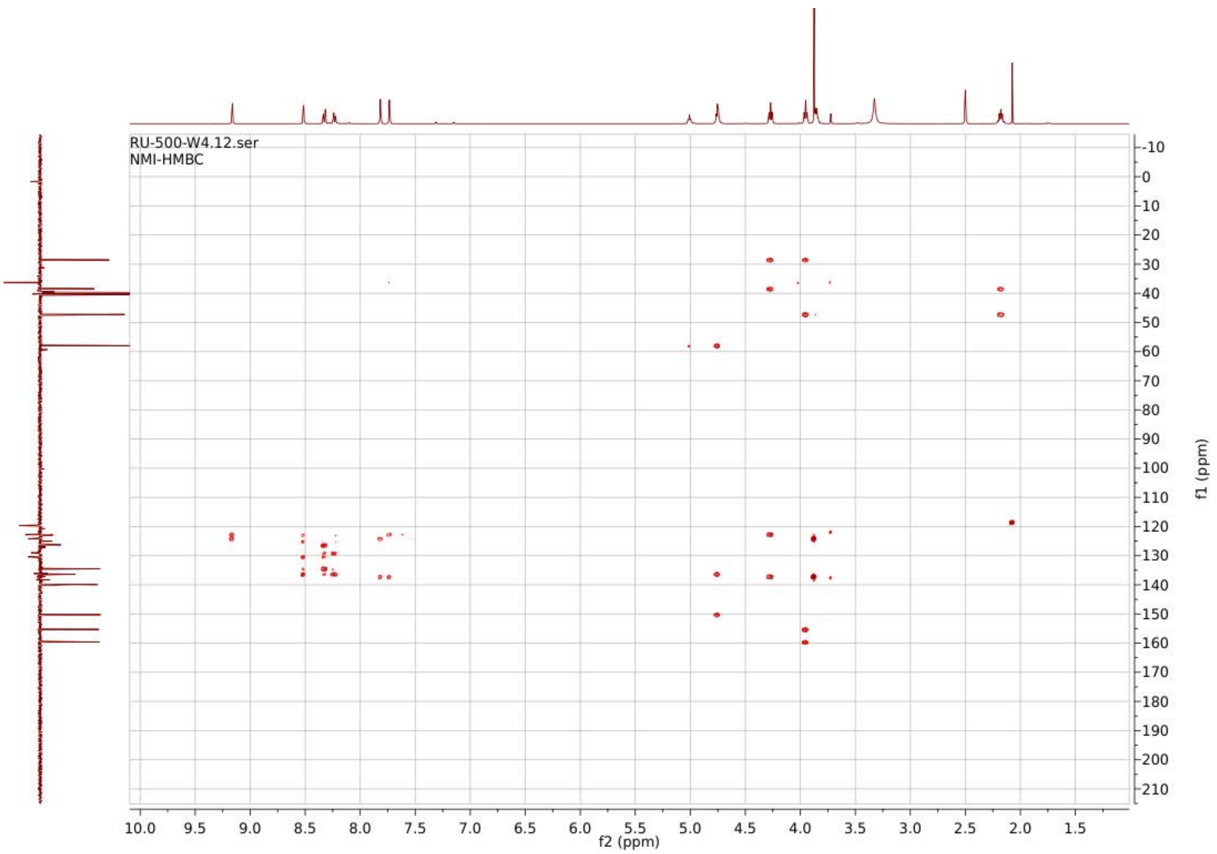
RU-500-W4.12.ser
NMI-HMBC
f2 (ppm)
f1 (ppm)

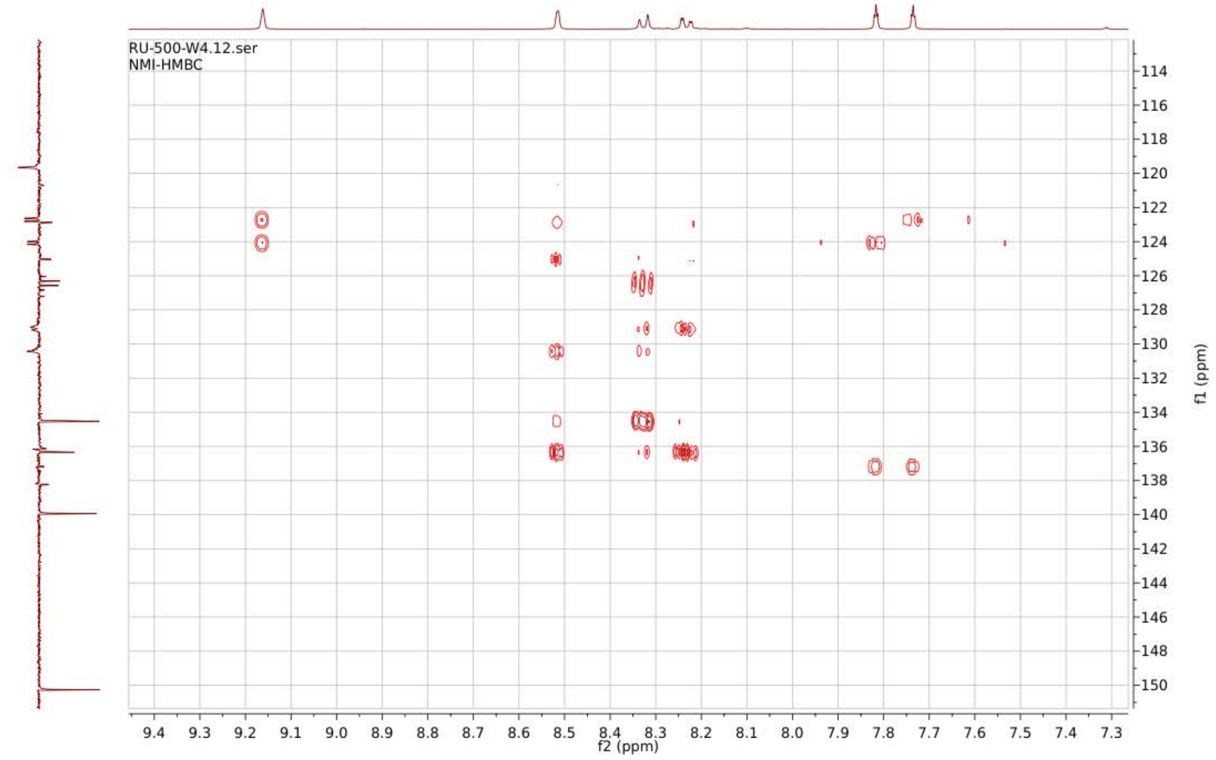
RU-500-W4.12.ser
NMI-HMBC
f2 (ppm)
f1 (ppm)

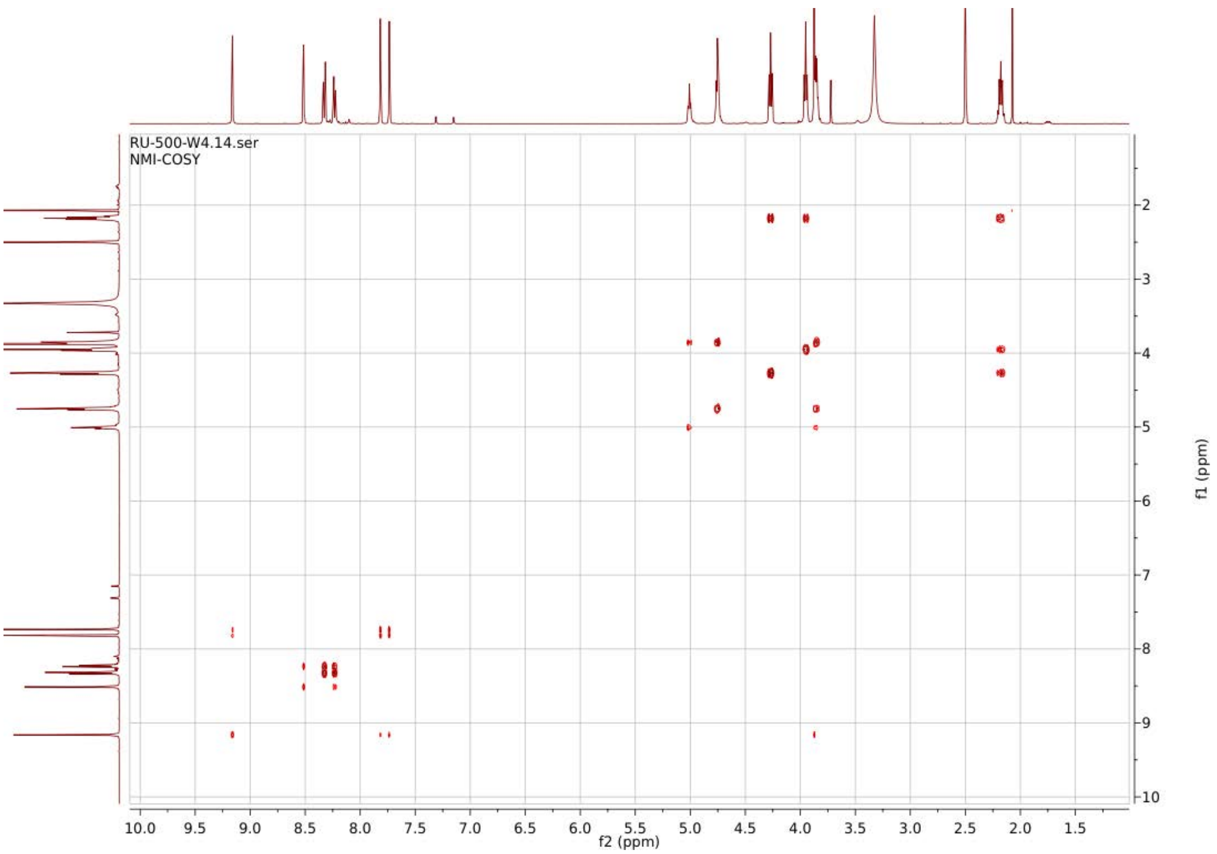
RU-500-W4.14.ser
NMI-COSY
f2 (ppm)
f1 (ppm)

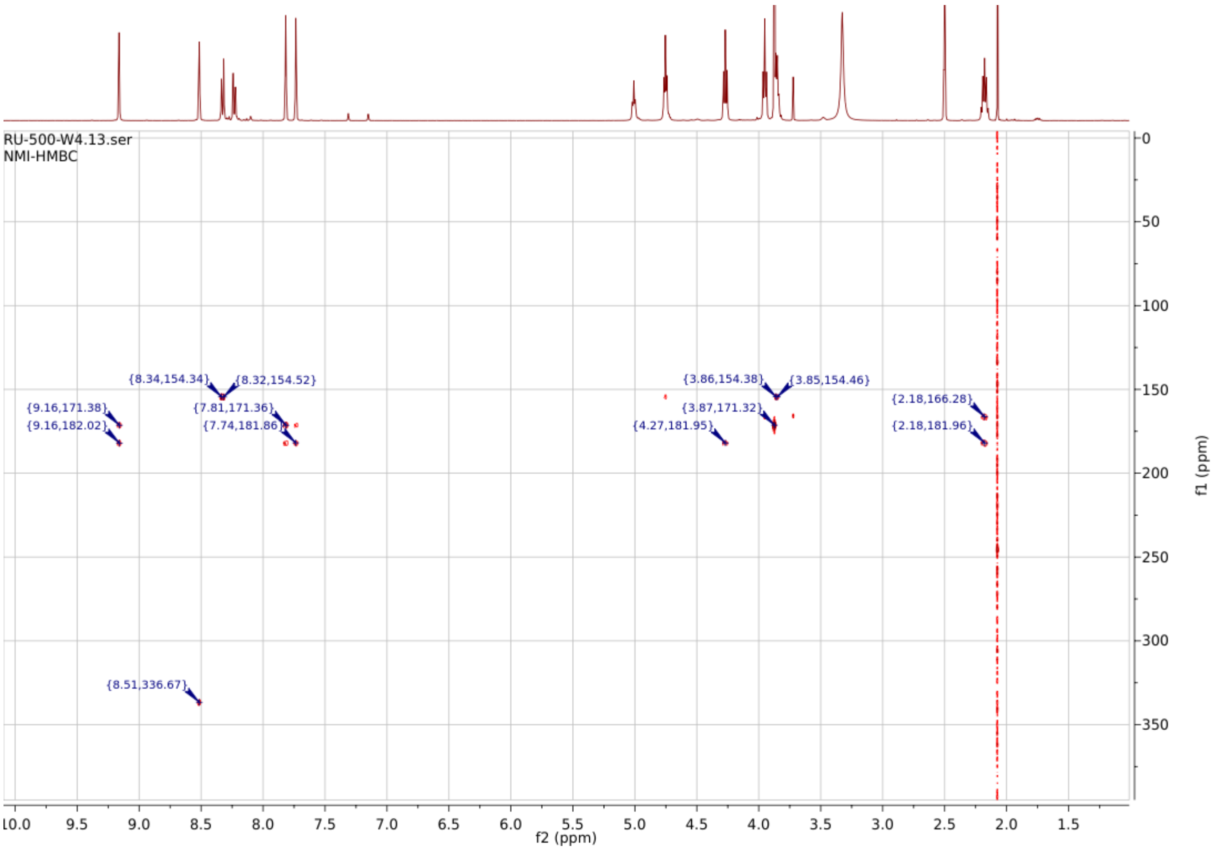
RU-500-W4.13.ser
NMI-HMBC
{8.34,154.34}
{8.32,154.52}
{9.16,171.38}
{9.16,182.02}
{7.81,171.36}
{7.74,181.86}
{3.86,154.38}
{3.85,154.46}
{3.87,171.32}
{4.27,181.95}
{2.18,166.28}
{2.18,181.96}
{8.51,336.67}
f2 (ppm)
f1 (ppm)

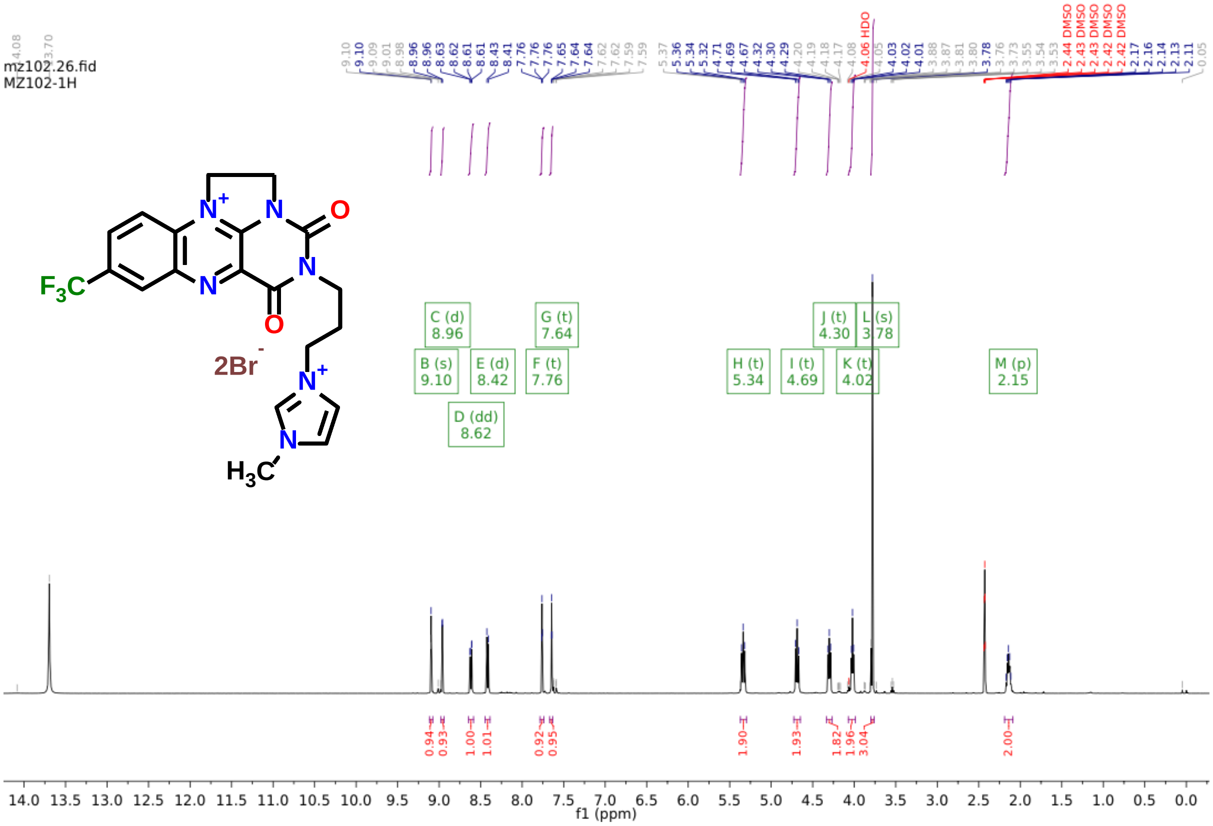
mz102.26.fid
MZ102-1H
2Br-
B (s) 9.10
C (d) 8.96
D (dd) 8.62
E (d) 8.42
F (t) 7.76
G (t) 7.64
H (t) 5.34
I (t) 4.69
J (t) 4.30
K (t) 4.02
L (s) 3.78
M (p) 2.15
4.06 HDO
f1 (ppm)

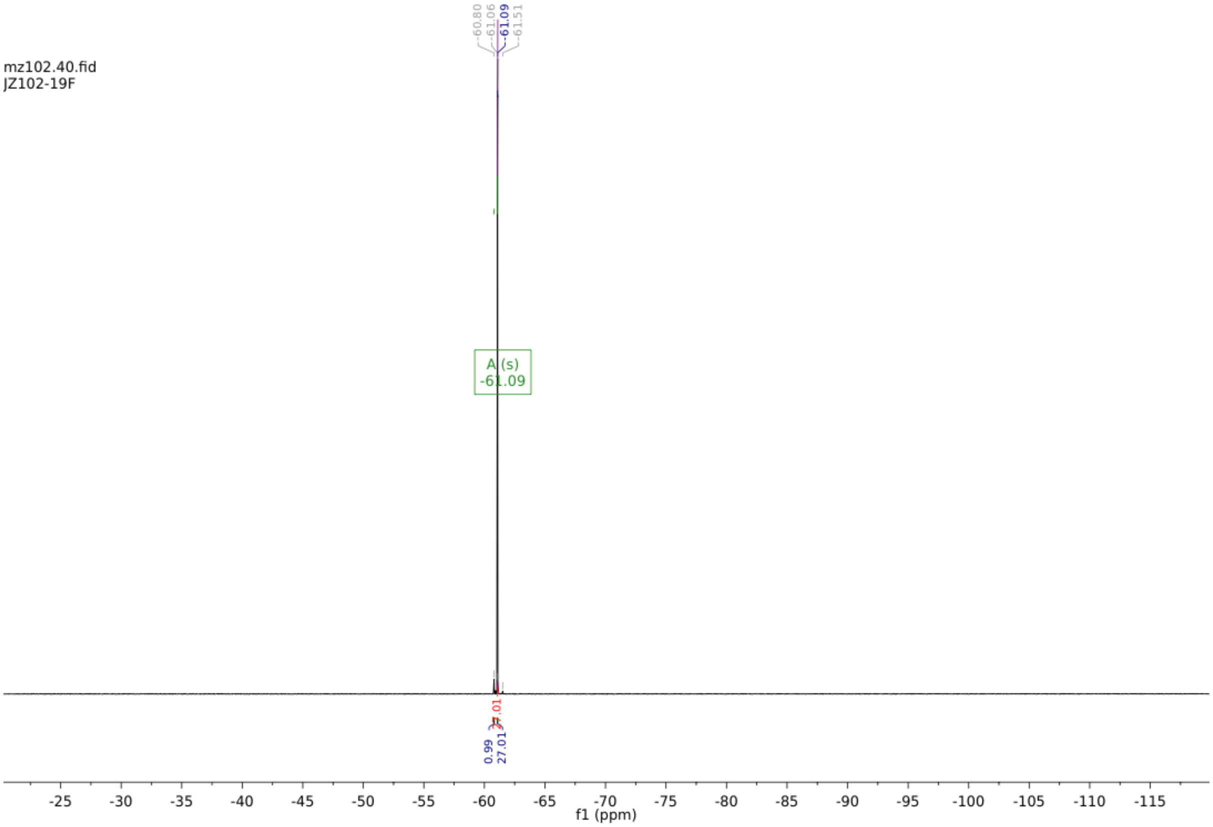
mz102.40.fid
JZ102-19F
A (s) -61.09
f1 (ppm)

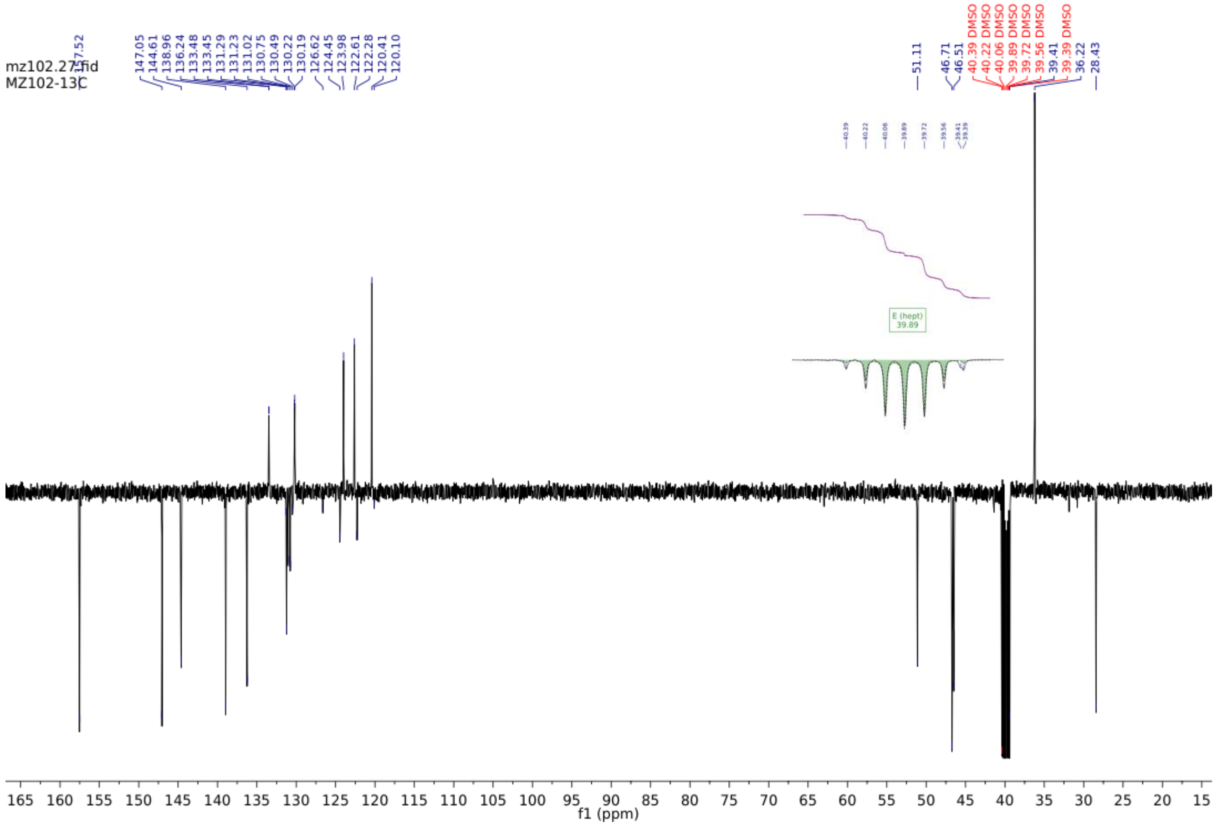
mz102.27.fid
MZ102-13C
157.52
147.05
144.61
138.96
136.24
133.48
133.45
131.29
131.23
131.02
130.75
130.49
130.22
130.19
126.62
124.45
123.98
122.61
122.28
120.41
120.10
51.11
46.71
46.51
40.39 DMSO
40.22 DMSO
40.06 DMSO
39.89 DMSO
39.72 DMSO
39.56 DMSO
39.41
39.39 DMSO
36.22
28.43
f1 (ppm)

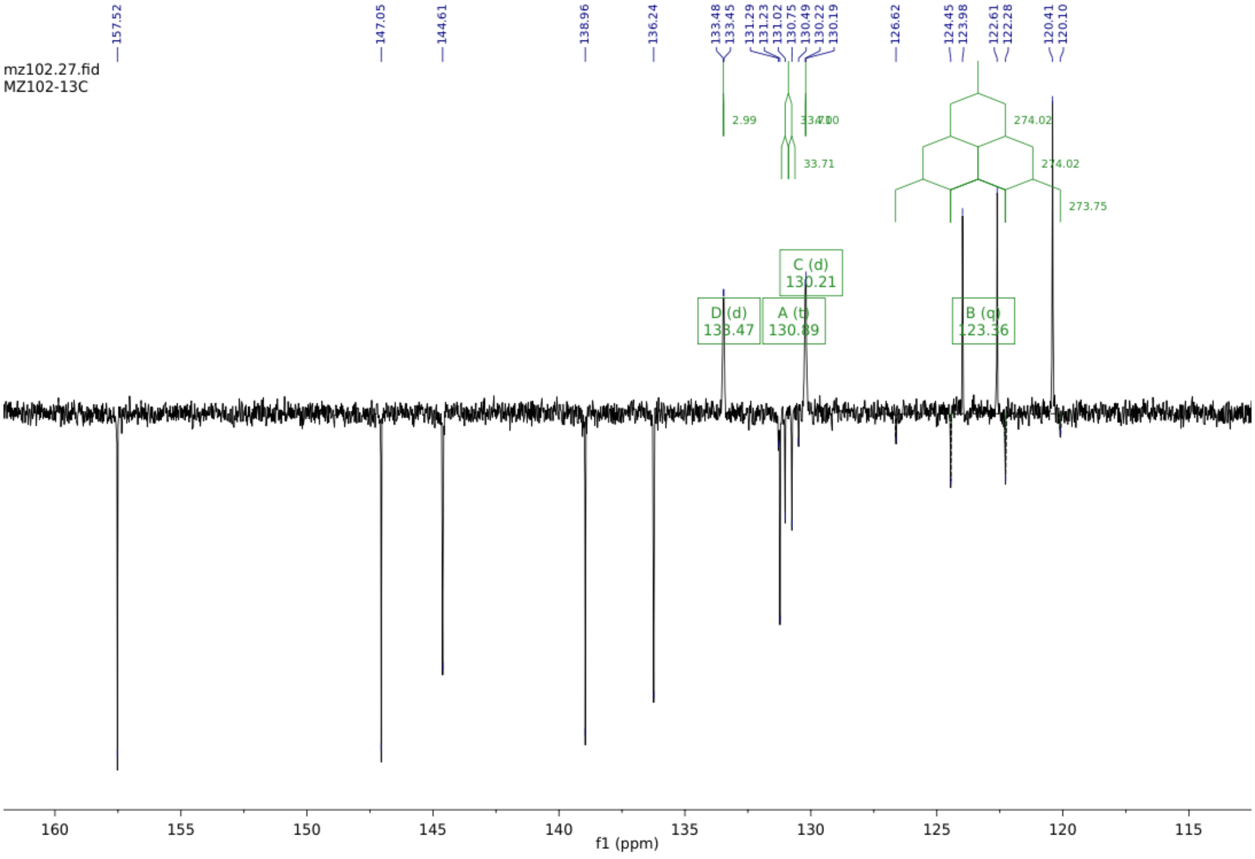
mz102.27.fid
MZ102-13C
157.52
147.05
144.61
138.96
136.24
133.48
133.45
131.29
131.23
131.02
130.75
130.49
130.22
130.19
126.62
124.45
123.98
122.61
122.28
120.41
120.10
C (d)
130.21
D (d)
133.47
A (t)
130.89
B (q)
123.36
f1 (ppm)

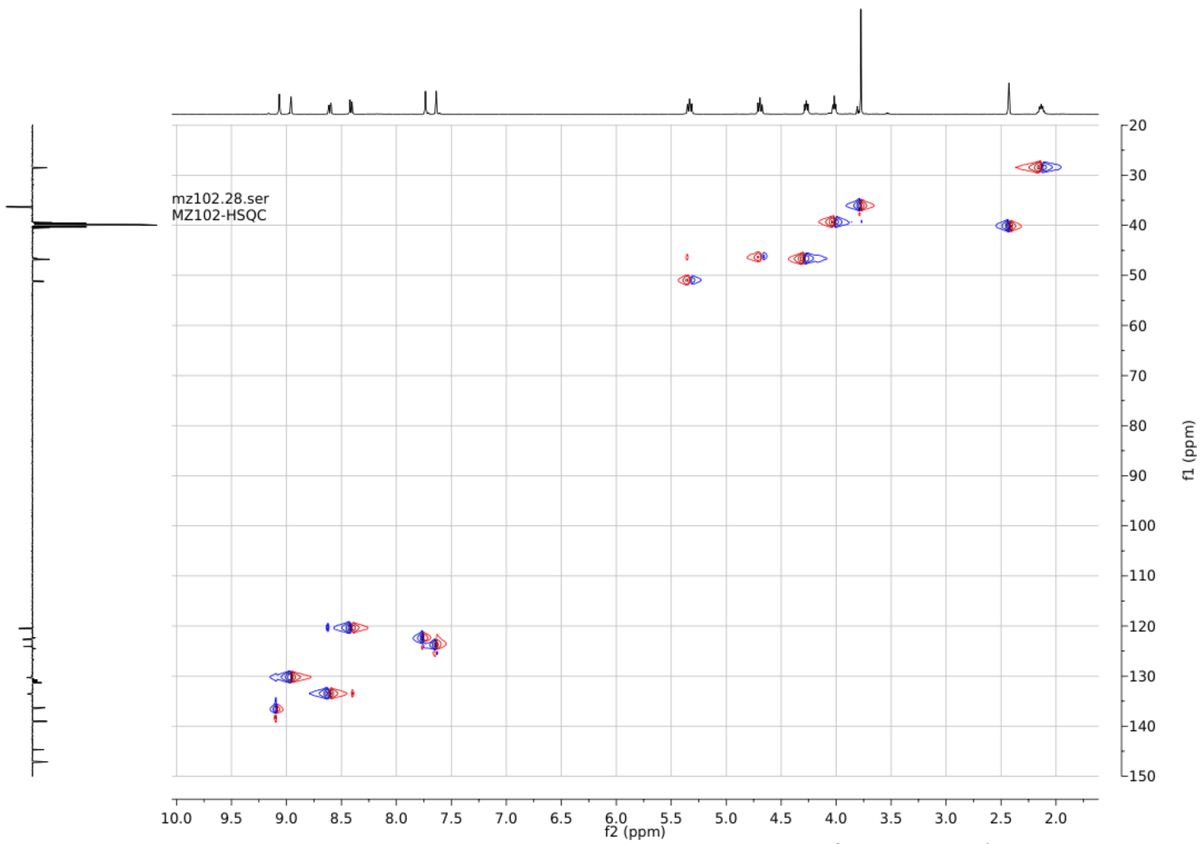
mz102.28.ser
MZ102-HSQC
f2 (ppm)
f1 (ppm)

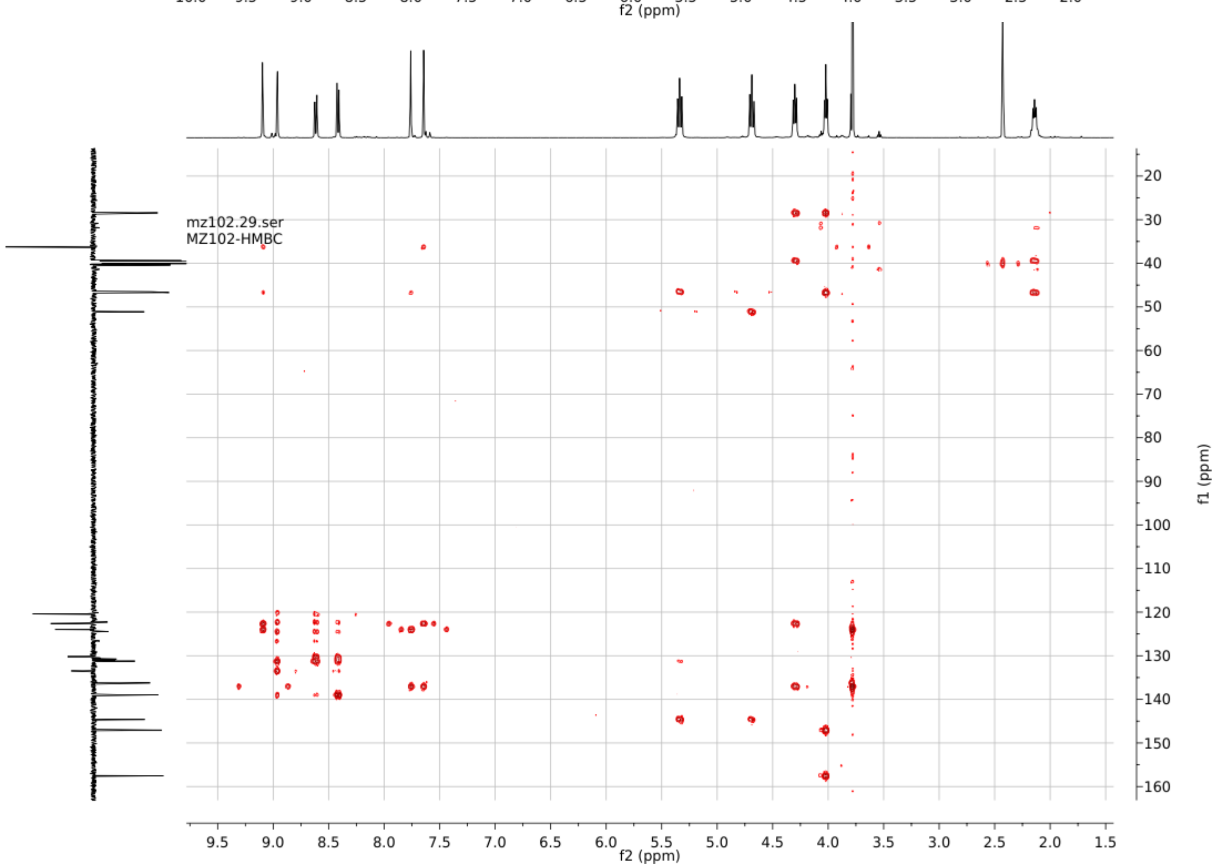
mz102.29.ser
MZ102-HMBC
f2 (ppm)
f1 (ppm)

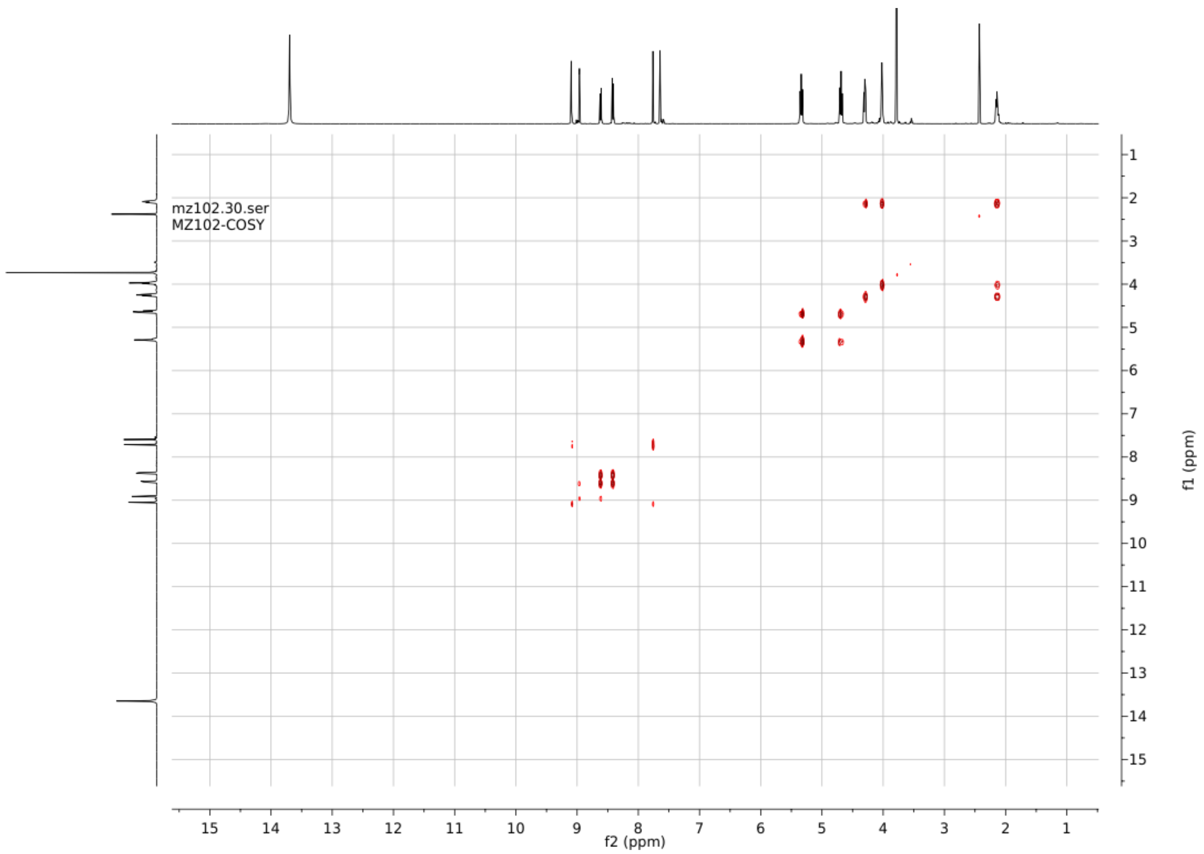
mz102.30.ser
MZ102-COSY
f2 (ppm)
f1 (ppm)

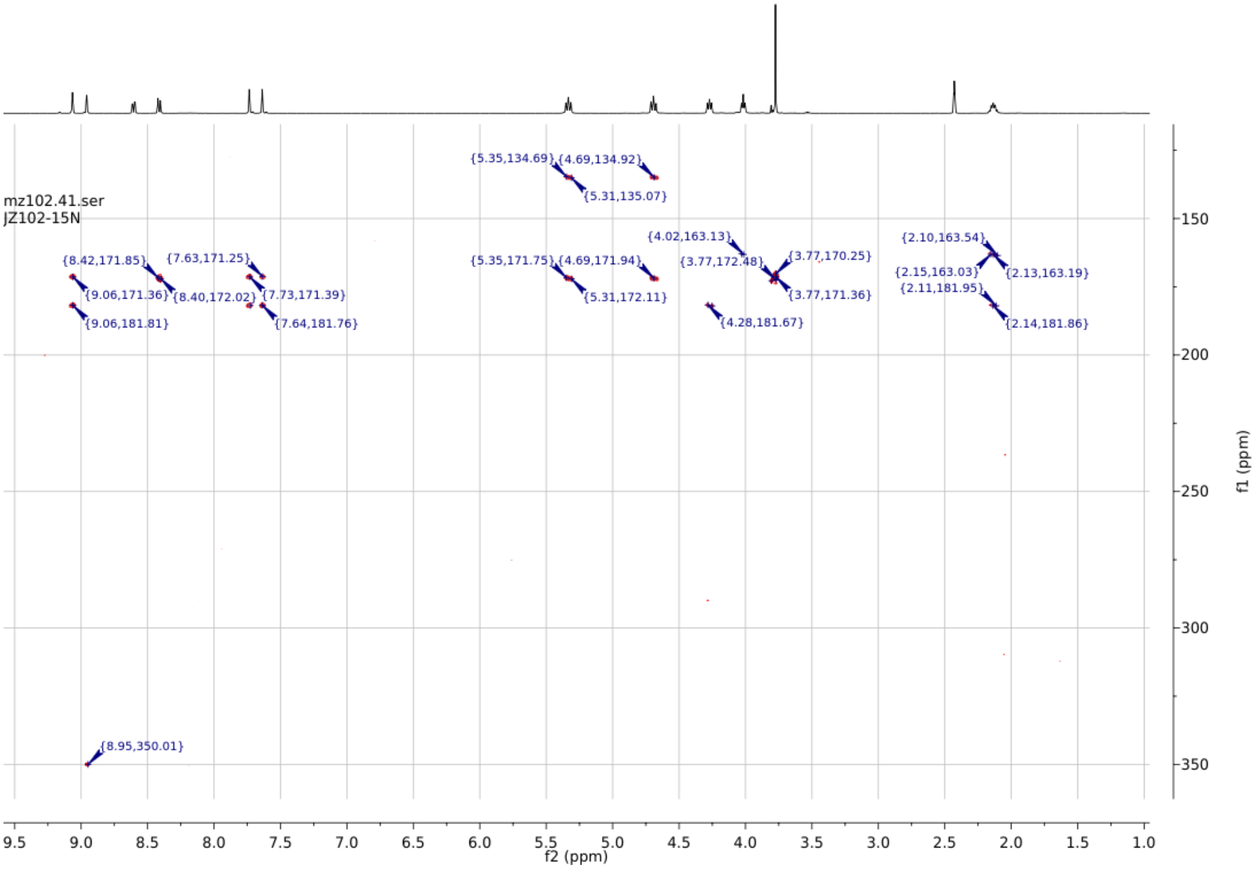
mz102.41.ser
JZ102-15N
{5.35,134.69}
{4.69,134.92}
{5.31,135.07}
{4.02,163.13}
{2.10,163.54}
{8.42,171.85}
{7.63,171.25}
{5.35,171.75}
{4.69,171.94}
{3.77,172.48}
{3.77,170.25}
{2.15,163.03}
{2.13,163.19}
{2.11,181.95}
{9.06,171.36}
{8.40,172.02}
{7.73,171.39}
{5.31,172.11}
{3.77,171.36}
{9.06,181.81}
{7.64,181.76}
{4.28,181.67}
{2.14,181.86}
{8.95,350.01}
f2 (ppm)
f1 (ppm)

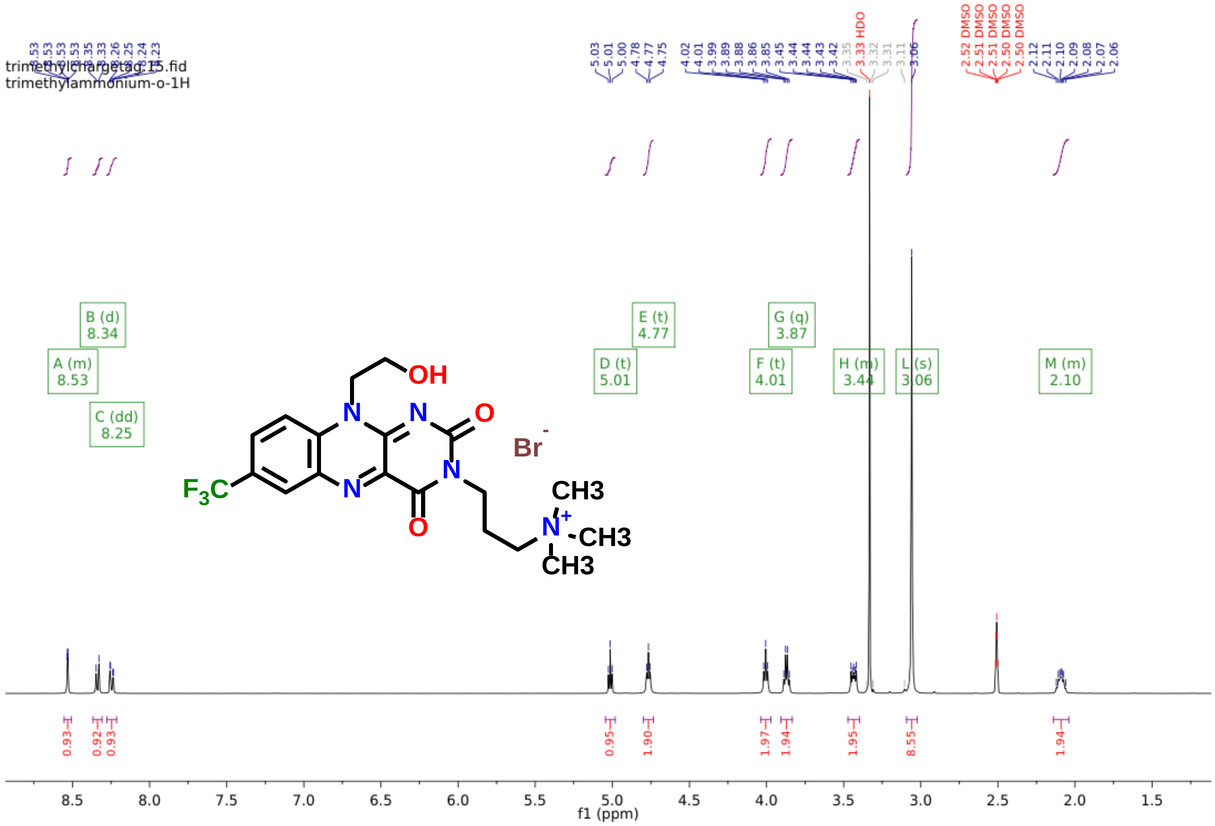
trimethylchargetag.15.fid
trimethylammonium-o-1H
A (m) 8.53
B (d) 8.34
C (dd) 8.25
D (t) 5.01
E (t) 4.77
F (t) 4.01
G (q) 3.87
H (m) 3.44
L (s) 3.06
M (m) 2.10
f1 (ppm)

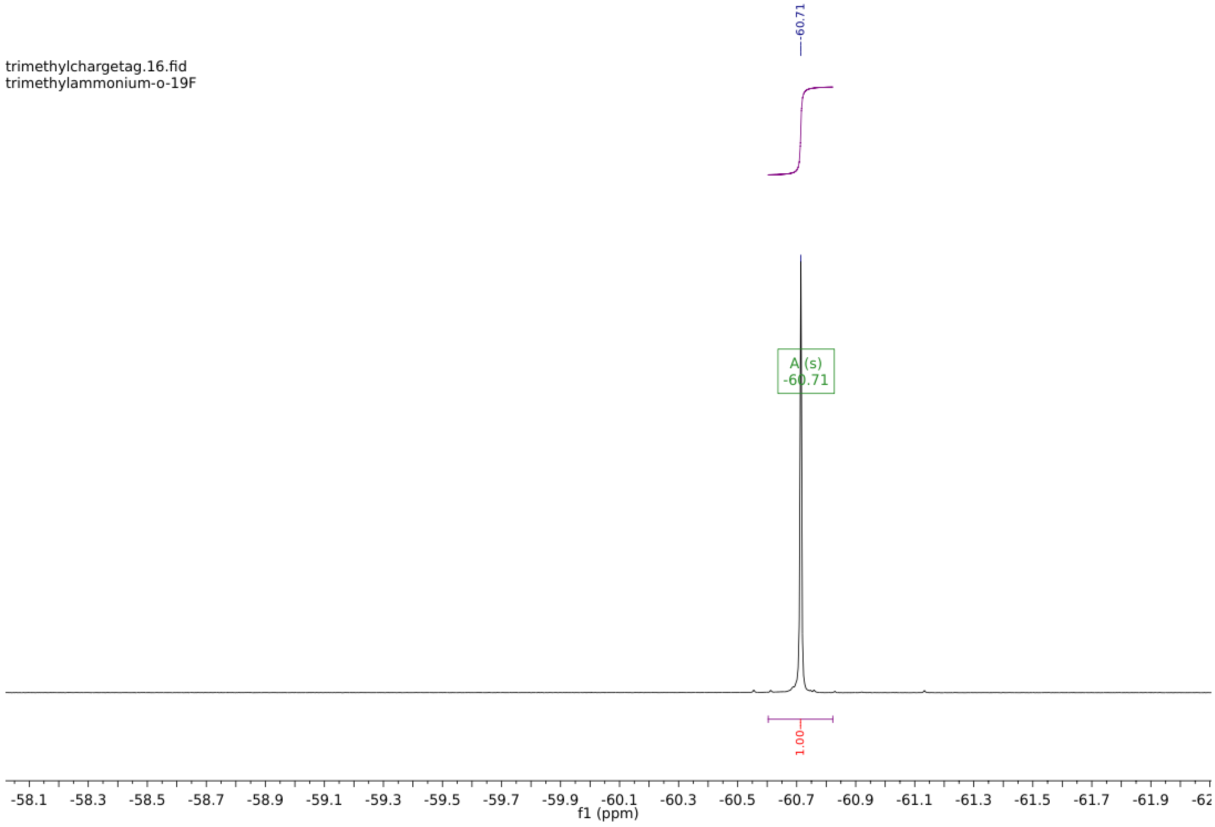
trimethylchargetag.16.fid
trimethylammonium-o-19F
A (s) -60.71
f1 (ppm)

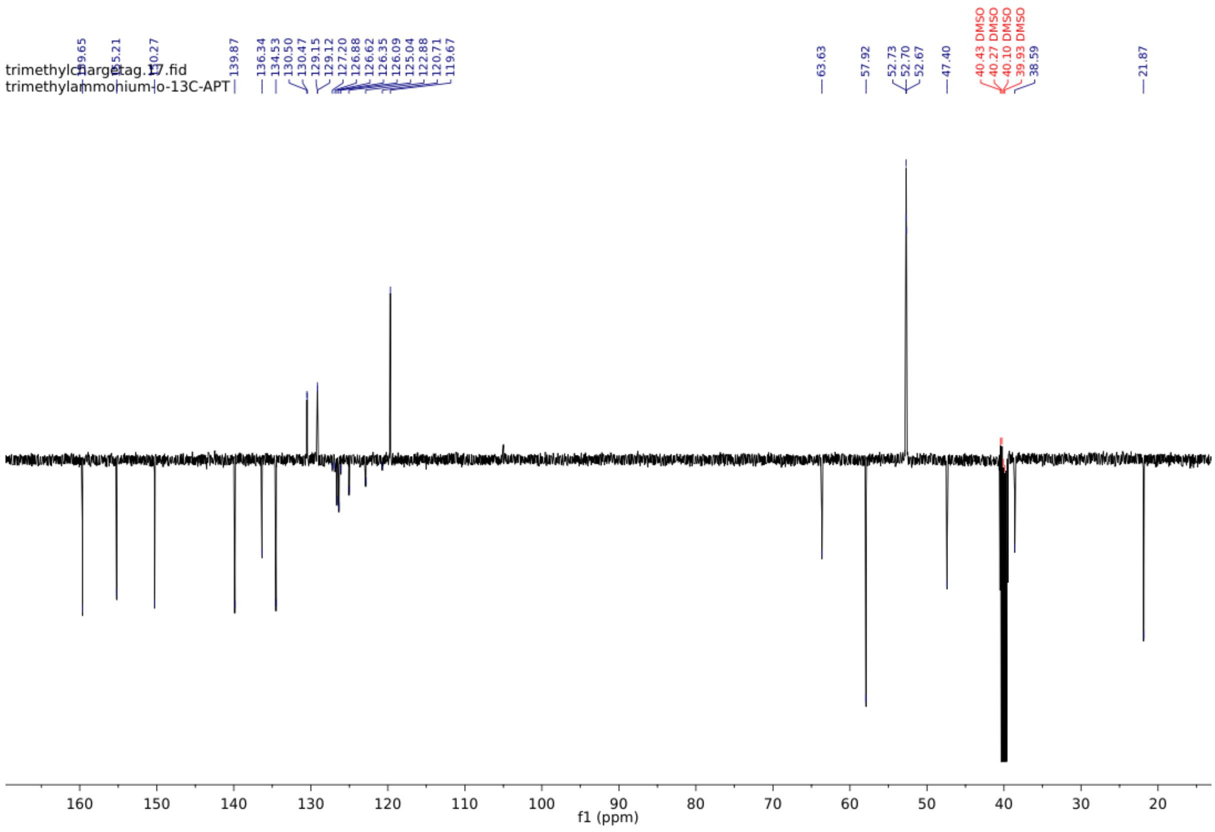
trimethylchargetag.17.fid
trimethylammonium-o-13C-APT
159.65
155.21
150.27
139.87
136.34
134.53
130.50
130.47
129.15
129.12
127.20
126.88
126.62
126.35
126.09
125.04
122.88
120.71
119.67
63.63
57.92
52.73
52.70
52.67
47.40
40.43 DMSO
40.27 DMSO
40.10 DMSO
39.93 DMSO
38.59
21.87
f1 (ppm)

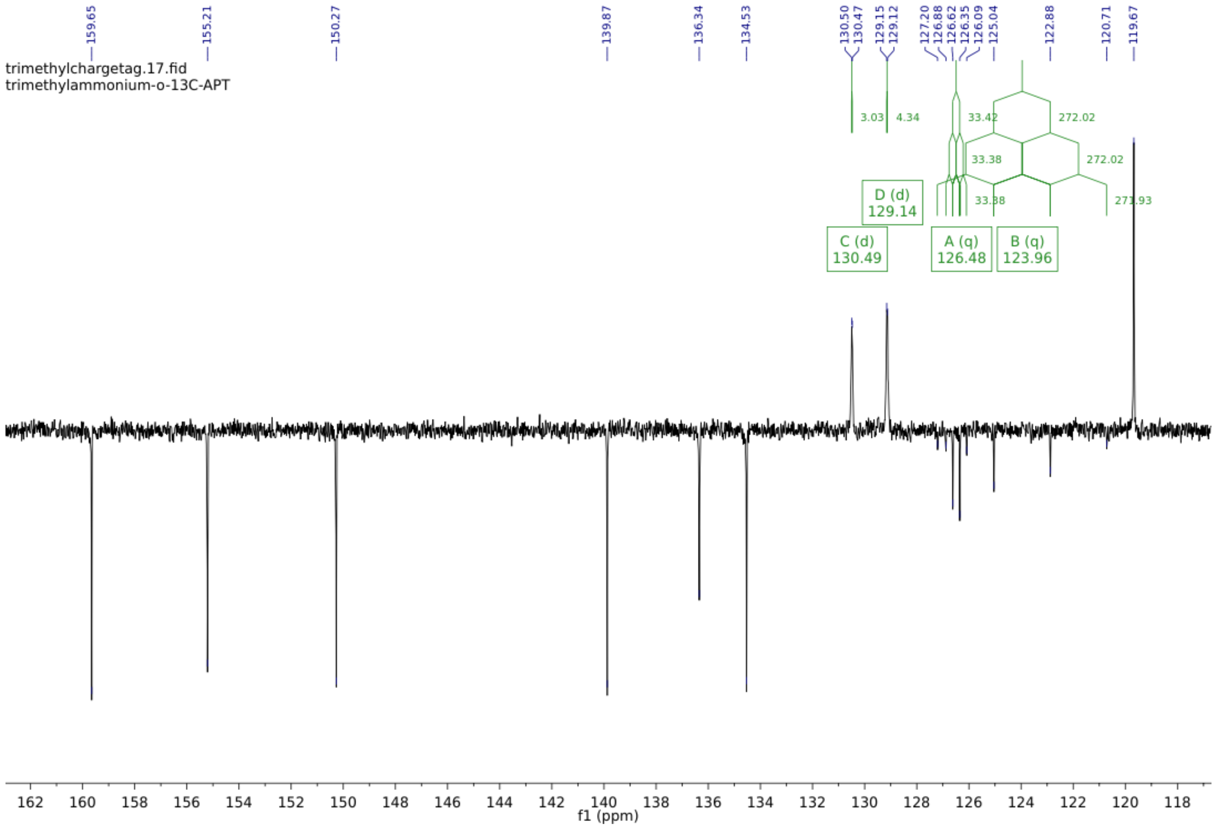
trimethylchargetag.17.fid
trimethylammonium-o-13C-APT
159.65
155.21
150.27
139.87
136.34
134.53
130.50
130.47
129.15
129.12
127.20
126.88
126.62
126.35
126.09
125.04
122.88
120.71
119.67
3.03
4.34
33.42
272.02
33.38
272.02
33.38
271.93
D (d)
129.14
C (d)
130.49
A (q)
126.48
B (q)
123.96
f1 (ppm)

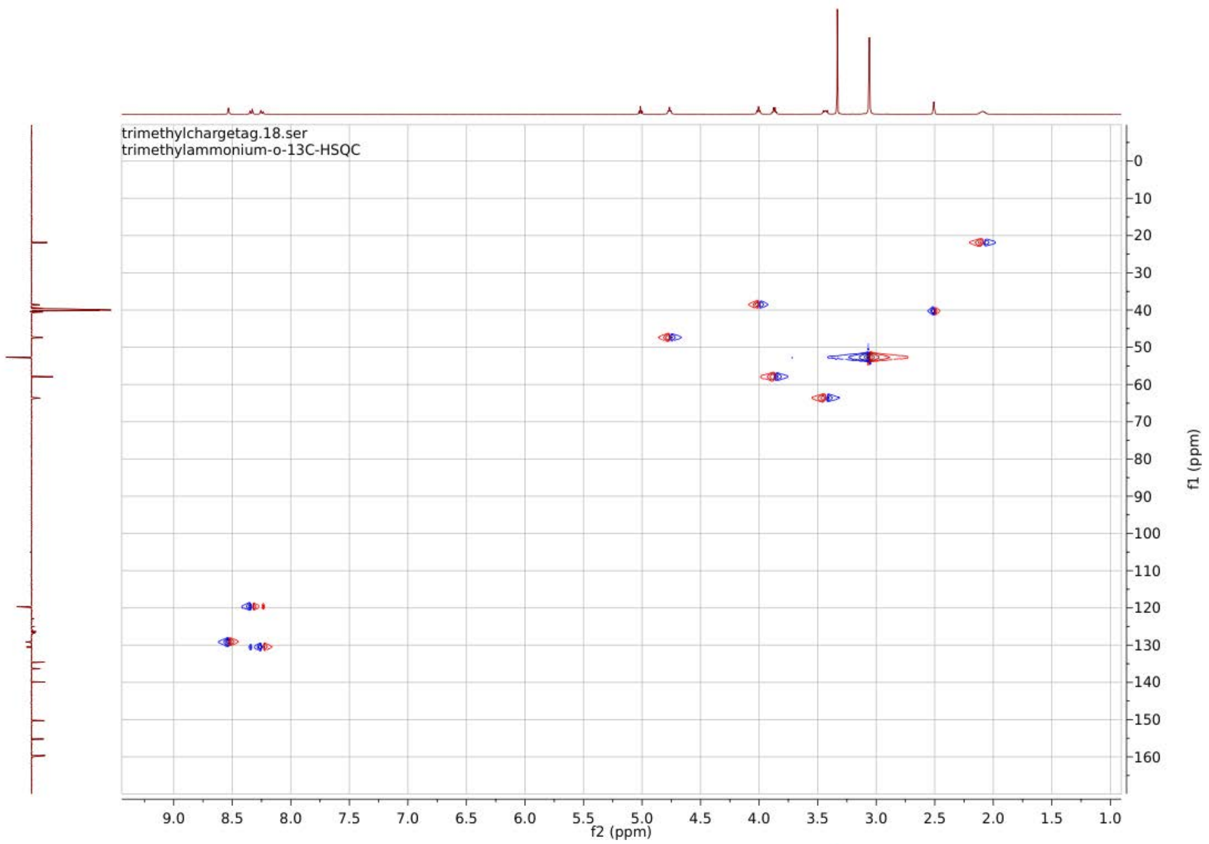
trimethylchargetag.18.ser
trimethylammonium-o-13C-HSQC
f2 (ppm)
f1 (ppm)

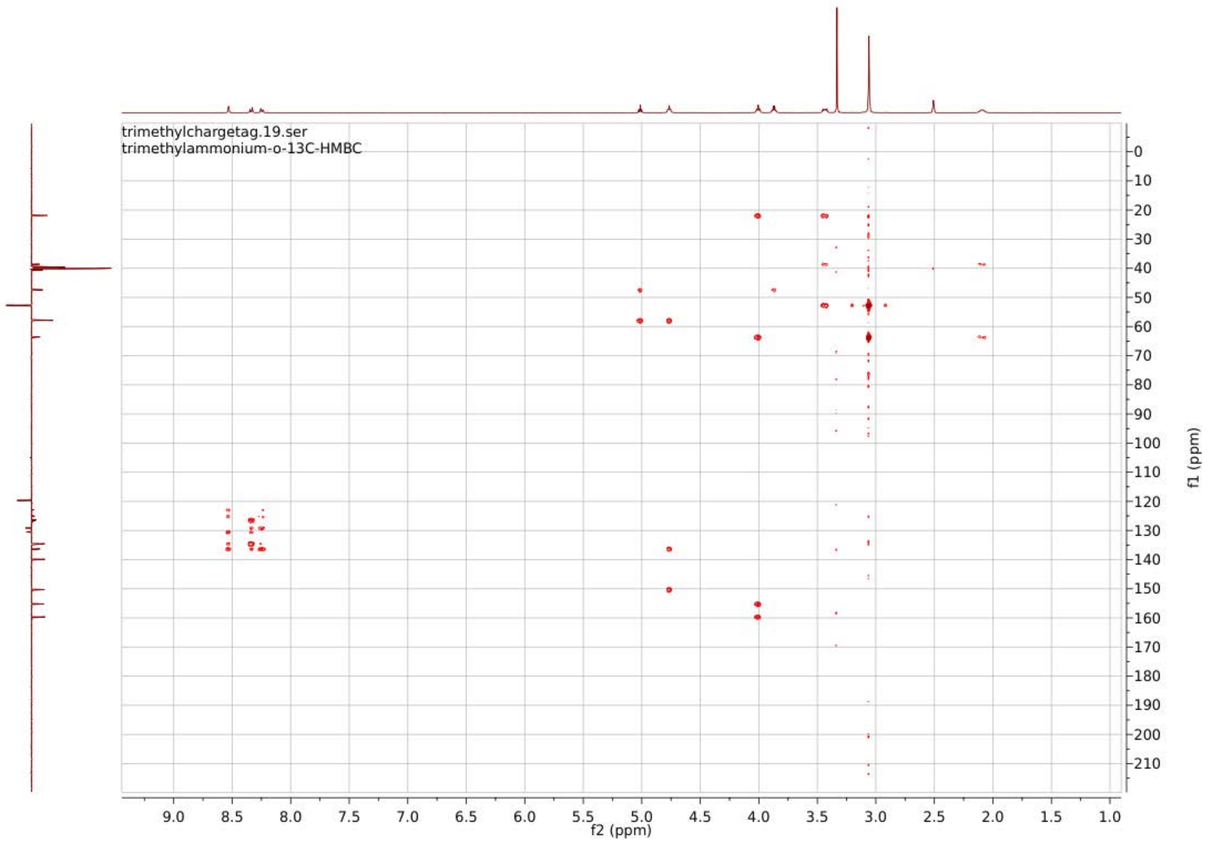
trimethylchargetag.19.ser
trimethylammonium-o-13C-HMBC
f2 (ppm)
f1 (ppm)

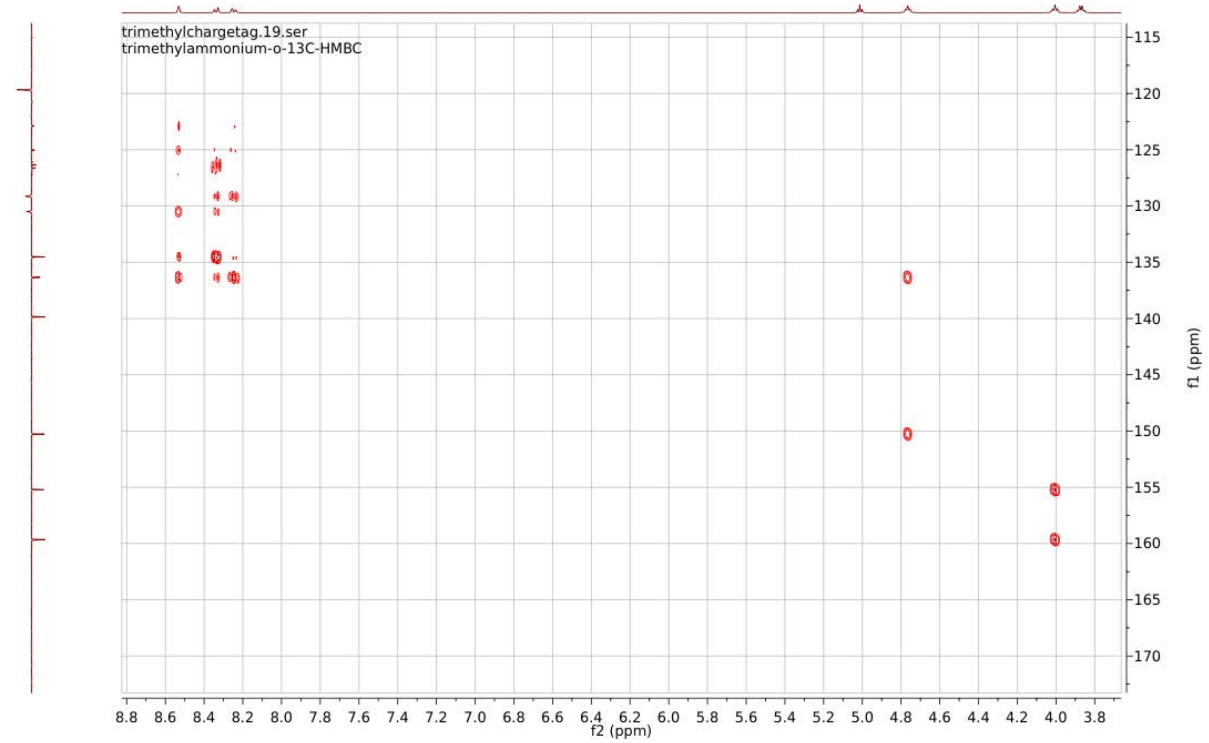

trimethylchargetag.19.ser
trimethylammonium-o-13C-HMBC
f1 (ppm)
f2 (ppm)

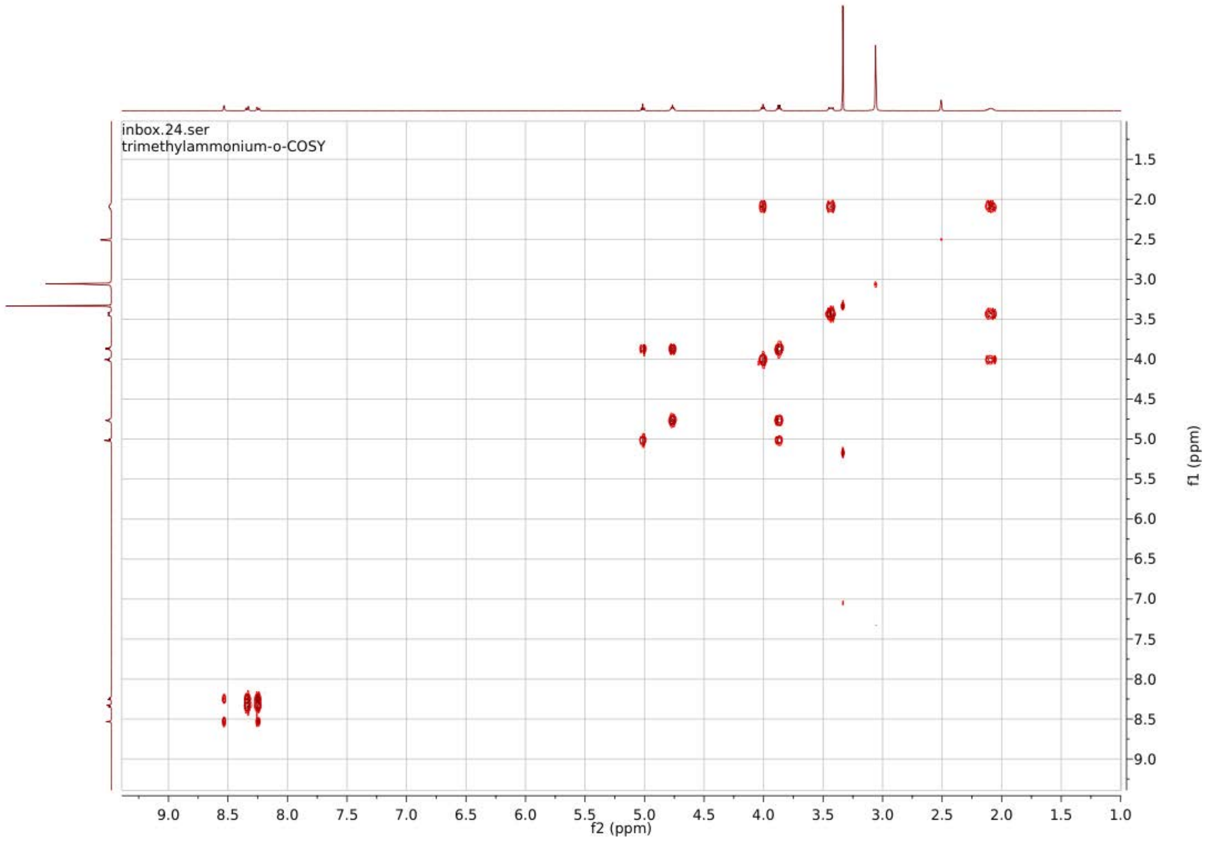

inbox.24.ser
trimethylammonium-o-COSY
f1 (ppm)
f2 (ppm)

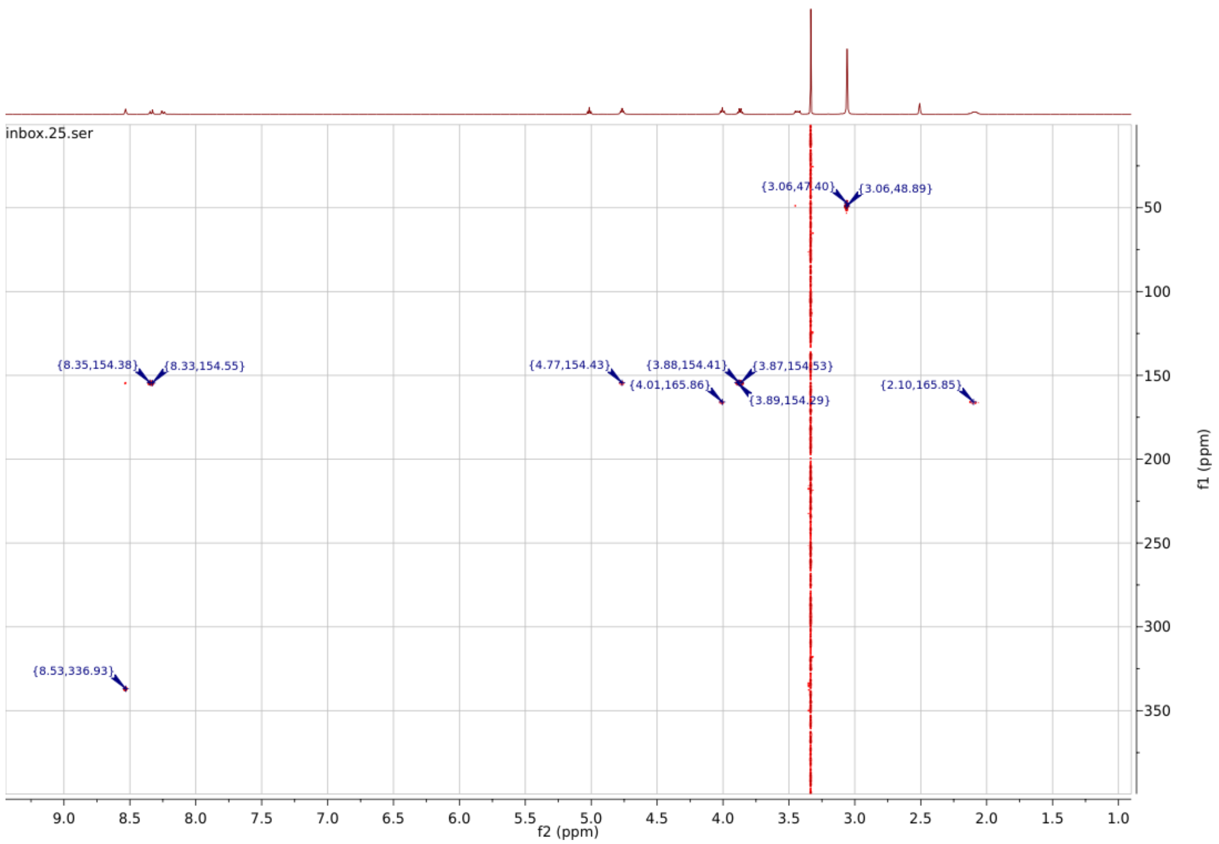
inbox.25.ser
{3.06,47.40}
{3.06,48.89}
{8.35,154.38}
{8.33,154.55}
{4.77,154.43}
{3.88,154.41}
{3.87,154.53}
{4.01,165.86}
{3.89,154.29}
{2.10,165.85}
{8.53,336.93}
f2 (ppm)
f1 (ppm)

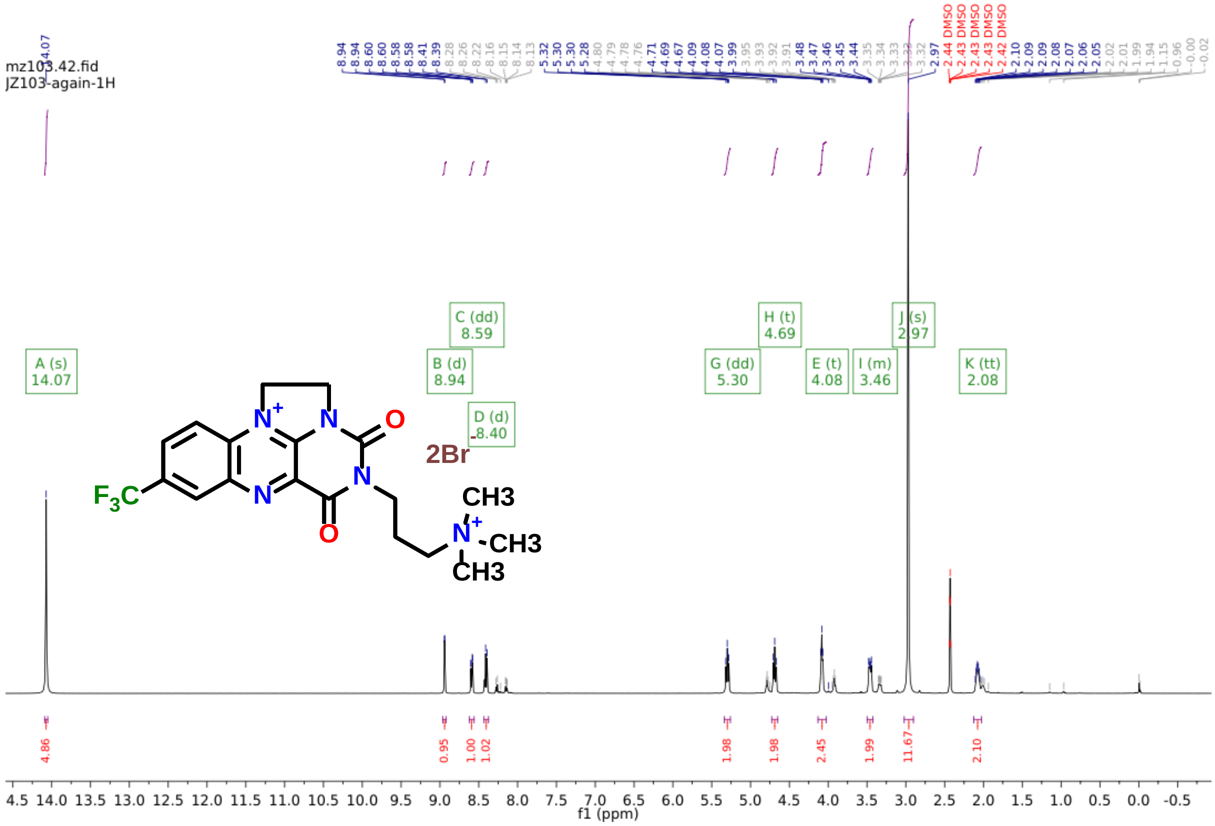
mz103.42.fid
JZ103-again-1H
A (s) 14.07
B (d) 8.94
C (dd) 8.59
D (d) 8.40
G (dd) 5.30
H (t) 4.69
E (t) 4.08
I (m) 3.46
J (s) 2.97
K (tt) 2.08
2Br
4.86
0.95
1.00
1.02
1.98
1.98
2.45
1.99
11.67
2.10
f1 (ppm)

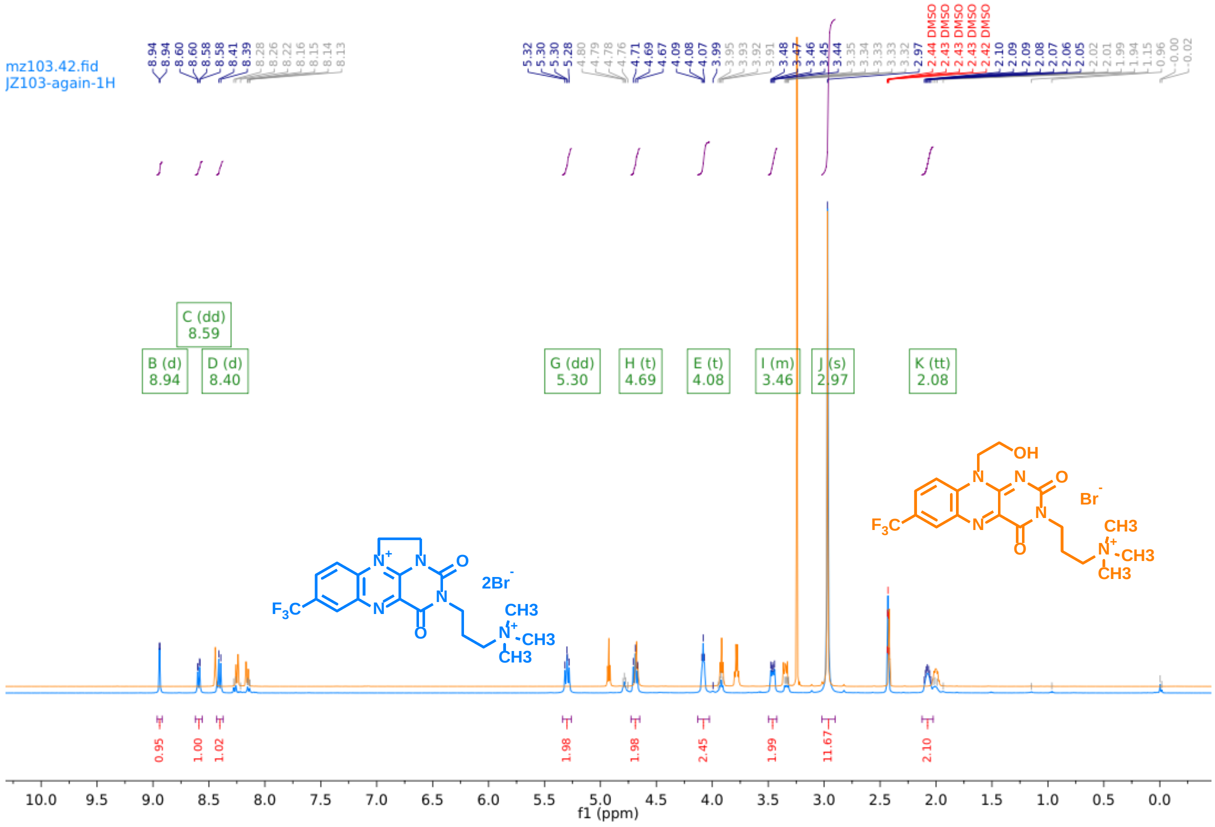
mz103.42.fid
JZ103-again-1H
B (d) 8.94
C (dd) 8.59
D (d) 8.40
G (dd) 5.30
H (t) 4.69
E (t) 4.08
I (m) 3.46
J (s) 2.97
K (tt) 2.08
2Br
Br
0.95
1.00
1.02
1.98
1.98
2.45
1.99
11.67
2.10
f1 (ppm)

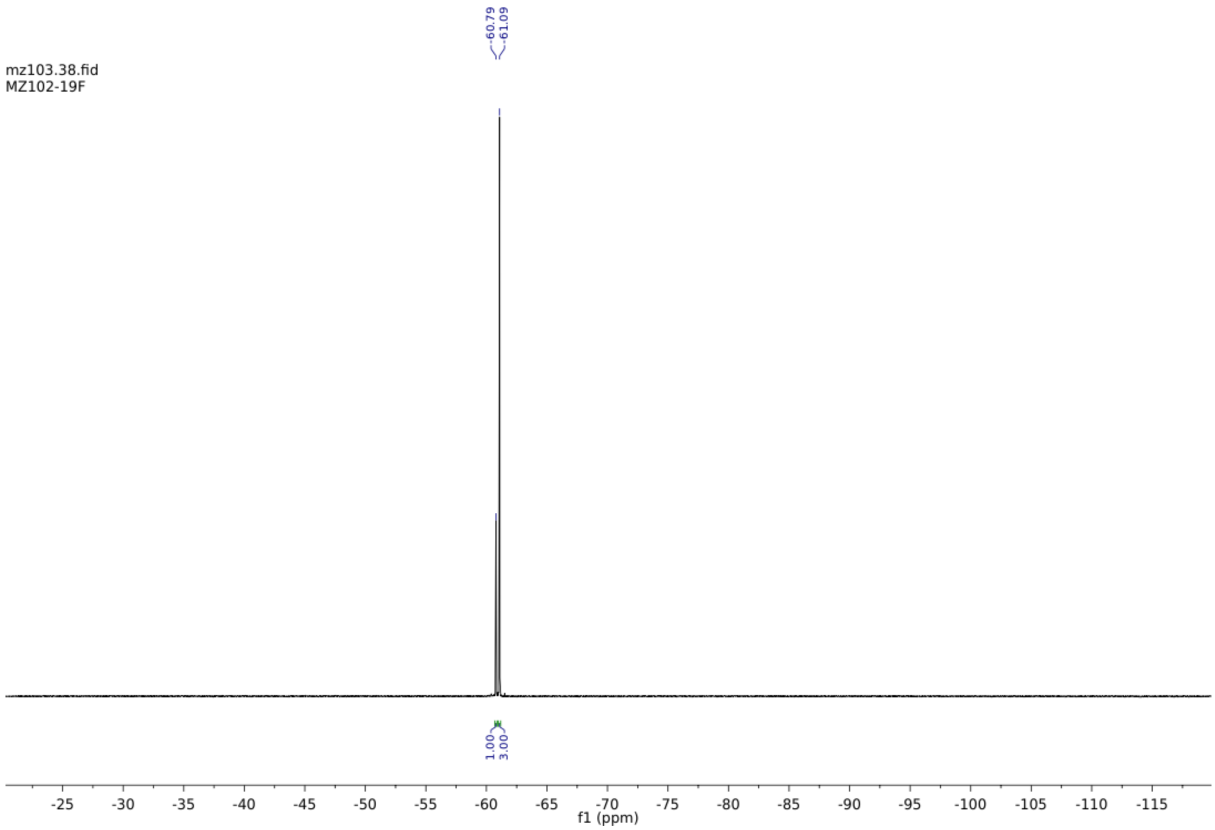
mz103.38.fid
MZ102-19F
-60.79
-61.09
1.00
3.00
f1 (ppm)

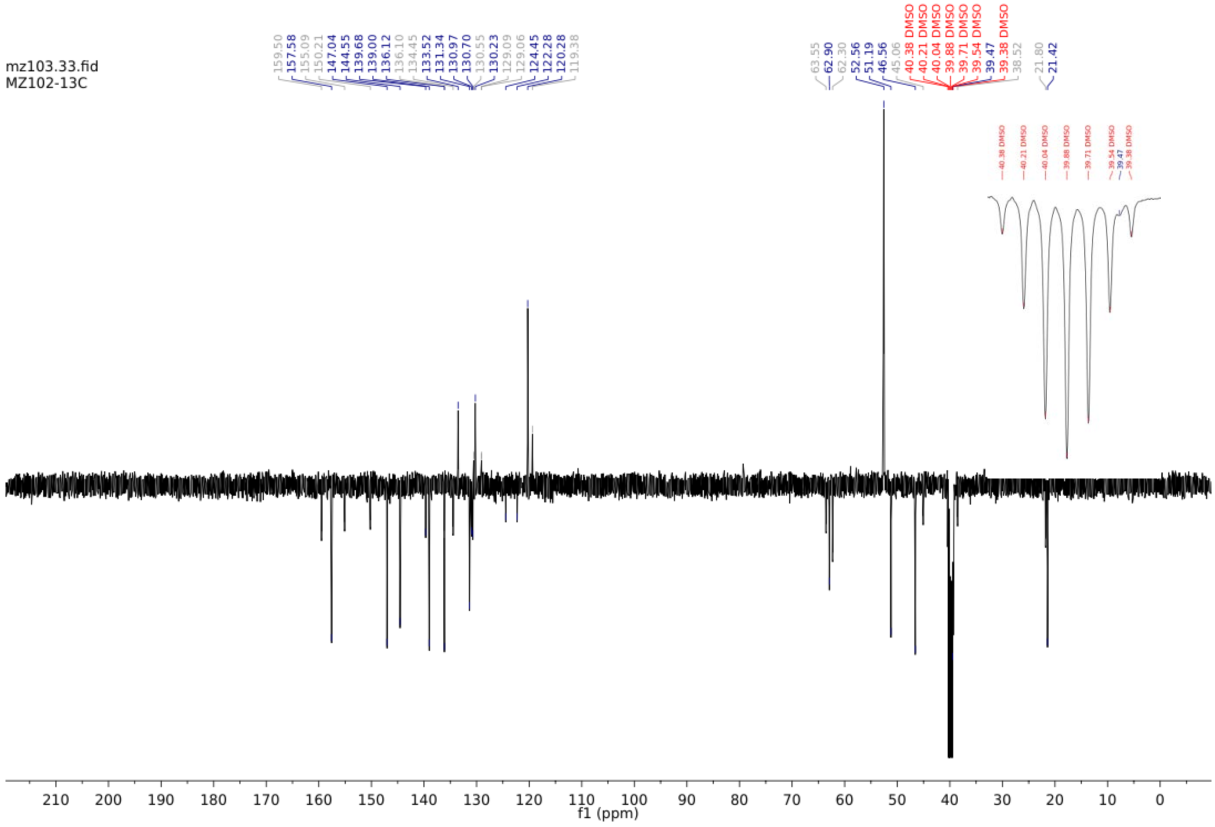
mz103.33.fid
MZ102-13C
159.50
157.58
155.09
150.21
147.04
144.55
139.68
139.00
136.12
136.10
134.45
133.52
131.34
130.97
130.70
130.55
130.23
129.09
129.06
124.45
122.28
120.28
119.38
63.55
62.90
62.30
52.56
51.19
46.56
45.06
40.38 DMSO
40.21 DMSO
40.04 DMSO
39.88 DMSO
39.71 DMSO
39.54 DMSO
39.47
39.38 DMSO
38.52
21.80
21.42
f1 (ppm)

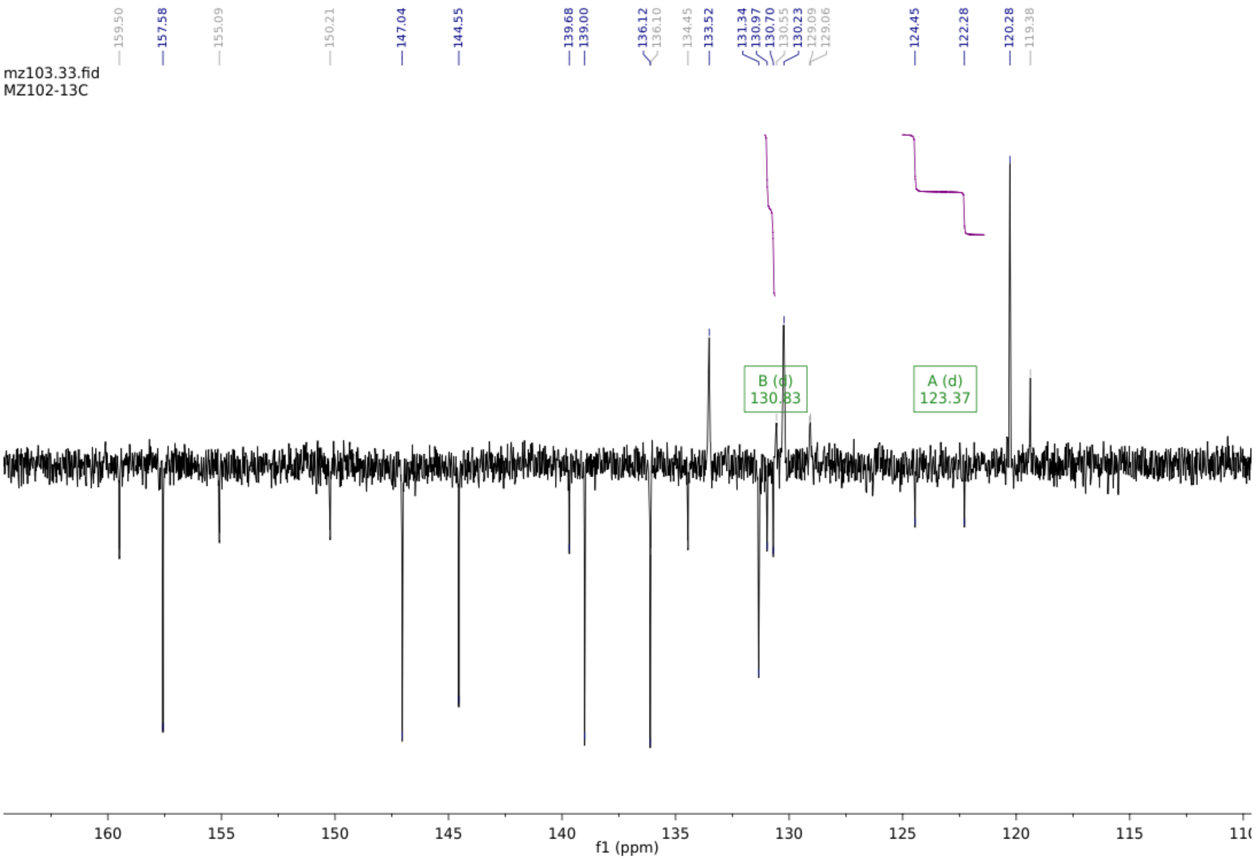
mz103.33.fid
MZ102-13C
159.50
157.58
155.09
150.21
147.04
144.55
139.68
139.00
136.12
136.10
134.45
133.52
131.34
130.97
130.70
130.55
130.23
129.09
129.06
124.45
122.28
120.28
119.38
B (d)
130.83
A (d)
123.37
f1 (ppm)

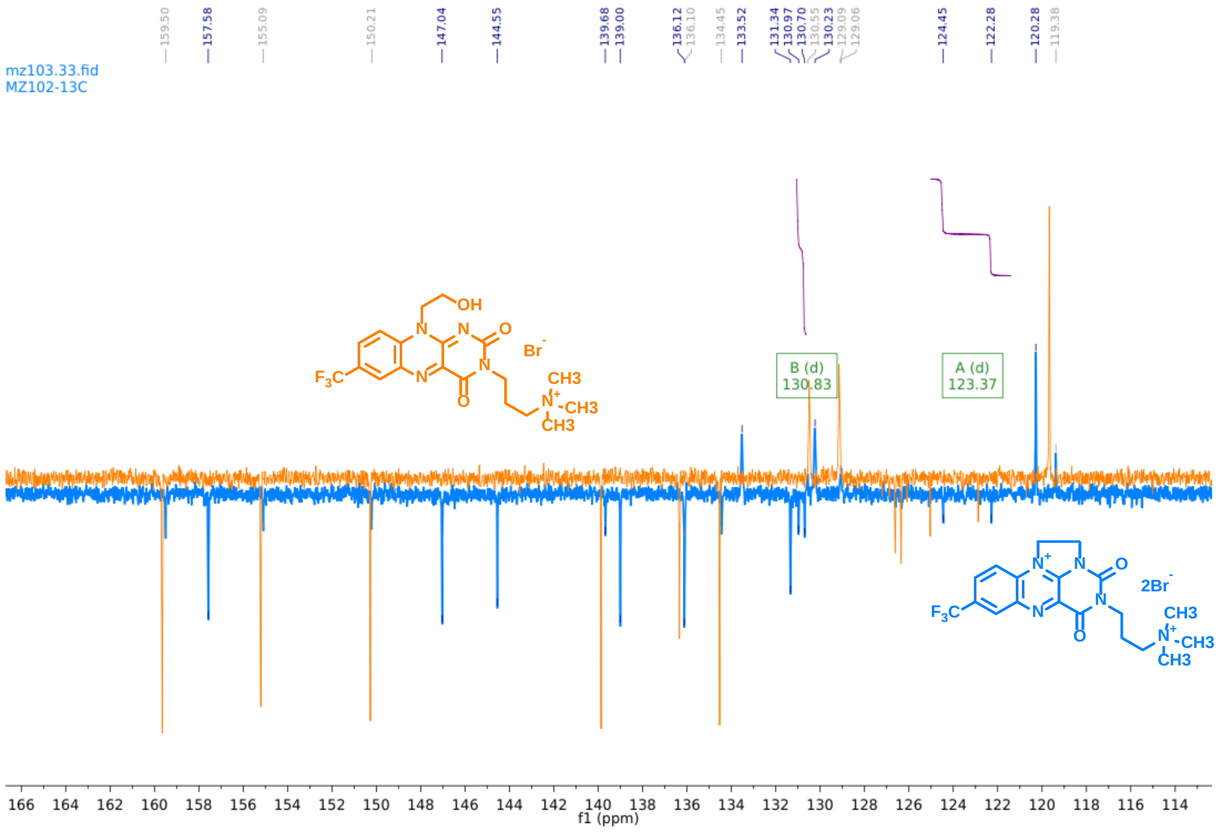
mz103.33.fid
MZ102-13C
159.50
157.58
155.09
150.21
147.04
144.55
139.68
139.00
136.12
136.10
134.45
133.52
131.34
130.97
130.70
130.55
130.23
129.09
129.06
124.45
122.28
120.28
119.38
B (d)
130.83
A (d)
123.37
2Br⁻
f1 (ppm)

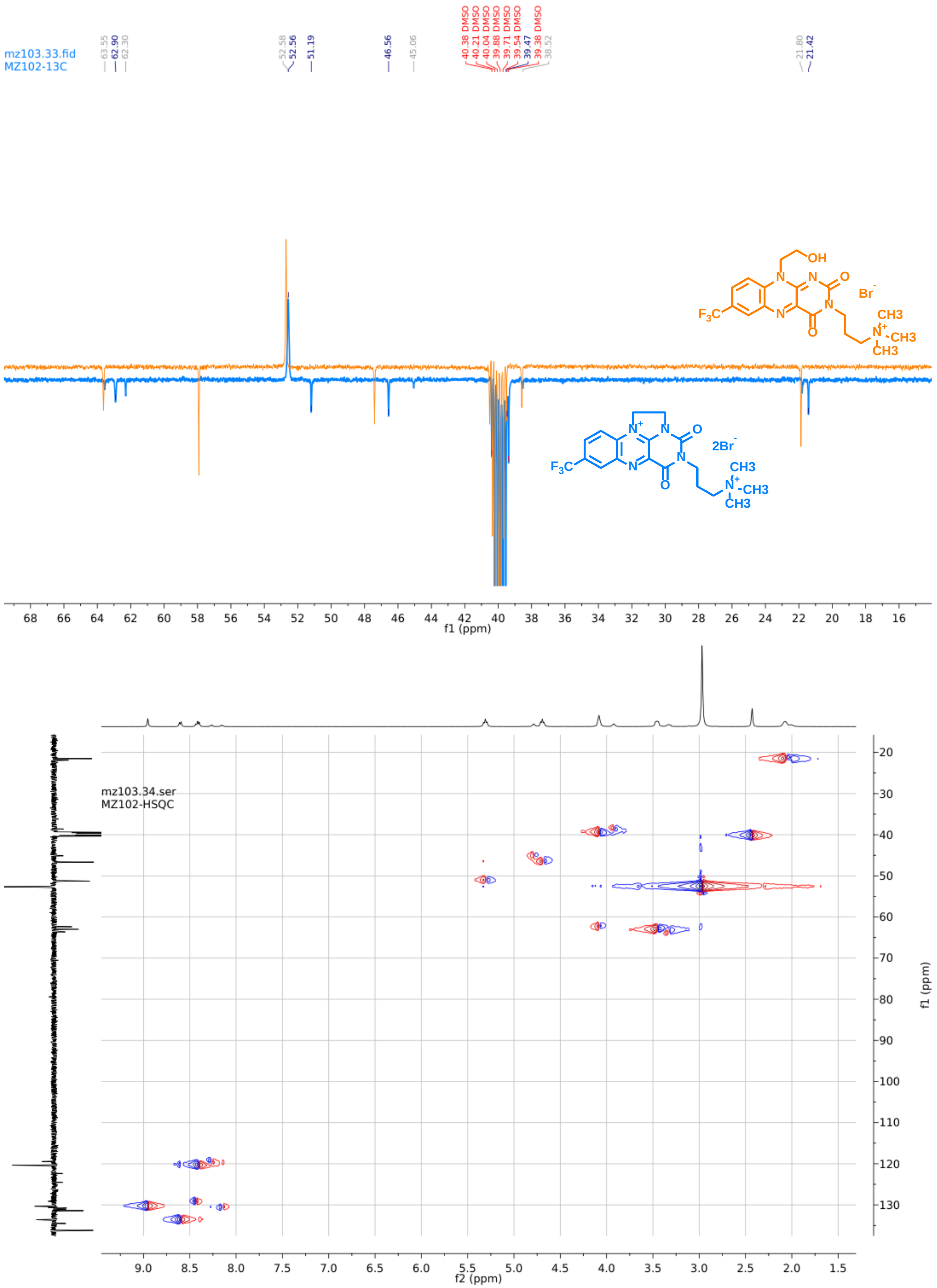

mz103.33.fid
MZ102-13C
63.55
62.90
62.30
52.58
52.56
51.19
46.56
45.06
40.38 DMSO
40.21 DMSO
40.04 DMSO
39.88 DMSO
39.71 DMSO
39.54 DMSO
39.47
39.38 DMSO
38.52
21.80
21.42
Br-
2Br-
f1 (ppm)
mz103.34.ser
MZ102-HSQC
f2 (ppm)
f1 (ppm)

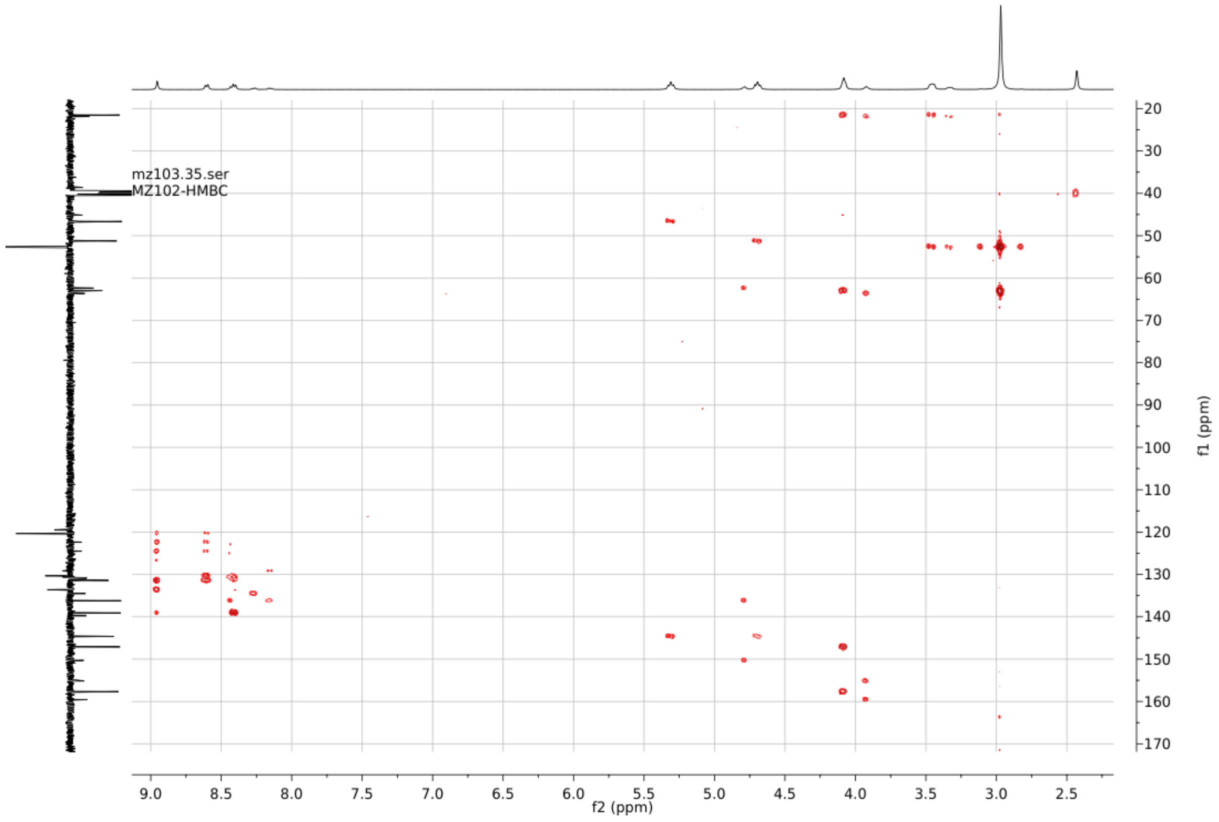
mz103.35.ser
MZ102-HMBC
f2 (ppm)
f1 (ppm)

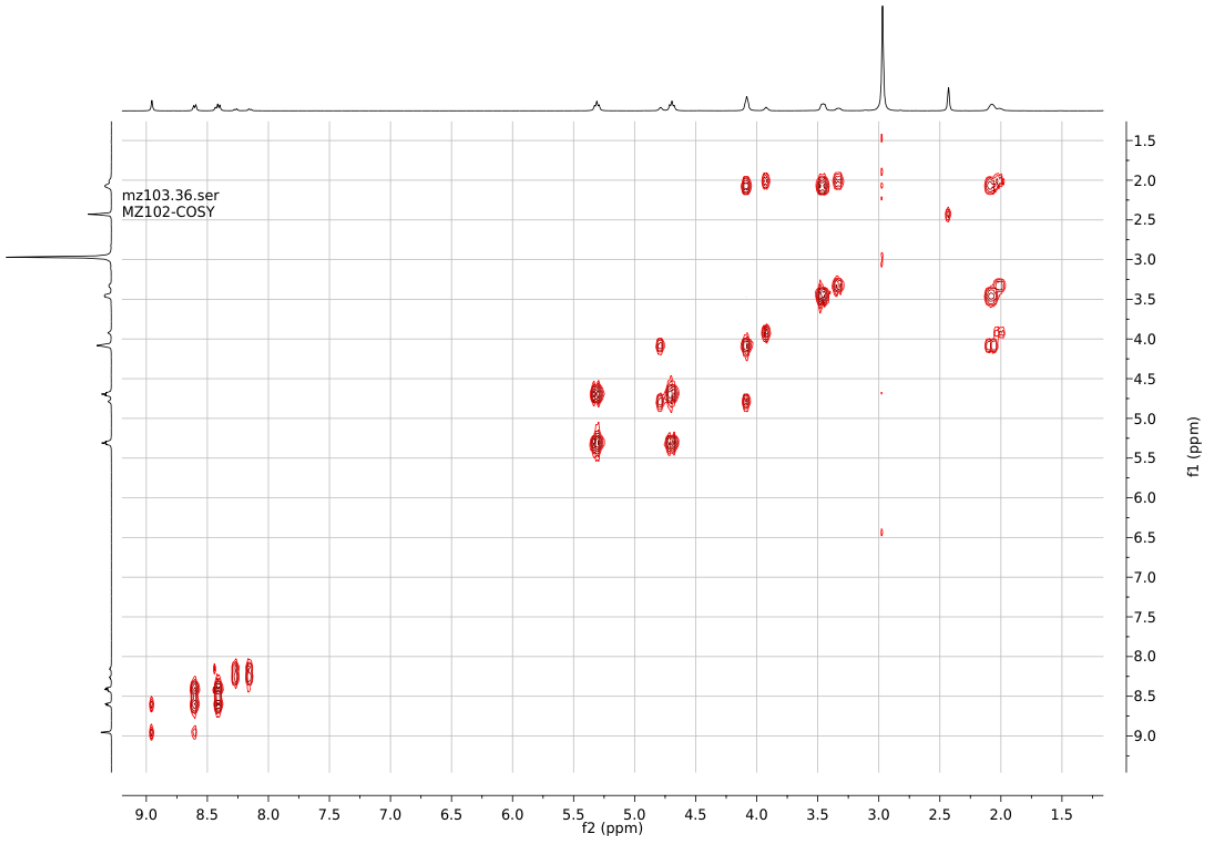
mz103.36.ser
MZ102-COSY
f2 (ppm)
f1 (ppm)

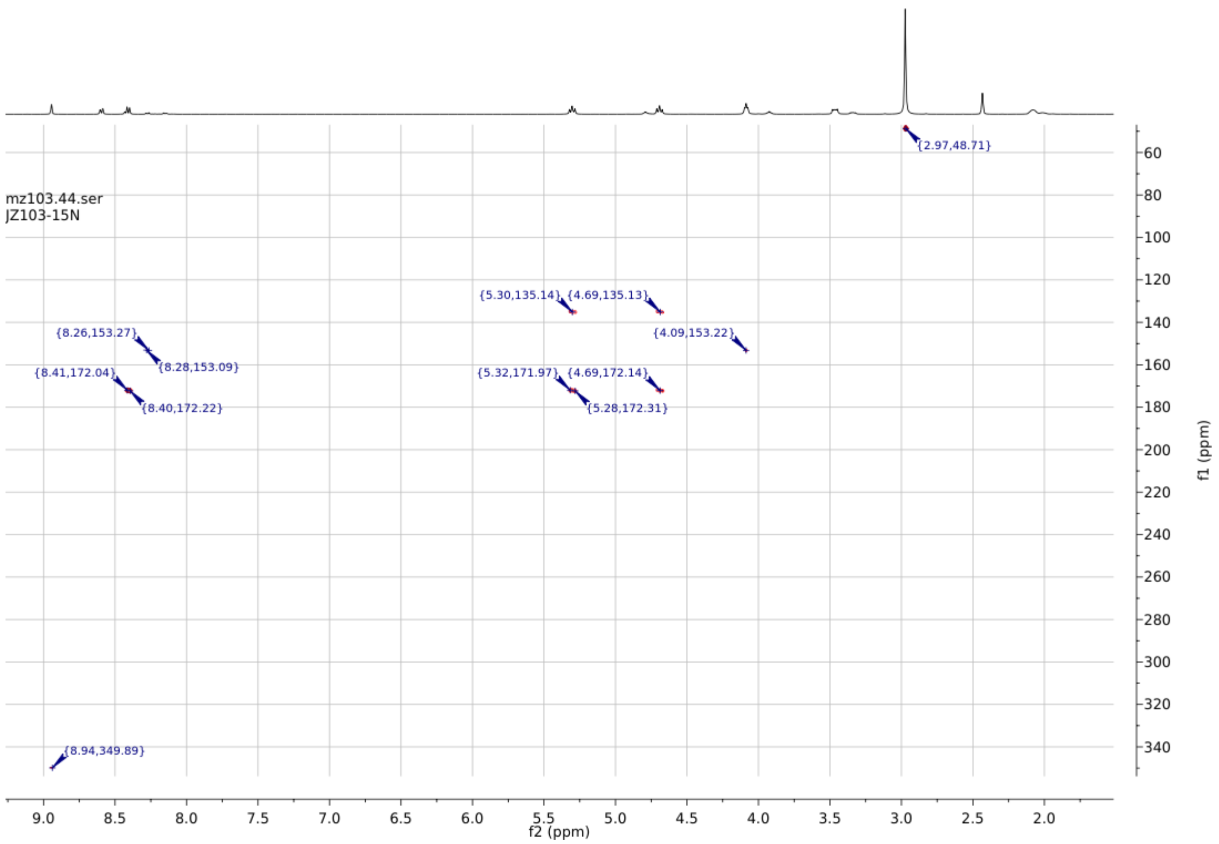

mz103.44.ser
JZ103-15N
{2.97,48.71}
{5.30,135.14}
{4.69,135.13}
{8.26,153.27}
{4.09,153.22}
{8.28,153.09}
{8.41,172.04}
{5.32,171.97}
{4.69,172.14}
{8.40,172.22}
{5.28,172.31}
{8.94,349.89}
f2 (ppm)
f1 (ppm)

7 HRMS-ESI+ spectra of the synthetized compounds

HRMS spectra were acquired on the JEOL JMS-T100CS AccuTOF-CS mass spectrometer using polypropylene glycol (PPG) as an internal standard for mass drift compensation. The allowed difference limit below m/z 1000 is 3 mmu.

Data:24-04
Sample Name:Jan Zelenka [Rol], UK28, M=446
Description:
Ionization Mode:ESI+
History:Determine m/z[Peak Detect[Centroid,30,Area];Correct Base[];Average(MS[1] 0.18..0.39)-1.0*Average(MS[1] 0.06..0.11);Correct Base[5.0%]]

Acquired:24-Aug-18 10:30:06
Operator:Accutof
Mass Calibration data:TFANa_ESI+_1000
Created:24-Aug-18 10:43:12
Created by:Accutof

Charge number:1
Tolerance:5.00(ppm), 3.00 .. (mmu)
Unsaturation Number:-1.5 .. 99.0 (Fraction:Both)
Element:^{12}C:13 .. 100, ^{1}H:1 .. 100, ^{79}Br:1 .. 3, ^{19}F:1 .. 5, ^{14}N:1 .. 10, ^{23}Na:0 .. 1, ^{16}O:1 .. 10

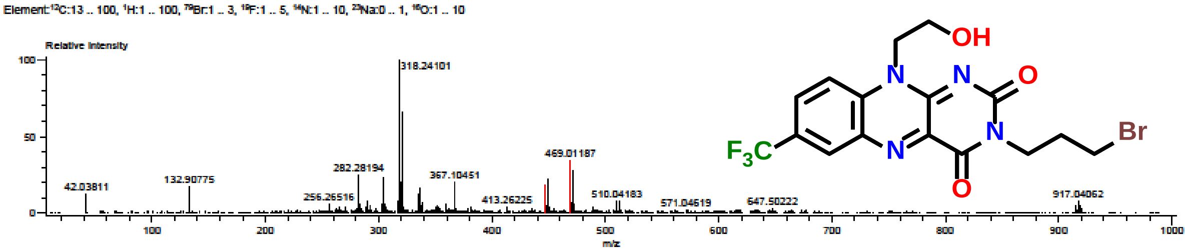

Mass	Decision	Calc. Mass	Mass Difference (mmu)	Mass Difference (ppm)	^{12}C	^{1}H	^{79}Br	^{19}F	^{14}N	^{23}Na	^{16}O	Unsaturation Number
447.03016	+	447.02796	2.20	4.93	16	15	1	3	4		3	9.5
	?	447.03021	-0.04	-0.10	13	15	1	1	7		5	9.0
	?	447.03045	-0.28	-0.63	15	18	1	4	1		5	5.0
	?	447.03048	-0.32	-0.71	14	14	1	1	8	1	2	10.5
	?	447.03049	-0.32	-0.72	15	20	1	1	1	1	7	5.0
	?	447.03072	-0.56	-1.25	16	17	1	4	2	1	2	6.5
	?	447.02958	0.58	1.31	19	16	1	3	2	1	1	10.5
	?	447.02942	0.74	1.66	14	26	2	1	2		3	1.5
	?	447.02938	0.78	1.75	14	15	1	4	5	1	1	7.0
	?	447.02930	0.86	1.92	18	17	1	3	1		4	9.0
	?	447.02914	1.02	2.28	13	18	1	1	4	1	6	5.5
	?	447.02911	1.06	2.37	13	16	1	4	4		4	5.5
	?	447.03155	-1.39	-3.10	15	17	1	1	4		6	8.5
	?	447.03179	-1.62	-3.63	16	14	1	4	5		1	10.0
	?	447.03182	-1.66	-3.71	16	16	1	1	5	1	3	10.0
	?	447.03187	-1.70	-3.81	13	18	1	5	2	1	3	2.5
	?	447.03199	-1.82	-4.07	21	15	1	3	2		1	13.5
	?	447.02816	2.00	4.48	21	16	1	2	1		3	13.0
	?	447.02804	2.12	4.75	13	19	1	4	1	1	5	2.0
	?	447.03289	-2.72	-6.09	16	13	1	1	8		2	13.5
	?	447.03289	-2.73	-6.10	17	19	1	1	1		7	8.0
	?	447.03293	-2.76	-6.18	13	15	1	5	5		2	6.0
	?	447.03297	-2.80	-6.27	13	17	1	2	5	1	4	6.0
	?	447.03313	-2.96	-6.63	18	16	1	4	2		2	9.5
469.01187	+	469.00991	1.96	4.19	16	14	1	3	4	1	3	9.5
	?	469.01211	-0.24	-0.52	13	12	1	4	7		3	9.0
	?	469.01215	-0.28	-0.60	13	14	1	1	7	1	5	9.0

Data:24-05
Sample Name:Jan Zelenka [Rol], UK10, M=449
Description:
Ionization Mode:ESI+
History:Determine m/z[Peak Detect[Centroid,30,Area];Correct Base[]];Average(MS[1] 0.22..0.32)-1.0"...

Acquired:24-Aug-18 10:33:08
Operator:Accutof
Mass Calibration data:TFANa_ESI+_1000
Created:24-Aug-18 10:54:11
Created by:Accutof

Charge number:1
Tolerance:5.00(ppm), 3.00 .. (mmu)
Unsaturation Number:-1.5 .. 99.0 (Fraction:Both)
Element:^{12}C:15 .. 100, ^{1}H:1 .. 100, ^{19}F:1 .. 5, ^{14}N:1 .. 10, ^{23}Na:0 .. 1, ^{16}O:1 .. 10

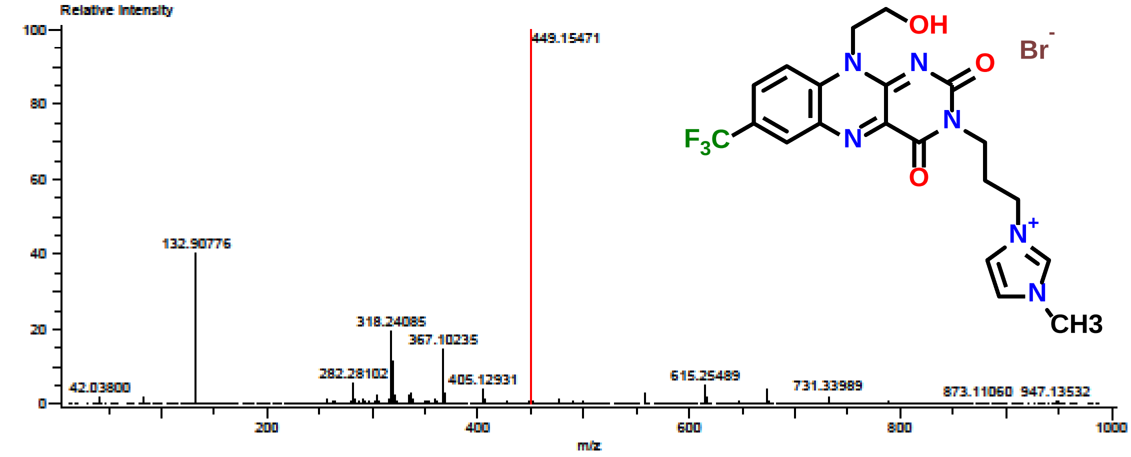

Mass	Decision	Calc. Mass	Mass Difference (mmu)	Mass Difference (ppm)	^{12}C	^{1}H	^{19}F	^{14}N	^{23}Na	^{16}O	Unsaturation Number
449.15471	+	449.15490	-0.18	-0.41	20	20	3	6		3	12.5
	?	449.15470	0.01	0.03	16	25	4	2		8	3.5
	?	449.15470	0.02	0.04	15	19	4	9		3	9.0
	?	449.15474	-0.02	-0.05	15	21	1	9	1	5	9.0
	?	449.15474	-0.03	-0.06	16	27	1	2	1	10	3.5
	?	449.15498	-0.26	-0.59	17	24	4	3	1	5	5.0
	?	449.15510	-0.38	-0.85	25	21	2	3		3	16.0
	?	449.15395	0.76	1.69	28	20	1	3		2	20.0
	?	449.15383	0.88	1.96	20	23	3	3	1	4	9.0
	?	449.15375	0.96	2.14	23	19	2	6		2	16.5
	?	449.15363	1.08	2.40	15	22	4	6	1	4	5.5
	?	449.15580	-1.09	-2.43	16	24	1	5		9	7.0
	?	449.15356	1.15	2.57	19	24	3	2		7	7.5
	?	449.15355	1.16	2.58	18	18	3	9		2	13.0
	?	449.15604	-1.33	-2.95	17	21	4	6		4	8.5
	?	449.15608	-1.36	-3.04	17	23	1	6	1	6	8.5
	?	449.15624	-1.53	-3.40	22	22	3	3		4	12.0
	?	449.15632	-1.60	-3.56	18	20	4	7	1	1	10.0
	?	449.15651	-1.80	-4.01	23	21	3	4	1	1	13.5
	?	449.15671	-2.00	-4.45	28	22	2	1	1	1	17.0
	?	449.15269	2.02	4.50	23	22	2	3	1	3	13.0
	?	449.15265	2.06	4.59	23	20	5	3		1	13.0
	?	449.15261	2.10	4.68	26	18	1	6		1	20.5
	?	449.15249	2.22	4.95	18	21	3	6	1	3	9.5
	?	449.15242	2.30	5.12	22	23	2	2		6	11.5
	?	449.15241	2.30	5.13	21	17	2	9		1	17.0
	?	449.15714	-2.43	-5.40	17	20	1	9		5	12.0
	?	449.15715	-2.43	-5.42	18	26	1	2		10	6.5
	?	449.15222	2.50	5.56	17	22	3	5		6	8.0
	?	449.15738	-2.67	-5.94	19	23	4	3		5	8.0
	?	449.15742	-2.70	-6.02	18	19	1	10	1	2	13.5
	?	449.15742	-2.71	-6.03	19	25	1	3	1	7	8.0
	?	449.15746	-2.74	-6.11	15	21	5	7	1	2	6.0
	?	449.15766	-2.94	-6.55	20	22	4	4	1	2	9.5

Data:24-06
Sample Name:Jan Zelenka [RoI], UK30, M=412
Description:
Ionization Mode:ESI+
History:Determine m/z[Peak Detect[Centroid,30,Area];Correct Base[]];Average(MS[1] 0.22..0.29)-1.0"...

Acquired:24-Aug-18 10:35:48
Operator:Accutof
Mass Calibration data:TFANa_ESI+_1000
Created:24-Aug-18 10:57:38
Created by:Accutof

Charge number:1
Tolerance:5.00(ppm), 3.00 .. (mmu)
Unsaturation Number:-1.5 .. 99.0 (Fraction:Both)
Element:^{12}C:15 .. 100, ^{1}H:1 .. 100, ^{19}F:1 .. 5, ^{14}N:1 .. 10, ^{23}Na:0 .. 1, ^{16}O:1 .. 10

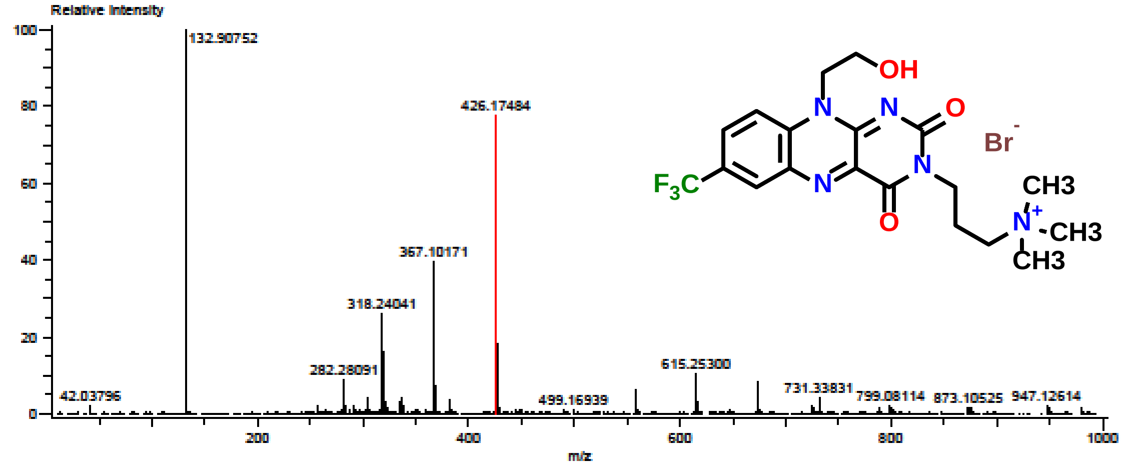

Mass	Decision	Calc. Mass	Mass Difference (mmu)	Mass Difference (ppm)	^{12}C	^{1}H	^{19}F	^{14}N	^{23}Na	^{16}O	Unsaturation Number
426.17484	+	426.17530	-0.46	-1.08	19	23	3	5		3	9.5
	?	426.17510	-0.27	-0.63	15	28	4	1		8	0.5
	?	426.17514	-0.31	-0.72	15	30	1	1	1	10	0.5
	?	426.17436	0.48	1.13	27	23	1	2		2	17.0
	?	426.17538	-0.54	-1.27	16	27	4	2	1	5	2.0
	?	426.17424	0.60	1.41	19	26	3	2	1	4	6.0
	?	426.17550	-0.66	-1.55	24	24	2	2		3	13.0
	?	426.17416	0.68	1.60	22	22	2	5		2	13.5
	?	426.17396	0.88	2.05	18	27	3	1		7	4.5
	?	426.17396	0.88	2.07	17	21	3	8		2	10.0
	?	426.17621	-1.37	-3.21	15	27	1	4		9	4.0
	?	426.17644	-1.60	-3.77	16	24	4	5		4	5.5
	?	426.17648	-1.64	-3.86	16	26	1	5	1	6	5.5
	?	426.17309	1.74	4.09	22	25	2	2	1	3	10.0
	?	426.17305	1.78	4.18	22	23	5	2		1	10.0
	?	426.17664	-1.80	-4.23	21	25	3	2		4	9.0
	?	426.17301	1.82	4.28	25	21	1	5		1	17.5
	?	426.17672	-1.88	-4.41	17	23	4	6	1	1	7.0
	?	426.17289	1.94	4.56	17	24	3	5	1	3	6.5
	?	426.17282	2.02	4.74	21	26	2	1		6	8.5
	?	426.17281	2.02	4.75	20	20	2	8		1	14.0
	?	426.17692	-2.08	-4.88	22	24	3	3	1	1	10.5
	?	426.17262	2.22	5.21	16	25	3	4		6	5.0
	?	426.17754	-2.71	-6.35	16	23	1	8		5	9.0
	?	426.17755	-2.71	-6.36	17	29	1	1		10	3.5
	?	426.17195	2.89	6.77	25	24	1	2	1	2	14.0
	?	426.17778	-2.95	-6.92	18	26	4	2		5	5.0
	?	426.17782	-2.98	-7.00	17	22	1	9	1	2	10.5
	?	426.17782	-2.99	-7.01	18	28	1	2	1	7	5.0

Data:25-02
Sample Name:Jan Zelenka [JR], MZ99
Description:
Ionization Mode:ESI+
History:Determine m/z[Peak Detect[Centroid,30,Area];Correct Base[]];Average(MS[1] 0.25..0.30)-1.0*Average(MS[1] 0.05..0.12);Correct Base[5....

Acquired:25-Oct-18 09:57:56
Operator:Accutof
Mass Calibration data:TFANa_ESI+_1000
Created:25-Oct-18 10:00:15
Created by:Accutof

Charge number:1
Tolerance:5.00(ppm), 3.00 .. (mmu)
Unsaturation Number:-1.5 .. 99.0 (Fraction:Both)
Element:^{12}C:17 .. 46, ^{1}H:1 .. 100, ^{79}Br:0 .. 3, ^{19}F:3 .. 3, ^{14}N:1 .. 20, ^{16}O:1 .. 20

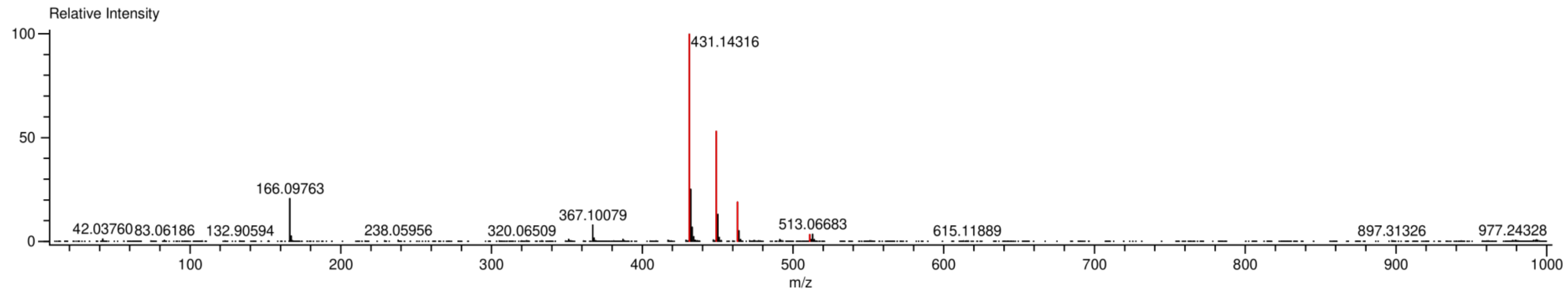

Mass	Decision	Calc. Mass	Mass Difference (mmu)	Mass Difference (ppm)	^{12}C	^{1}H	^{79}Br	^{19}F	^{14}N	^{16}O	Unsaturation Number
431.14316	+	431.14433	-1.17	-2.71	20	18		3	6	2	13.5
	?	431.14300	0.17	0.39	19	22		3	2	6	8.5
	?	431.14299	0.17	0.40	18	16		3	9	1	14.0
	?	431.14165	1.51	3.50	17	20		3	5	5	9.0
	?	431.14568	-2.51	-5.83	22	20		3	3	3	13.0
449.15286	+	449.15490	-2.03	-4.53	20	20		3	6	3	12.5
	?	449.15222	0.65	1.44	17	22		3	5	6	8.0
	?	449.15355	-0.69	-1.54	18	18		3	9	2	13.0
	?	449.15356	-0.70	-1.55	19	24		3	2	7	7.5
	?	449.15411	-1.25	-2.78	21	31	1	3	1	1	5.0
463.16831	+	463.17055	-2.24	-4.84	21	22		3	6	3	12.5
	?	463.16787	0.44	0.95	18	24		3	5	6	8.0
	?	463.16786	0.44	0.96	17	18		3	12	1	13.5
	?	463.16920	-0.90	-1.94	19	20		3	9	2	13.0
	?	463.16921	-0.90	-1.95	20	26		3	2	7	7.5
	?	463.16976	-1.45	-3.14	22	33	1	3	1	1	5.0
	?	463.16653	1.78	3.84	17	28		3	1	10	3.0
	?	463.16574	2.57	5.55	17	33	1	3	3	3	1.0
511.06862	+	511.07050	-1.88	-3.67	20	19	1	3	6	2	12.5
	?	511.06861	0.01	0.02	17	16		3	3	12	10.0
	?	511.06809	0.52	1.03	30	8		3	5	1	28.0
	?	511.06915	-0.53	-1.04	18	17	1	3	9	1	13.0
	?	511.06916	-0.54	-1.05	19	23	1	3	2	6	7.5
	?	511.06782	0.80	1.57	17	21	1	3	5	5	8.0
	?	511.06944	-0.82	-1.60	32	10		3	2	2	27.5
	?	511.06994	-1.32	-2.58	17	6		3	14	3	20.5
	?	511.06994	-1.33	-2.59	18	12		3	7	8	15.0

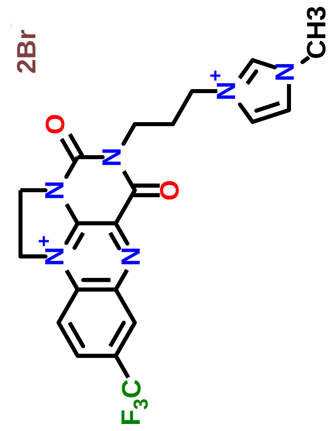

On the request of Reviewer 1, we list unrelated studies from other laboratories that applied helium tagging photodissociation technique:

1. Asmis group:

Gas phase infrared spectroscopy of mono- and divanadium oxide cluster cations

Asmis, KR; Meijer, G; Brummer, M; Kaposta, C; Santambrogio, G; Woste, L; Sauer, J.; *J. Chem. Phys.* **2004**, *120*, 6461-6470.

Structure and Chemistry of the Heteronuclear Oxo-Cluster [VPO4](center dot+): A Model System for the Gas-Phase Oxidation of Small Hydrocarbons

Dietl, N; Wende, T; Chen, K; Jiang, L; Schlangen, M; Zhang, XH; Asmis, KR; Schwarz, H., *J. Am. Chem. Soc.* **2013**, *135*, 3711-3721.

Note: Probably more papers on metal oxide clusters using helium tagging spectroscopy originated from this group, but we are not aware of them.

2. Maier group: this groups has many papers on astrochemically relevant ions (fullerenes of PAHs) using this approach. We give just one example, the interested reader can trace the rest as papers citing this first article.

Laboratory confirmation of C-60(+) as the carrier of two diffuse interstellar bands

Campbell, EK; Holz, M; Gerlich, D; Maier, JP, *Nature*, 523, 322.

3. Schlemmer group: this groups has also a series of papers on astrochemically relevant ions using this approach. We give just one example, the interested reader can trace the rest as papers citing this first article.

COLTRAP: a 22-Pole Ion Trapping Machine for Spectroscopy at 4 K.

Asvany, O.; Brunken, S.; Kluge, L.; Schlemmer, S. *Appl. Phys. B: Lasers Opt.* **2014**, *114*, 203−211.

4. Johnson group:

He-tagged vibrational spectra of the SarGlyH(+) and H+(H2O)(2,3) ions: Quantifying tag effects in cryogenic ion vibrational predissociation (CIVP) spectroscopy

Johnson, CJ; Wolk, AB; Fournier, JA; Sullivan, EN; Weddle, GH; Johnson, MA; J. *J. Chem. Phys.* **2014**, *140*, 221101.

5. Chen group:

A 4 K FT-ICR cell for infrared ion spectroscopy

Fritsche, L; Bach, A; Miloglyadova, L; Tsybizova, A; Chen, P., Rev. Scientific Intrum. 2018, 89, 063119.

6. Rizzo group:

Cryogenic Ion Spectroscopy for Identification of Monosaccharide Anomers

Scutelnic, V; Rizzo, TR; *J. Phys. Chem. A* **2019**, *123*, 2815-2819.

We do not guarantee that there is no other work using helium tagging spectroscopy in a trap. However, there is an ample of works using tagging with different rare atoms or small inert molecules such as H_2 or D_2 (e.g. see publications from the Johnson group). Please note, that in most of the cases, the results obtained are analogous. The noticeable difference concerns highly reactive ions. Several groups published papers on this topic. Also, helium tagged ions can be produced from helium droplets. This is a large field with many active groups.

References:

[1] For synthesis of **1a** and **S1** see: J. Zelenka, E. Svobodová, J. Tarábek, I. Hoskovcová, V. Boguschová, S. Bailly, M. Sikorski, J. Roithová, R. Cibulka, *Org. Lett.* **2019**, *21*, 114–119; For synthesis of methyl analogue see: W.-S. Li, N. Zhang, L. M. Sayre, Tetrahedron **2001**, *57*, 4507–4522.

[2] Detection of Fleeting Amine Radical Cations and Elucidation of Chain Processes in Visible-Light-Mediated [3 + 2] Annulation by Online Mass Spectrometric Techniques Y. Cai, J. Wang, Y. Zhang, Z. Li, D. Hu, N. Zheng, and H. Chen, *J. Am. Chem. Soc.* **2017**, *139*, 12259-12266.

[3] Picomole - Scale Real - Time Photoreaction Screening: Discovery of the Visible - Light - Promoted Dehydrogenation of Tetrahydroquinolines under Ambient Conditions S. Chen, Q. Wan, A. K. Badu-Tawiah, *Angew. Chem. Int. Ed.* **2016**, *55*, 9345-9349

[4] a) K. L. Vikse, M. P. Woods, J. S. McIndoe, *Organometallics* **2010**, *29*, 6615–6618. b) K. L. Vikse, Z. Ahmadi, J. Luo, N. van der Wal, K. Daze, N. Taylor, J. S. McIndoe, *Int. J. Mass Spectrom*. **2012**, *323–324*, 8–13. c) X. Yan, E. Sokol, X. Li, G. Li, S. Xu, R. G. Cooks, *Angew. Chem. Int. Ed.* **2014**, *53*, 5931–5935.
